# Supplementary material for: Divergent synthesis and identification of the cellular targets of deoxyelephantopins
Source: Nat Commun. 2016 Aug 19;7:12470. doi: 10.1038/ncomms12470 (PMC4992173; doi:10.1038/ncomms12470)
Supplement: Supplementary Information — Supplementary Figures 1-98 and Supplementary Methods. [file ncomms12470-s1.pdf]

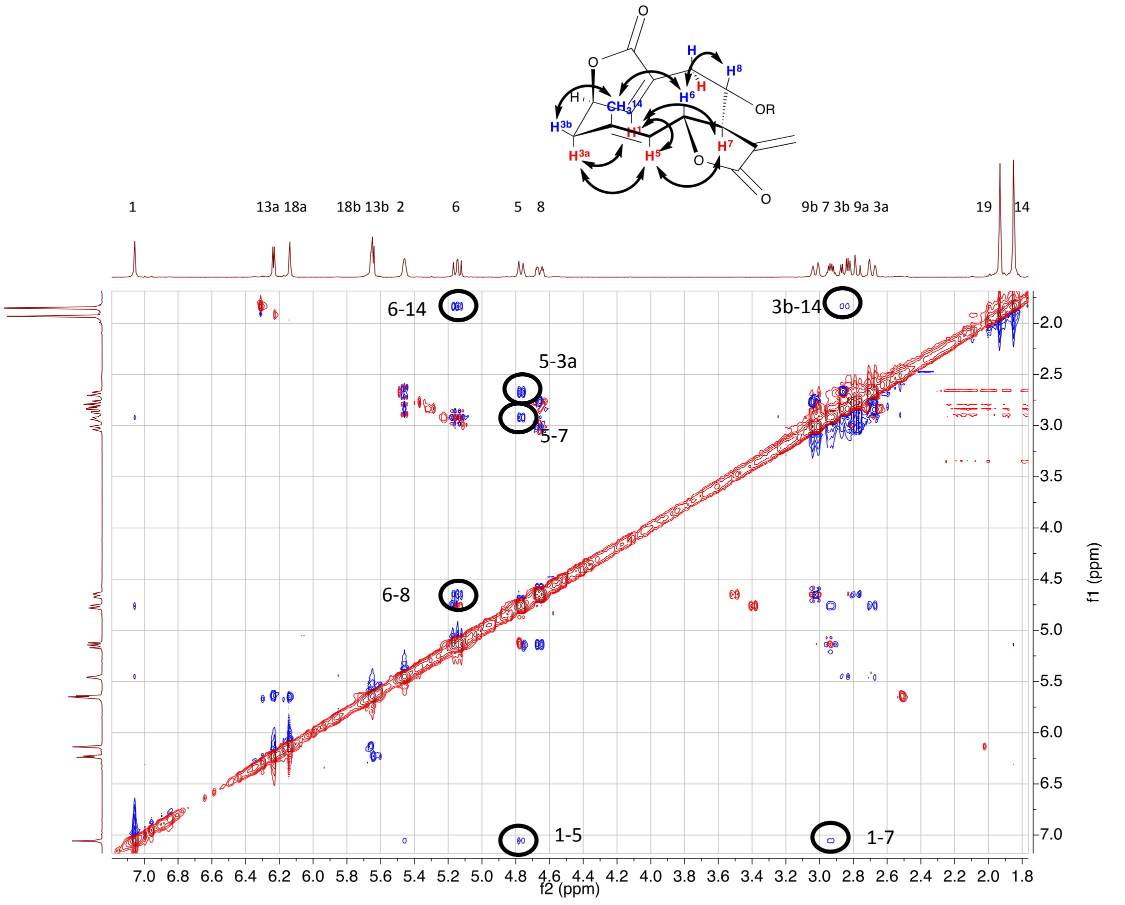

Supplementary figure 1: NOESY spectrum of Deoxyelephantopin.

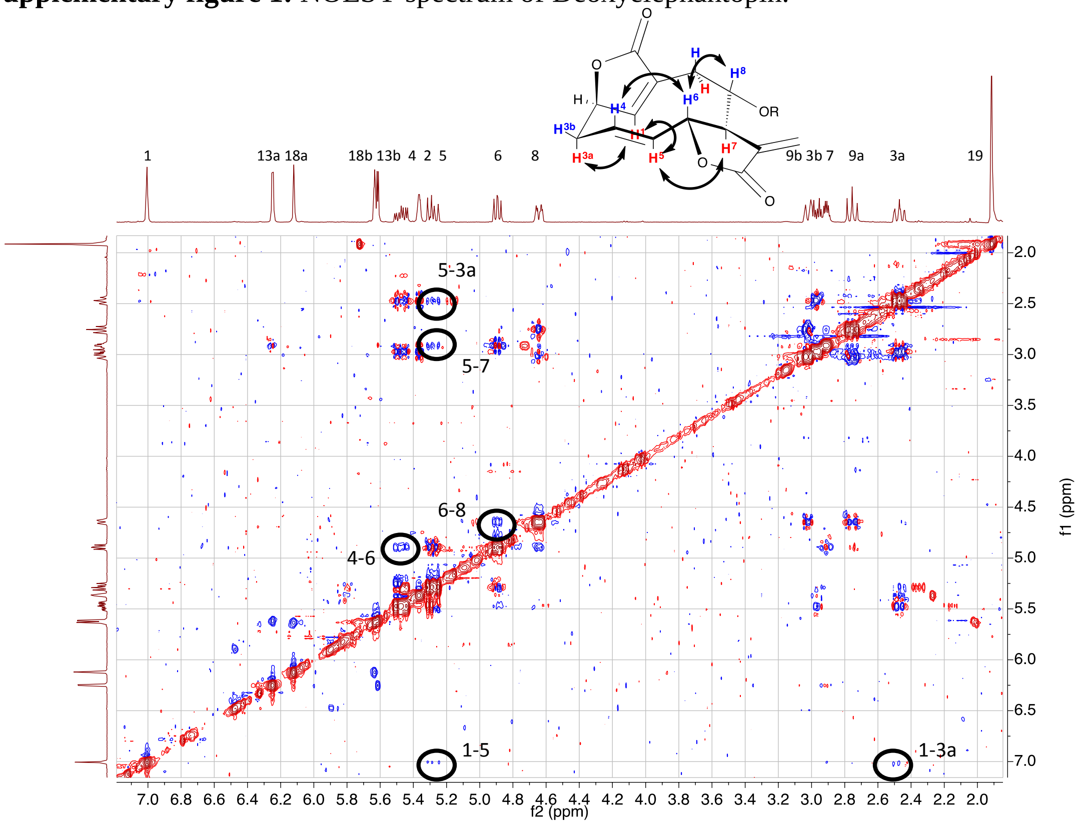

Supplementary figure 2: NOESY spectrum of Nordeoxyelephantopin.

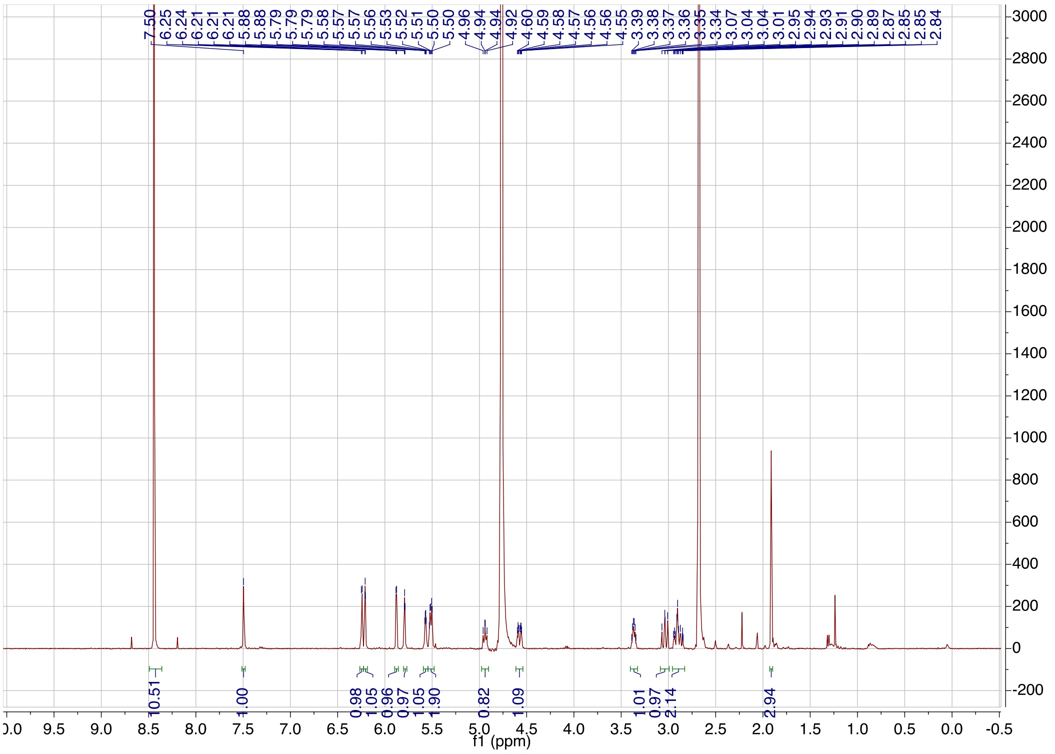

Supplementary figure 3. ^{1}H NMR spectrum of Nordeoxyelephantopin prior to GSH addition

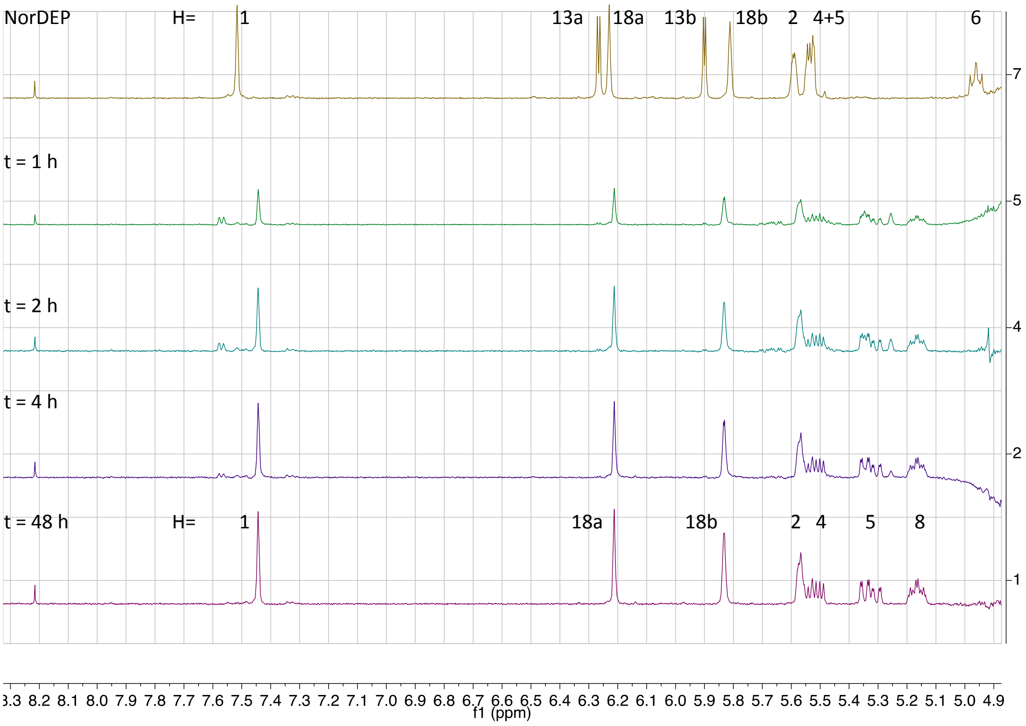

Supplementary figure 4: Overlay of ^{1}H NMR spectra at different time points.

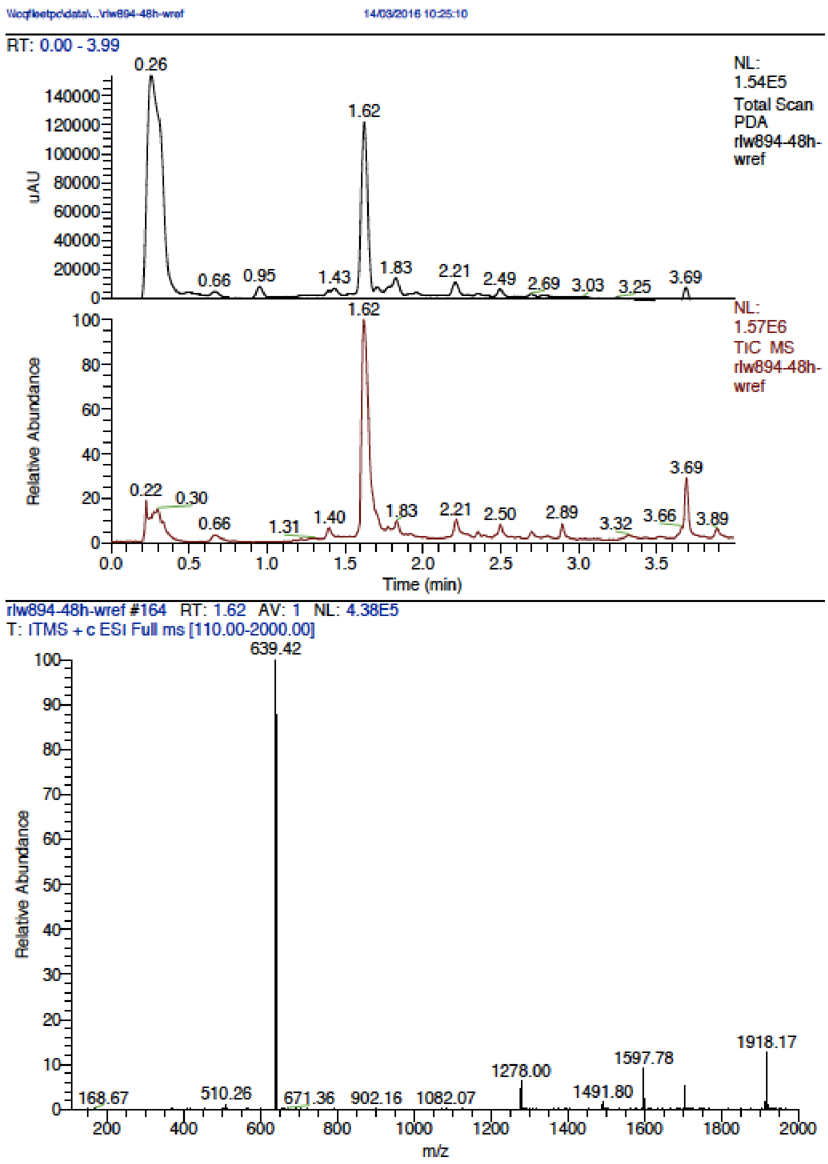

Supplementary figure 5. LC-MS chromatogram of the crude reaction mixture after 48 h. **LC-MS** (H_2O:MeCN, 0.1% TFA) m/z calculated for $C_{28}H_{35}DN_3O_{12}S^+$ $[M+H]^+$: 639.21; found: 639.42.

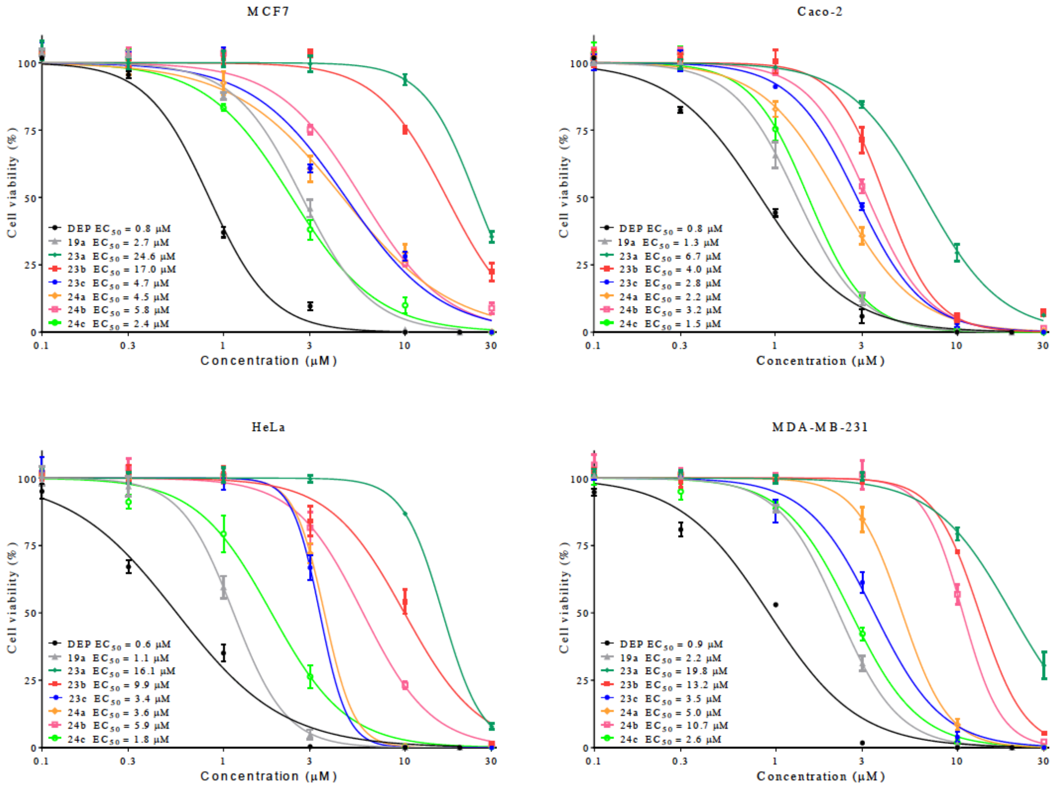

Supplementary figure 6: EC_{50} curves for indicated compounds in four different cell lines. Cells were treated for 3 days. (values±SD are shown; *n* = 3).

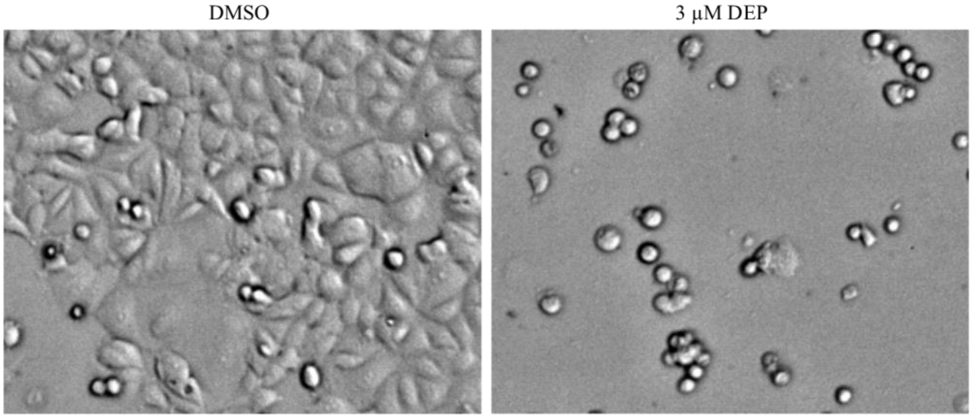

Supplementary figure 7: Microscopy images of MCF7 cells treated with 3 µM deoxyelephantopin (DEP) or DMSO as control.

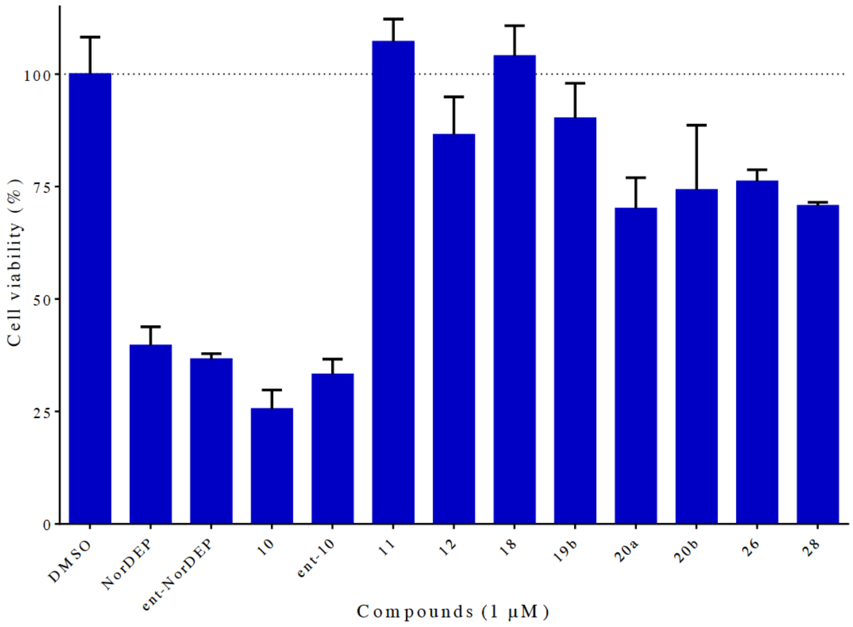

Supplementary figure 8: Cell viability measurements in MCF7 cells. All compounds were tested at 1 µM concentration for 3 days (values±SD are shown; *n* = 3).

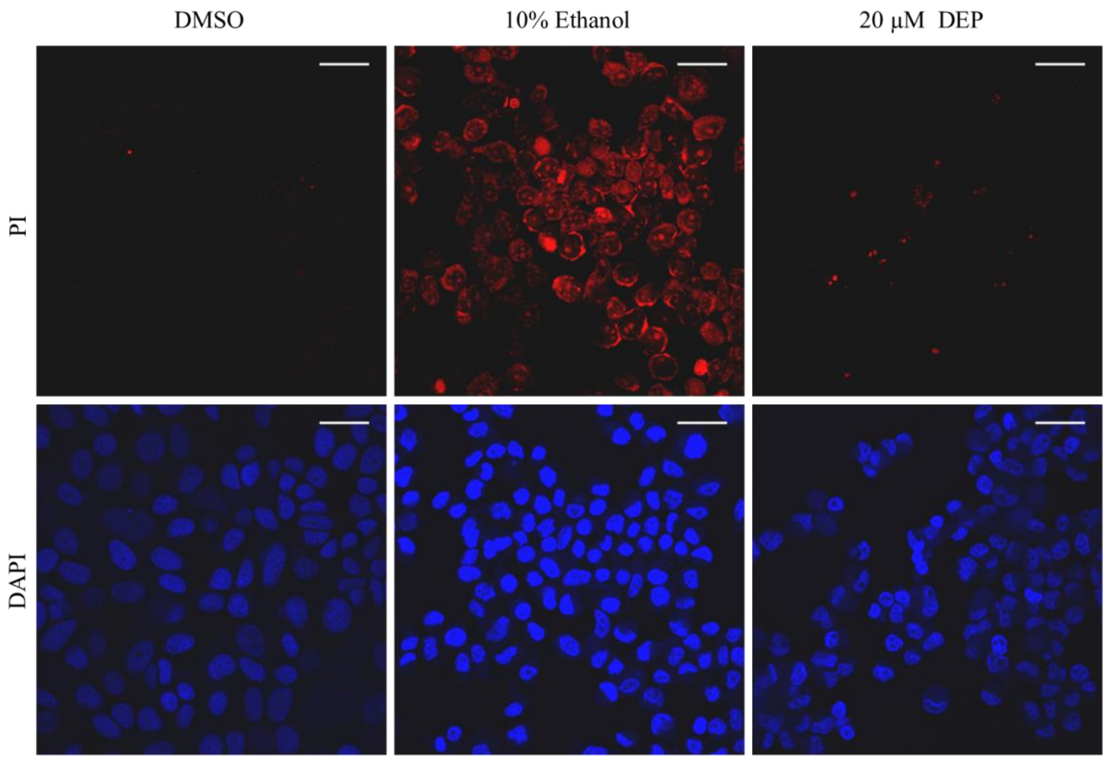

Supplementary figure 9: Propidium iodide and DAPI staining of MCF7 cells treated with either DMSO, 10% ethanol (necrosis positive control) or 20 µM DEP for 8 h. Scale bar represents 30 µm.

.

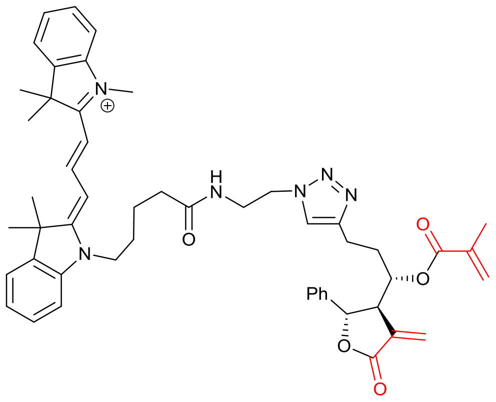

Supplementary figure 10: Chemical structure of fluorescent probe **24c-Cy3**.

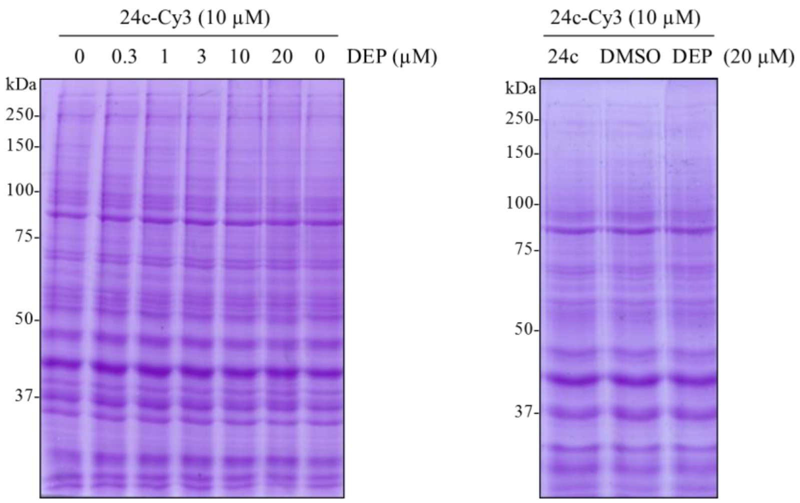

Supplementary figure 11: Coomassie stained gels corresponding to the fluorescence gels in the main text Figures 6a and 6b.

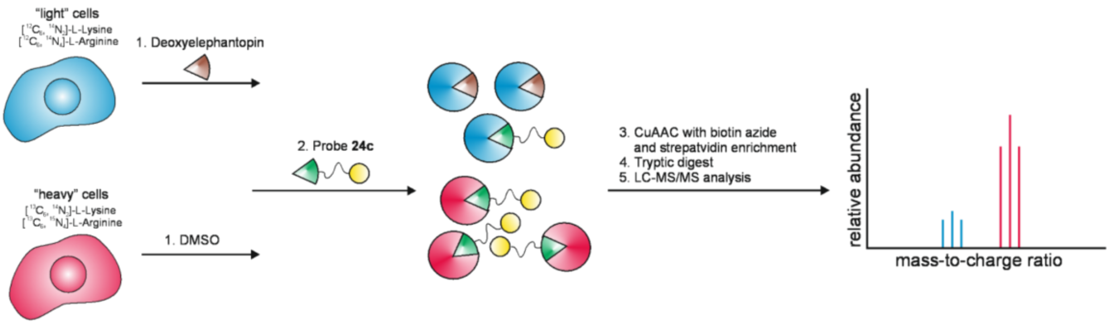

Protein name	Gene name	Mol. weight [kDa]	SILAC ratio Deoxyelephantopin/DMSO	SD
Thioredoxin doma in-containing protein 12	TXNDC12	19.21	0.12	0.010
Protein HGH1 homolog	HGH1	42.13	0.20	0.012
Zinc finger protein 346	ZNF346	32.93	0.22	0.004
Glyoxylate reductase/hydroxypyruvate reductase	GRHPR	38.70	0.23	0.020
Cystatin-B	CSTB	11.14	0.23	0.009
Cystathionine beta-synthase	CBS	60.59	0.24	0.079
Heme oxygenase 2	HMOX2	36.03	0.24	0.001
Src substrate cortactin	CTTN	61.59	0.25	0.008
Protein transport protein Sec24C	SEC24C	111.98	0.26	0.031
Nucleoporin p54	NUP54	50.78	0.27	0.035
FLYWCH family member 2	FLYWCH2	15.60	0.29	0.018

Supplementary figure 12: Top: Experimental workflow for the competitive proteomic profiling of DEP targets in SILAC-labeled MCF7 cells. Bottom: Proteomic targets of DEP (>70% competition) in MCF7 lysates identified in a SILAC experiment (n = 2).

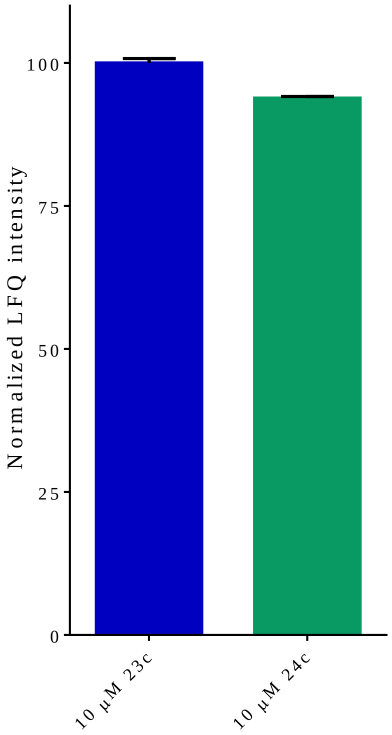

Supplementary figure 13: LFQ quantification of PPARγ enrichment from supplemented MCF7 lysates using 10 µM of **23c** or **24c** as enrichment probes (*n* = 3).

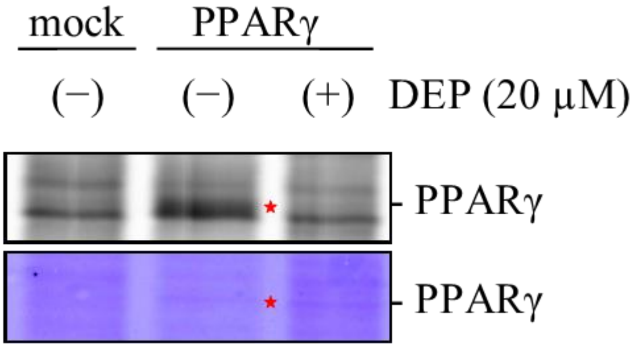

Supplementary figure 14: *In situ* competition of overexpressed PPARγ in HeLa cells. Cells were treated with either DMSO or 20 µM DEP for 4 h, lysed and treated with 10 µM **24c-Cy3**. Fluorescence gel is shown on the top and Coomassie stain on the bottom. Asterisk indicate PPARγ.

GO term	Description	P-value	Genes
GO:0034308	primary alcohol metabolic process	9.71E-05	ALDH3B1 - aldehyde dehydrogenase 3 family, member b1<br>TTR - transthyretin<br>RBP4 - retinol binding protein 4, plasma
GO:0042572	retinol metabolic process	1.80E-04	TTR - transthyretin<br>RBP4 - retinol binding protein 4, plasma
GO:0046621	negative regulation of organ growth	2.35E-04	RBP4 - retinol binding protein 4, plasma<br>STK4 - serine/threonine kinase 4
GO:1901685	glutathione derivative metabolic process	2.68E-04	GSTM3 - glutathione s-transferase mu 3 (brain)<br>GSTA2 - glutathione s-transferase alpha 2<br>GSTA1 - glutathione s-transferase alpha 1
GO:1901687	glutathione derivative biosynthetic process	2.68E-04	GSTM3 - glutathione s-transferase mu 3 (brain)<br>GSTA2 - glutathione s-transferase alpha 2<br>GSTA1 - glutathione s-transferase alpha 1
GO:0048640	negative regulation of developmental growth	6.99E-04	RBP4 - retinol binding protein 4, plasma<br>STK4 - serine/threonine kinase 4
GO:1901615	organic hydroxy compound metabolic process	7.09E-04	ALDH3B1 - aldehyde dehydrogenase 3 family, member b1<br>TTR - transthyretin<br>RBP4 - retinol binding protein 4, plasma<br>LIPA - lipase a, lysosomal acid, cholesterol esterase<br>CROT - carnitine o-octanoyltransferase
GO:0001523	retinoid metabolic process	8.75E-04	TTR - transthyretin<br>RBP4 - retinol binding protein 4, plasma

GO term	Description	P-value	Genes
GO:0048565	digestive tract development	3.61E-04	VPS52 - vacuolar protein sorting 52 homolog (s. cerevisiae)<br>ITGB4 - integrin, beta 4

Supplementary figure 15: GO terms pathway analysis of downregulated (top) and upregulated (bottom) proteins with PPARγ antagonist T0070907 and DEP using GOrilla.

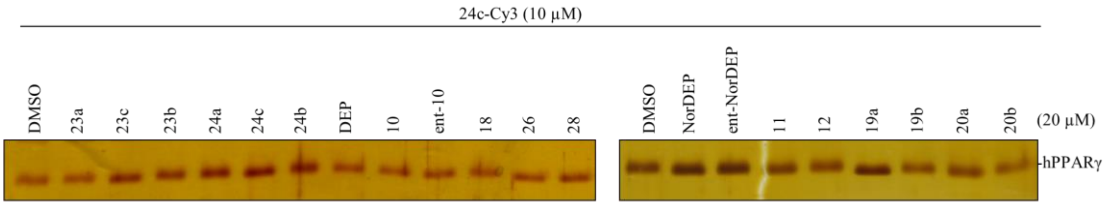

Supplementary figure 16: Silver stain images corresponding to the fluorescence gels in the main text Figure 7c.

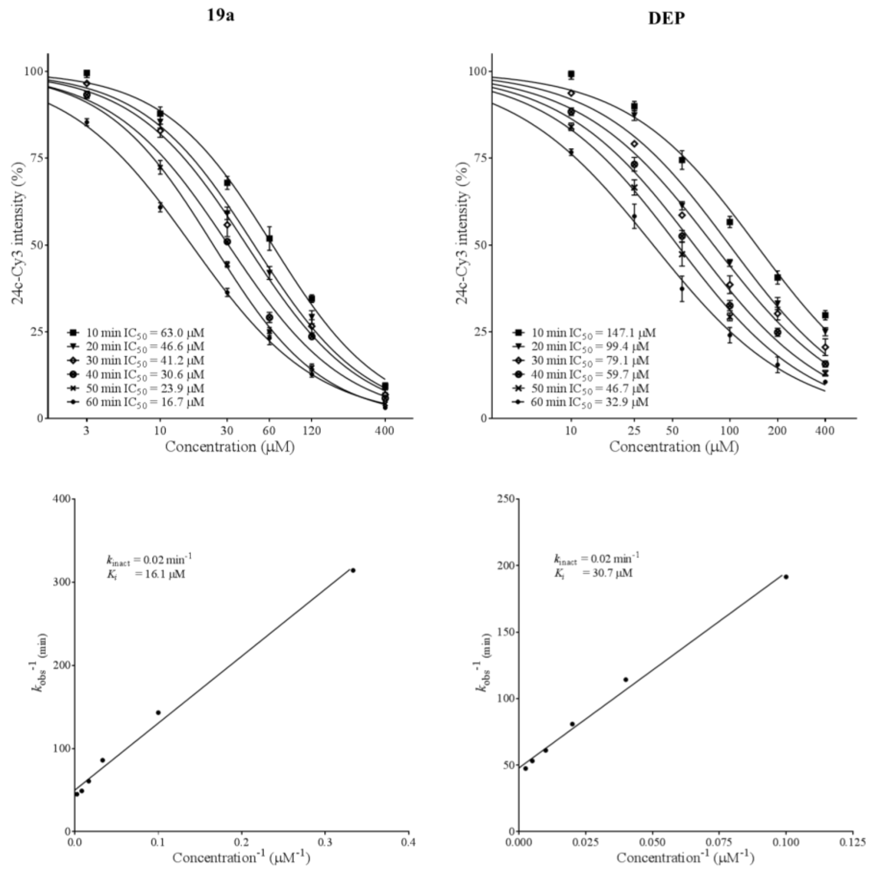

Supplementary figure 17: Determination of the kinetic values K_i and k_{inact} for **19a** and DEP as binders to human PPARγ (n = 3).

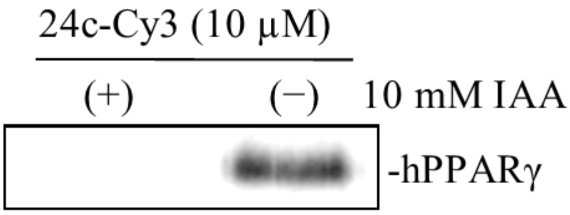

Supplementary figure 18: Fluorescence labeling of recombinant human PPARγ with probe **24c-Cy3** after pretreatment with 10 mM IAA or DMSO.

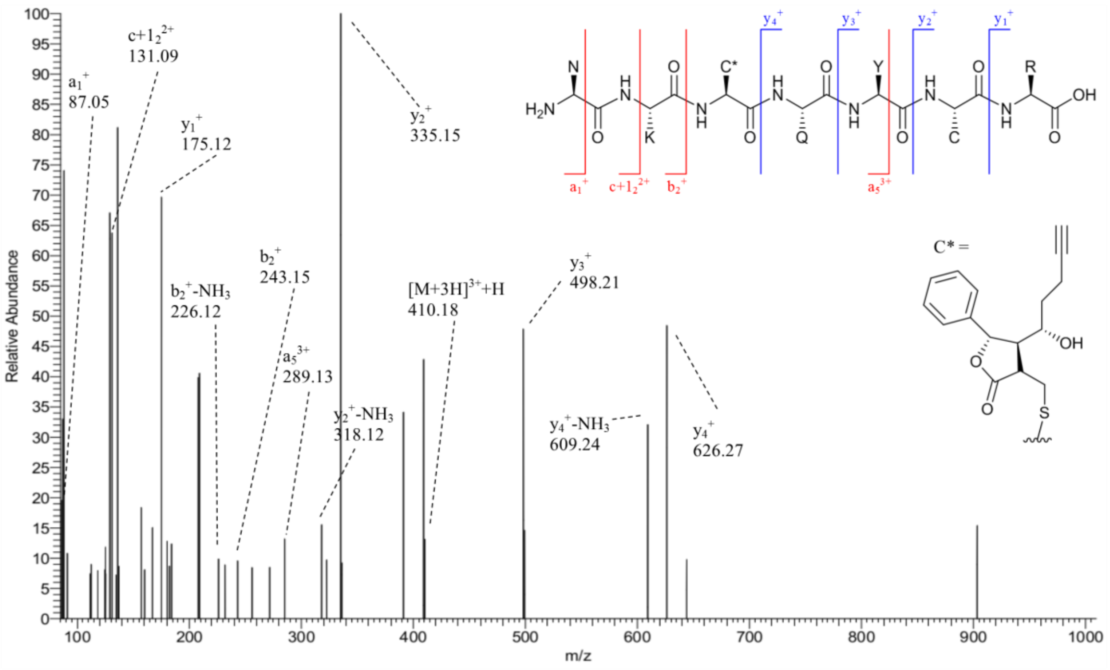

Supplementary figure 19: Annotated MS^2 spectrum of the PPARγ tryptic peptide with Cys190 (C*) covalently bound to compound **23c**.

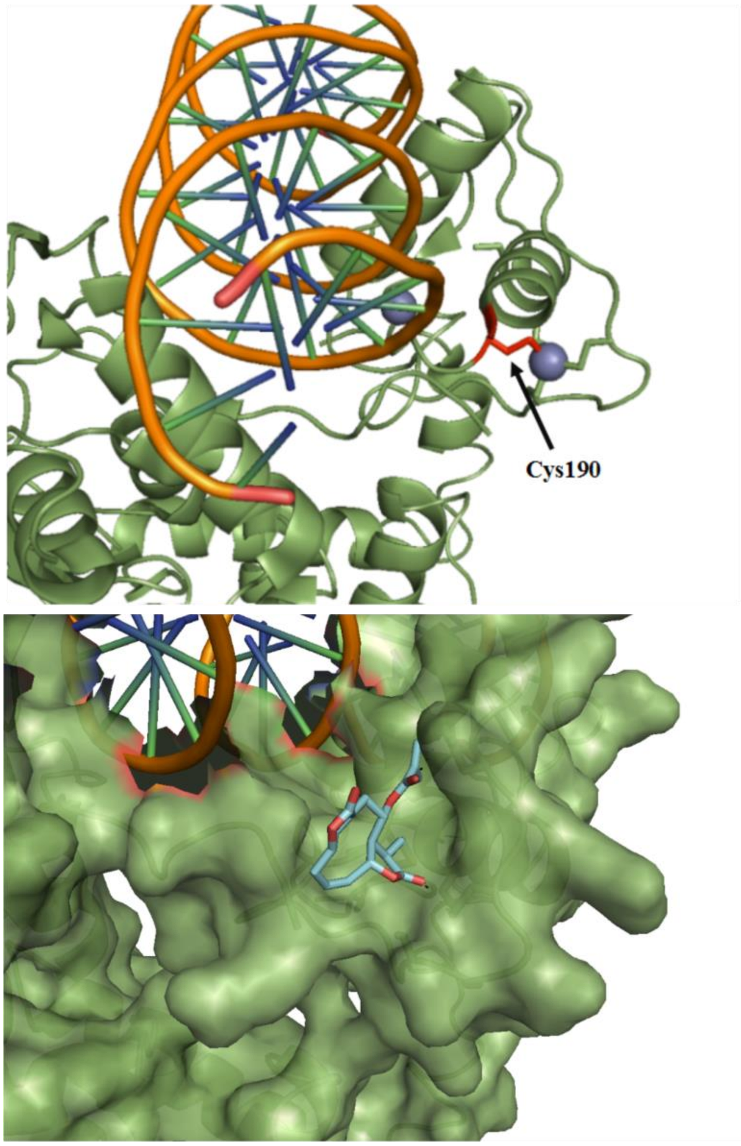

Supplementary figure 20: Top: A cartoon representation of human PPARγ structure (PDB: 3DZU) with the Zn^{2+}-bound Cys190 shown in red. Bottom: Surface view of human PPARγ with **19a** covalently bound to Cys190.

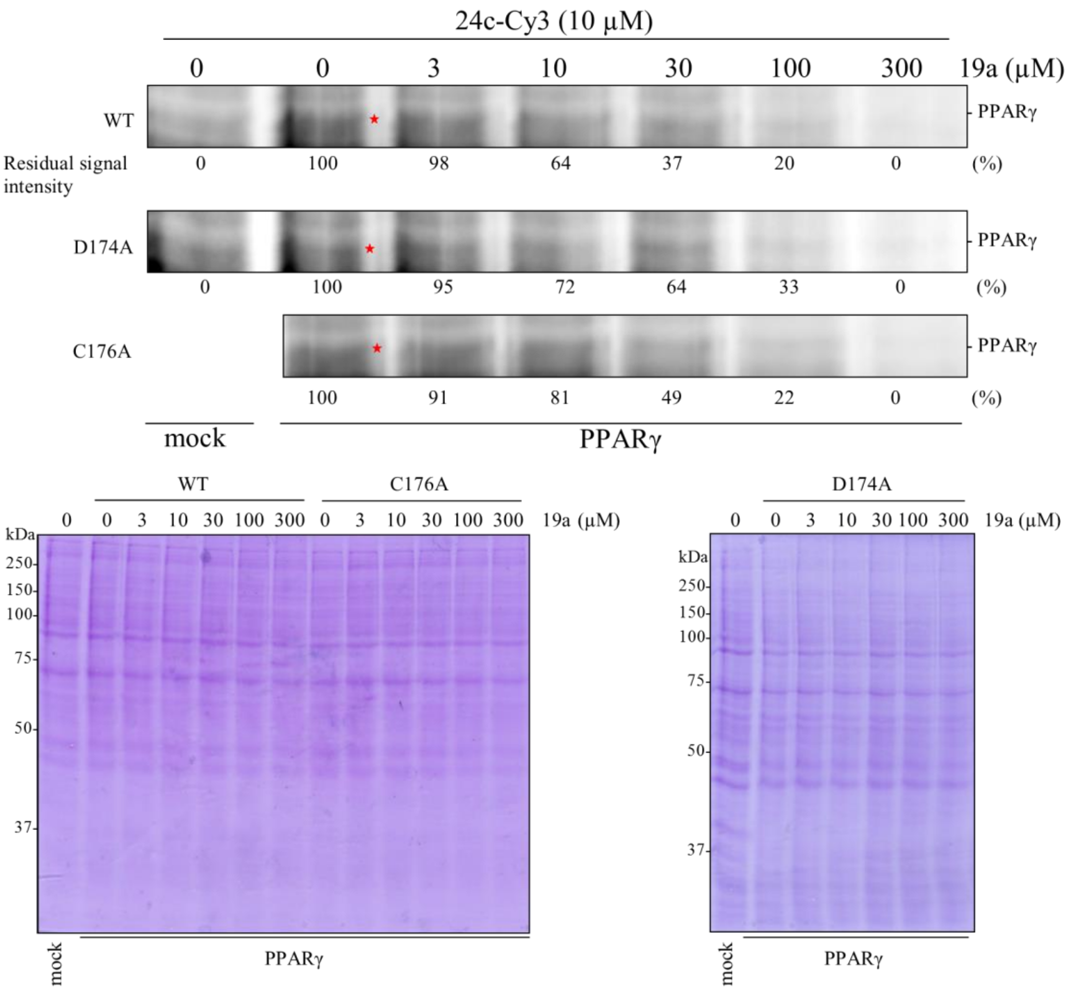

Supplementary figure 21: Top: Fluorescence gels corresponding to the IC_{50} curves presented in the main text Figure 8b. Values indicated below the gels show remaining PPARγ fluorescence signal in percentage after **19a** treatment. Asterisks indicate the PPARγ band. Bottom: Corresponding Coomassie stained gels.

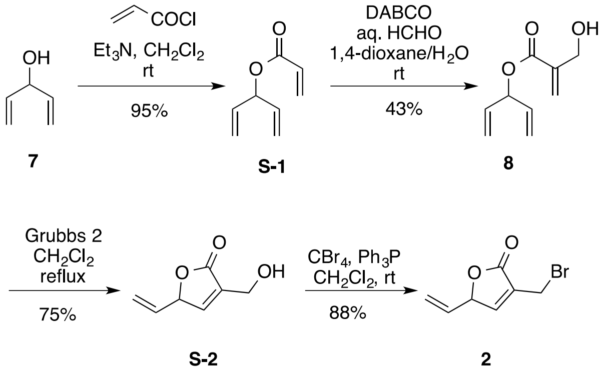

Supplementary figure 22: Synthesis of bromolactone **2**

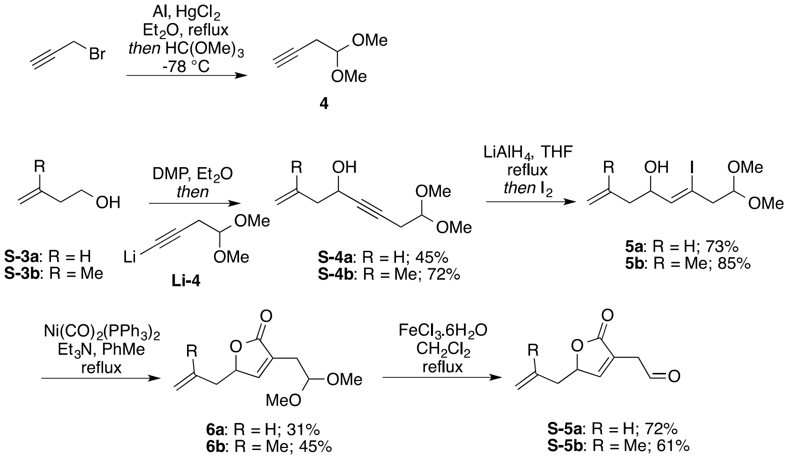

Supplementary figure 23: Racemic synthesis of butenolide **6**

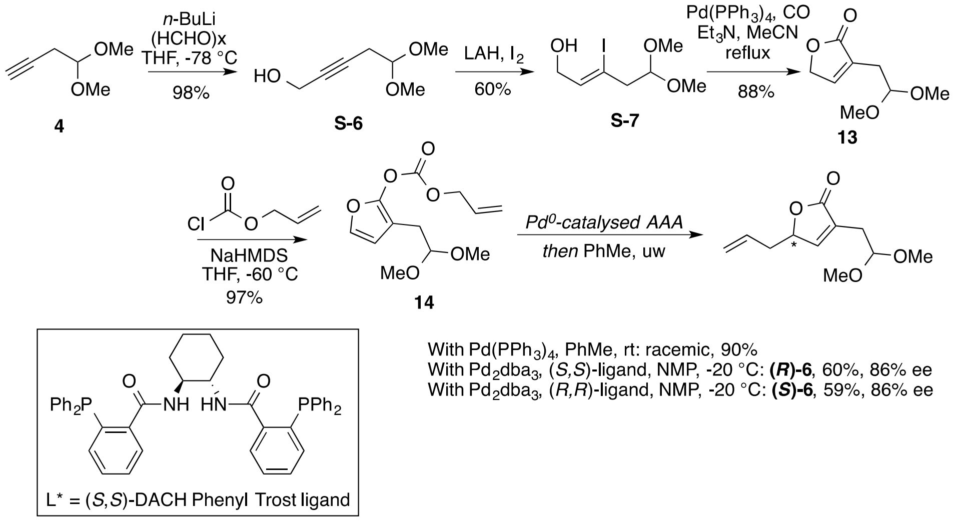

Supplementary figure 24: Asymmetric synthesis of butenolide **6**

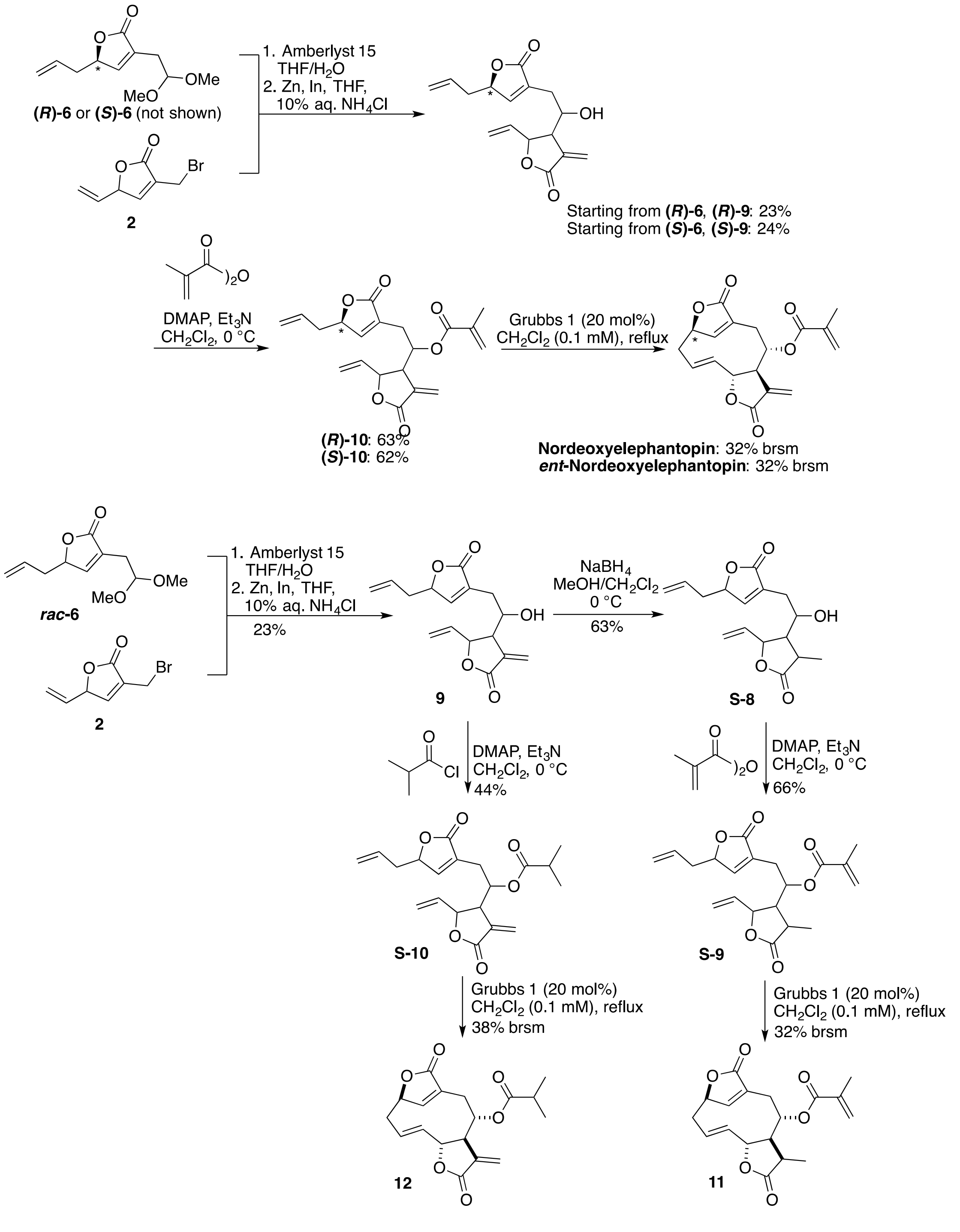

Supplementary figure 24: Synthesis of Nordeoxyelephantopins and compounds **11** and **12**

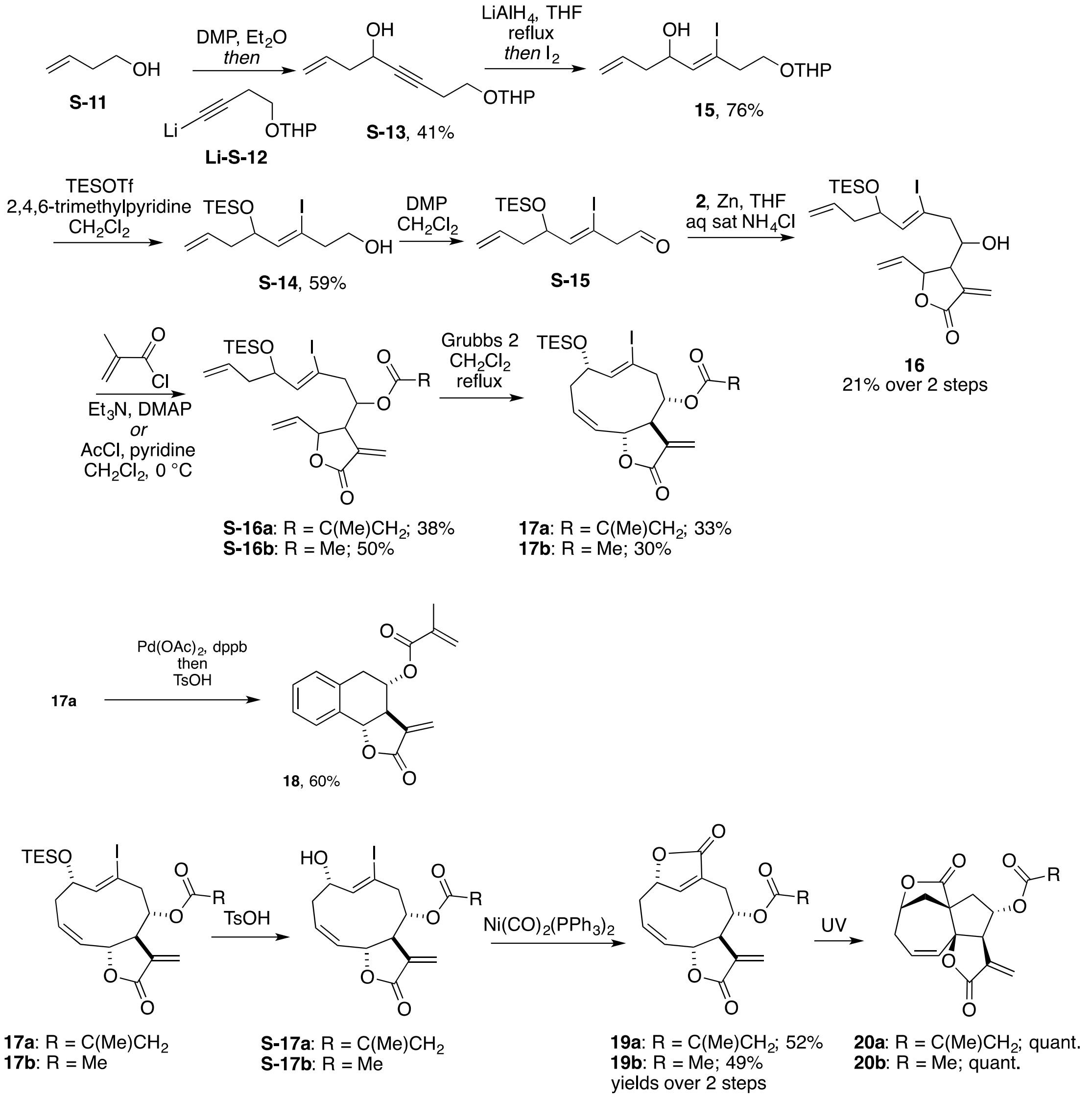

Supplementary figure 25: Synthesis of compounds **18-20**

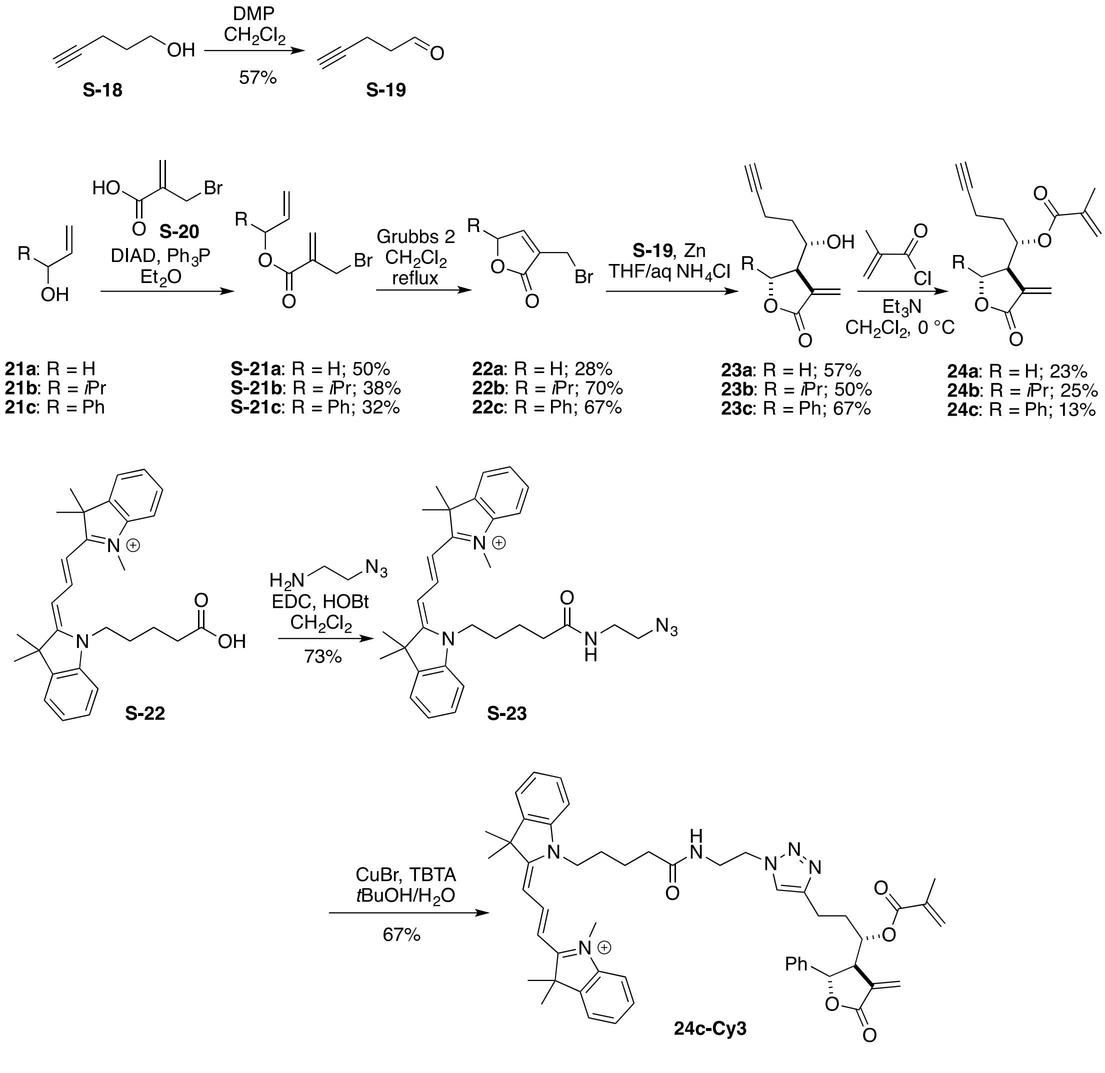

Supplementary figure 26: Synthesis of compounds **24a-c** and **24c-Cy3**

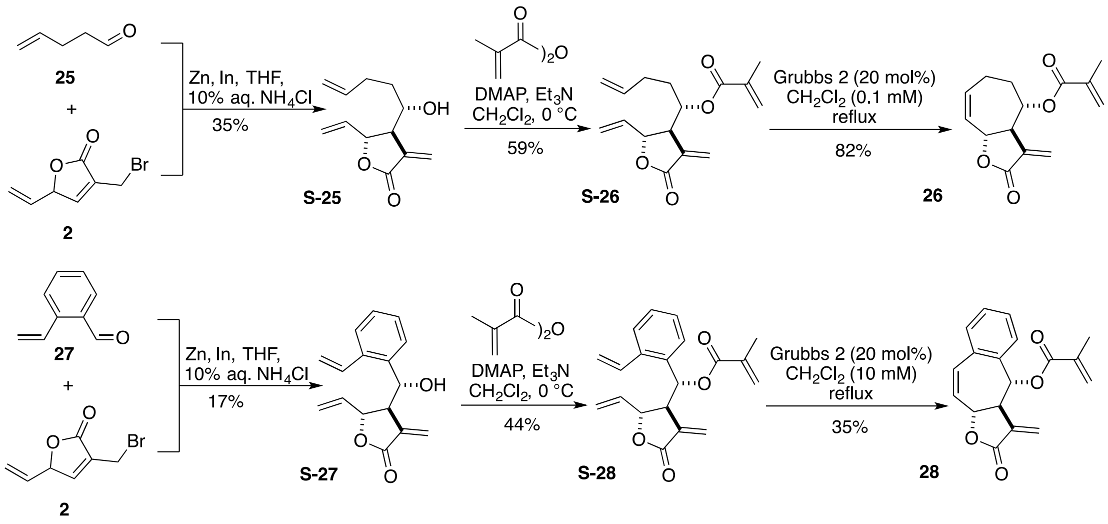

Supplementary figure 27: Synthesis of compounds **26** and **28**

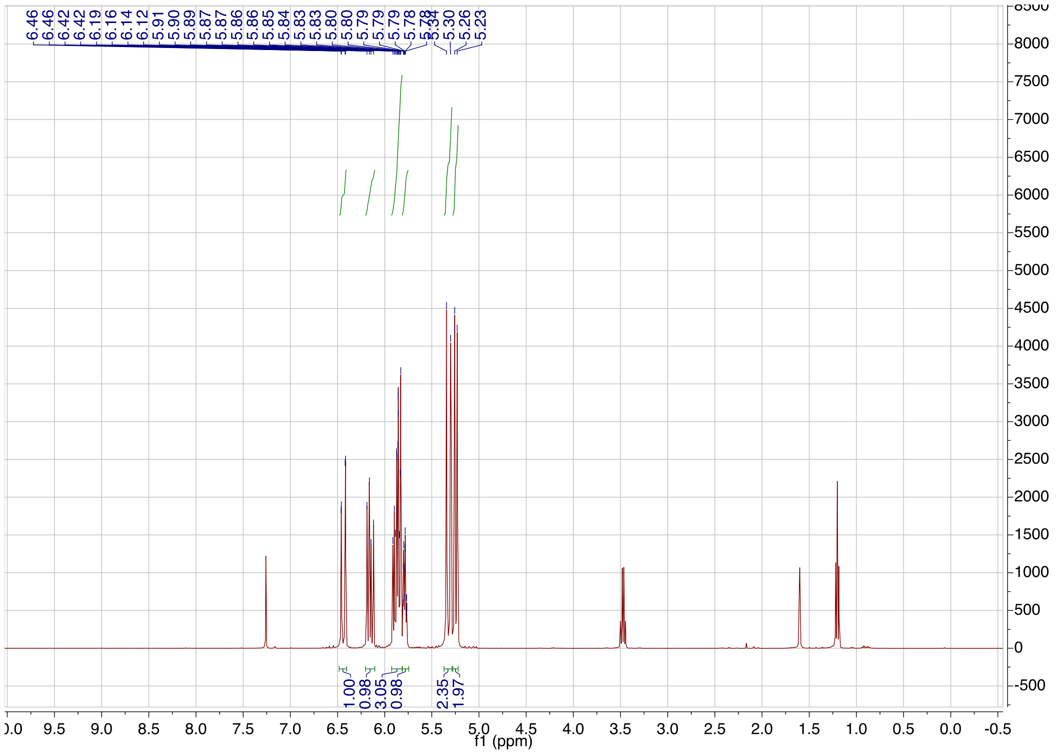

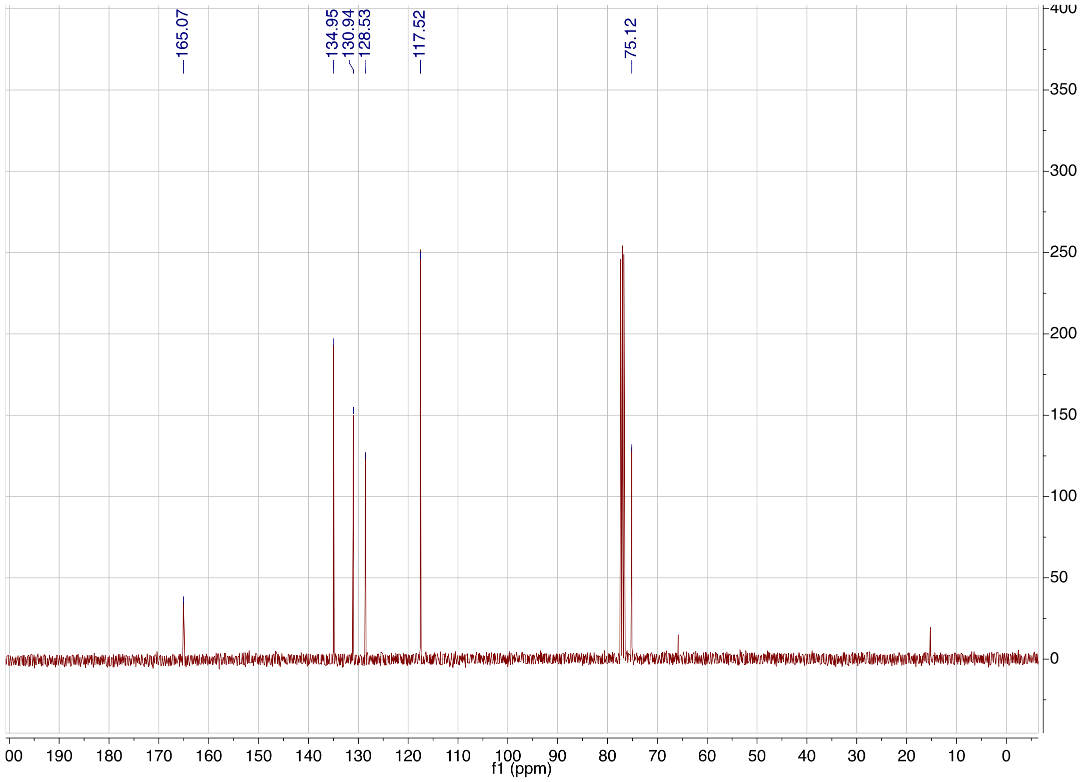

Supplementary figure 28: ^{1}H and ^{13}C NMR spectra of compound **S-1**

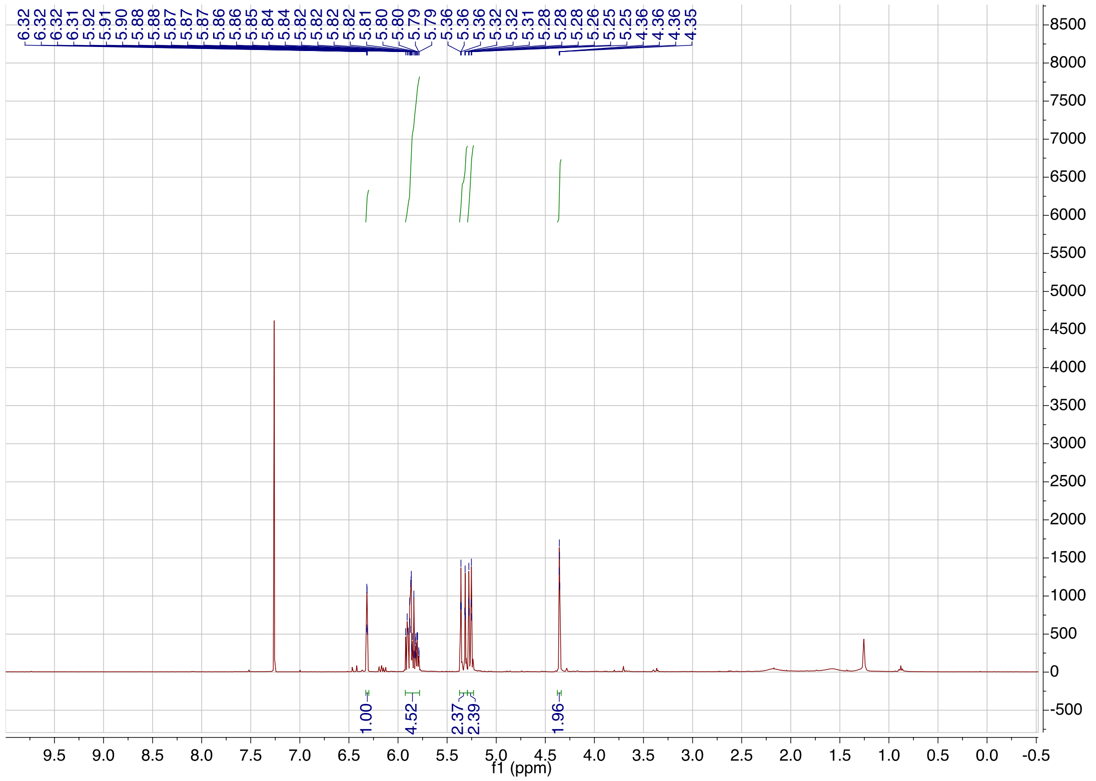

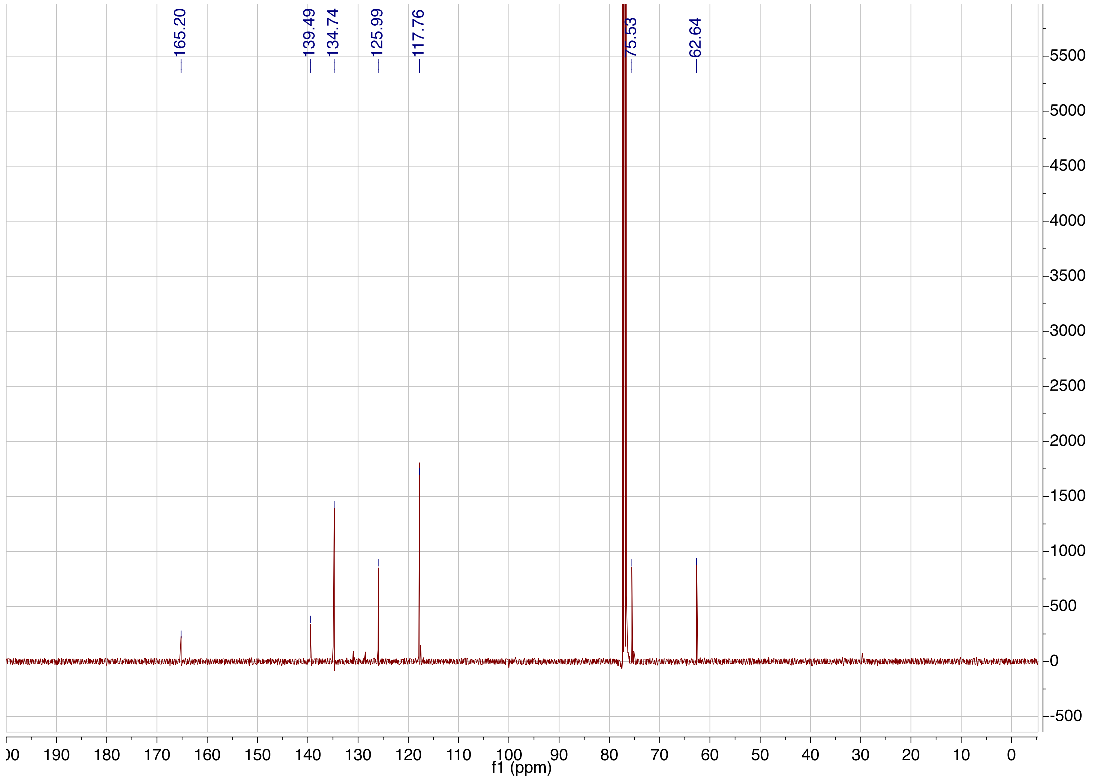

Supplementary figure 29: ^{1}H and ^{13}C NMR spectra of compound **8**

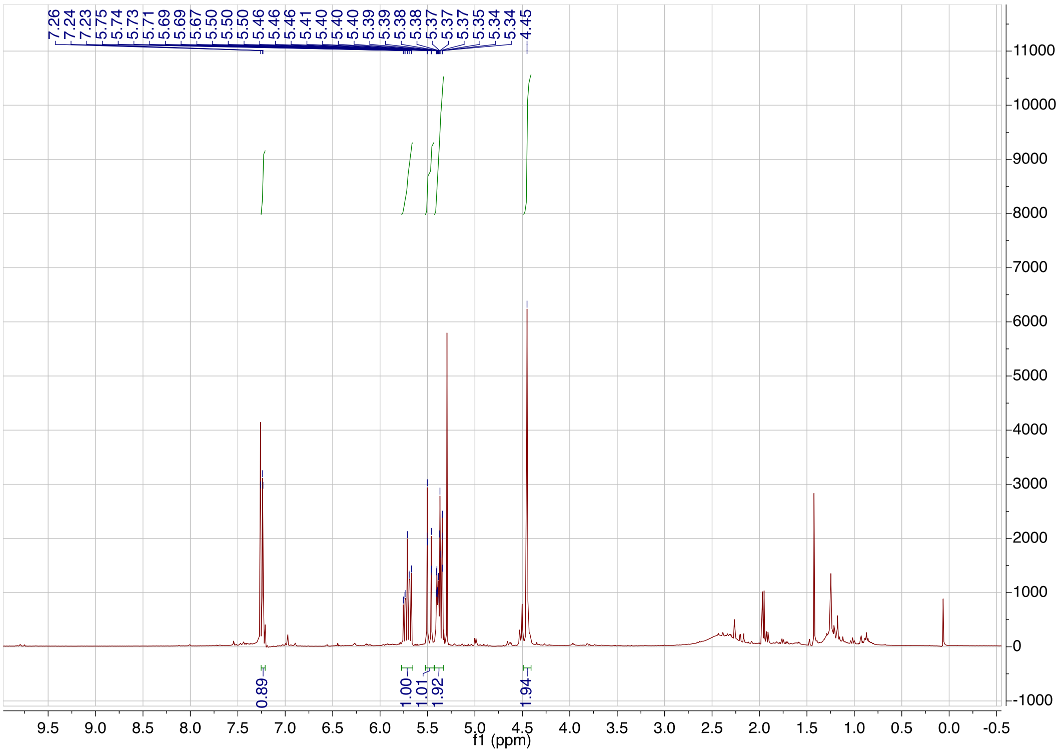

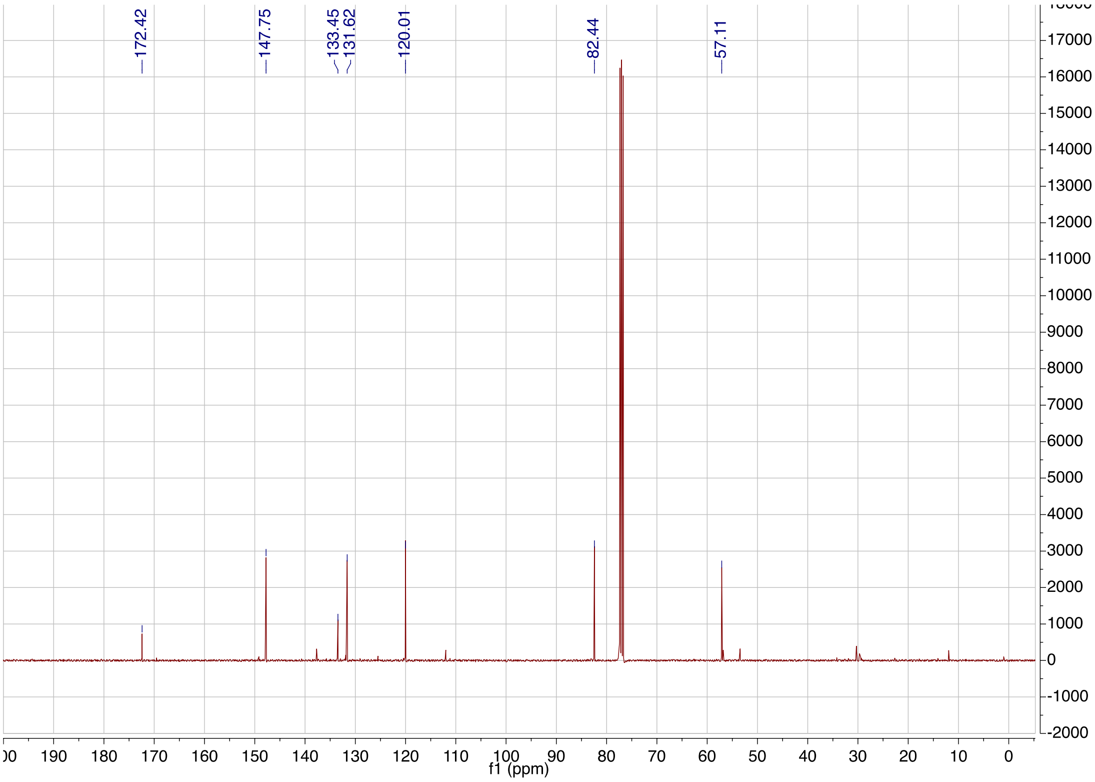

Supplementary figure 30: ^{1}H and ^{13}C NMR spectra of compound **S-2**

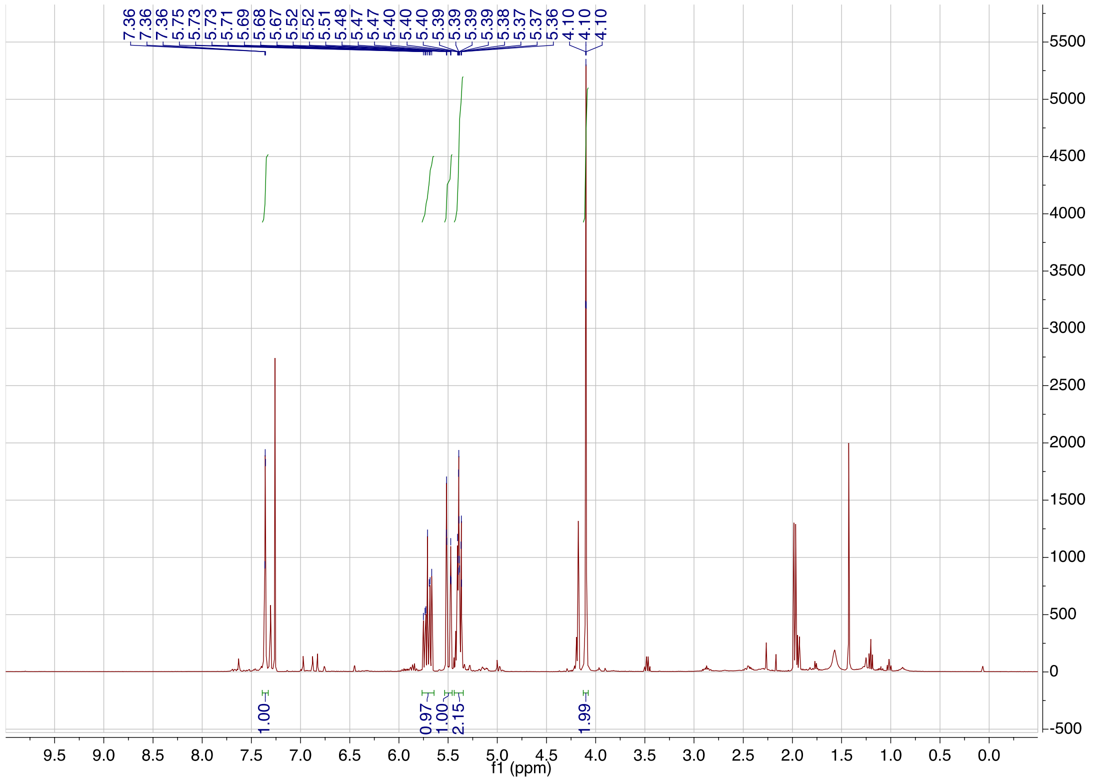

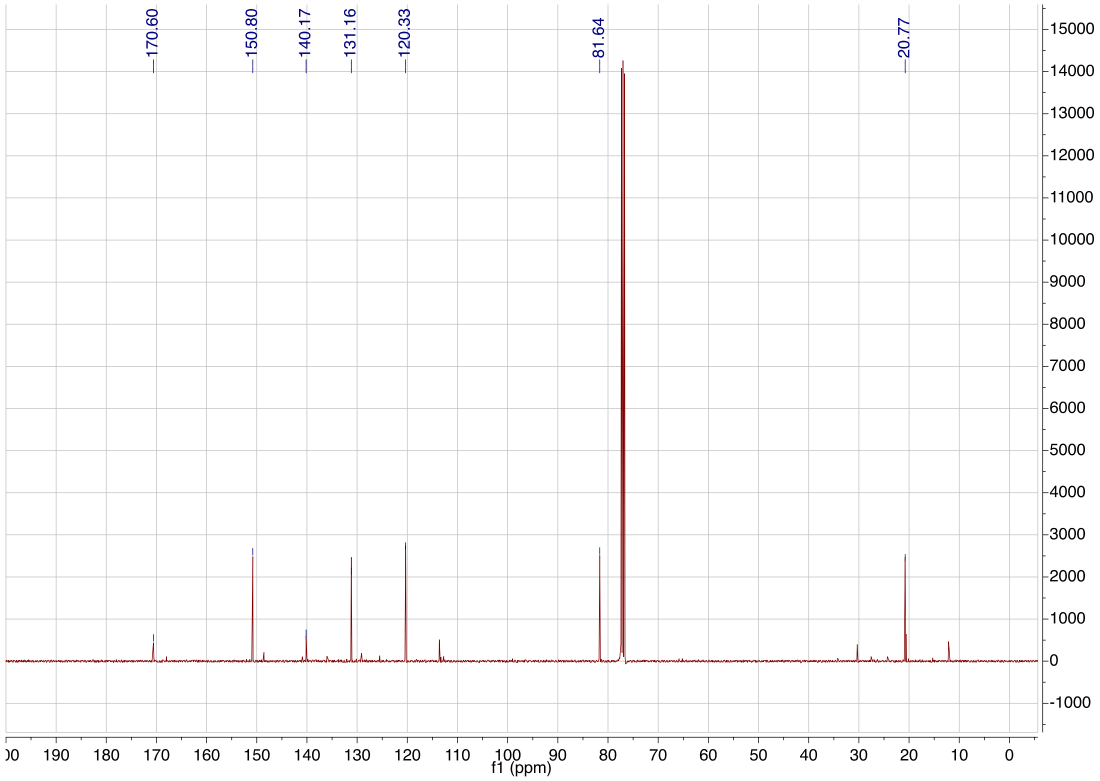

Supplementary figure 31: ^{1}H and ^{13}C NMR spectra of compound **2**

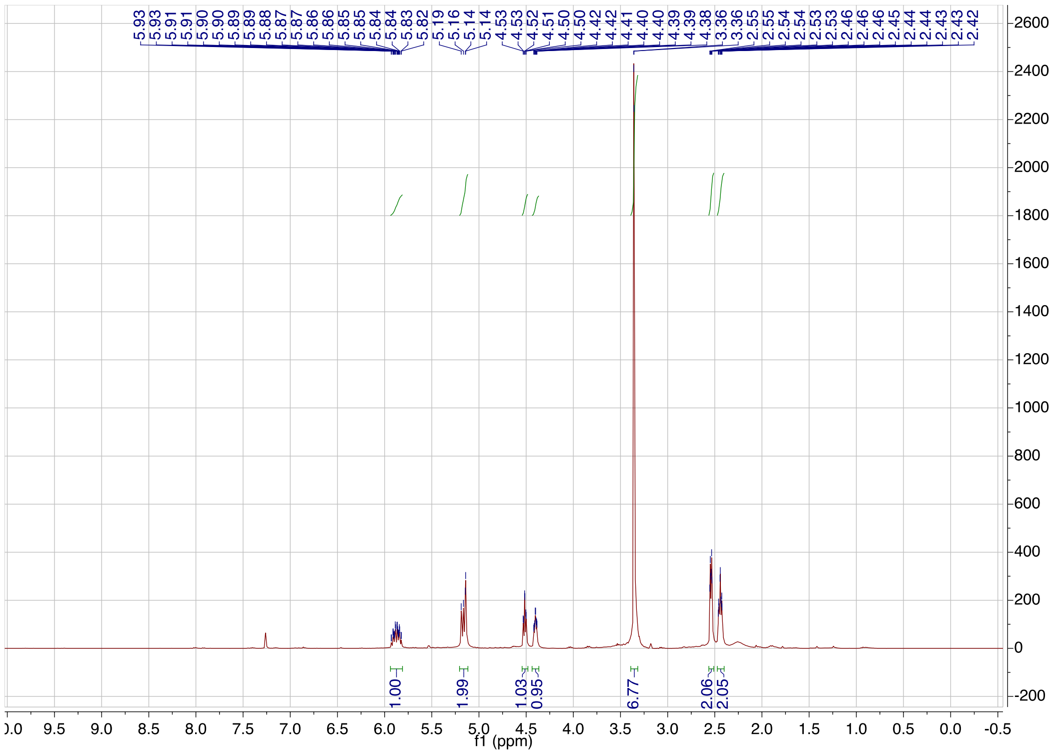

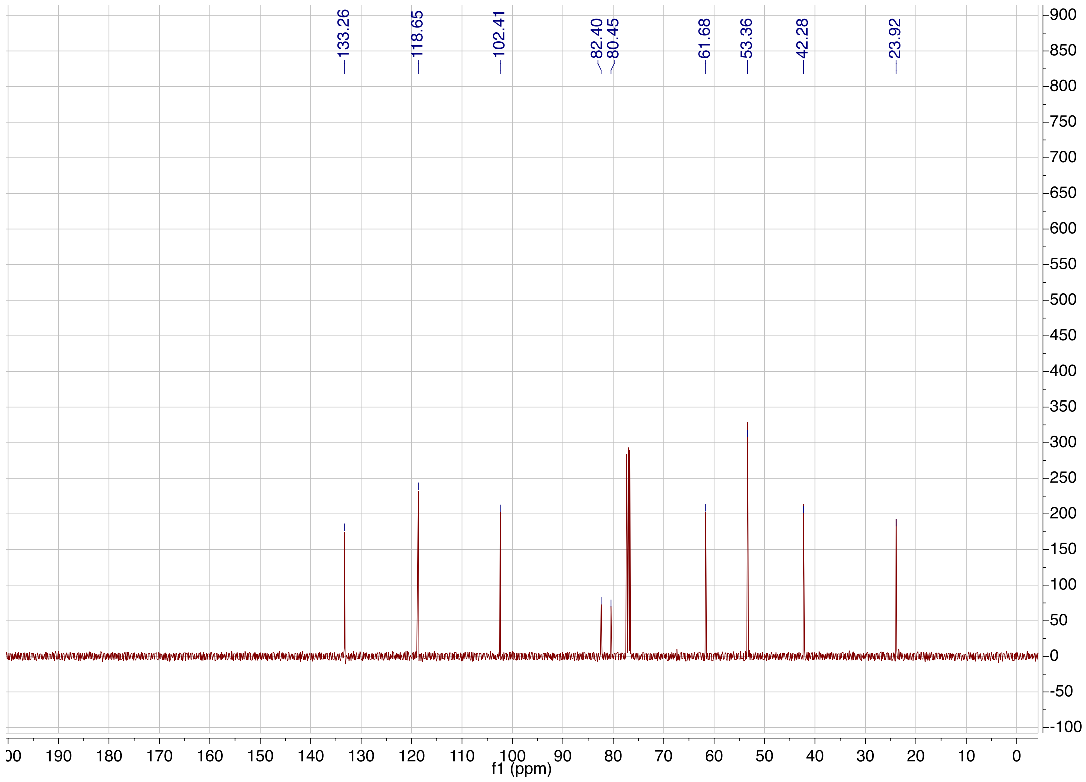

Supplementary figure 32: ^{1}H and ^{13}C NMR spectra of compound **S-4a**

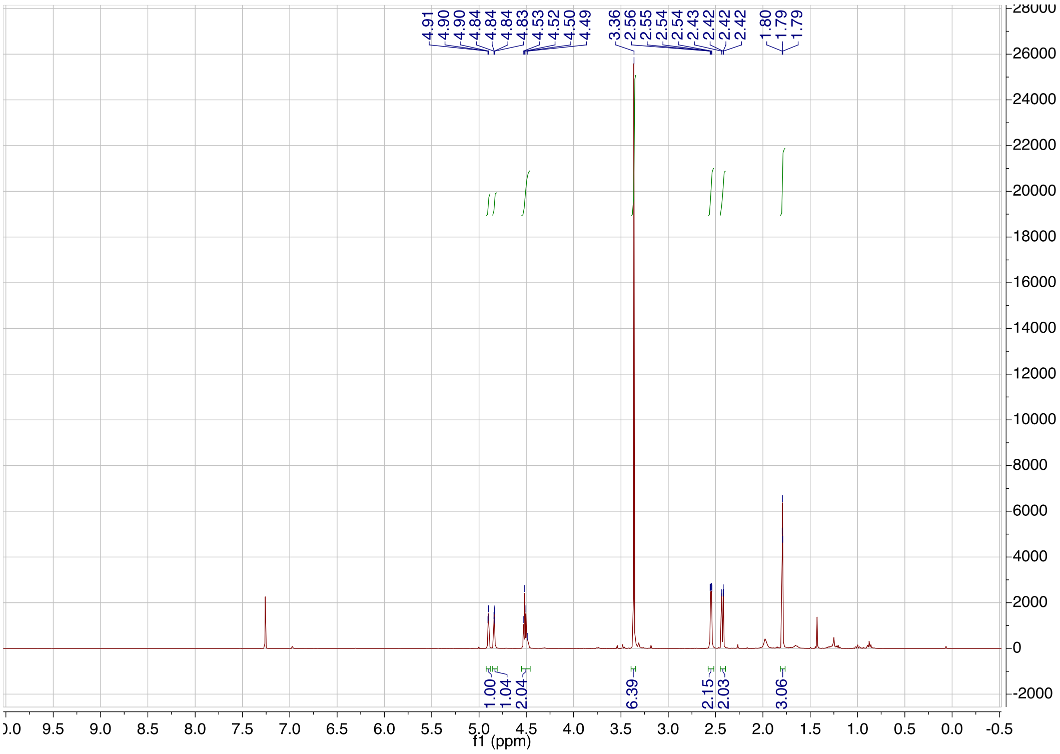

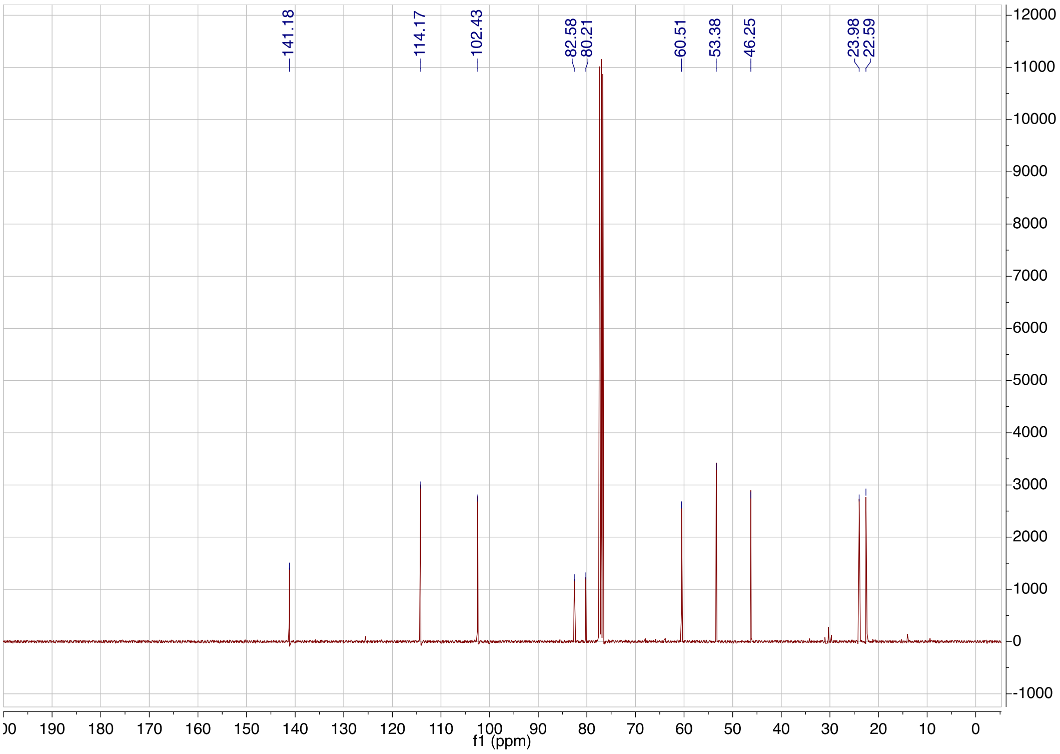

Supplementary figure 33: ^{1}H and ^{13}C NMR spectra of compound **S-4b**

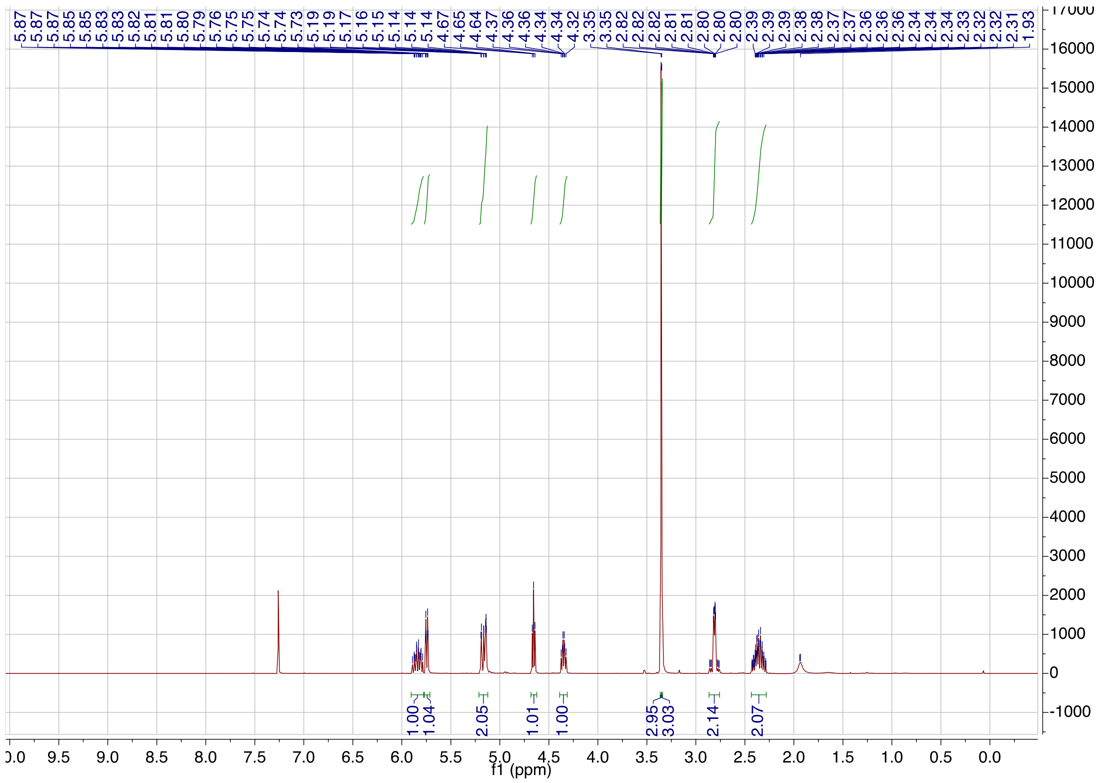

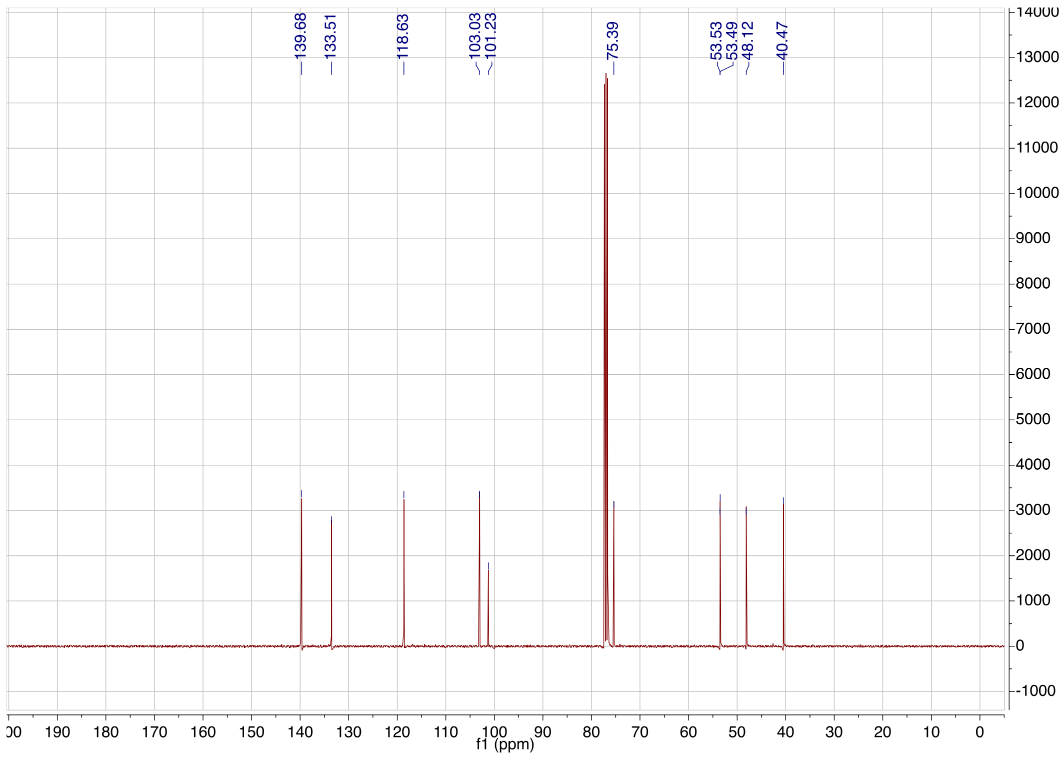

Supplementary figure 34: ^{1}H and ^{13}C NMR spectra of compound **5a**

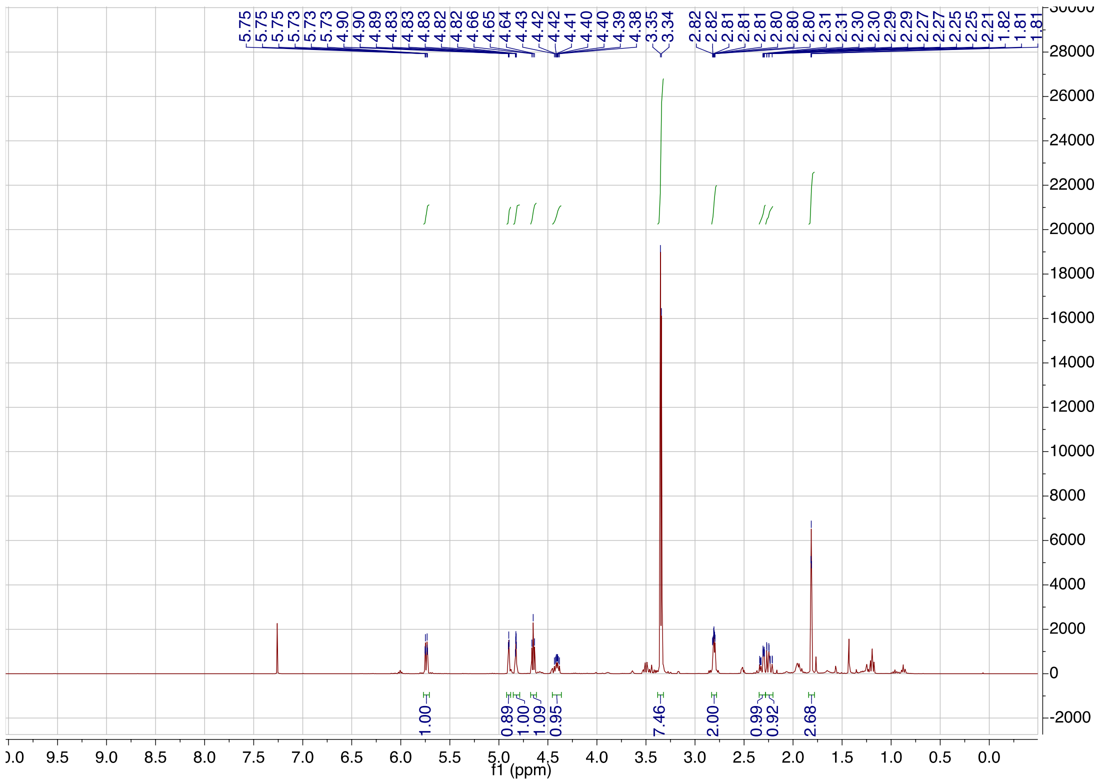

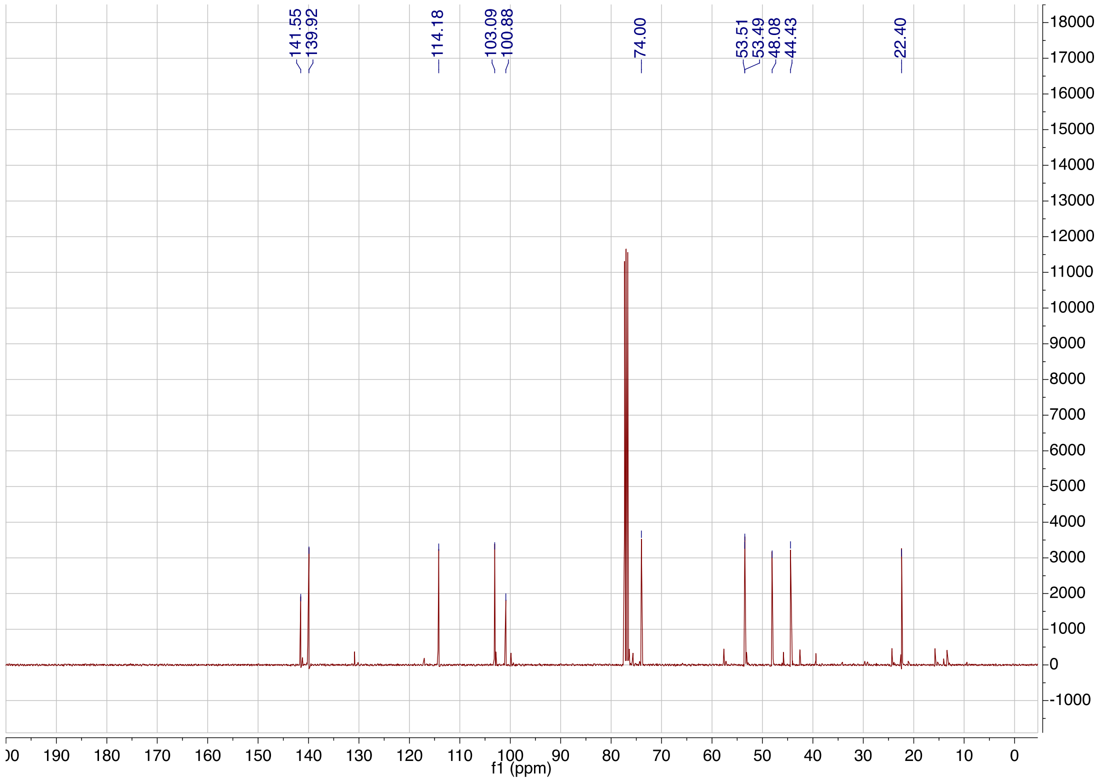

Supplementary figure 35: ^{1}H and ^{13}C NMR spectra of compound **5b**

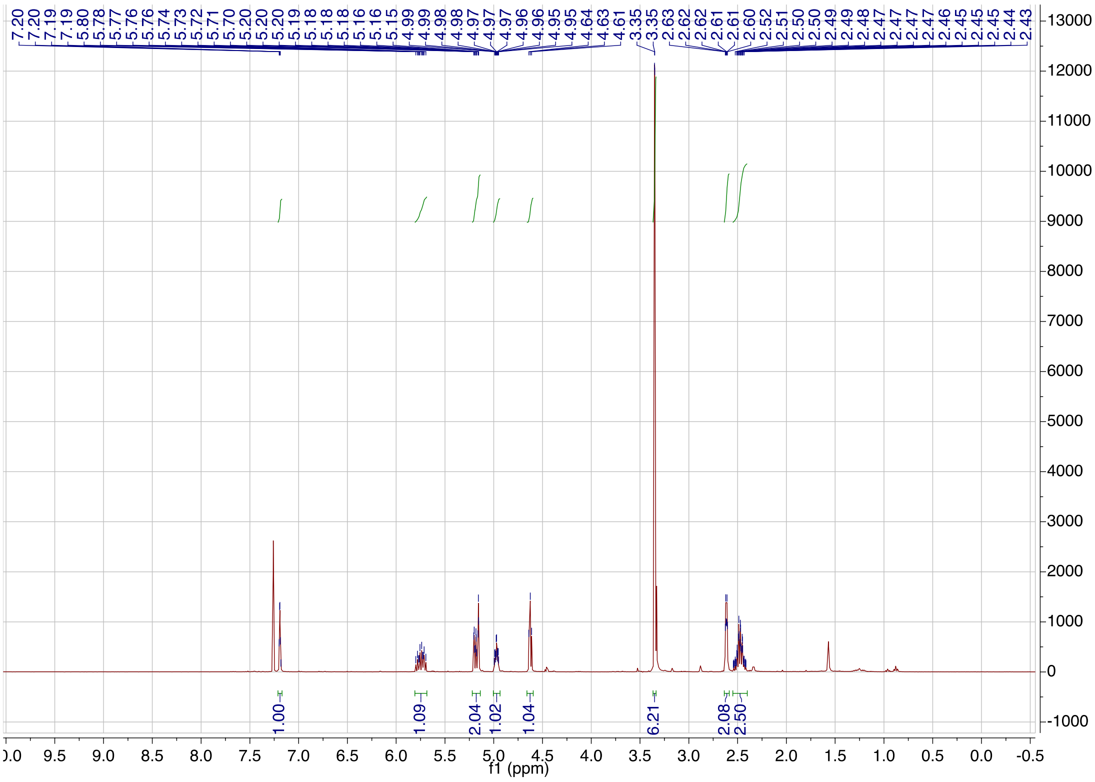

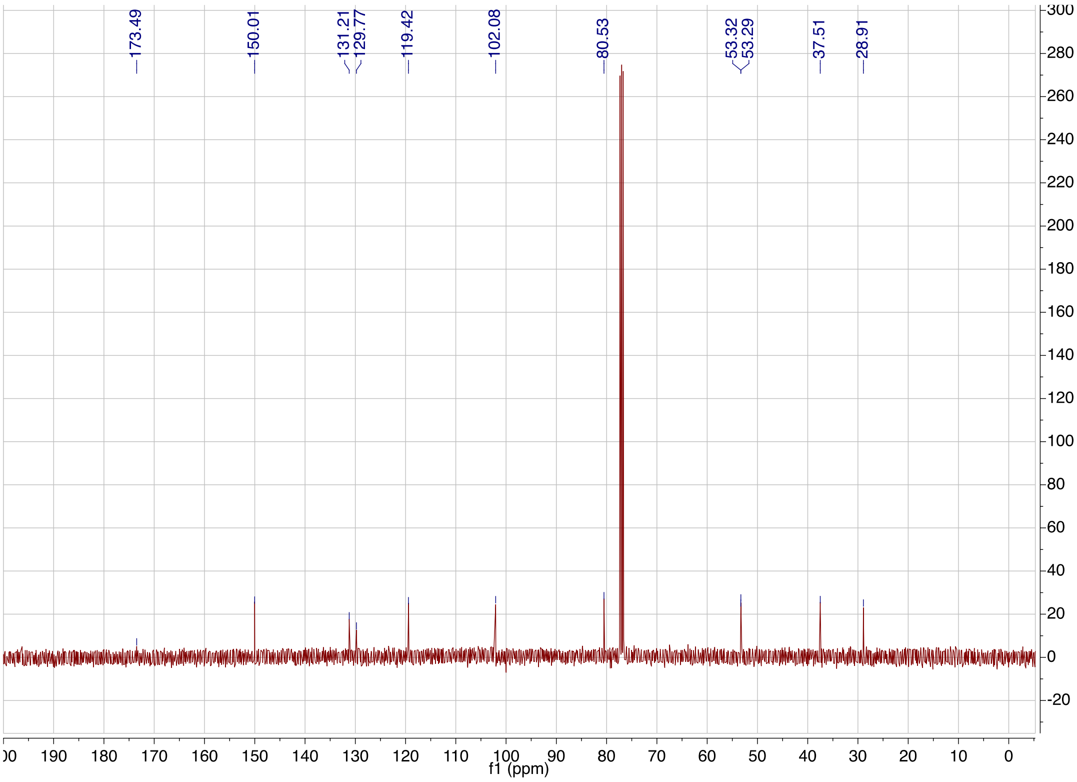

Supplementary figure 36: ^{1}H and ^{13}C NMR spectra of compound **6a**

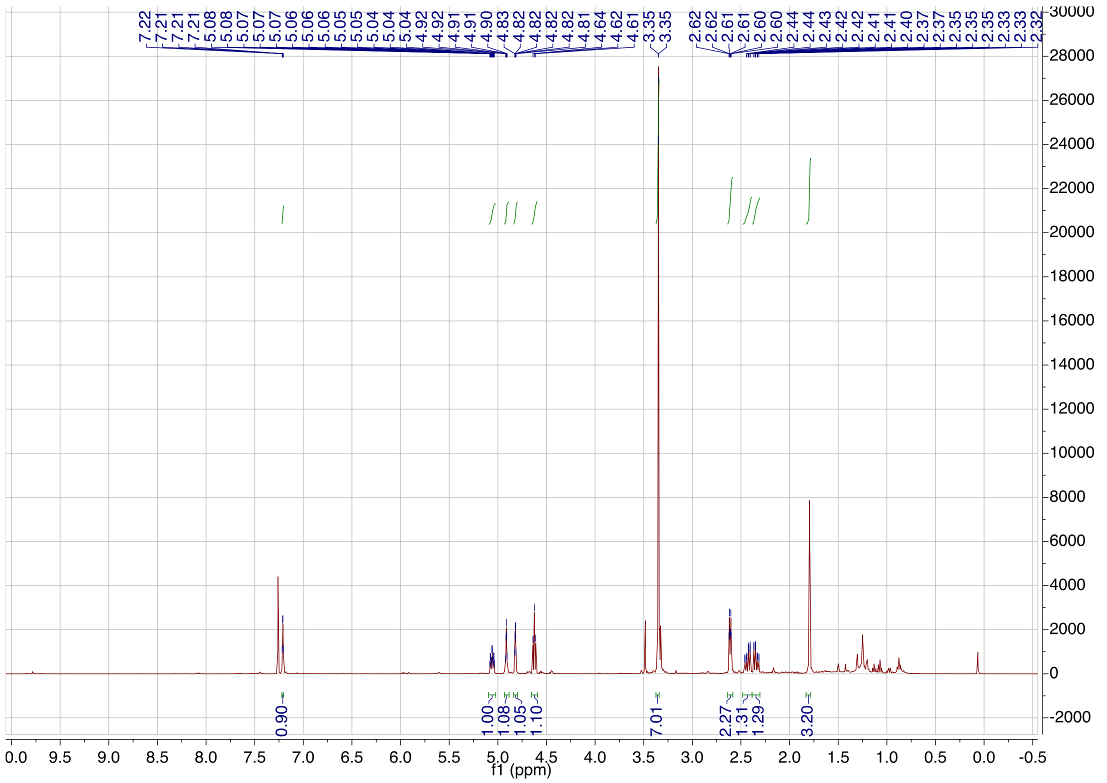

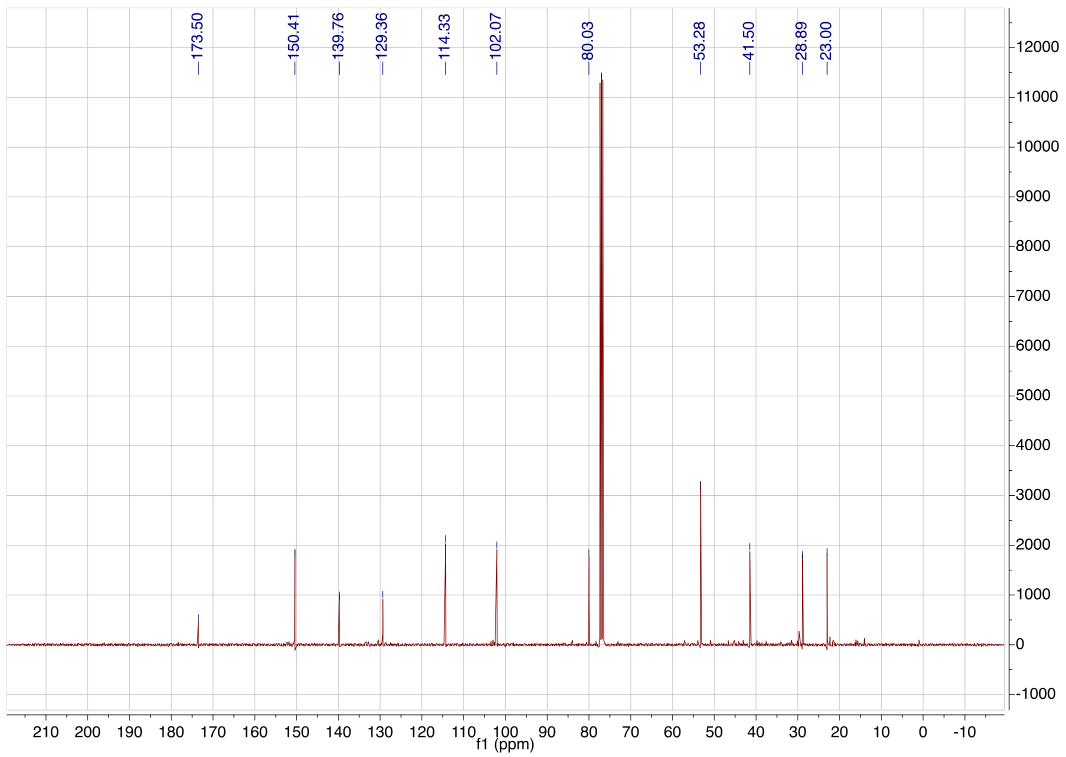

Supplementary figure 37: ^{1}H and ^{13}C NMR spectra of compound **6b**

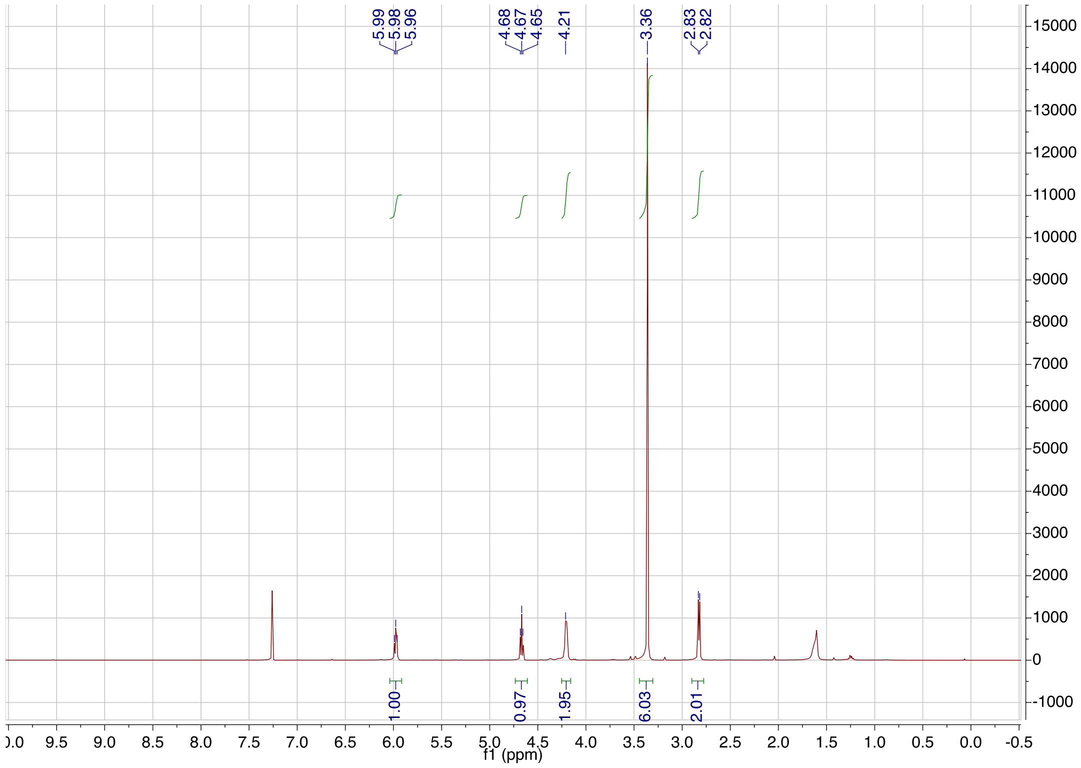

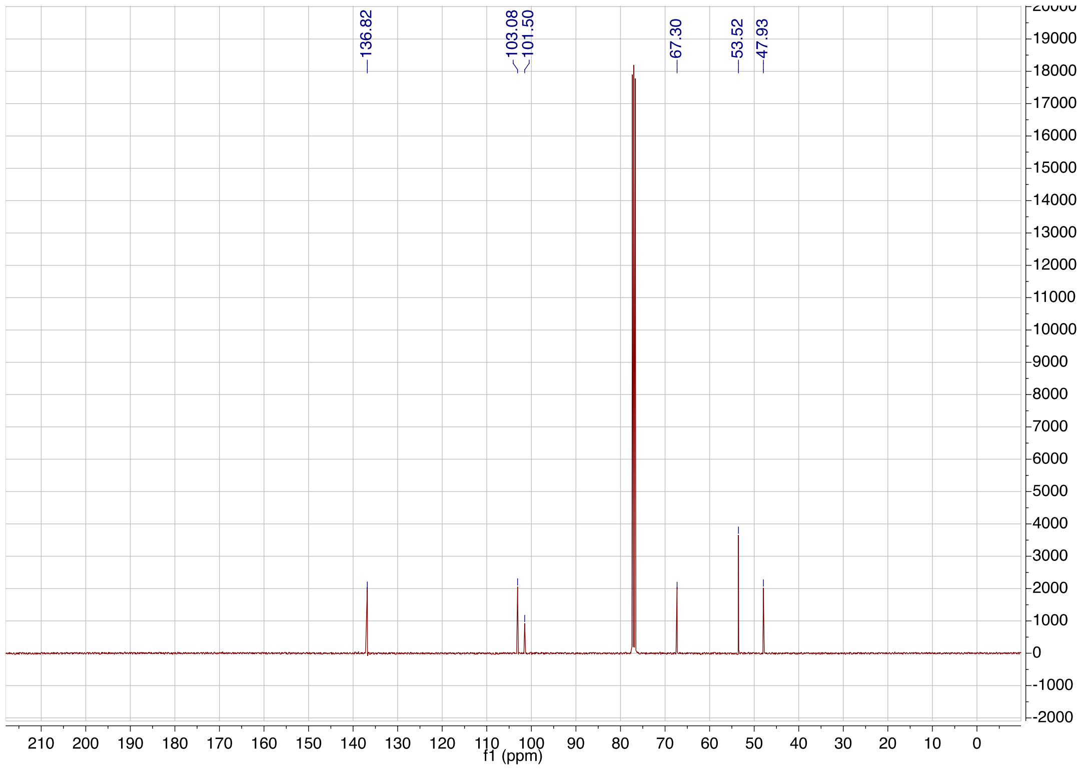

Supplementary figure 38: ^{1}H and ^{13}C NMR spectra of compound **S-7**

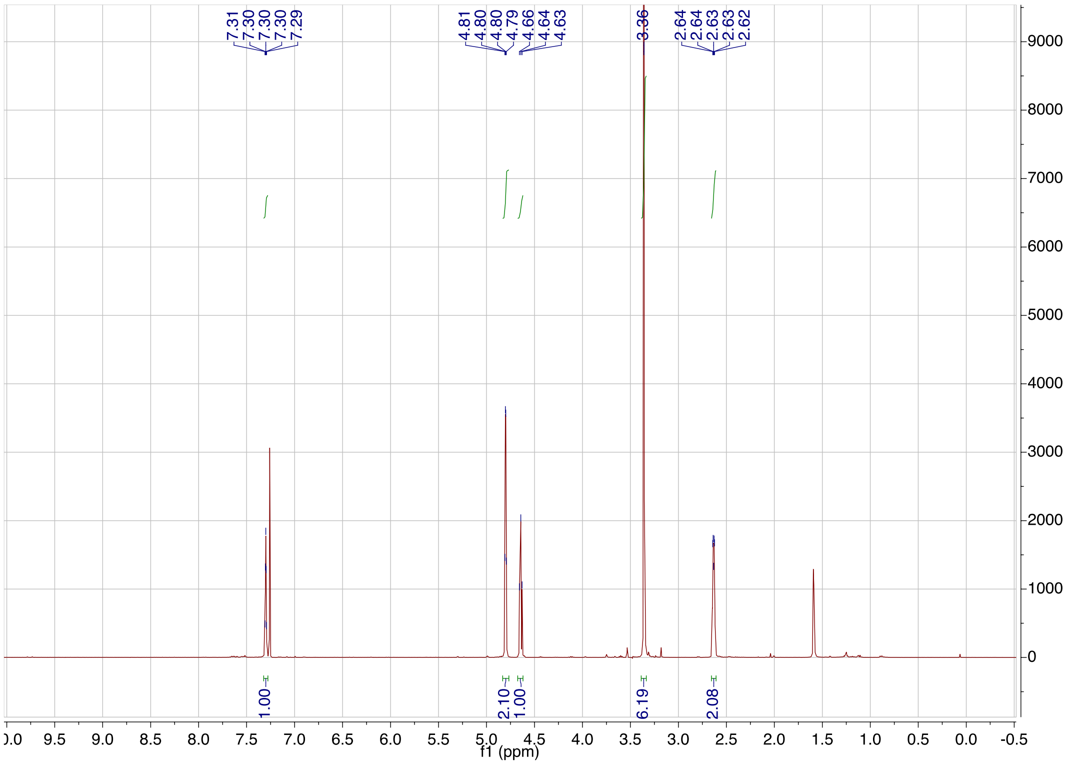

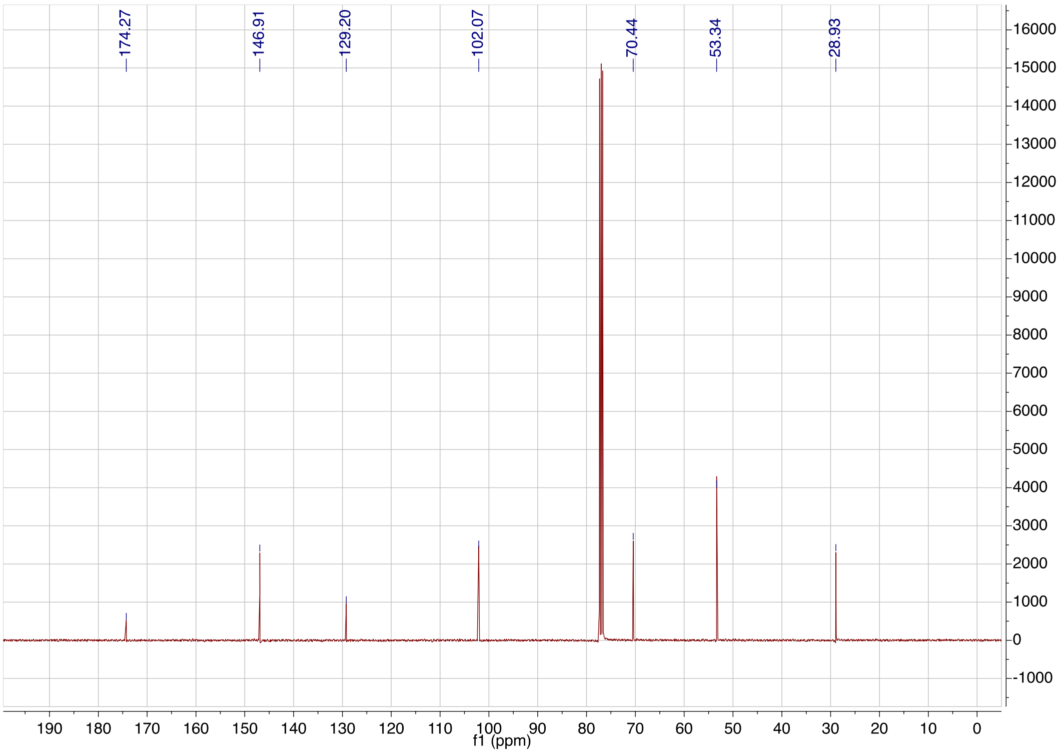

Supplementary figure 39: ^{1}H and ^{13}C NMR spectra of compound **13**

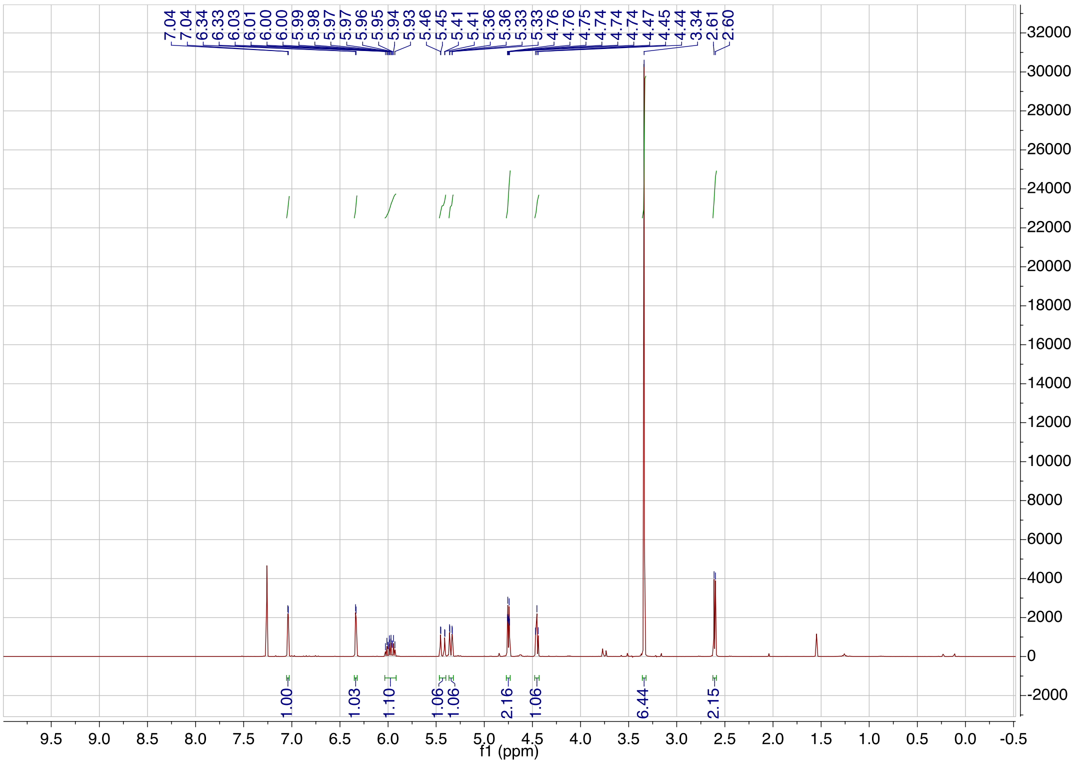

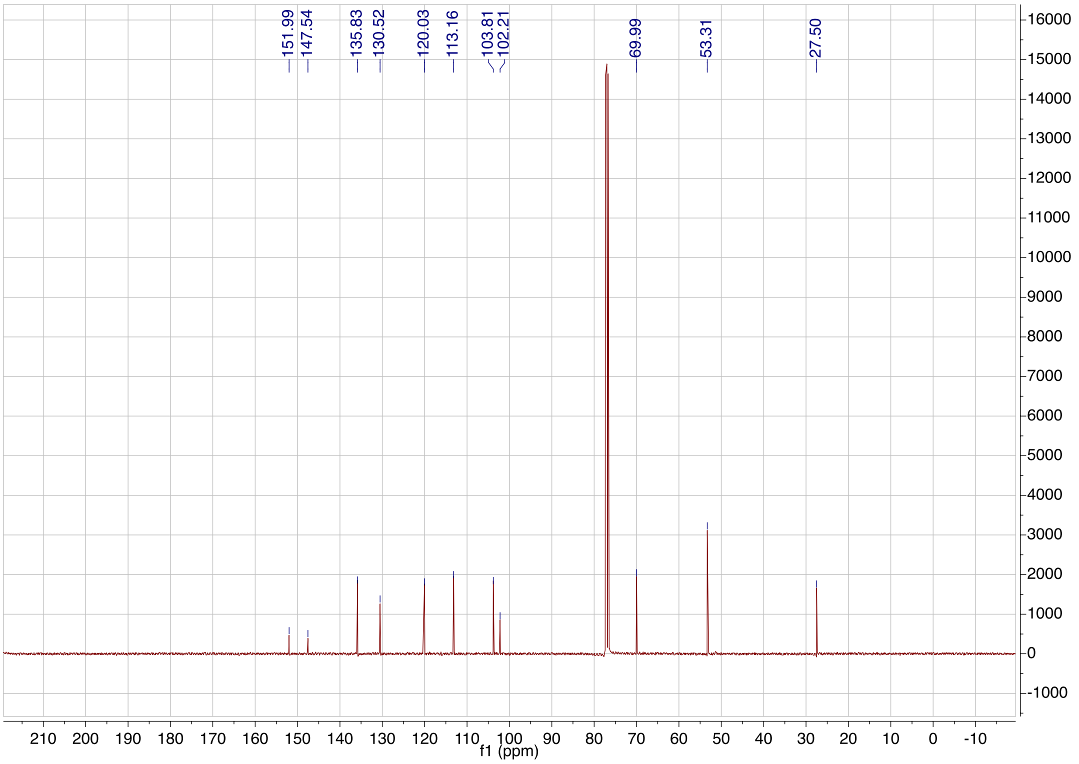

Supplementary figure 40: ^{1}H and ^{13}C NMR spectra of compound **14**

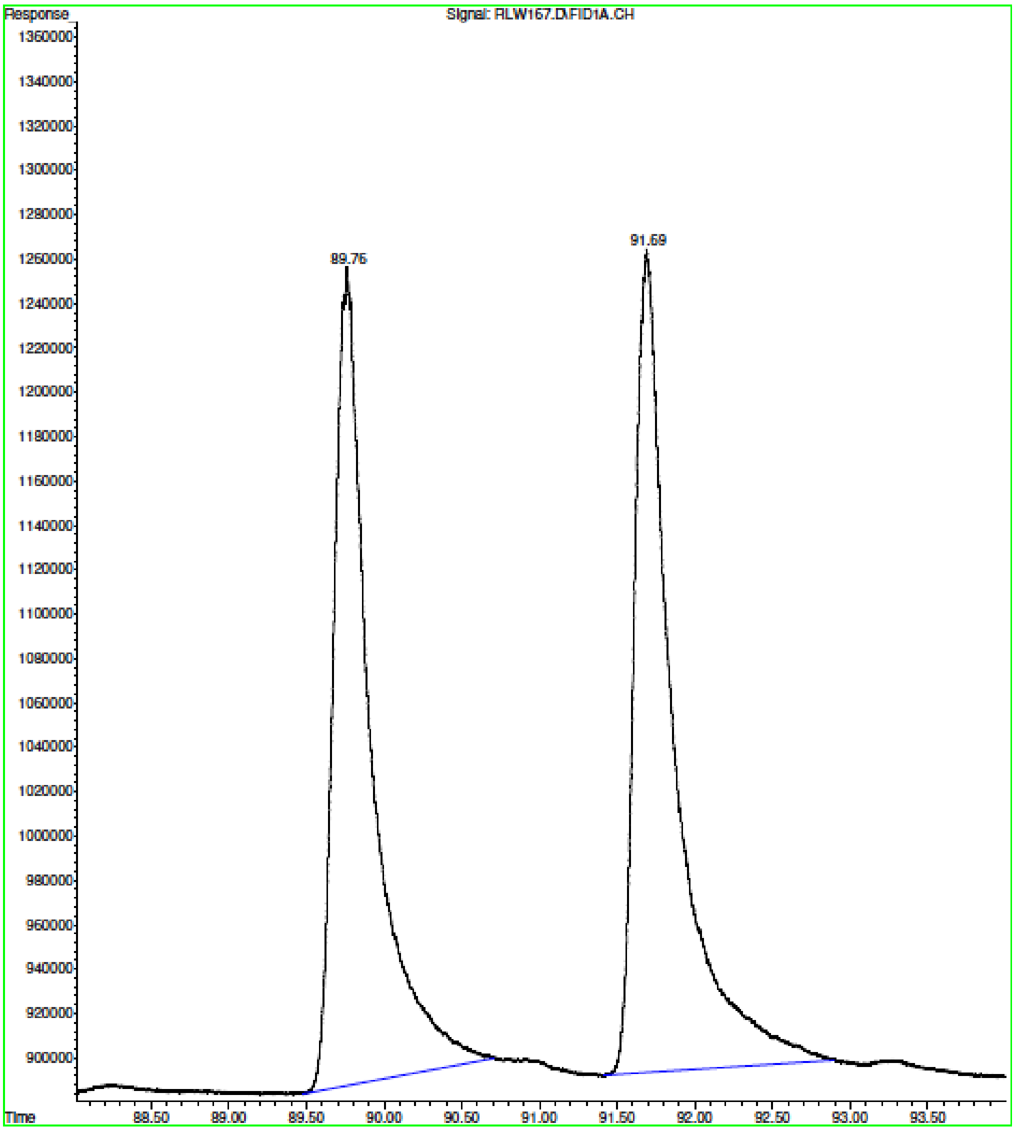

Signal #1 : FID1A.CH

peak #	R.T. min	Start min	End min	PK TY	peak height	peak area	peak % max.	% of total
1	89.759	89.476	90.700	M	369374	61601865	93.65%	48.360%
2	91.687	91.419	92.895	M	370962	65779710	100.00%	51.640%

Sum of corrected areas: 127381576

Supplementary figure 41: Chiral GC trace for compound ***rac*-6**

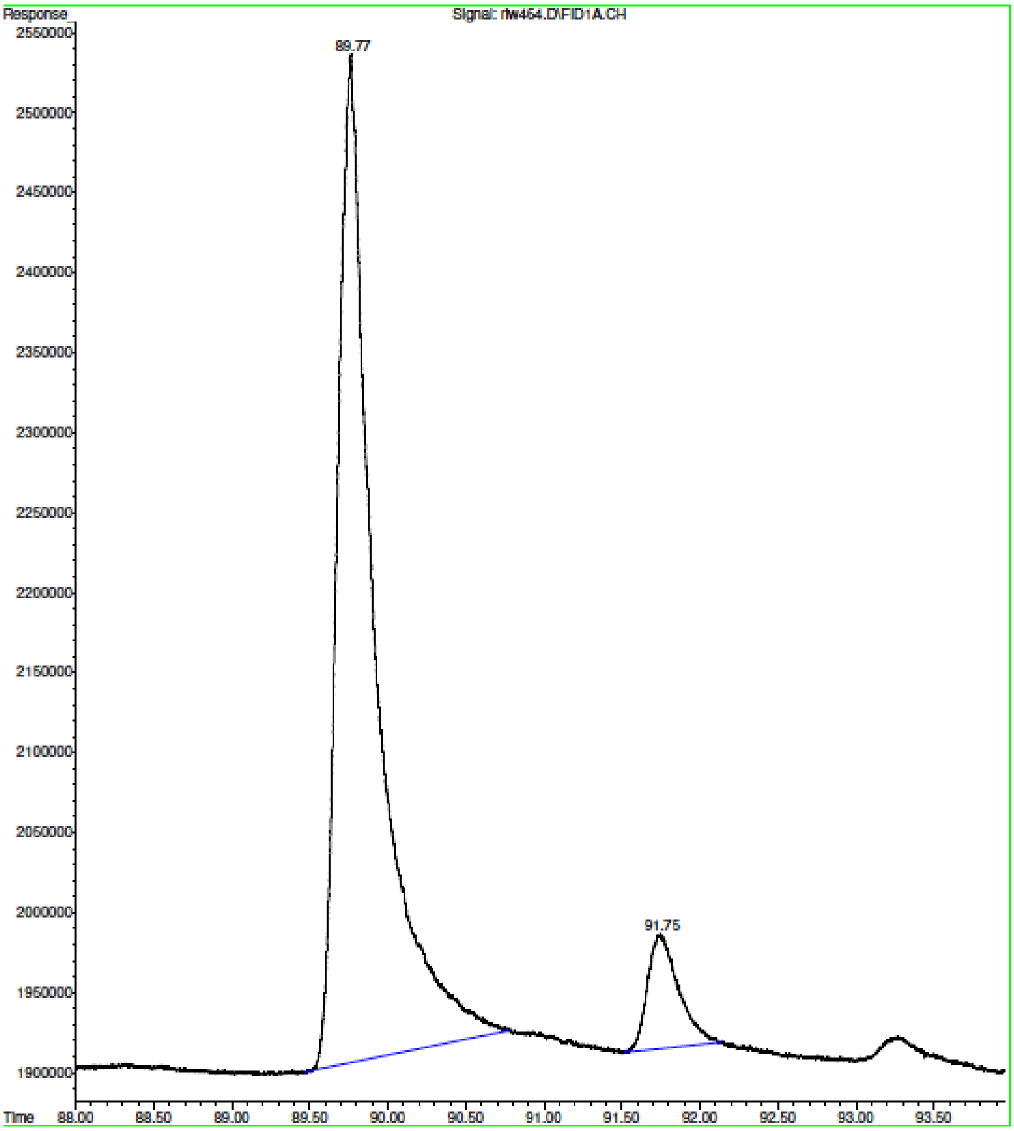

Signal : FID1A.CH

peak #	R.T. min	Start min	End min	PK TY	peak height	peak area	peak % max.	% of total
1	89.766	89.466	90.768	M	630344	106707584	100.00%	91.518%
2	91.746	91.525	92.127	M	71983	9890027	9.27%	8.482%

Sum of corrected areas: 116597611

Supplementary figure 42: Chiral GC trace for compound **(*R*)-6**

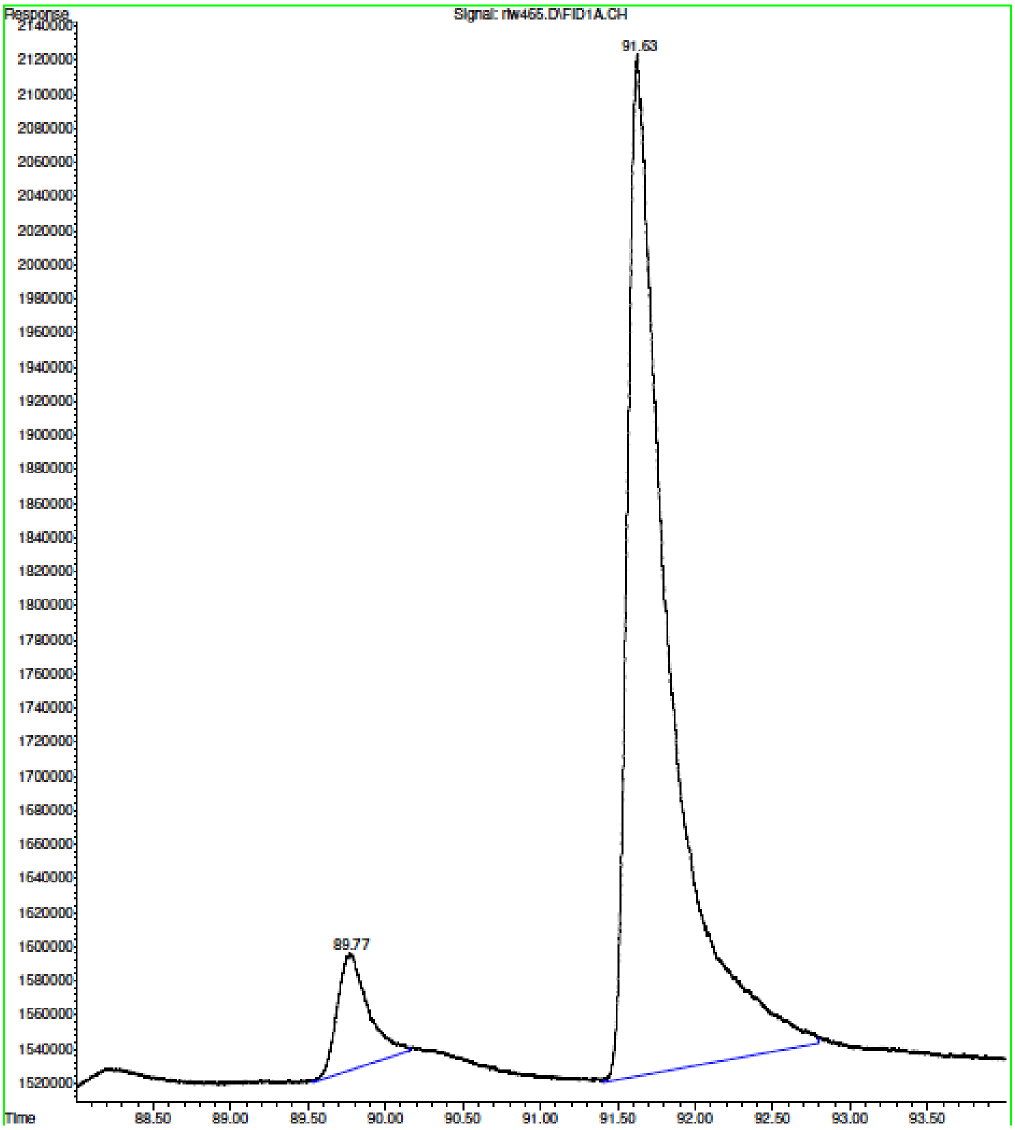

Signal : FID1A.CH

peak #	R.T. min	Start min	End min	PK TY	peak height	peak area	peak % max.	% of total
1	89.769	89.531	90.151	M	68546	9543139	8.55%	7.881%
2	91.629	91.403	92.799	M	599778	111552289	100.00%	92.119%

Sum of corrected areas: 121095428

Supplementary figure 43: Chiral GC trace for compound **(*S*)-6**

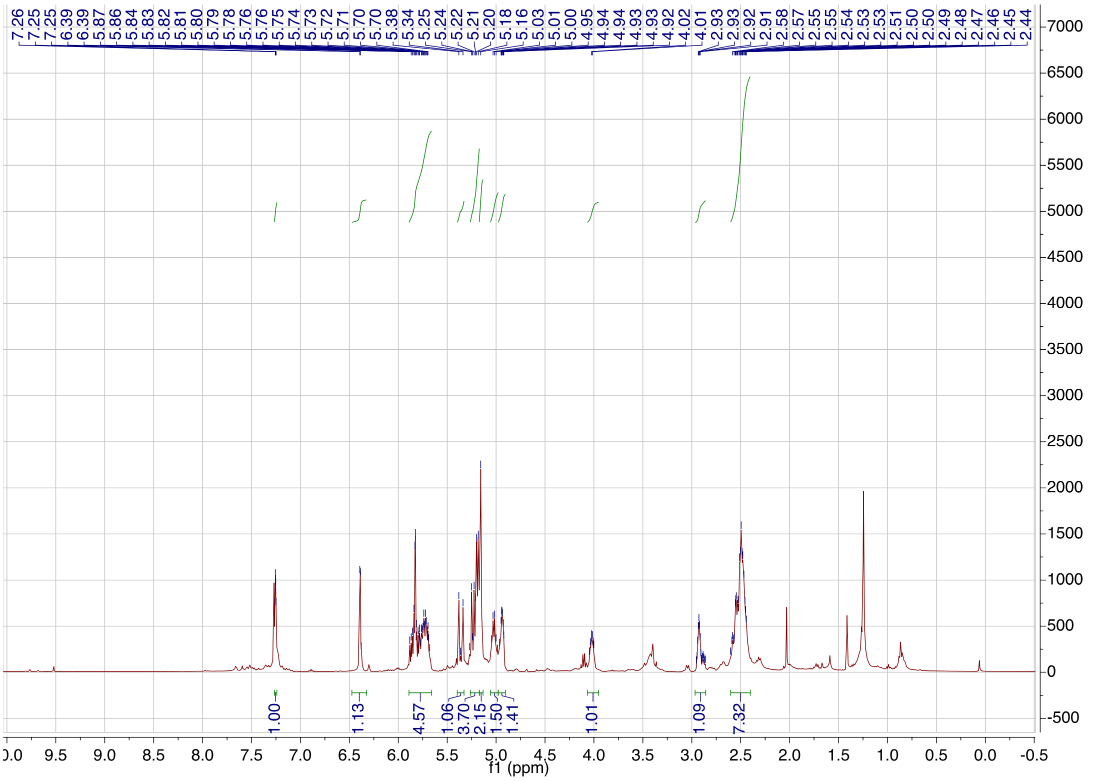

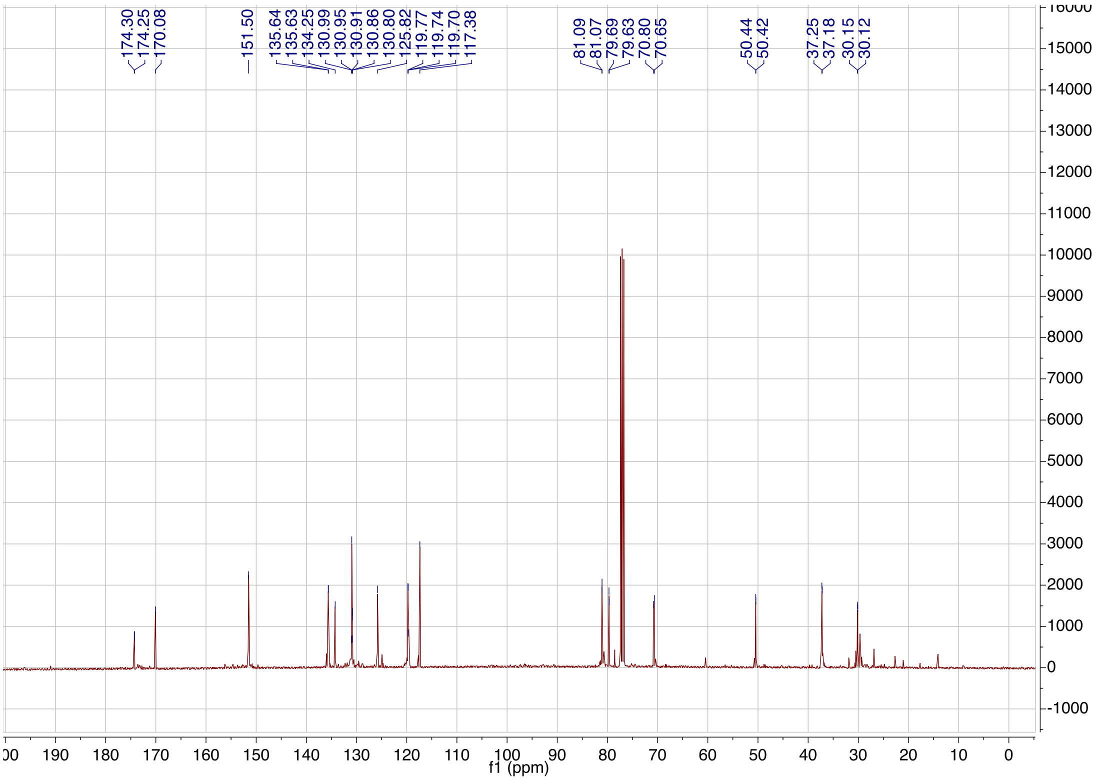

Supplementary figure 44: ^{1}H and ^{13}C NMR spectra of compound ***rac*-9**

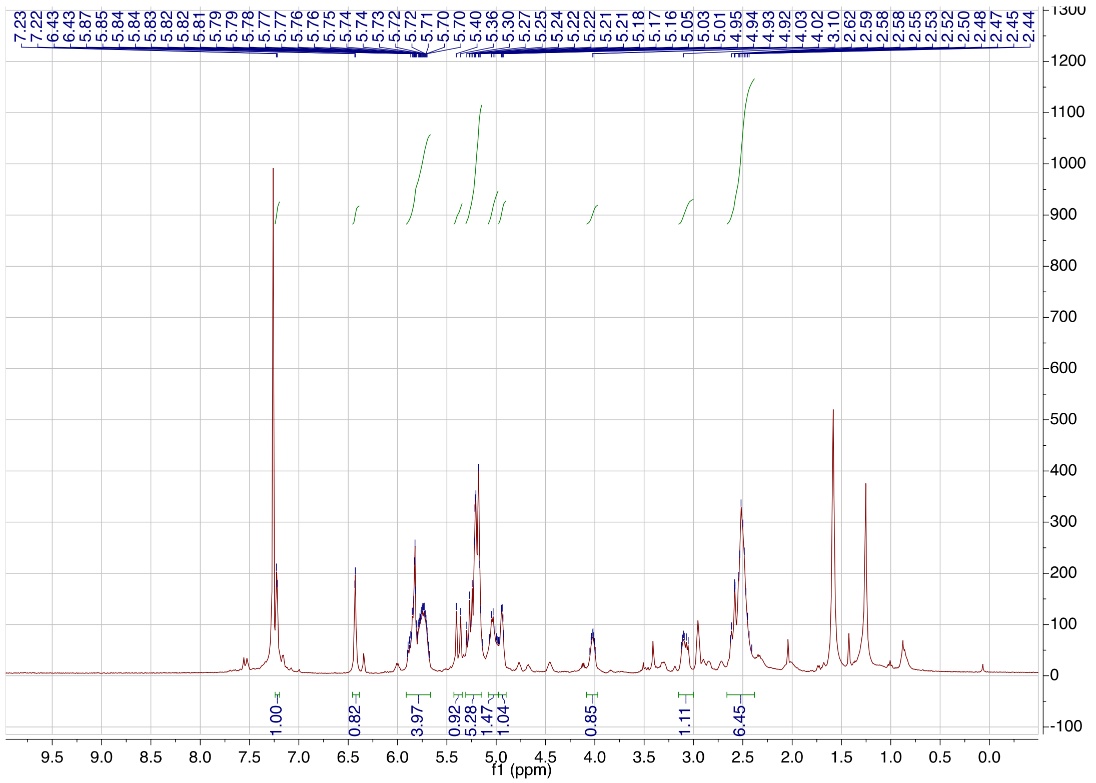

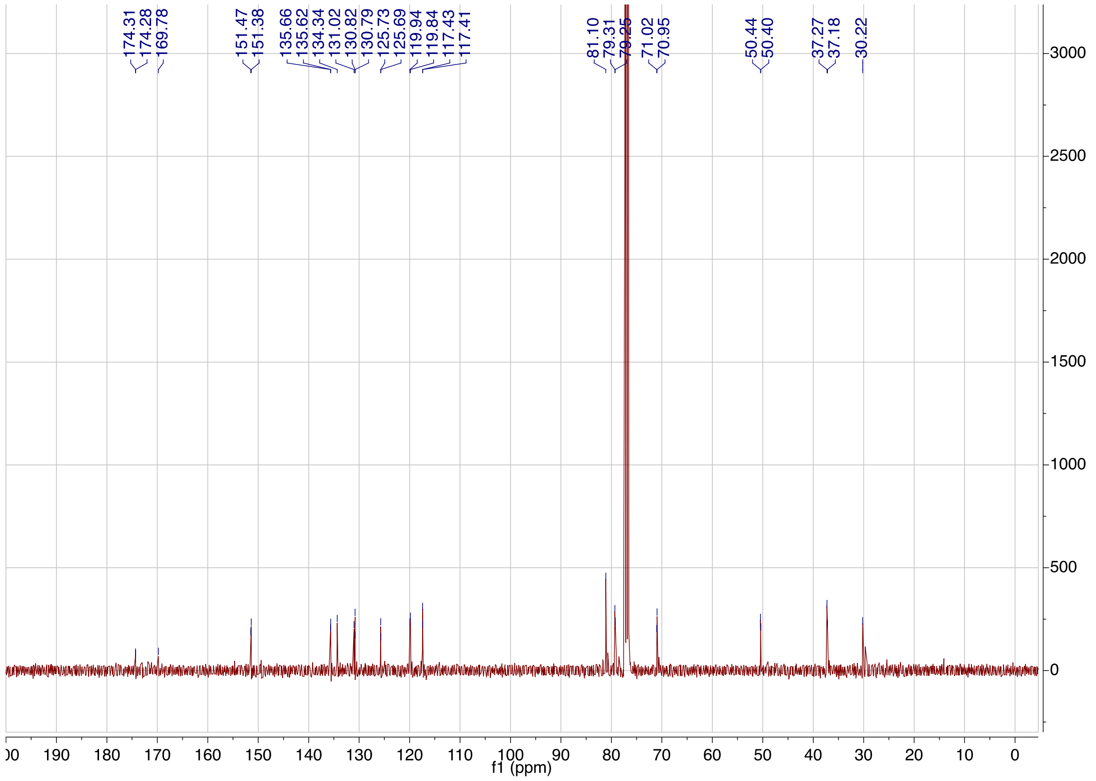

Supplementary figure 45: ^{1}H and ^{13}C NMR spectra of compound ***(R)*-9**

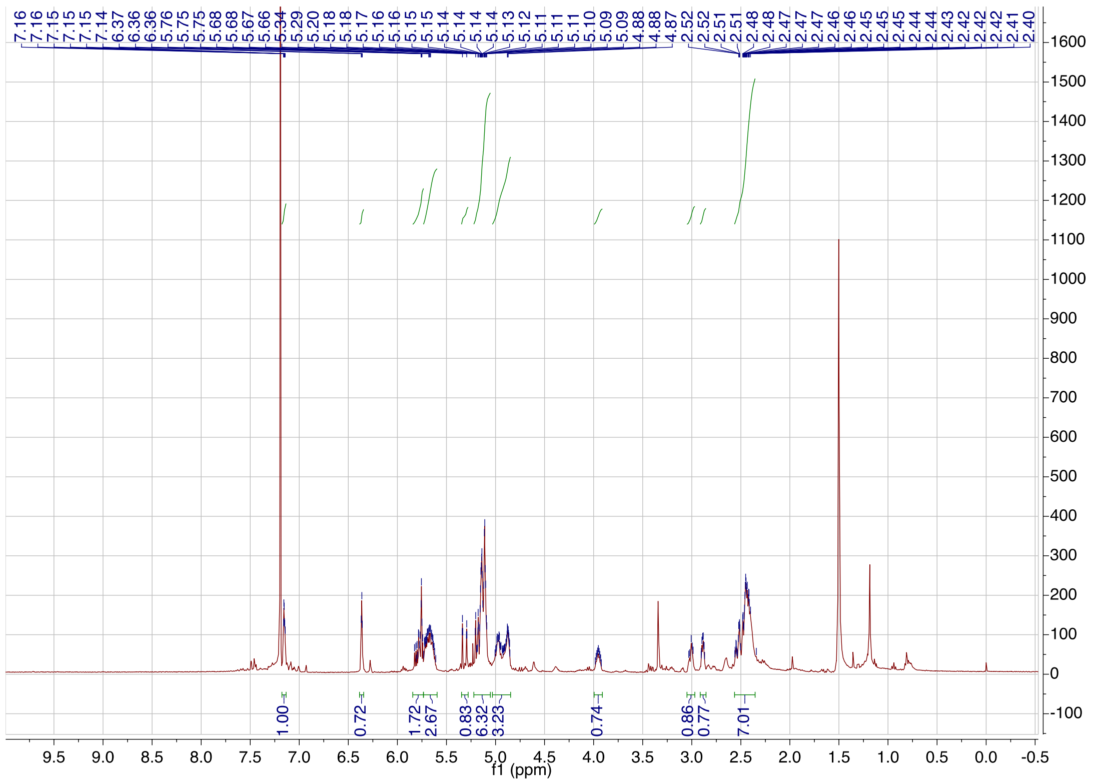

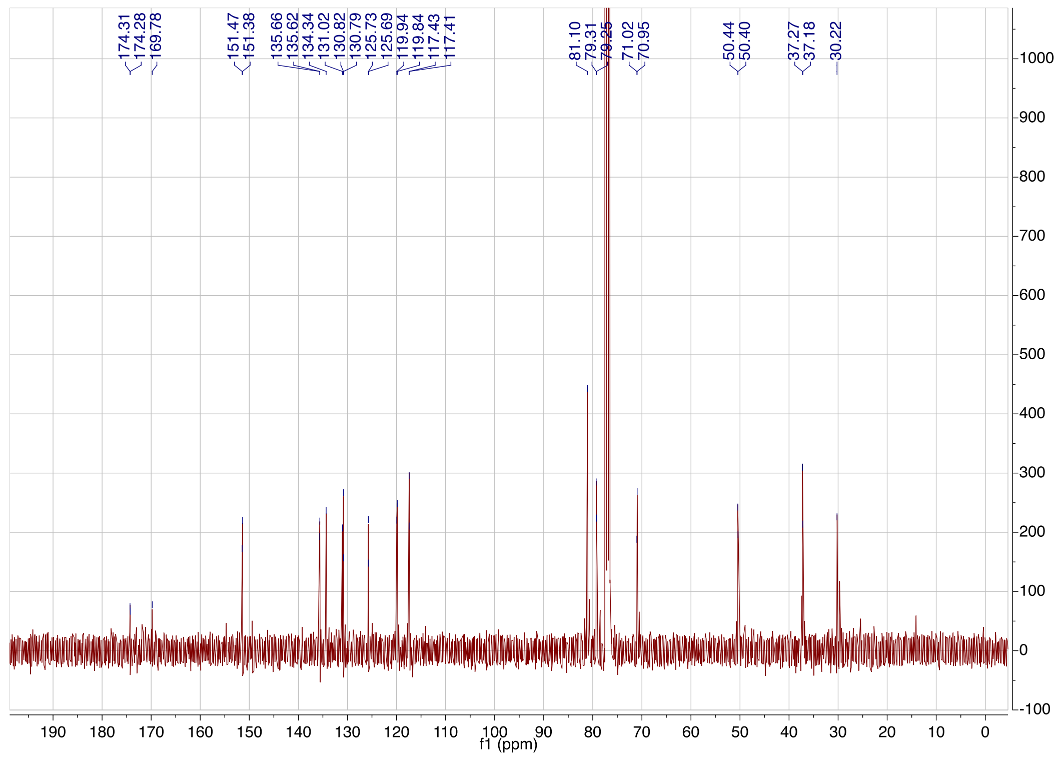

Supplementary figure 46: ^{1}H and ^{13}C NMR spectra of compound ***(S)*-9**

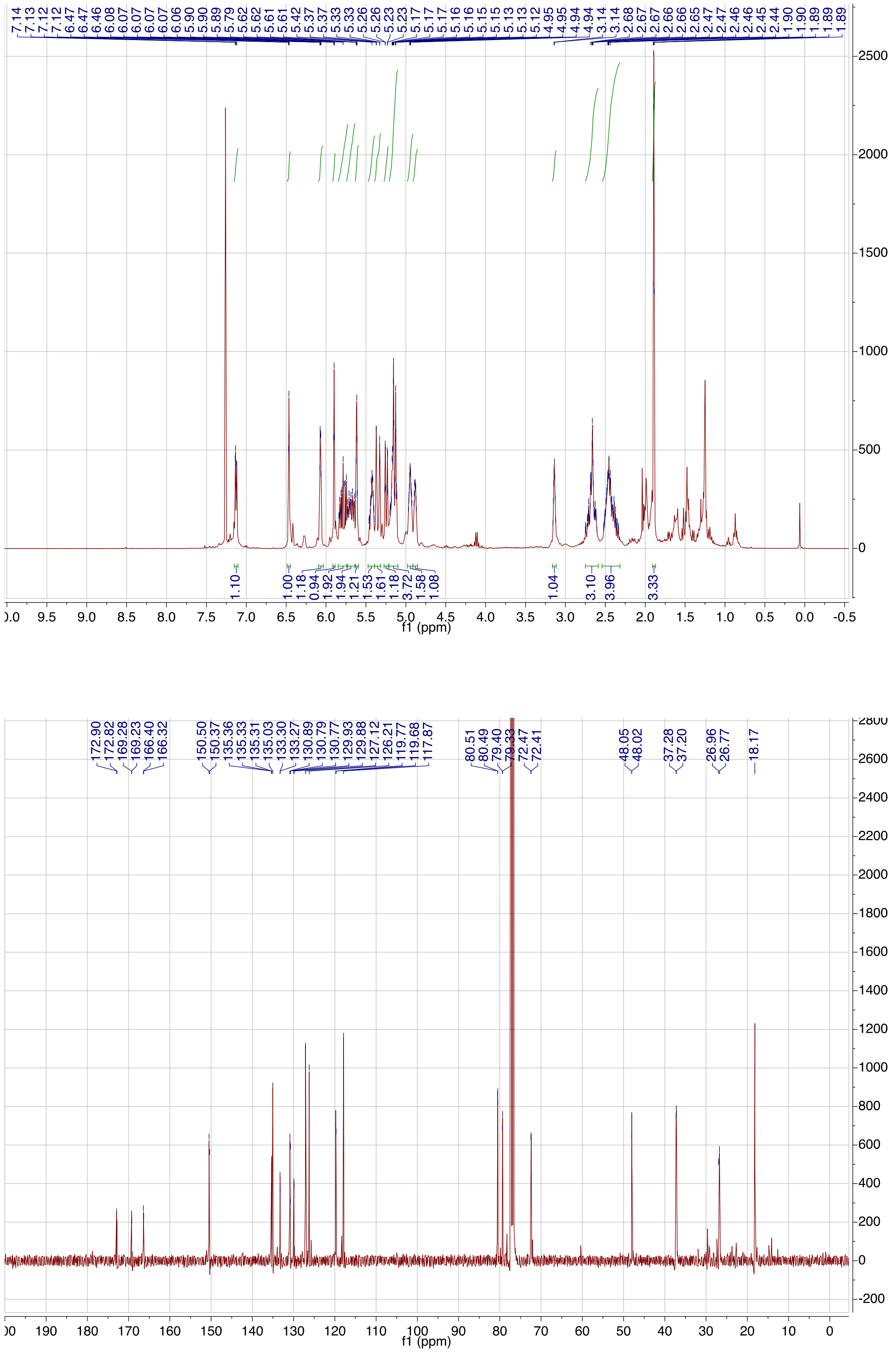

Supplementary figure 47: ^{1}H and ^{13}C NMR spectra of compound ***(R)*-10**

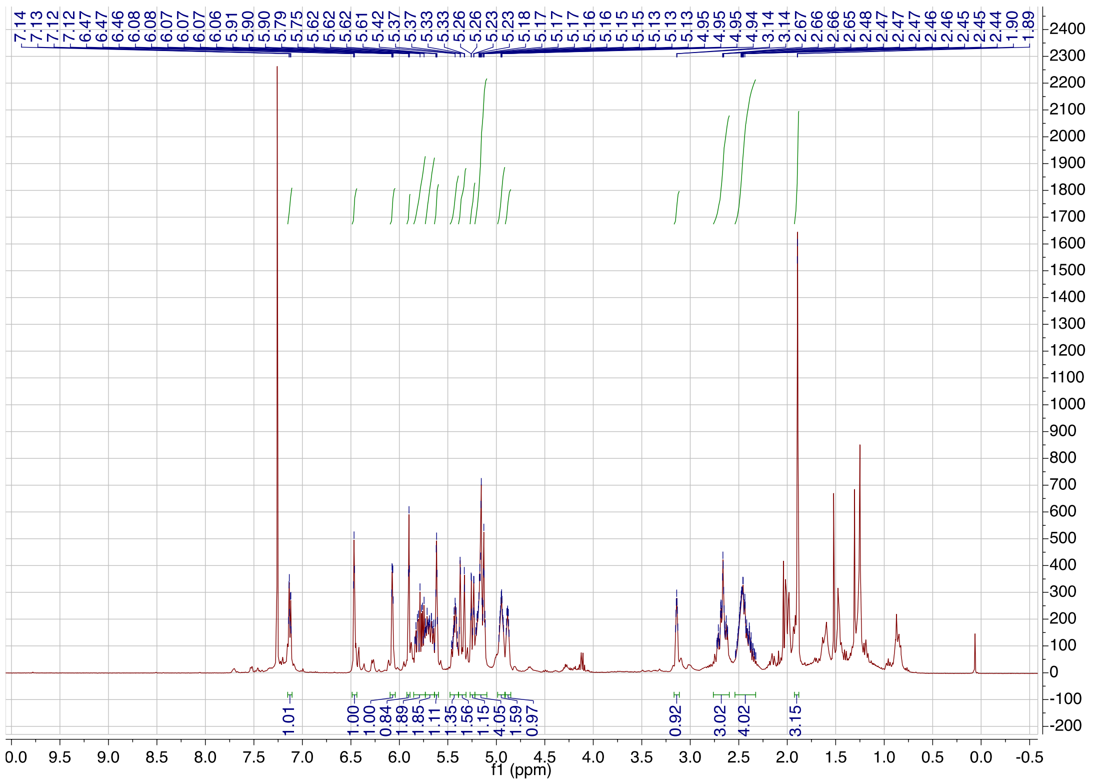

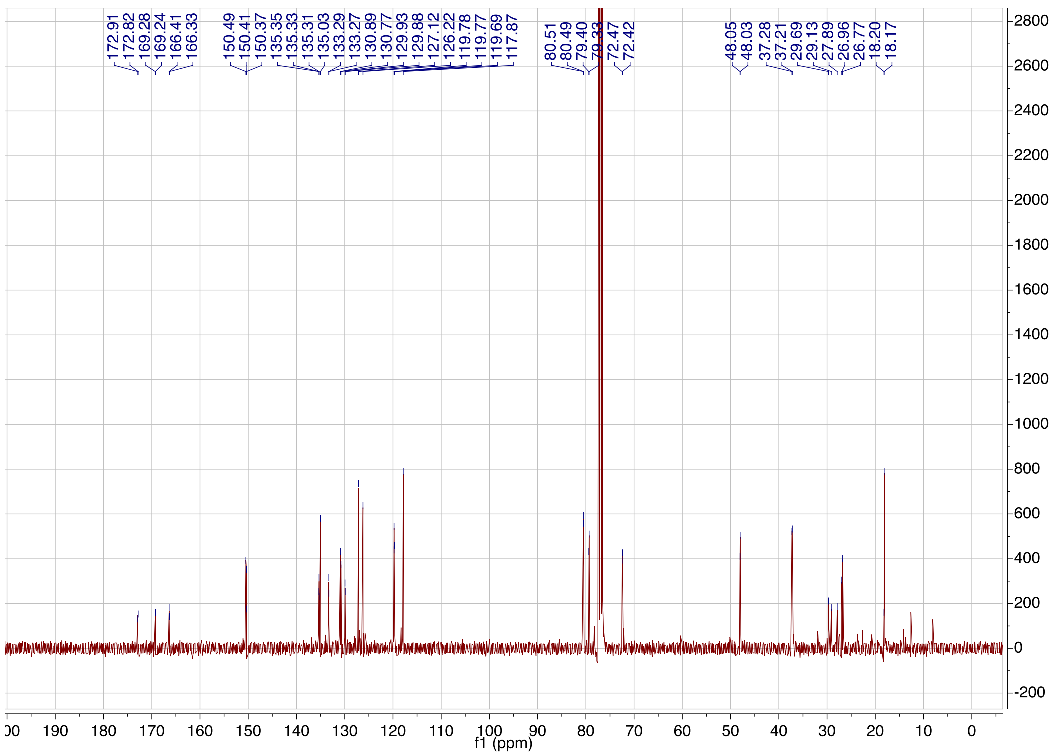

Supplementary figure 48: ^{1}H and ^{13}C NMR spectra of compound **(*S*)-10**

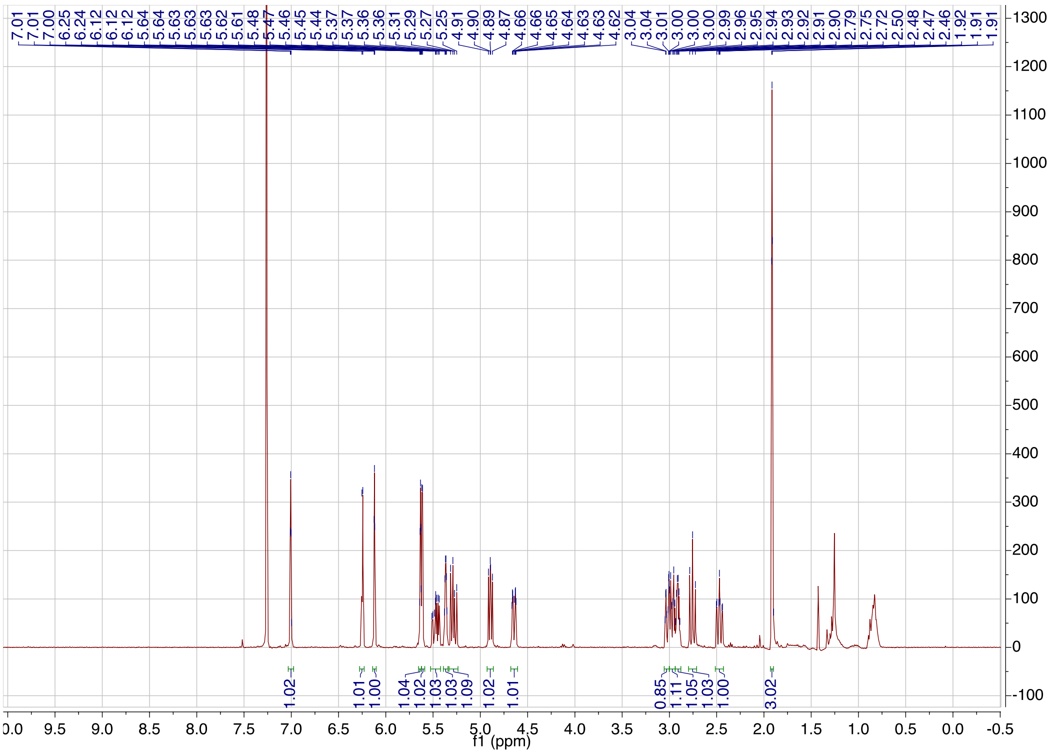

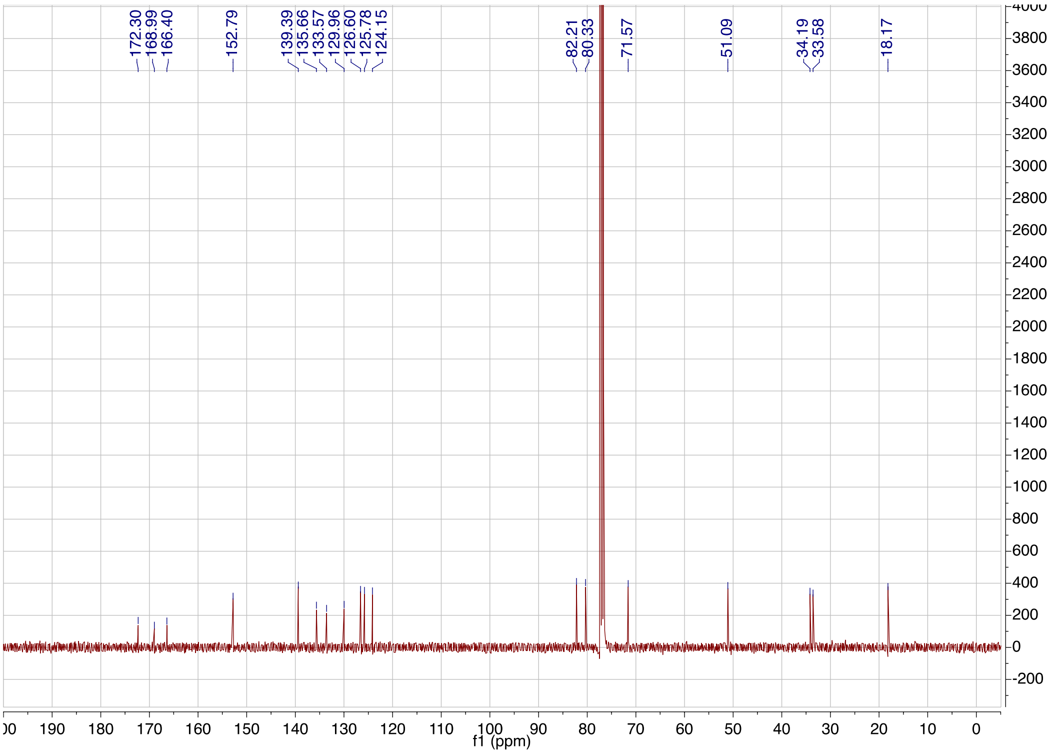

Supplementary figure 49: ^{1}H and ^{13}C NMR spectra of compound **Nordeoxyelephantopin**

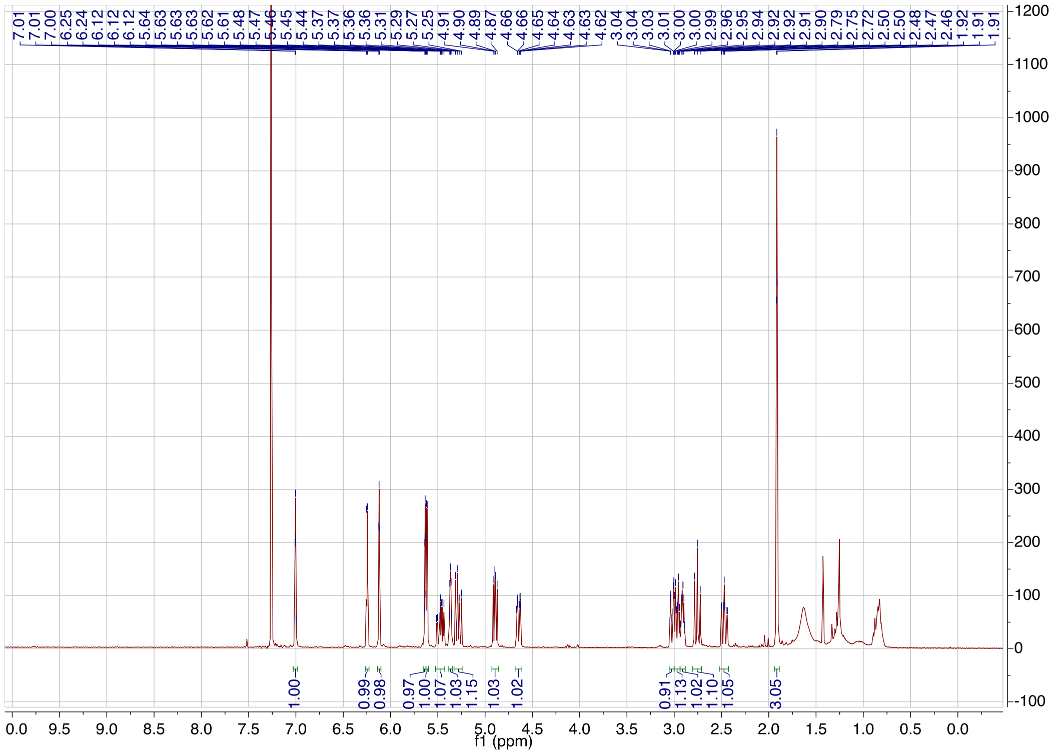

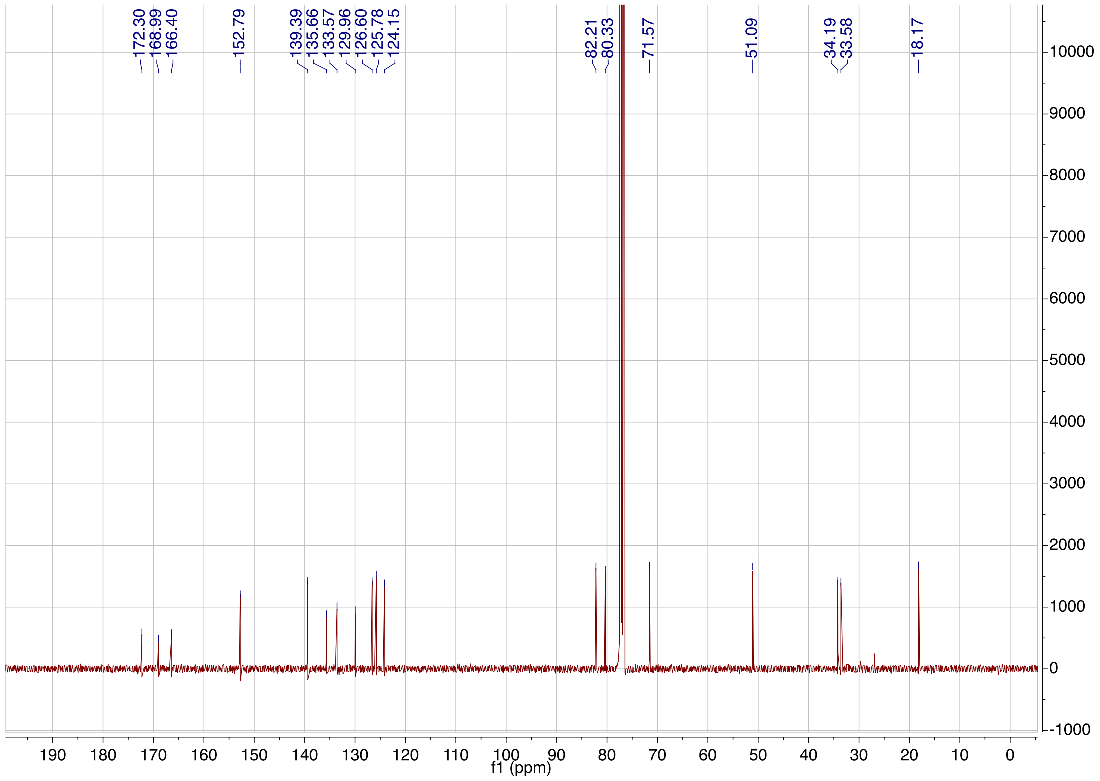

Supplementary figure 50: ^{1}H and ^{13}C NMR spectra of compound ***ent*-Nordeoxyelephantopin**

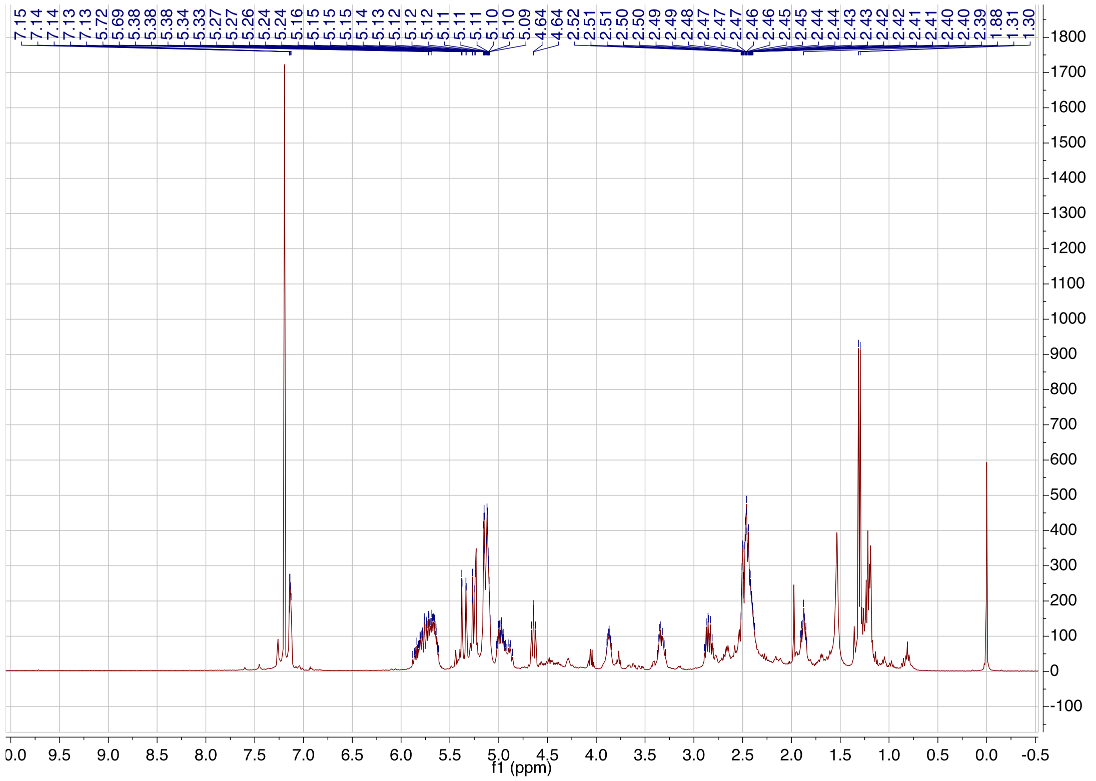

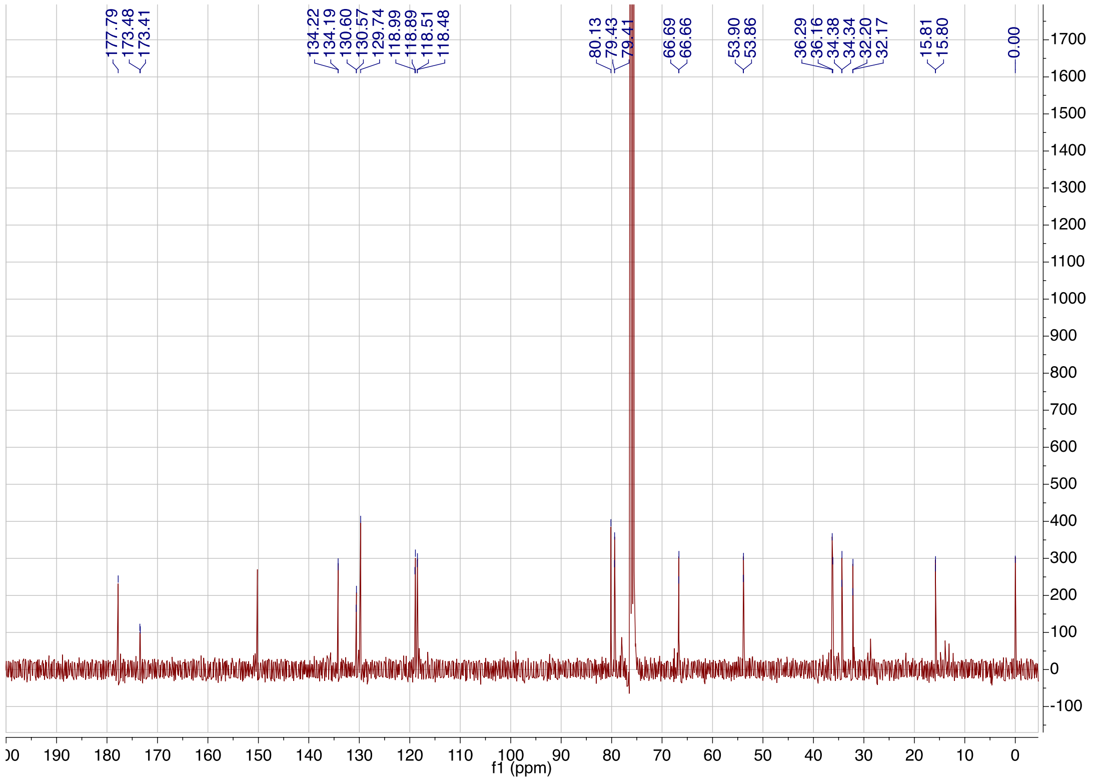

Supplementary figure 51: ^{1}H and ^{13}C NMR spectra of compound **S-8**

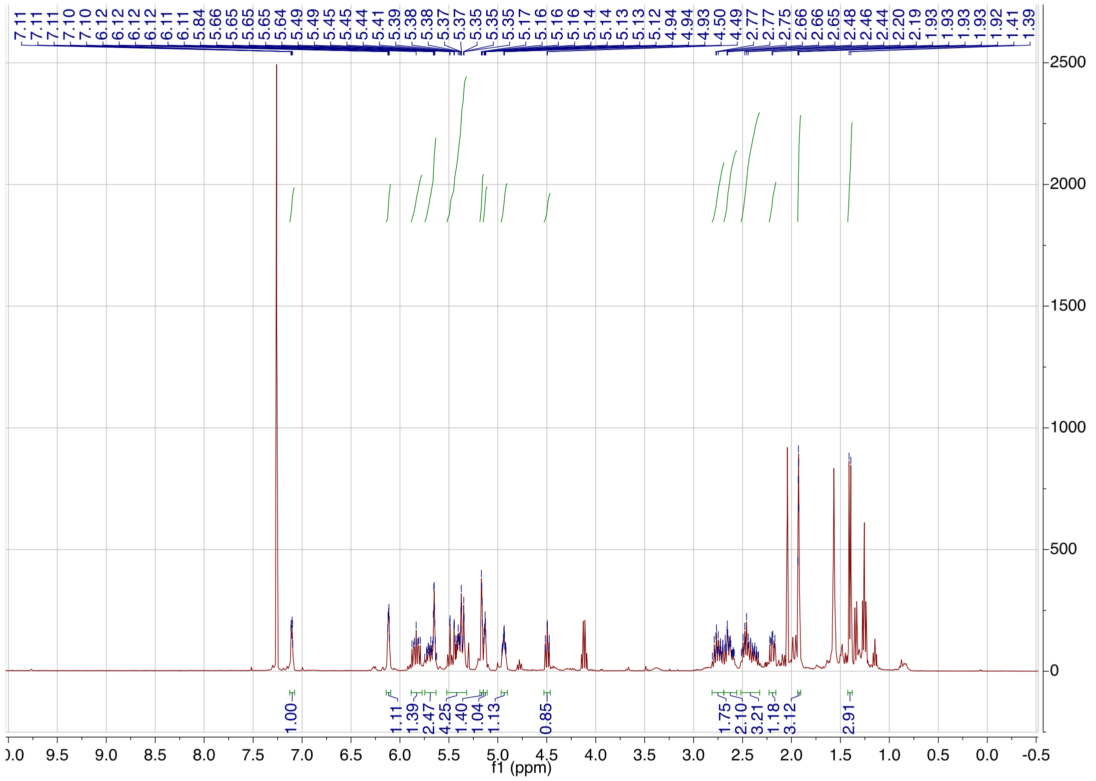

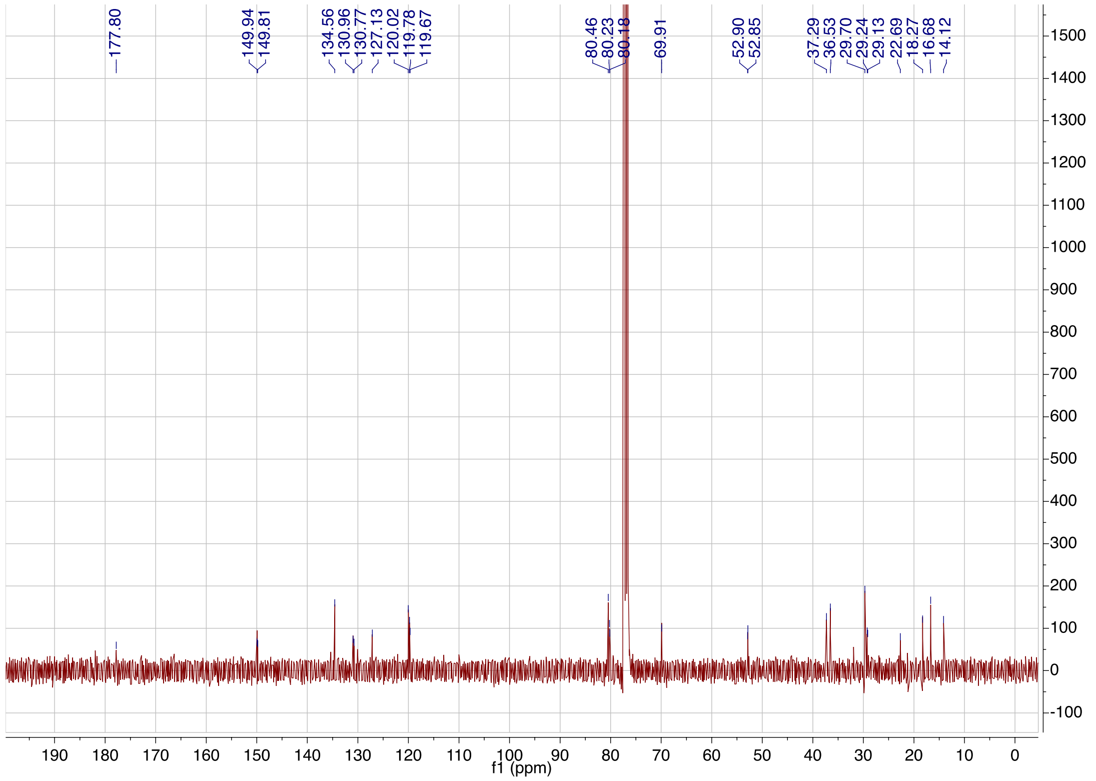

Supplementary figure 52: ^{1}H and ^{13}C NMR spectra of compound **S-9**

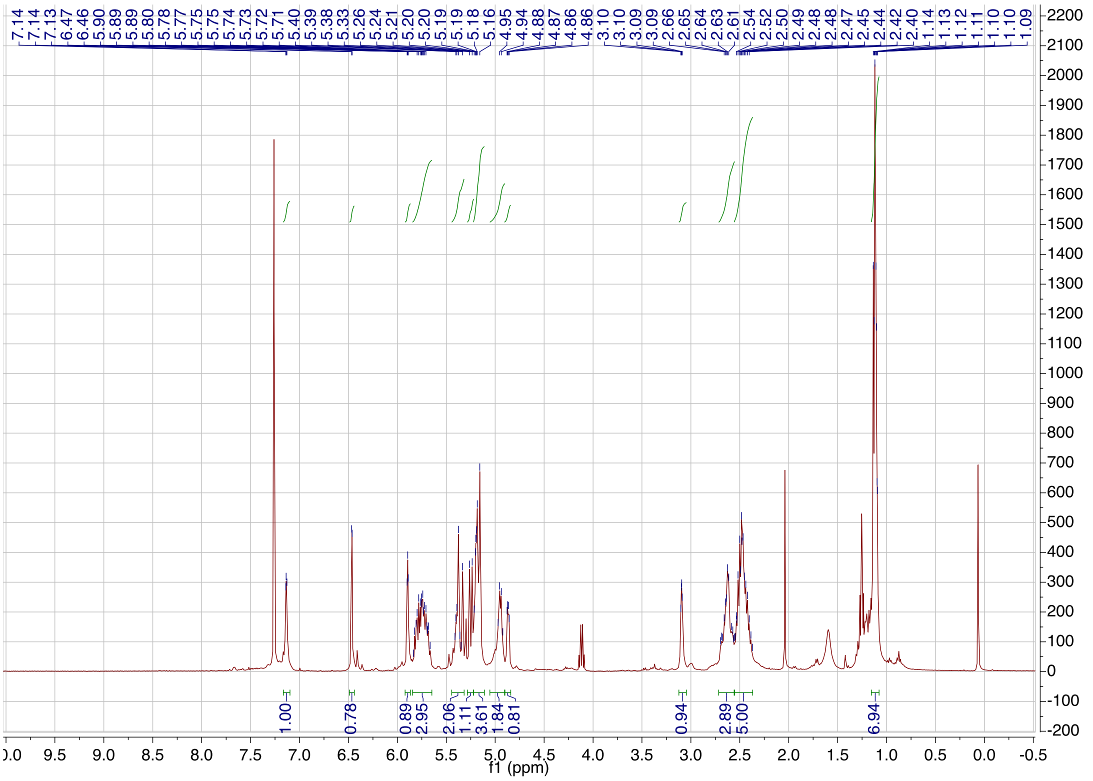

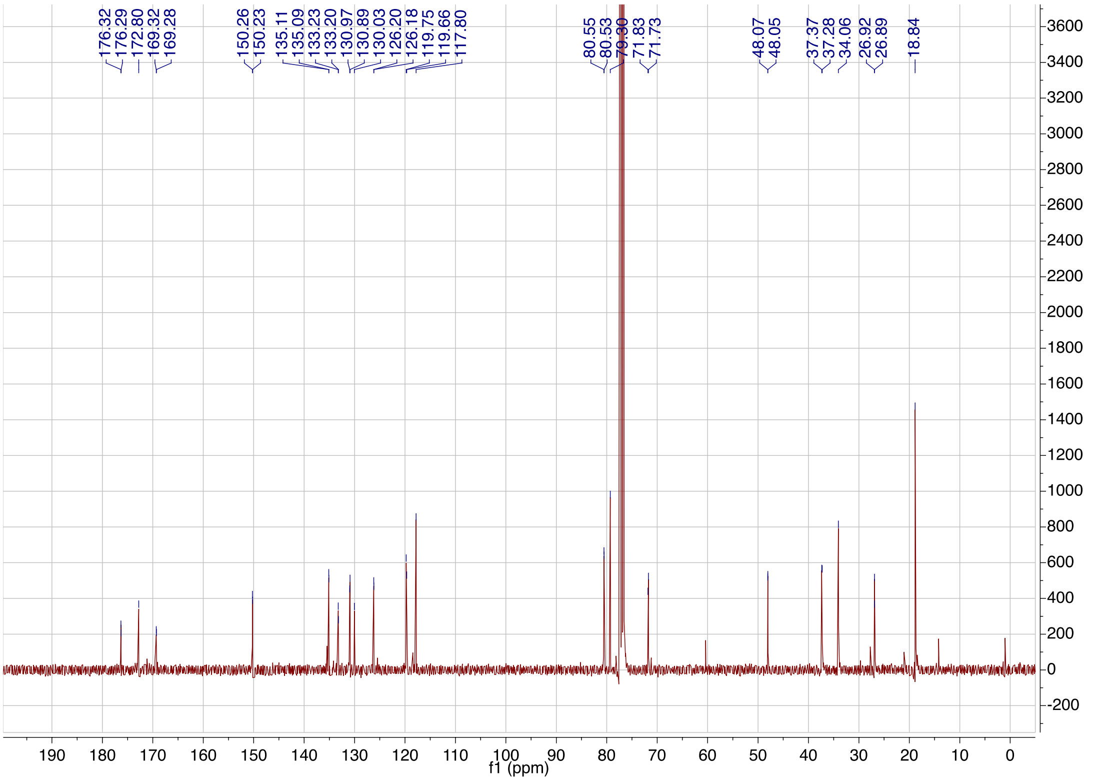

Supplementary figure 53: ^{1}H and ^{13}C NMR spectra of compound **S-10**

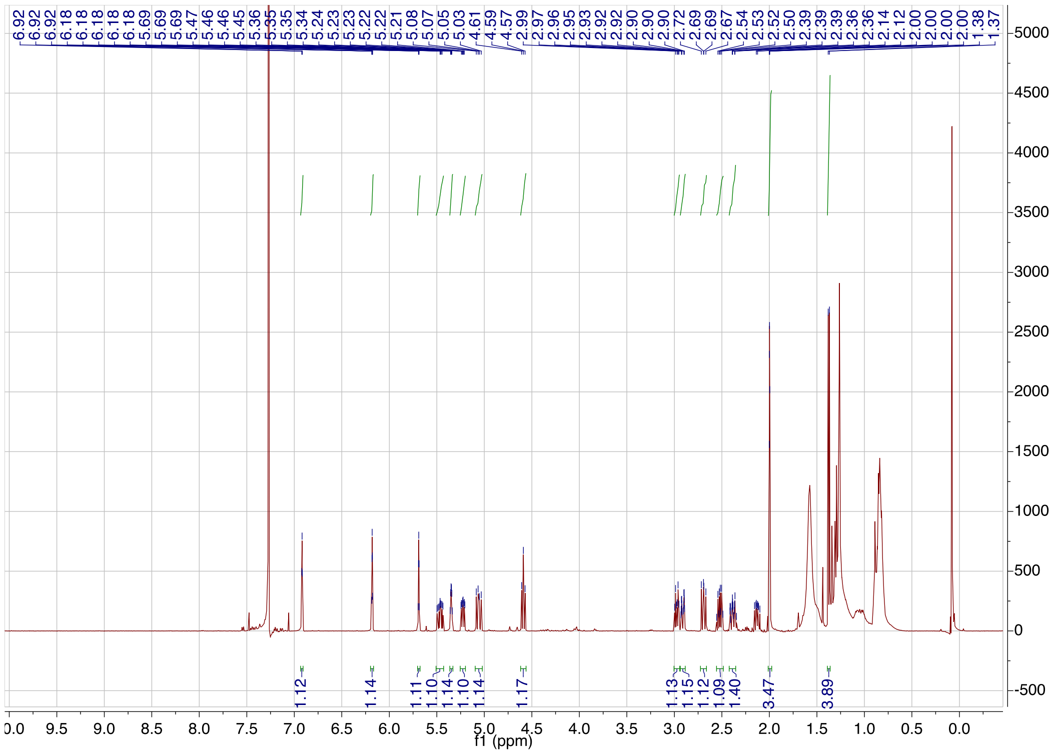

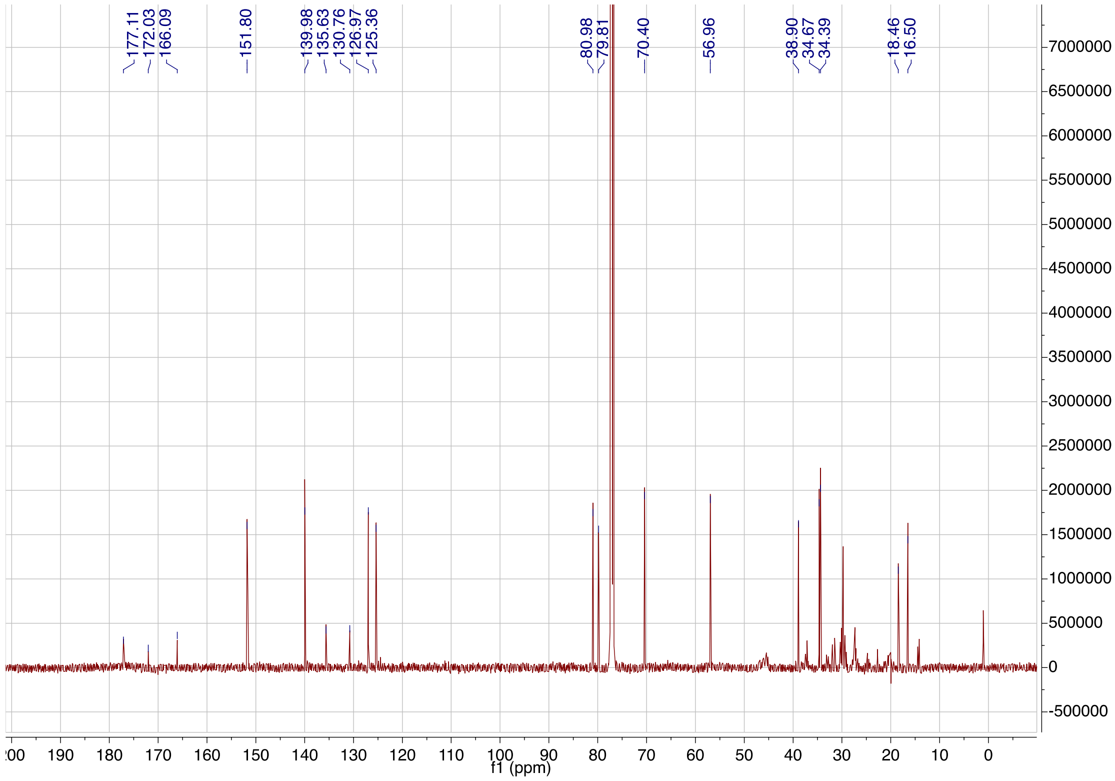

Supplementary figure 54: ^{1}H and ^{13}C NMR spectra of compound **11**

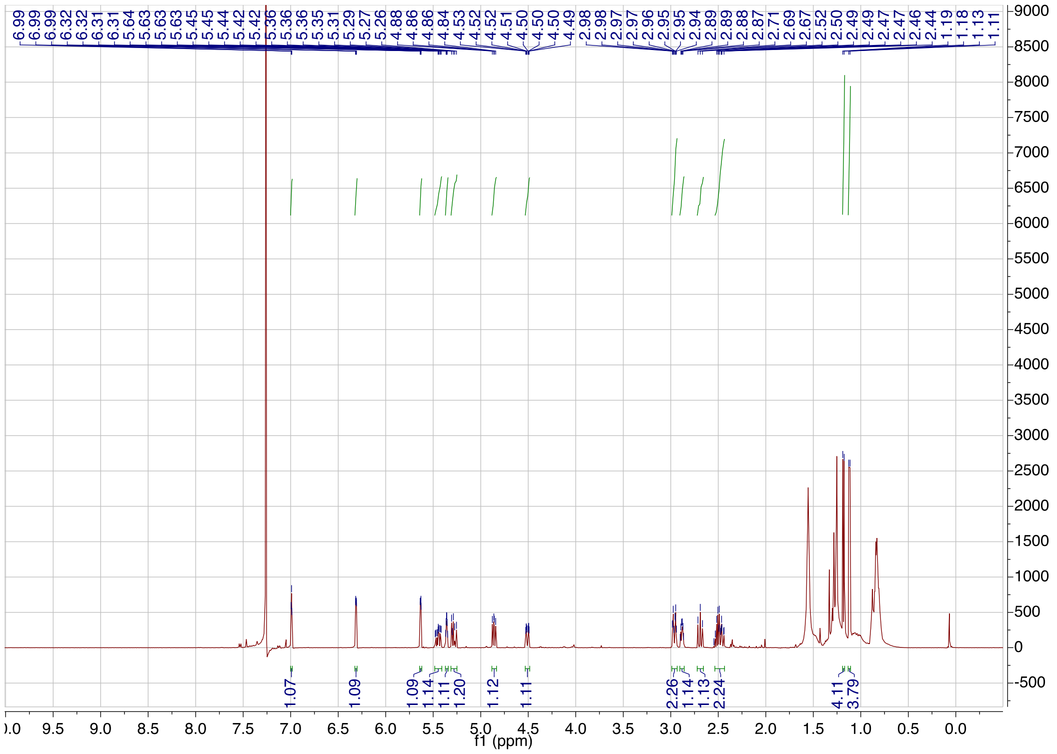

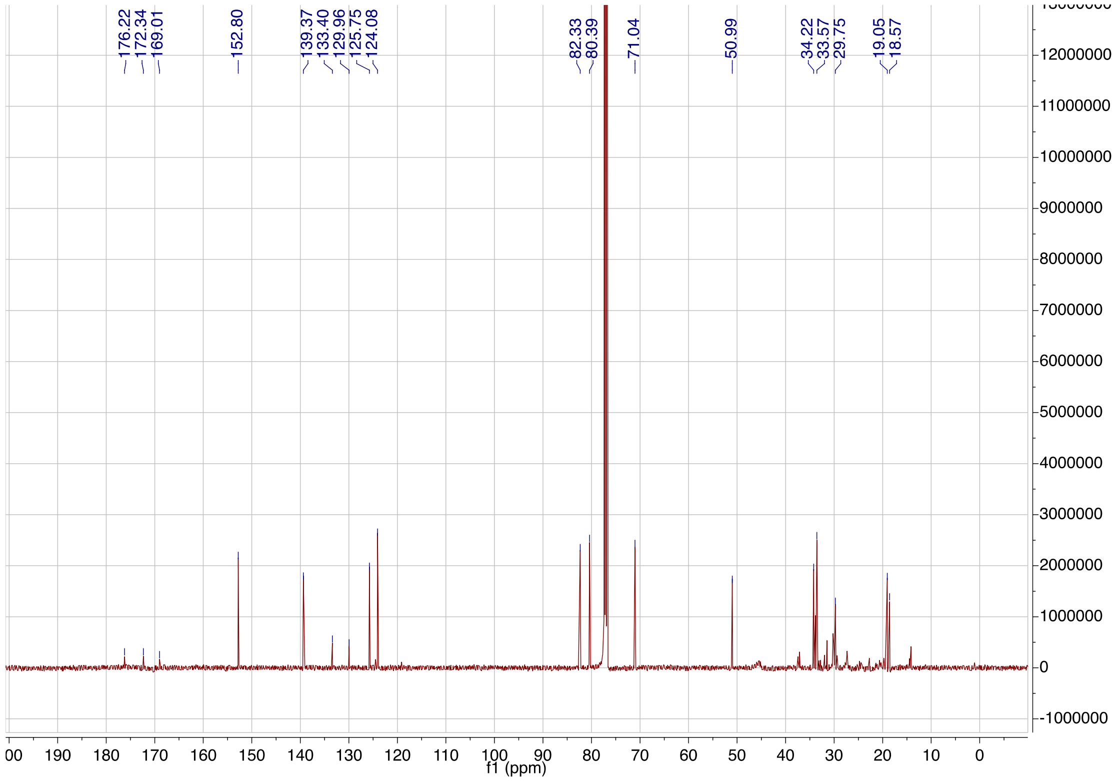

Supplementary figure 55: ^{1}H and ^{13}C NMR spectra of compound **12**

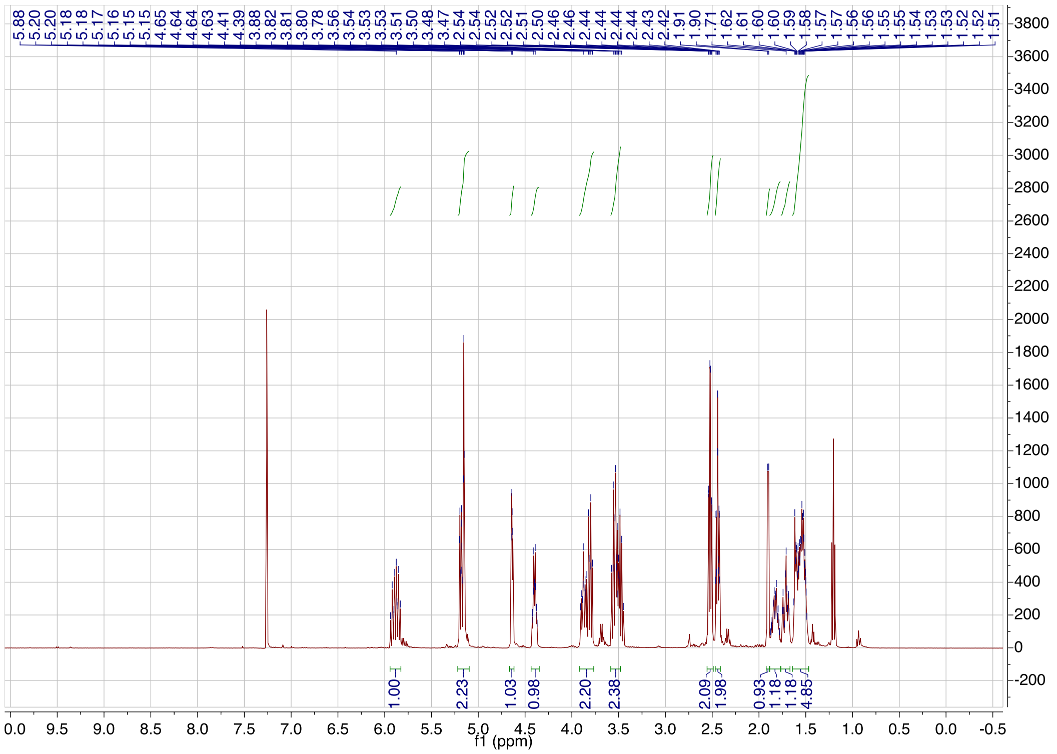

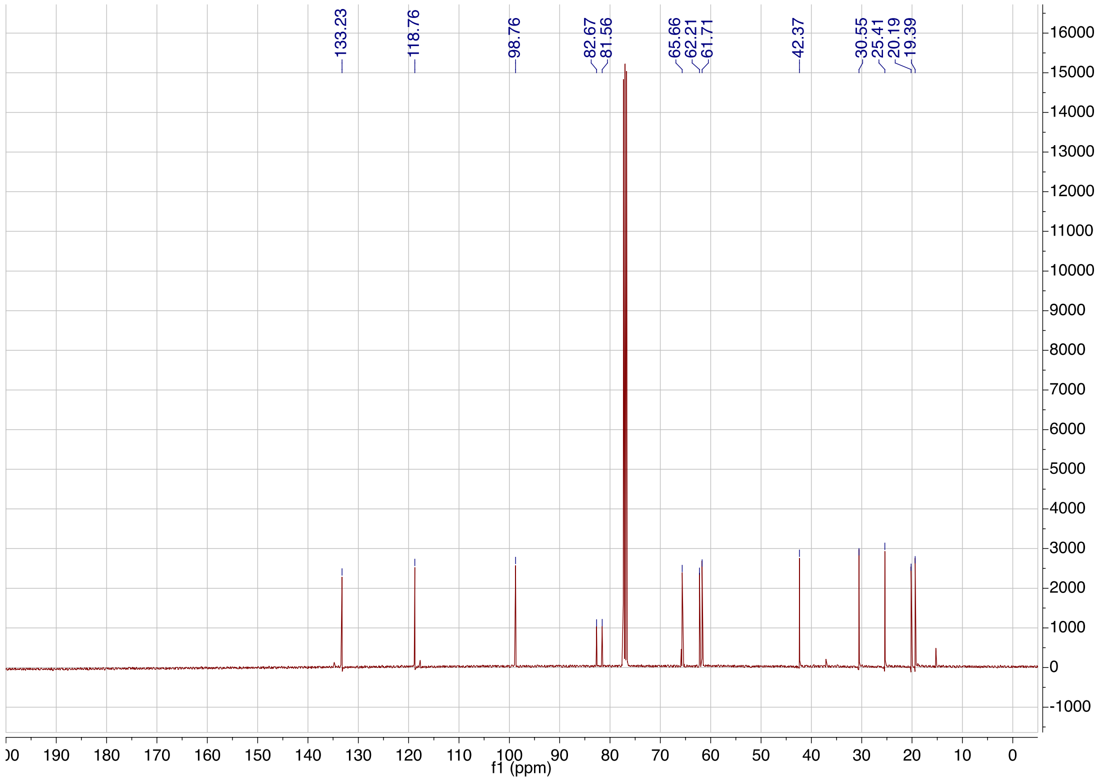

Supplementary figure 56: ^{1}H and ^{13}C NMR spectra of compound **S-13**

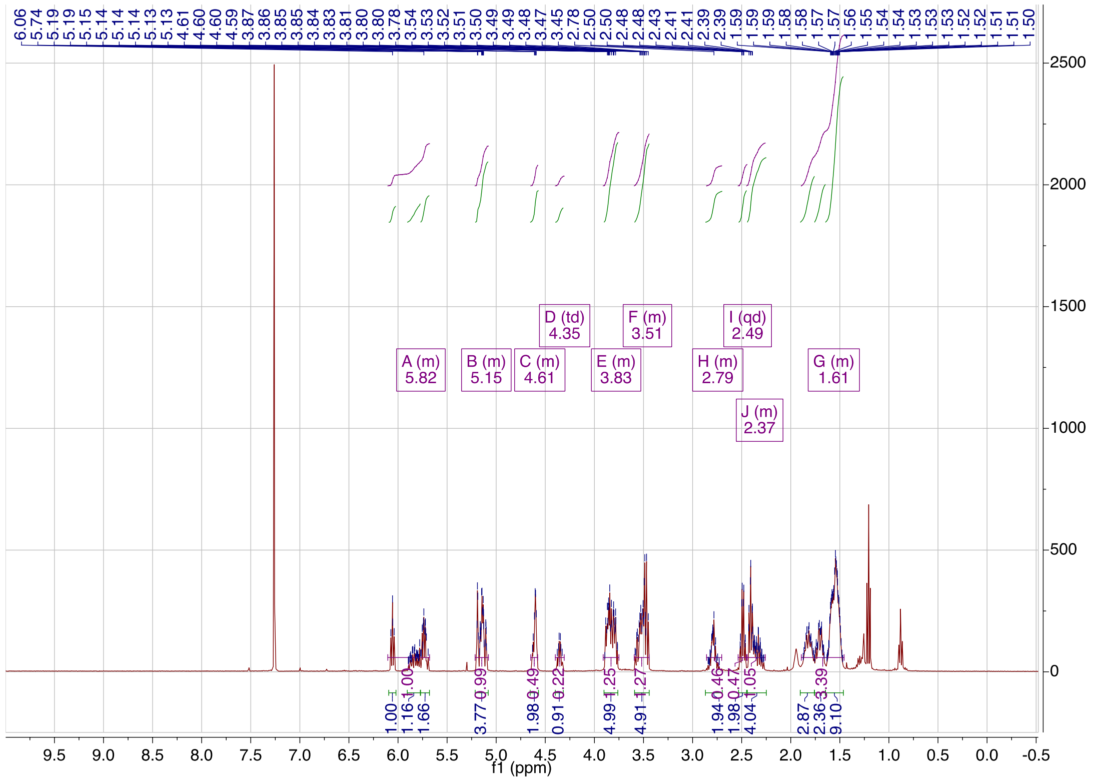

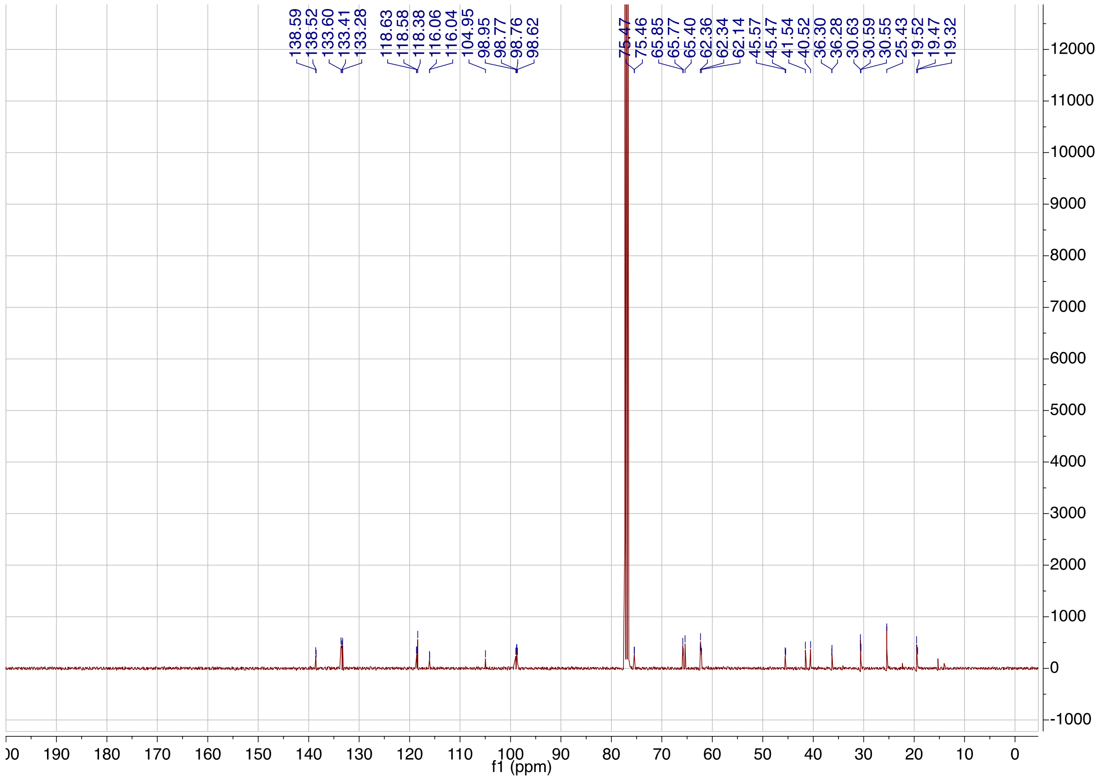

Supplementary figure 57: ^{1}H and ^{13}C NMR spectra of compound **15**

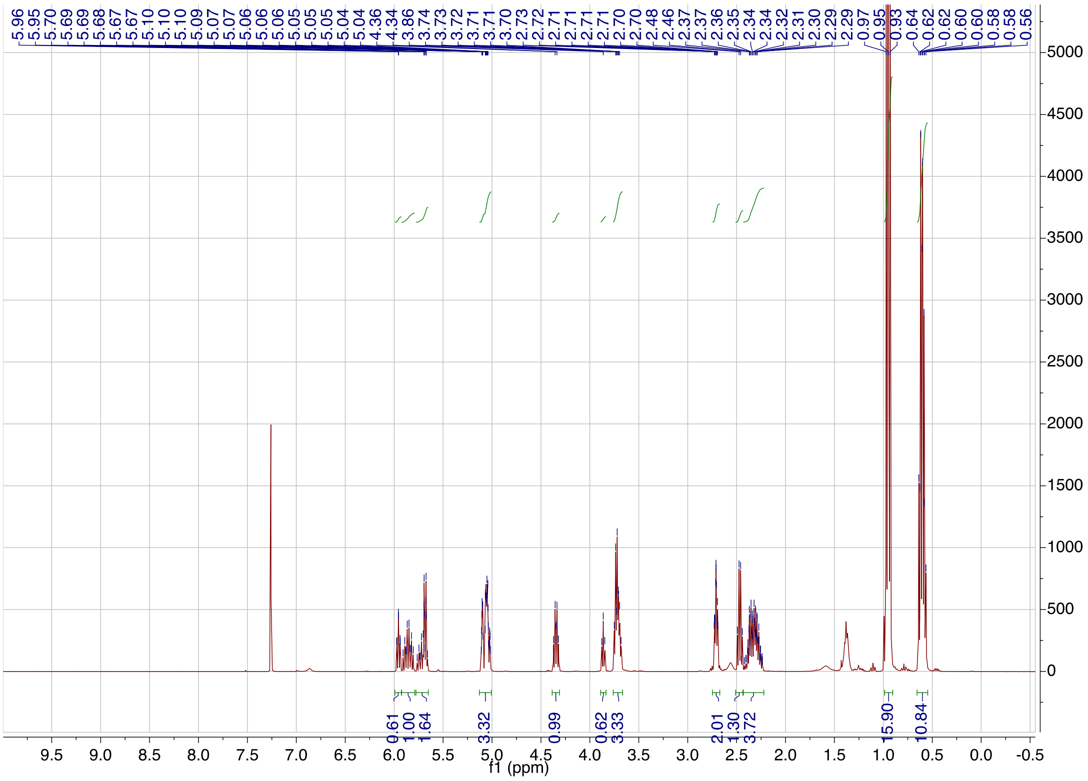

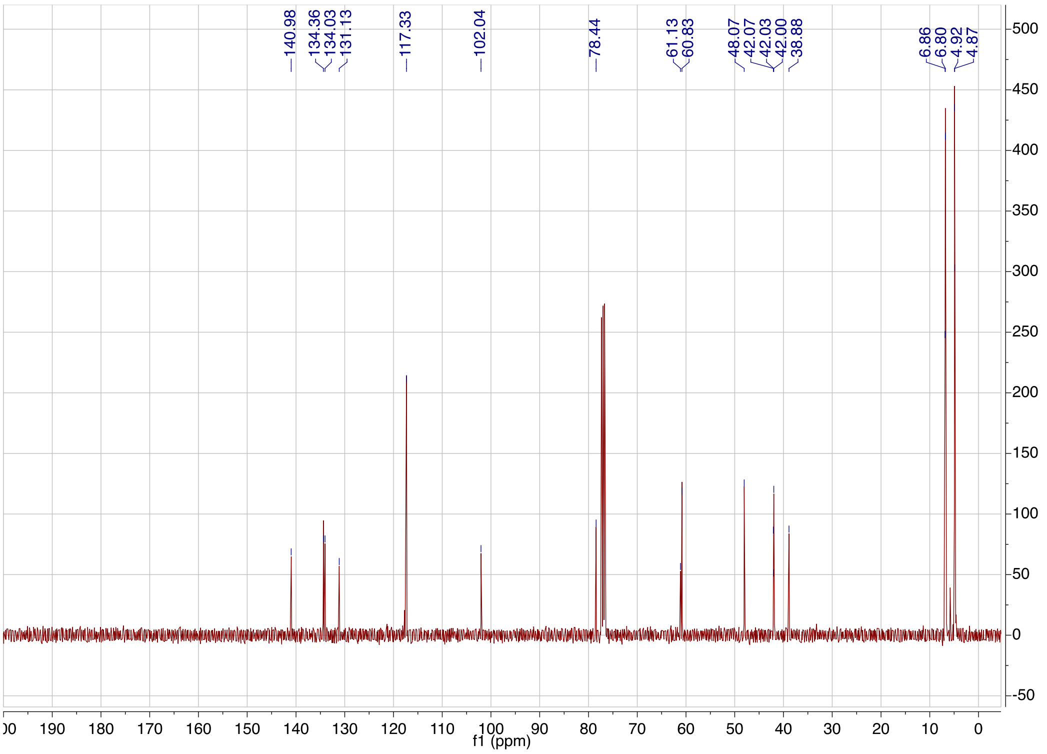

Supplementary figure 58: ^{1}H and ^{13}C NMR spectra of compound **S-14**

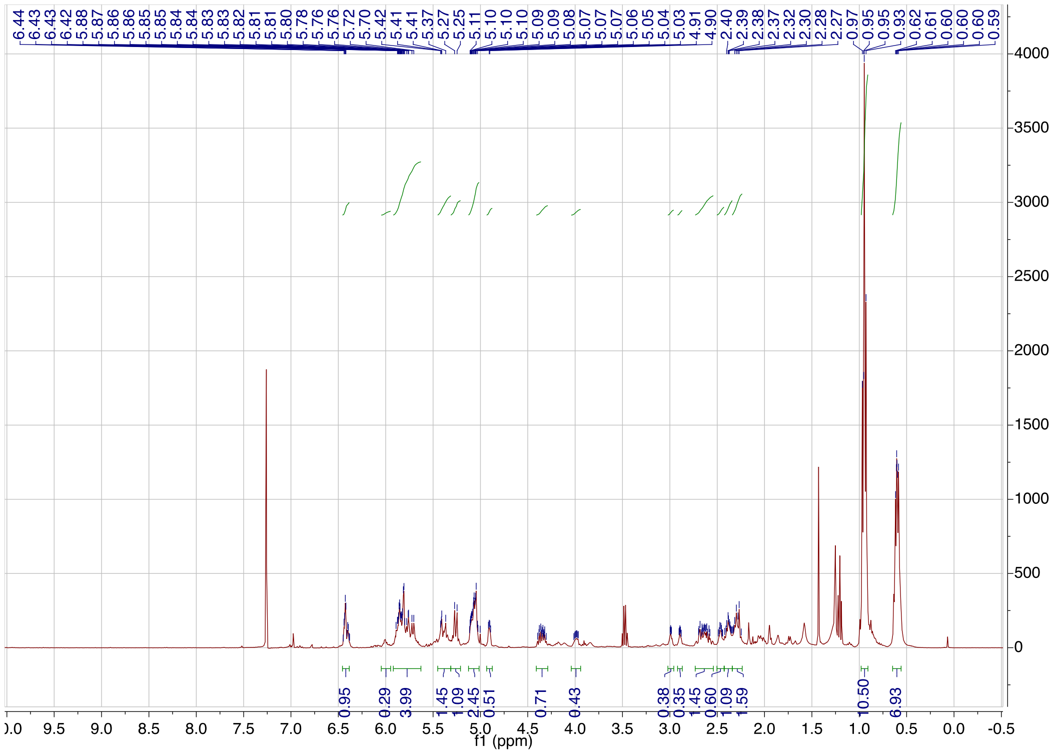

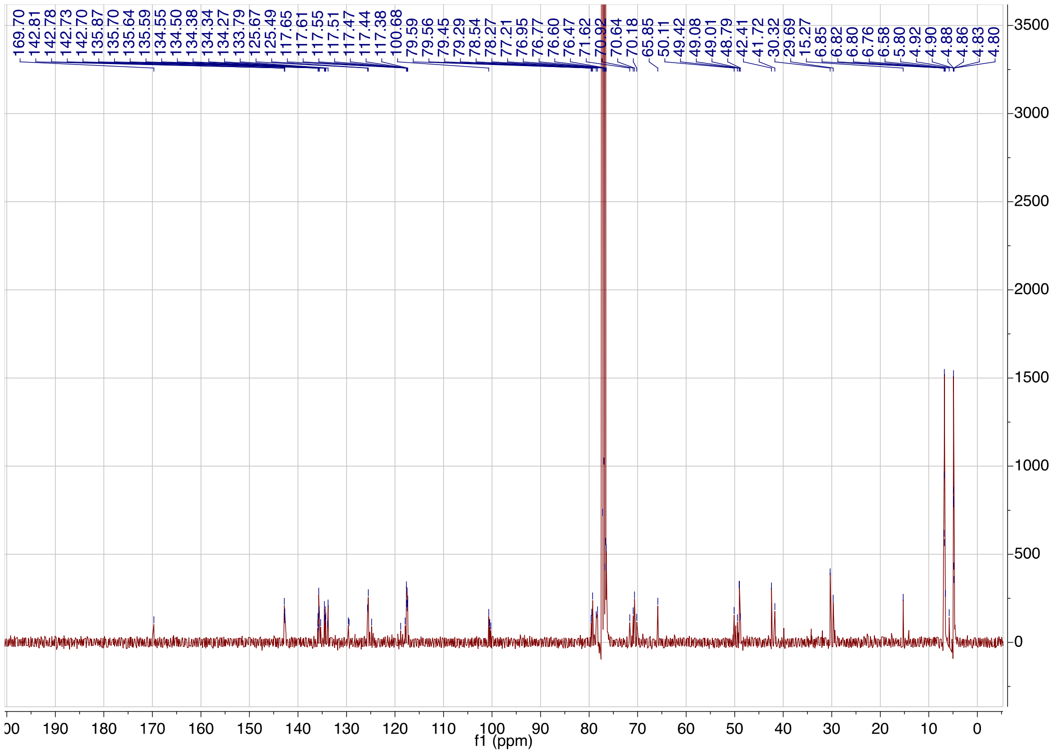

Supplementary figure 59: ^{1}H and ^{13}C NMR spectra of compound **16**

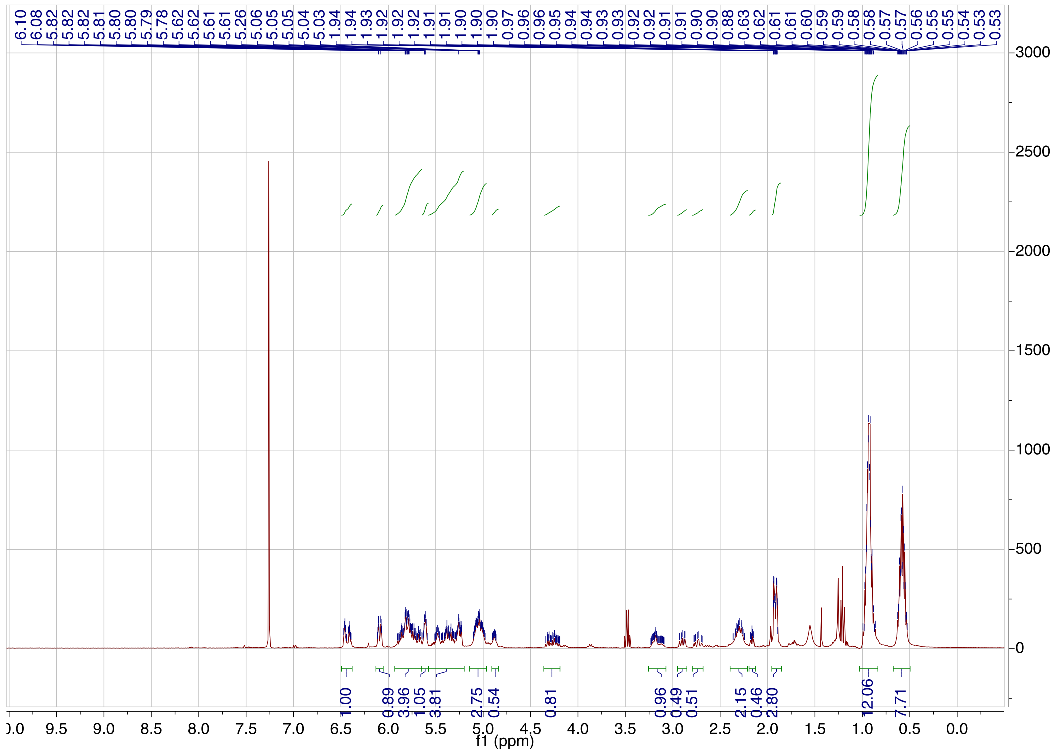

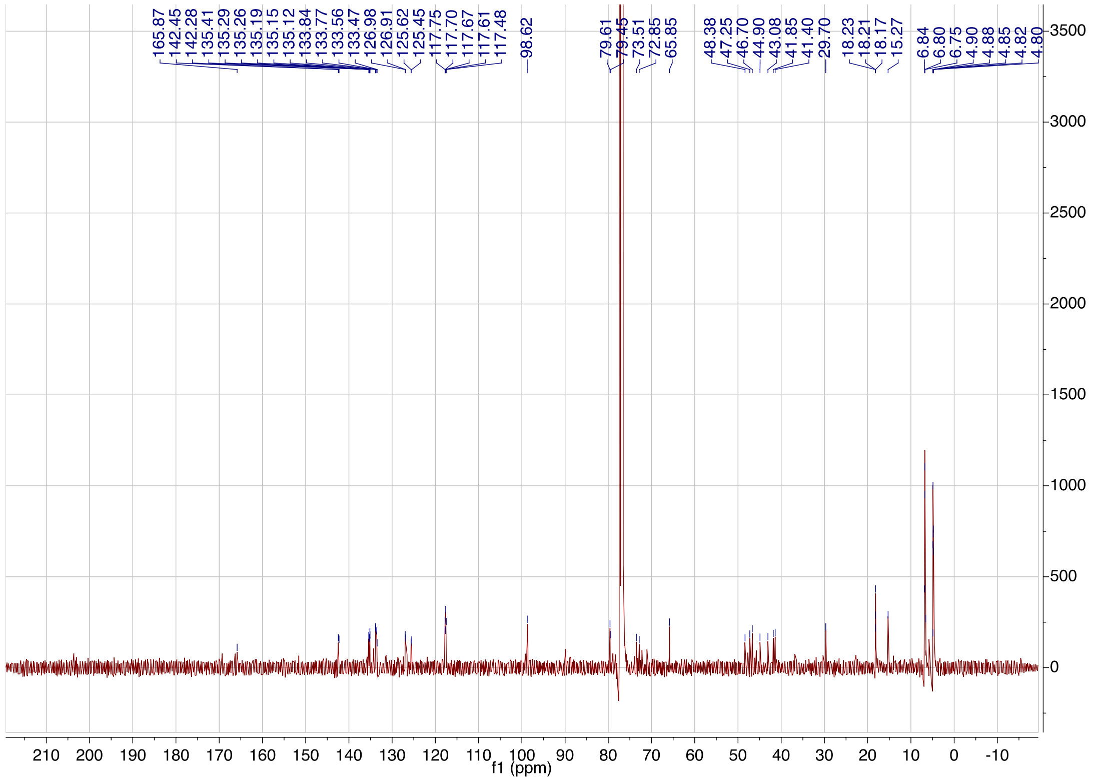

Supplementary figure 60: ^{1}H and ^{13}C NMR spectra of compound **S-16a**

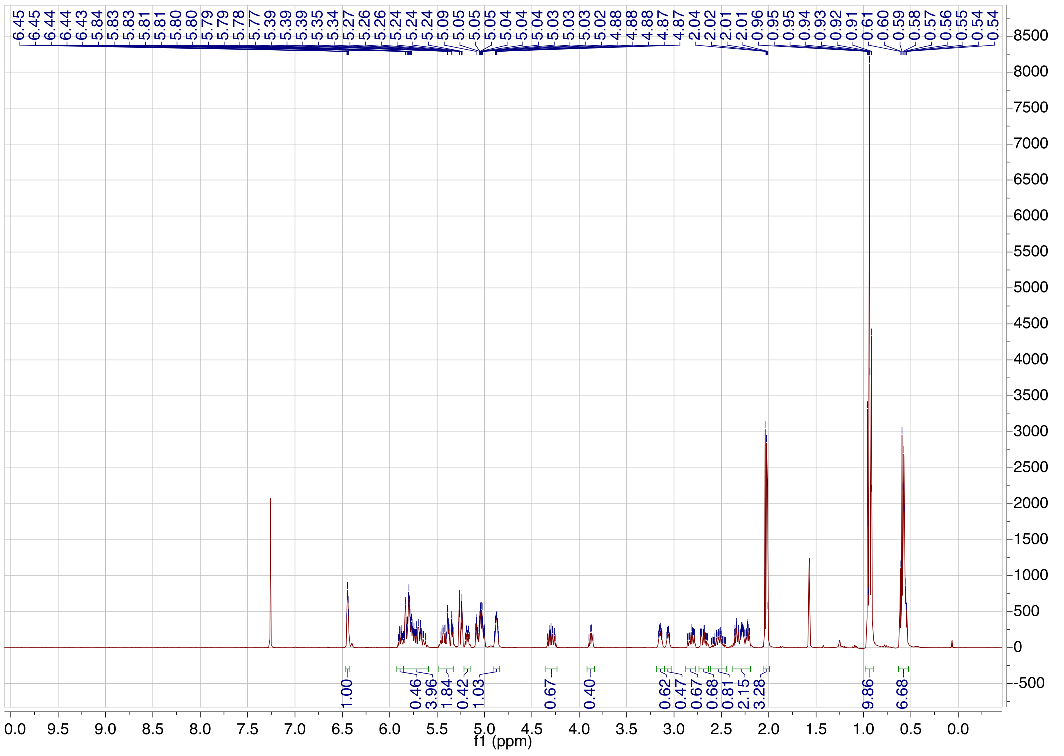

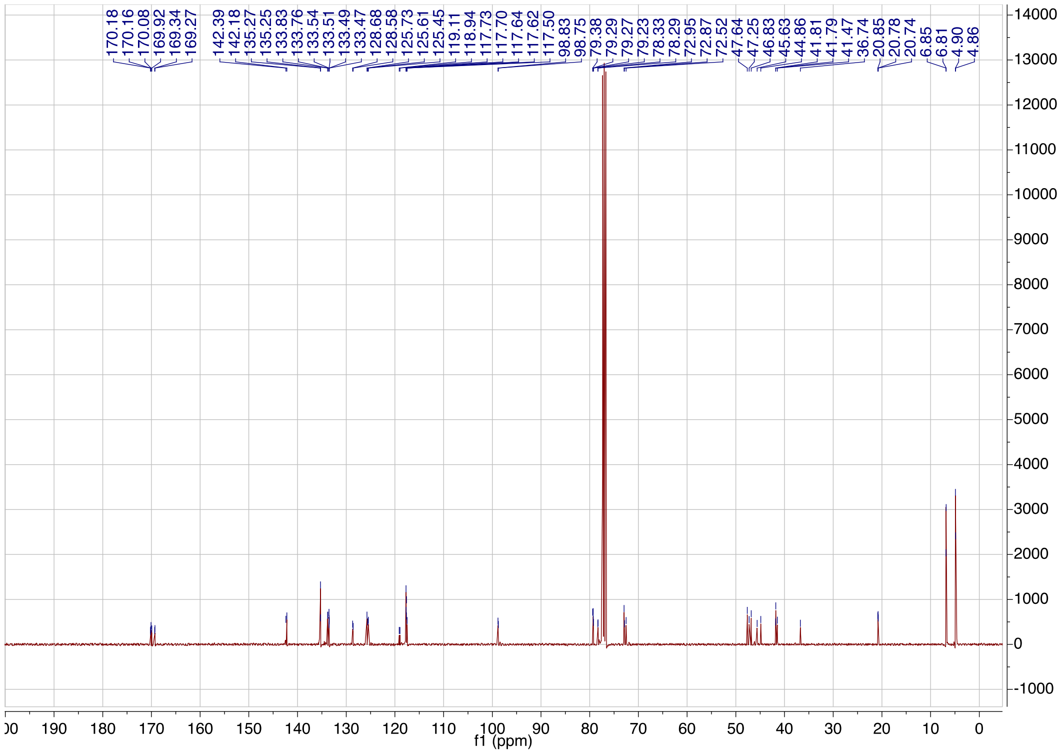

Supplementary figure 61: ^{1}H and ^{13}C NMR spectra of compound **S-16b**

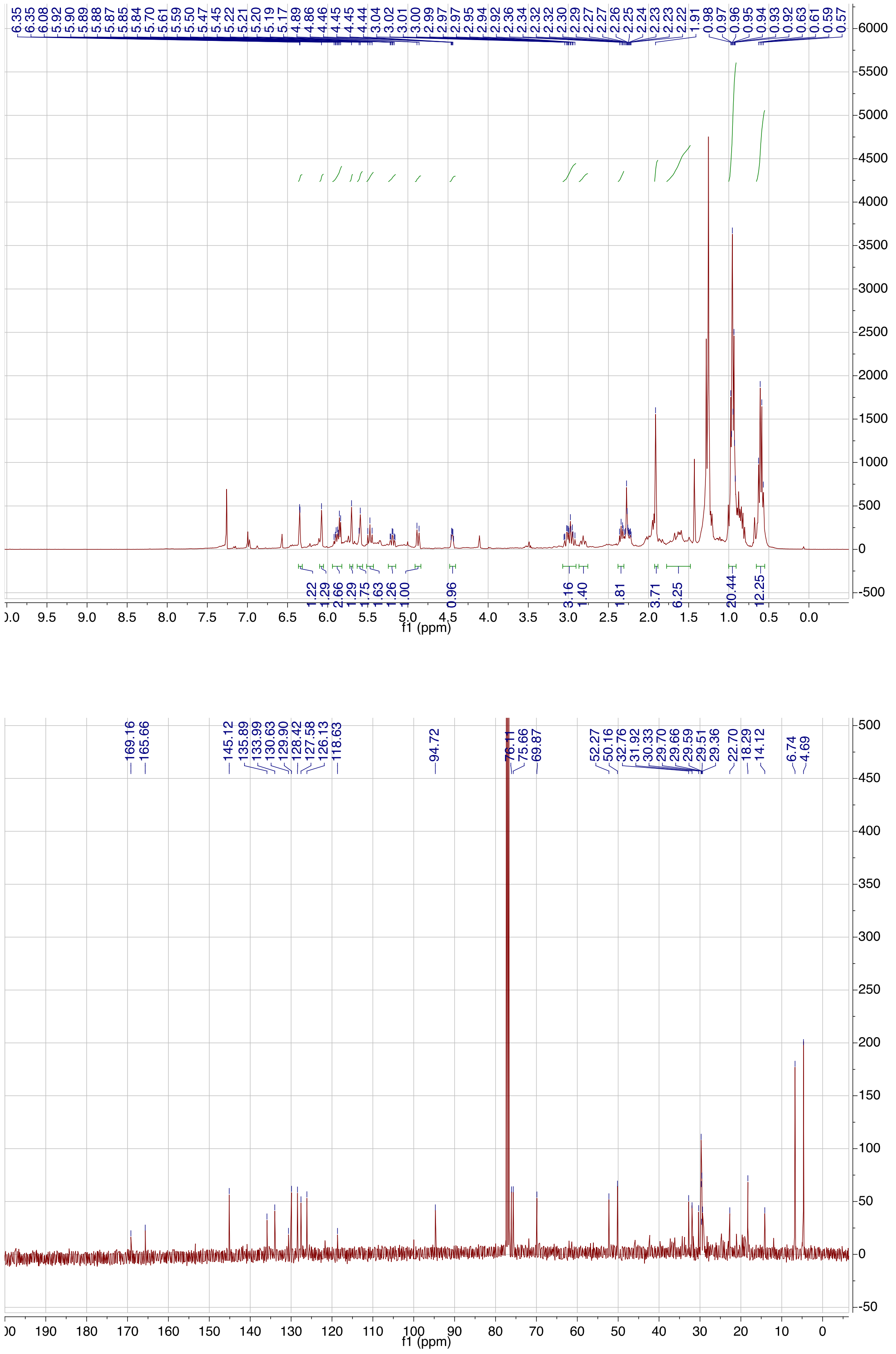

Supplementary figure 62: ^{1}H and ^{13}C NMR spectra of compound **17a**

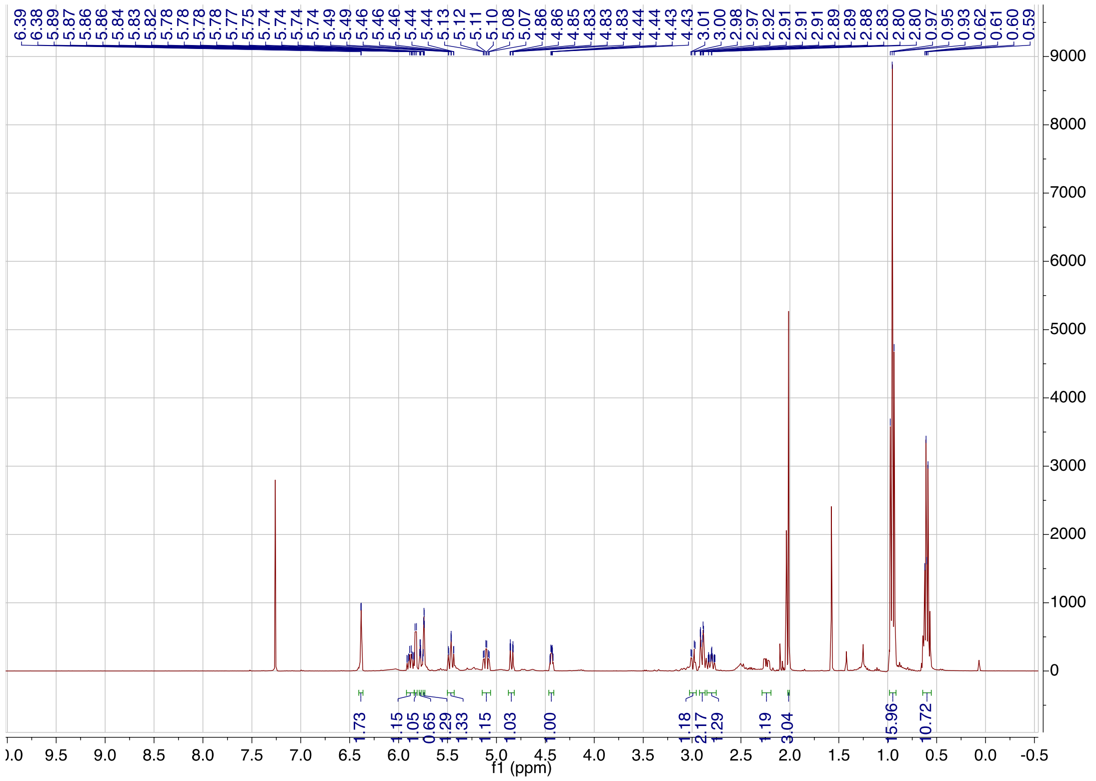

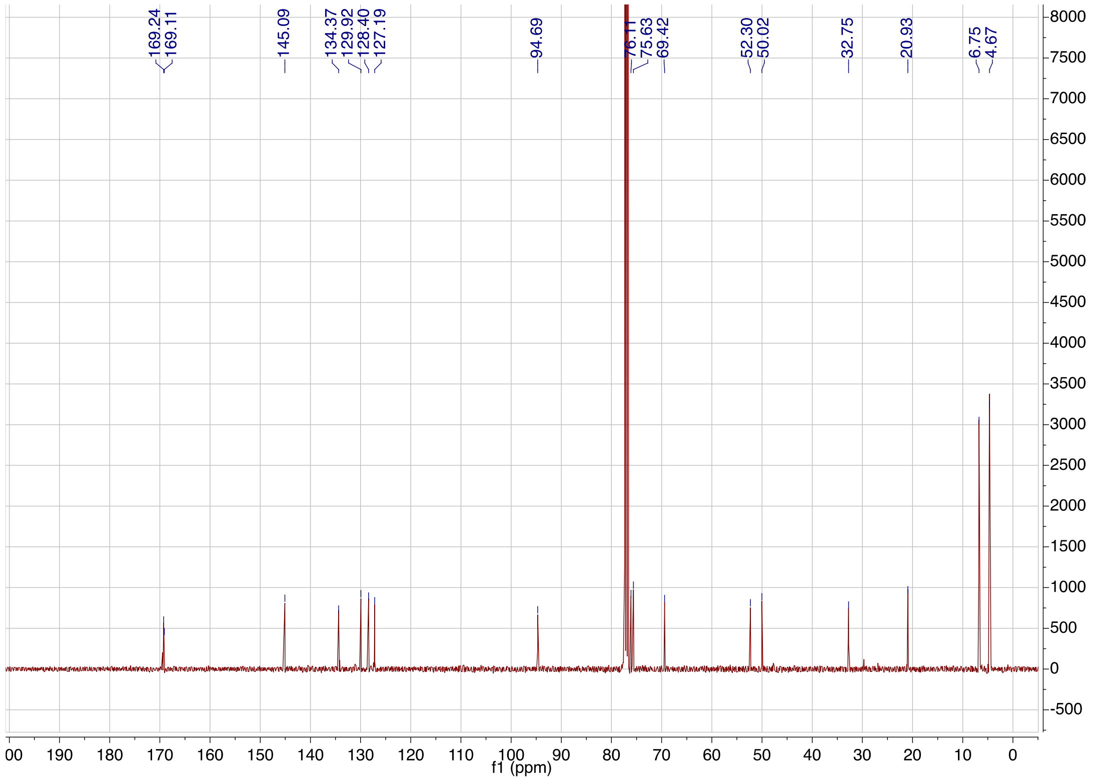

Supplementary figure 63: ^{1}H and ^{13}C NMR spectra of compound **17b**

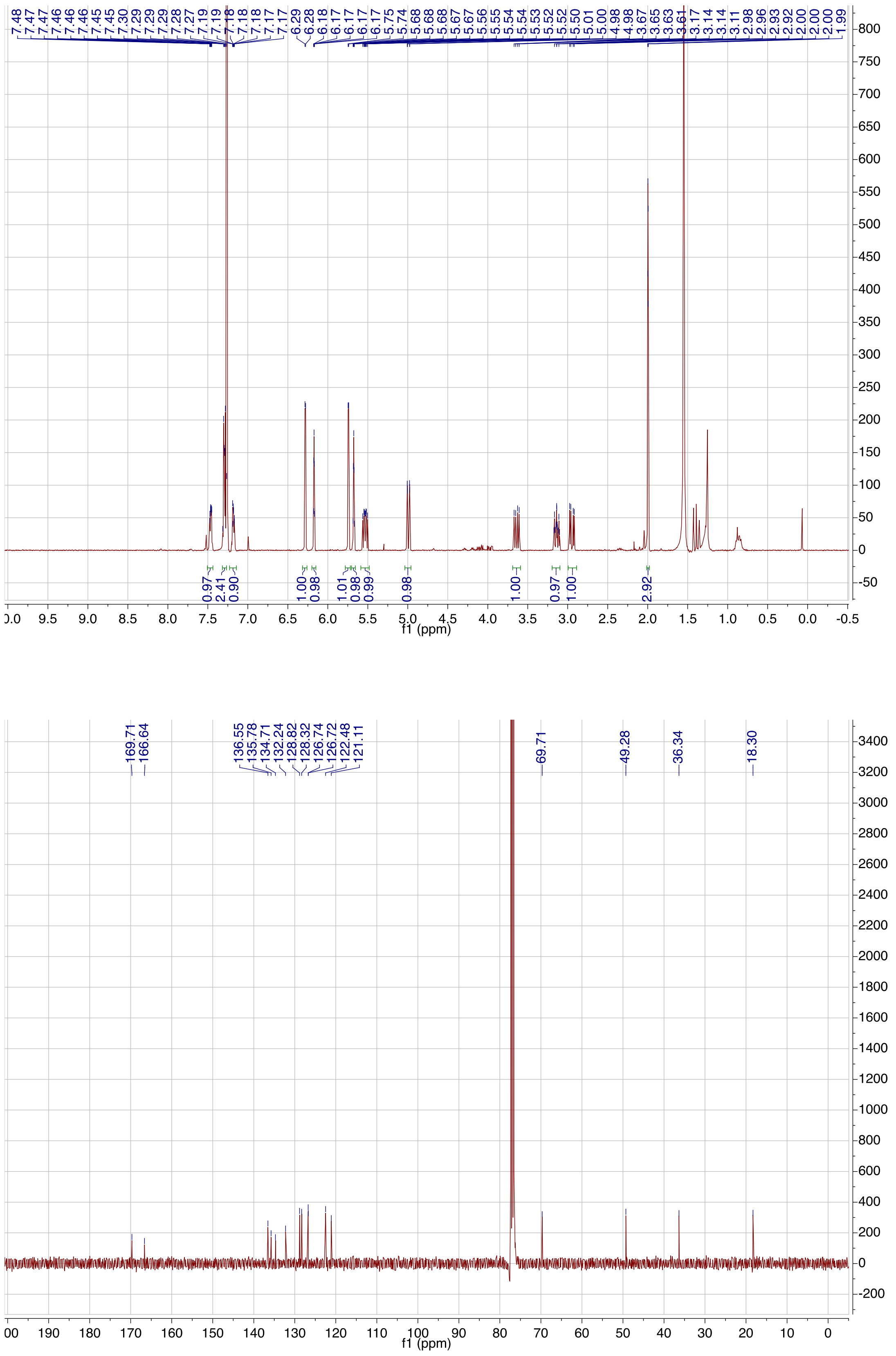

Supplementary figure 64: ^{1}H and ^{13}C NMR spectra of compound **18**

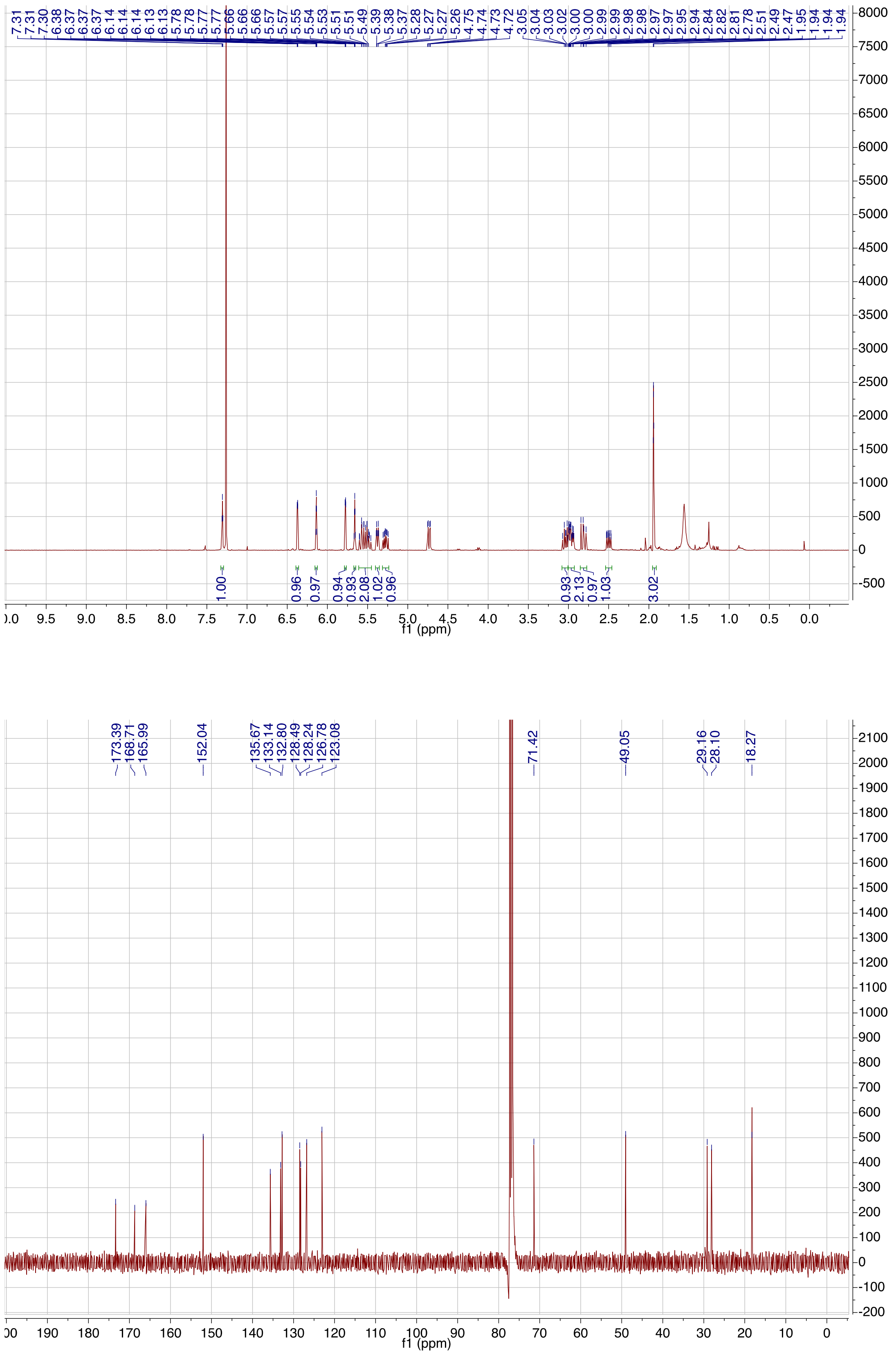

Supplementary figure 65: ^{1}H and ^{13}C NMR spectra of compound **19a**

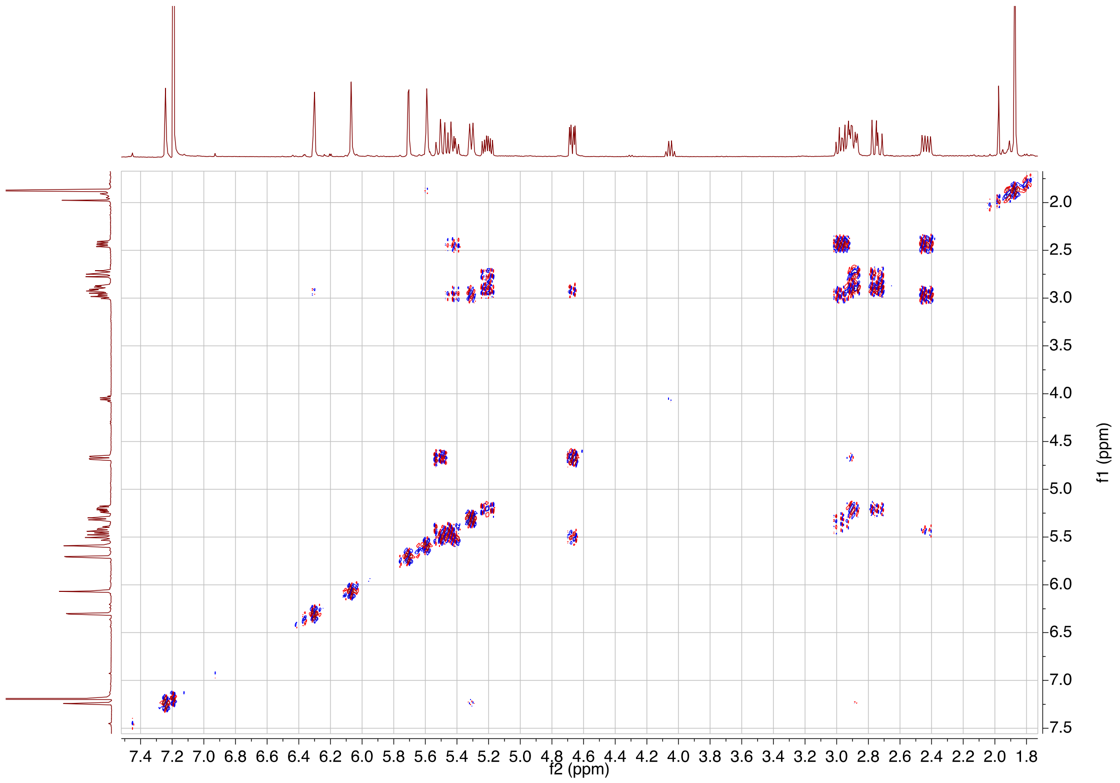

Supplementary figure 66: COSY spectrum of compound **19a**

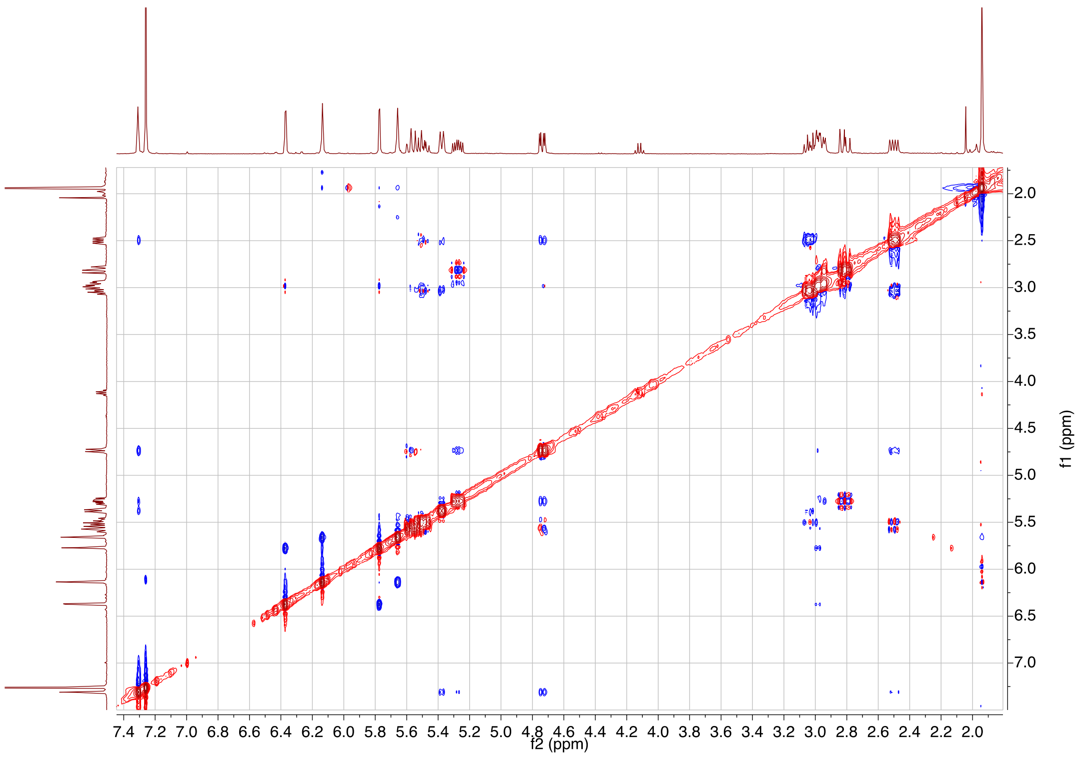

Supplementary figure 67: NOESY spectrum of compound **19a**

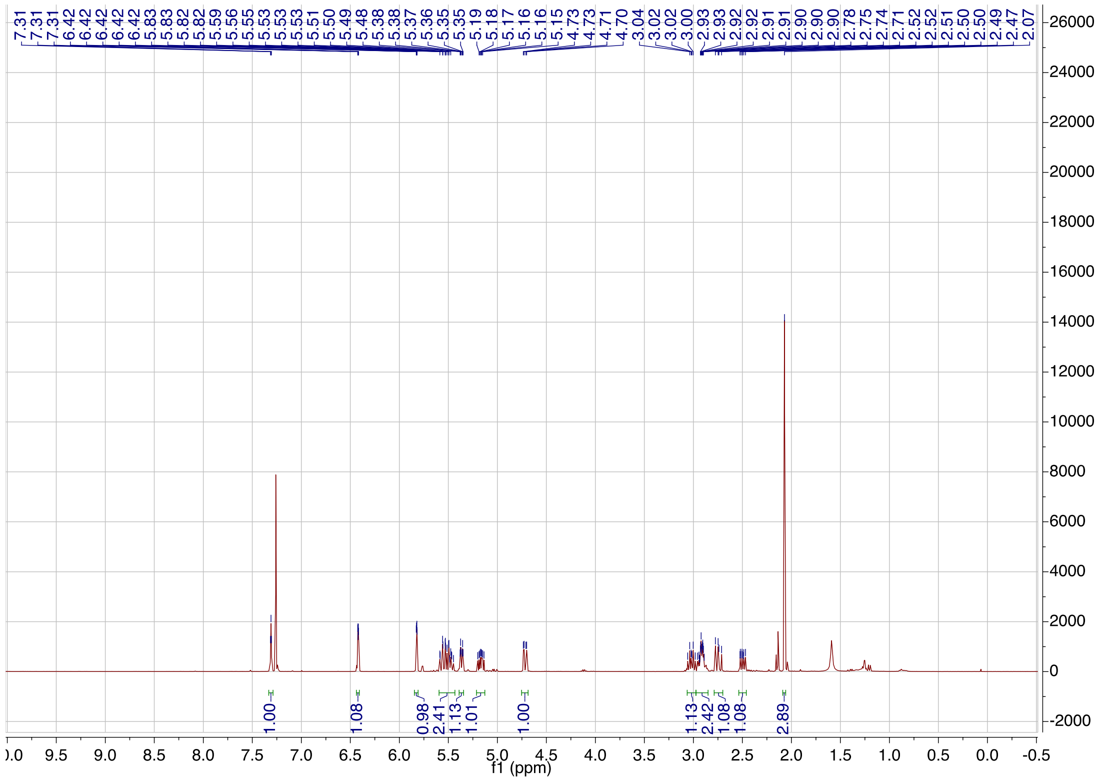

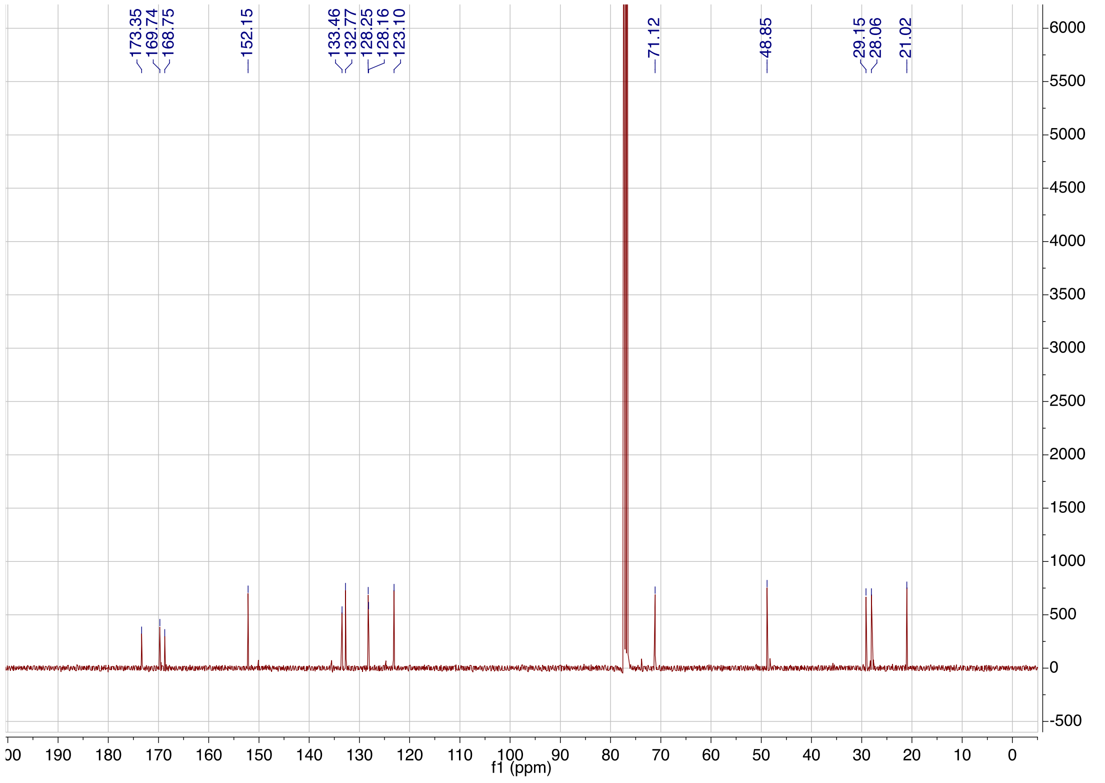

Supplementary figure 68: ^{1}H and ^{13}C NMR spectra of compound **19b**

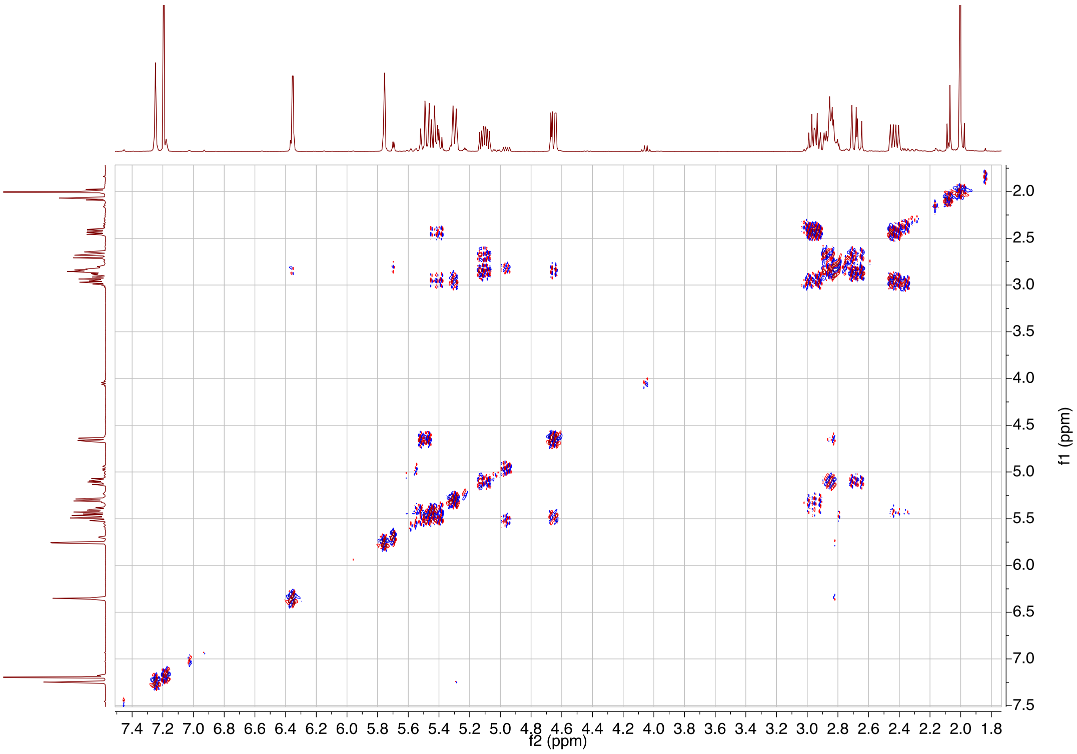

Supplementary figure 69: COSY spectrum of compound **19b**

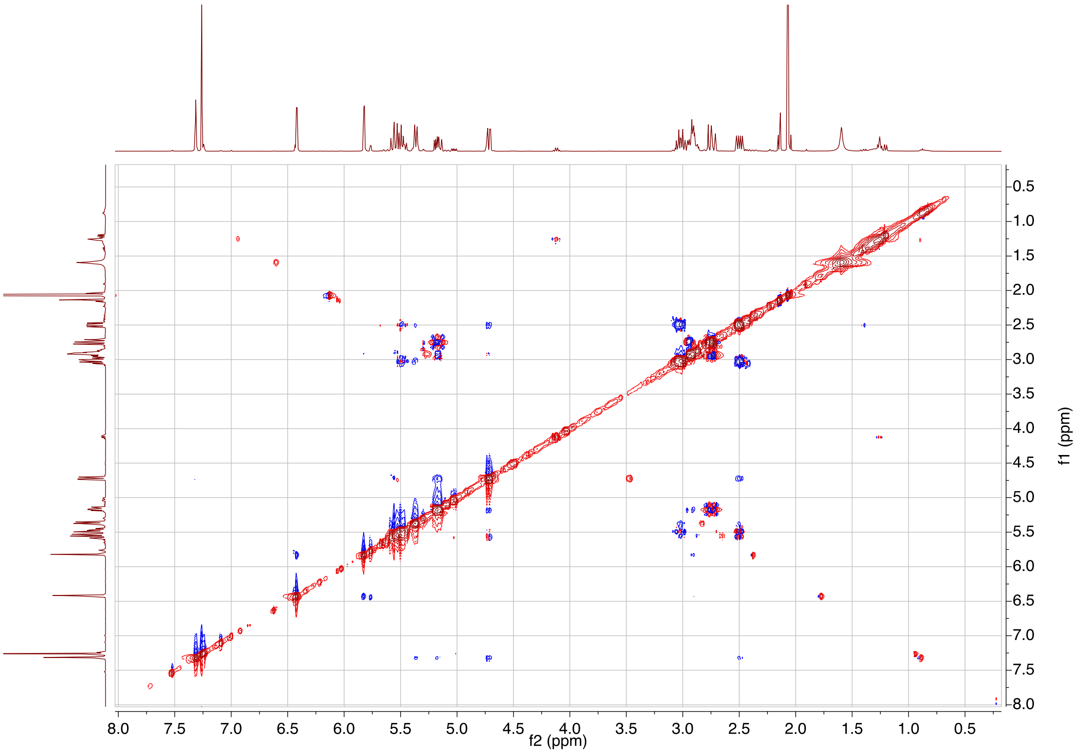

Supplementary figure 70: NOESY spectrum of compound **19b**

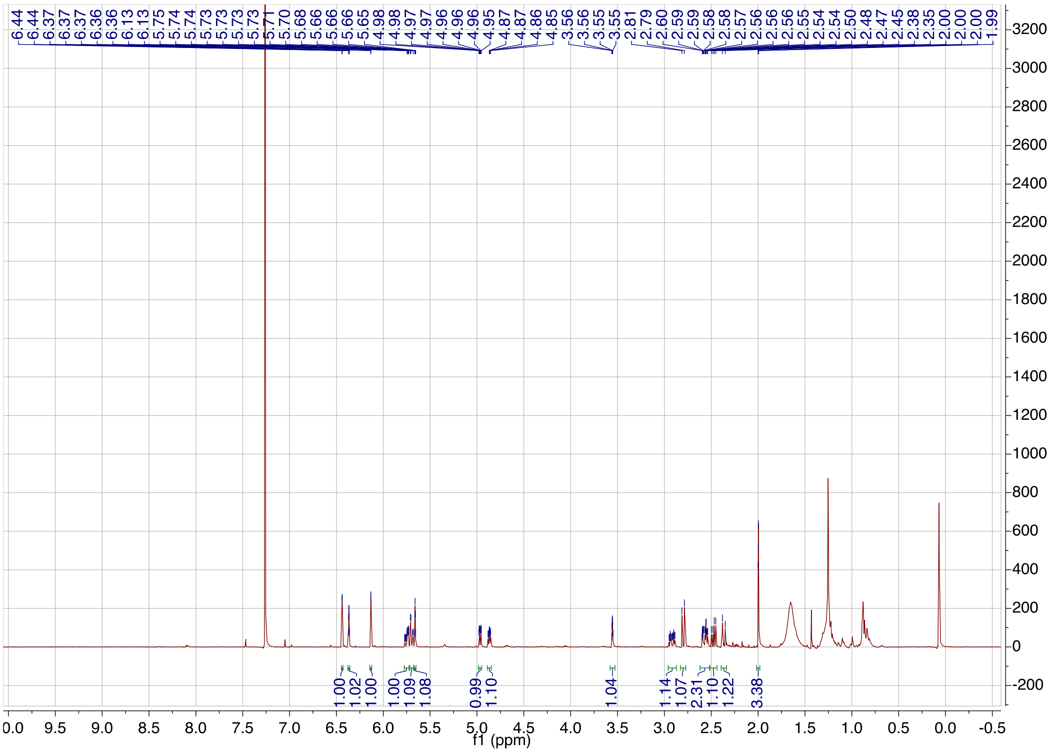

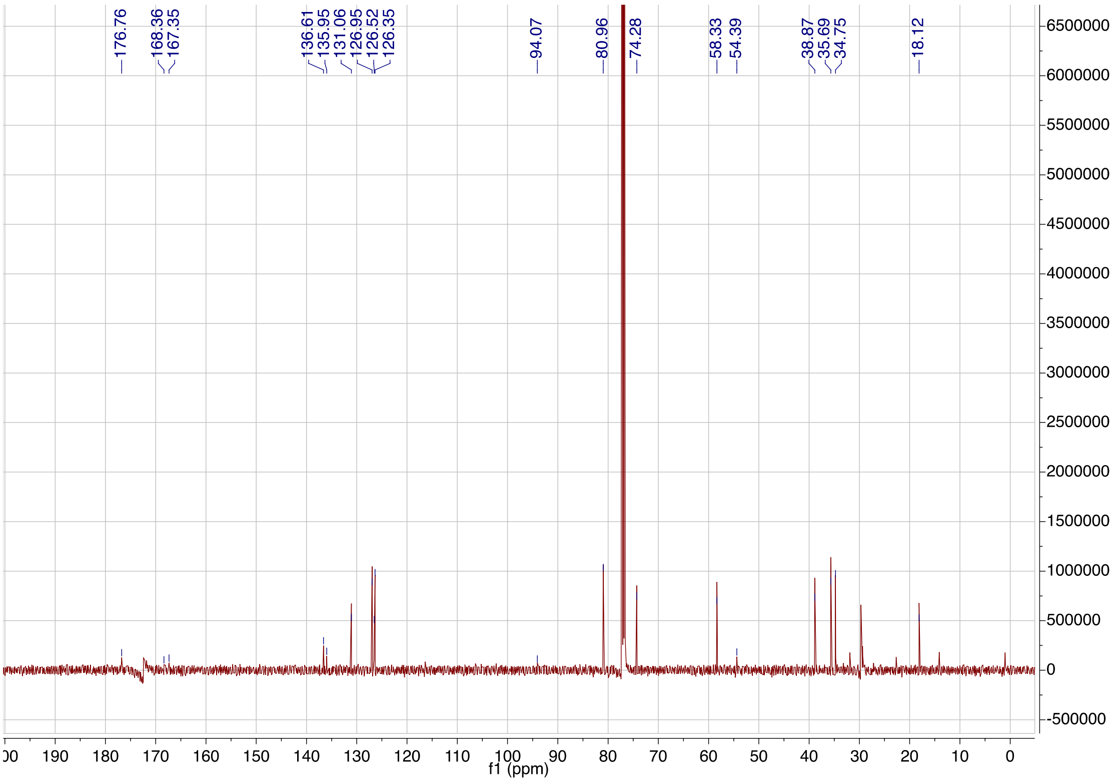

Supplementary figure 71: ^{1}H and ^{13}C NMR spectra of compound **20a**

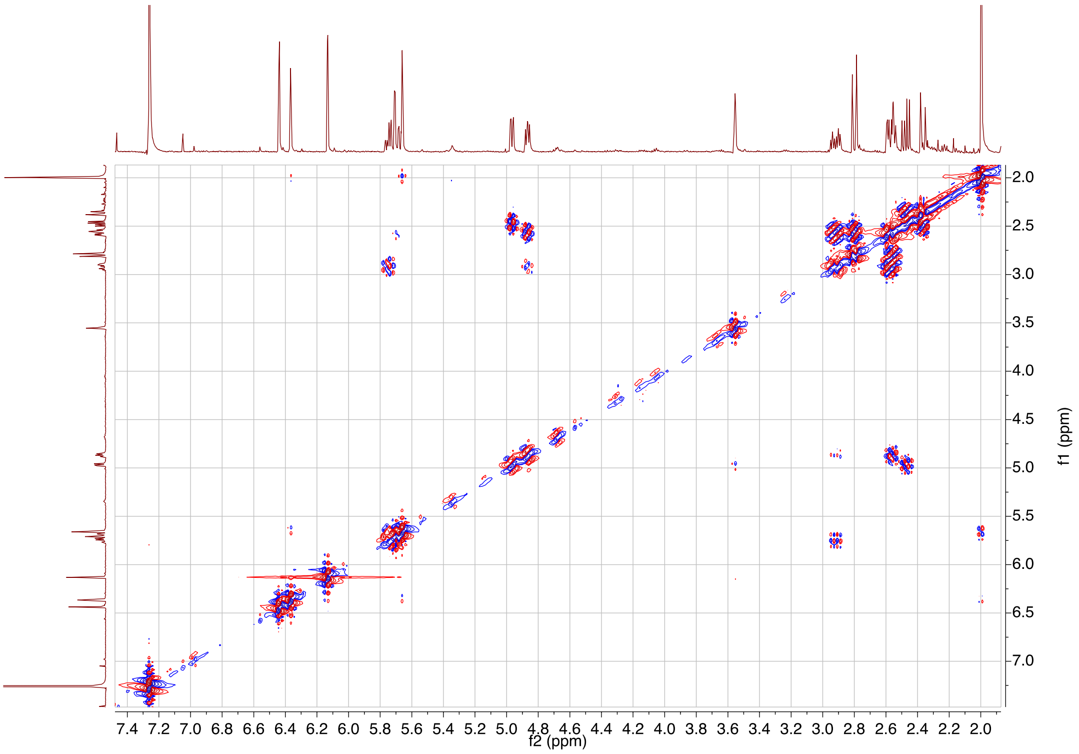

Supplementary figure 72: COSY spectrum of compound **20a**

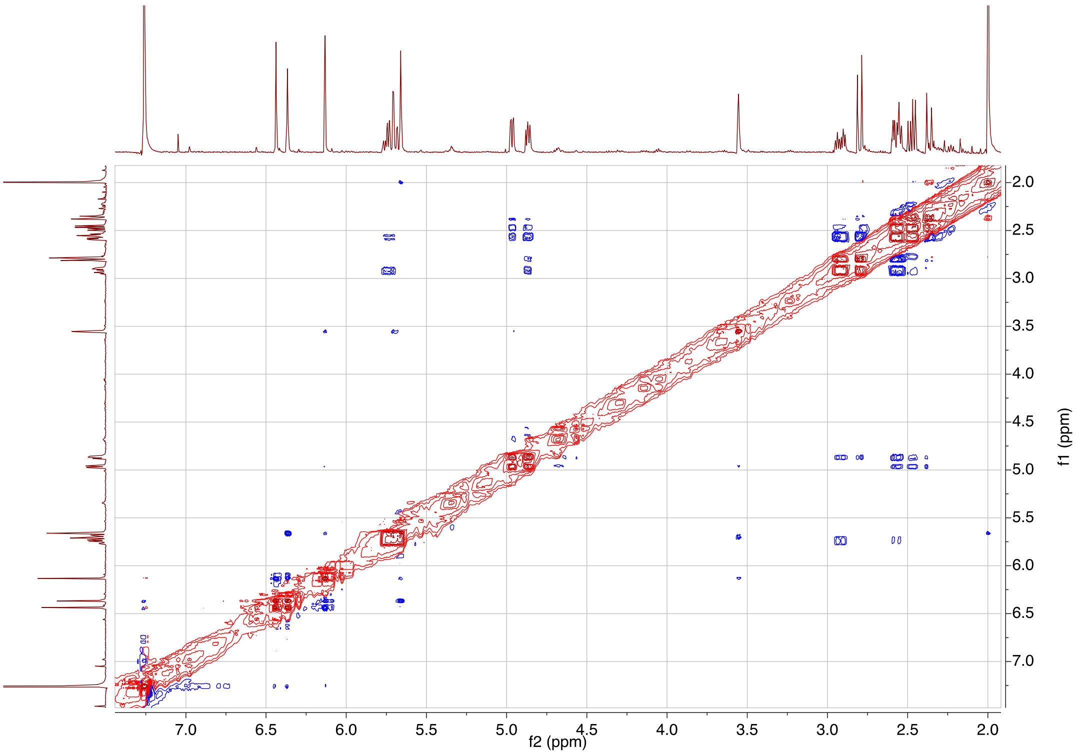

Supplementary figure 73: NOESY spectrum of compound **20a**

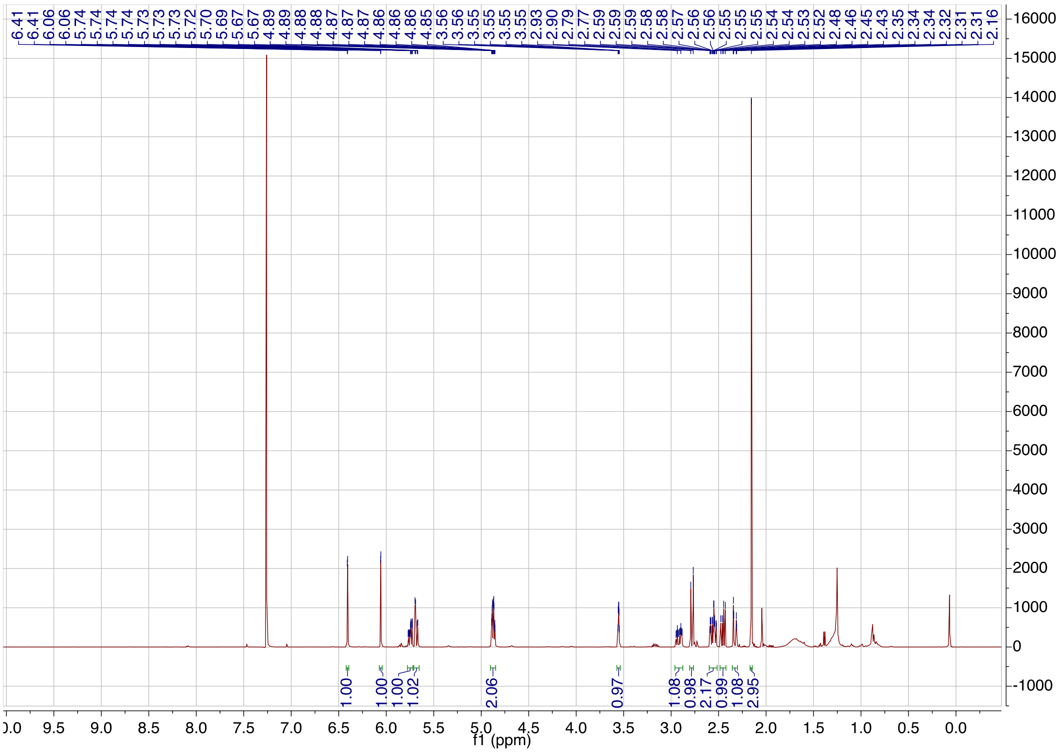

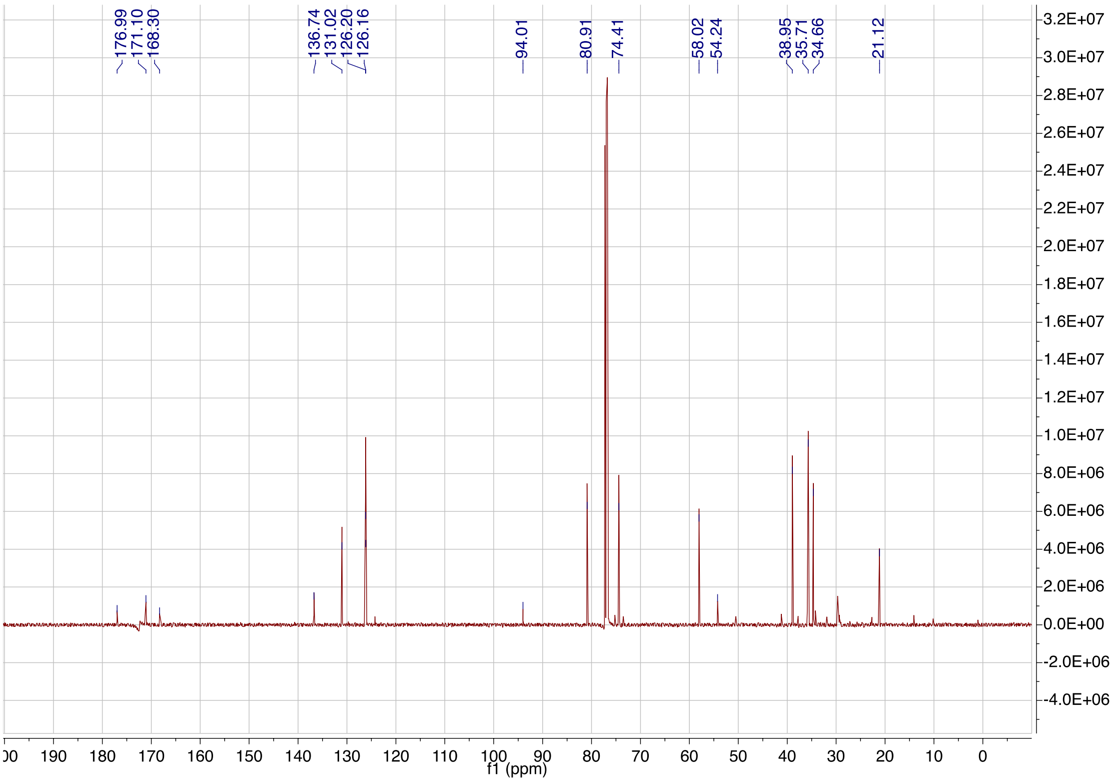

Supplementary figure 74: ^{1}H and ^{13}C NMR spectra of compound **20b**

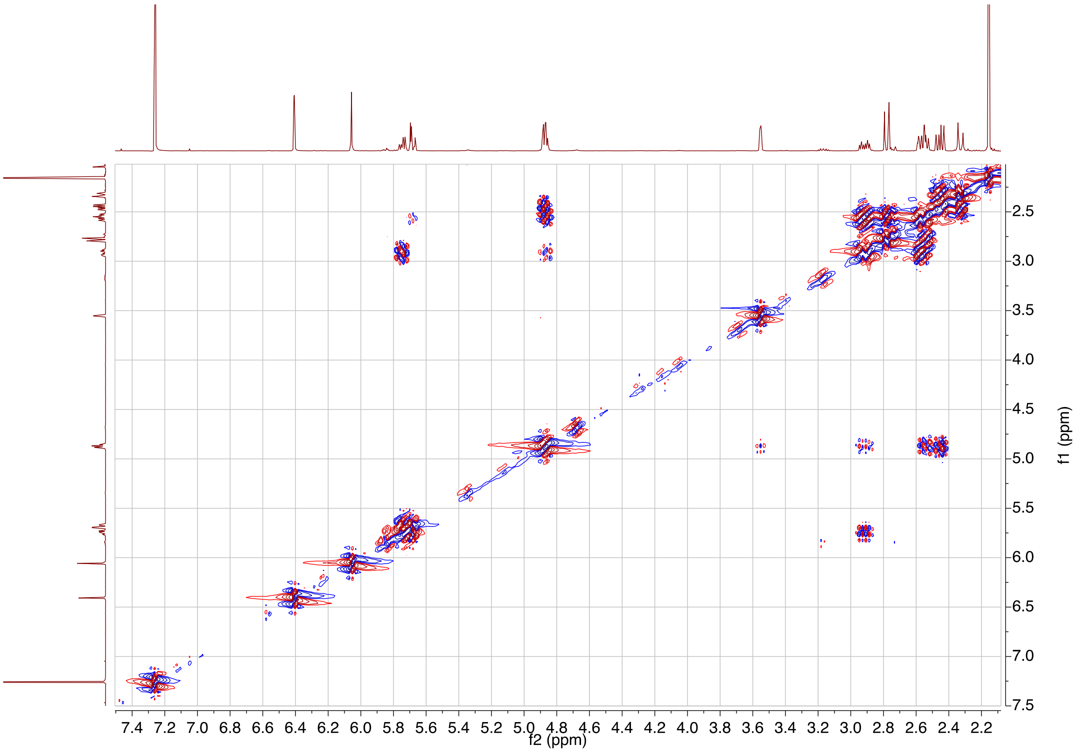

Supplementary figure 75: COSY spectrum of compound **20b**

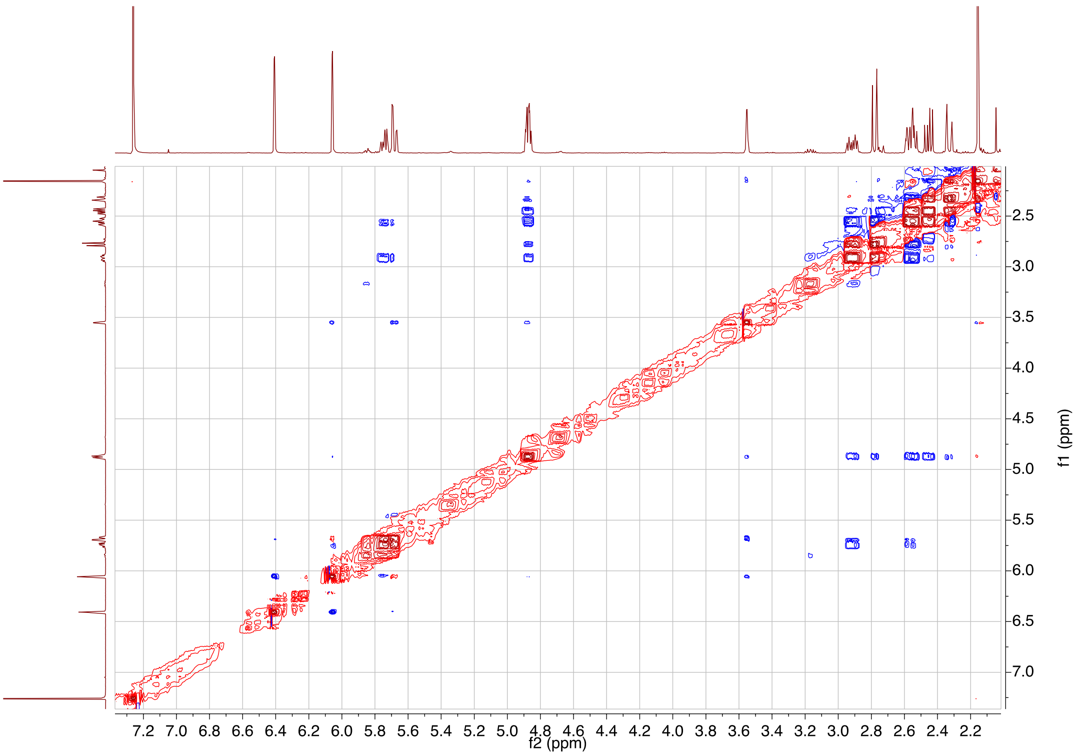

Supplementary figure 76: NOESY spectrum of compound **20b**

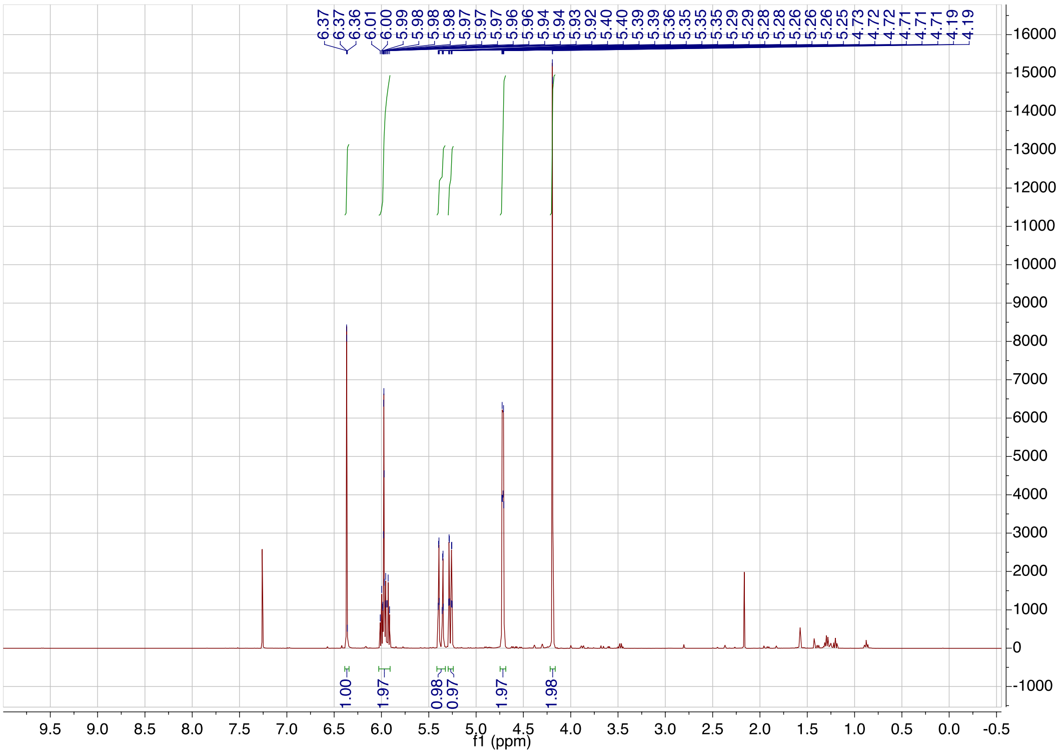

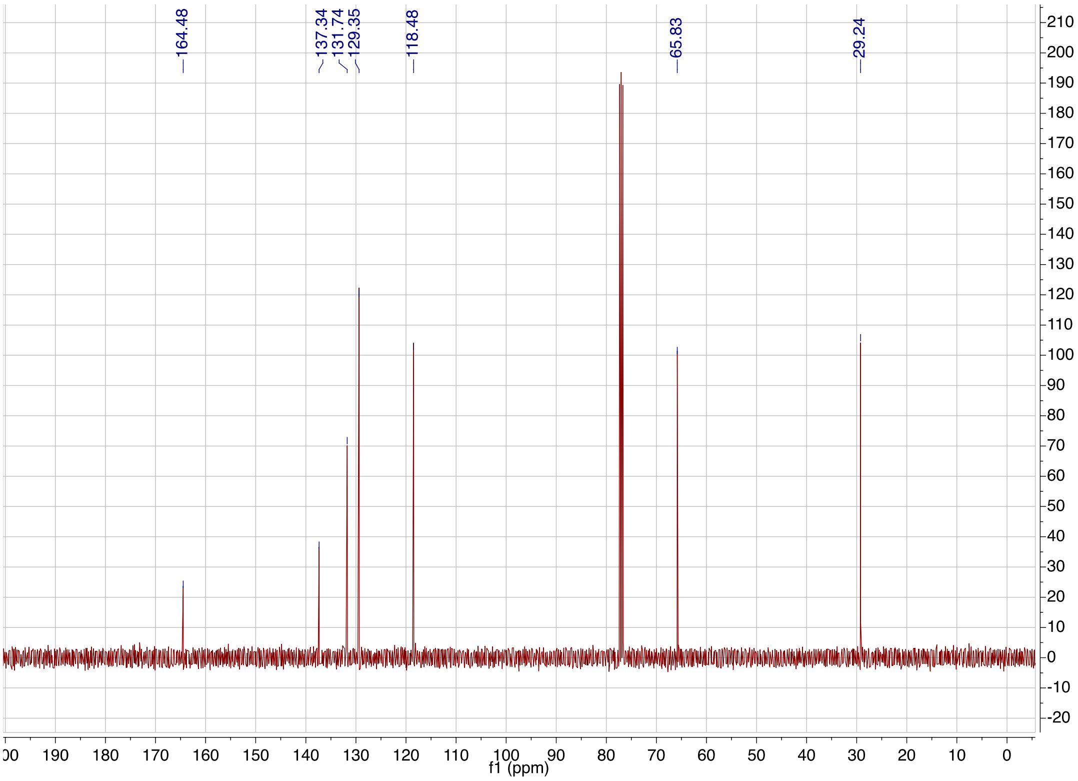

Supplementary figure 77: ^{1}H and ^{13}C NMR spectra of compound **S-21a**

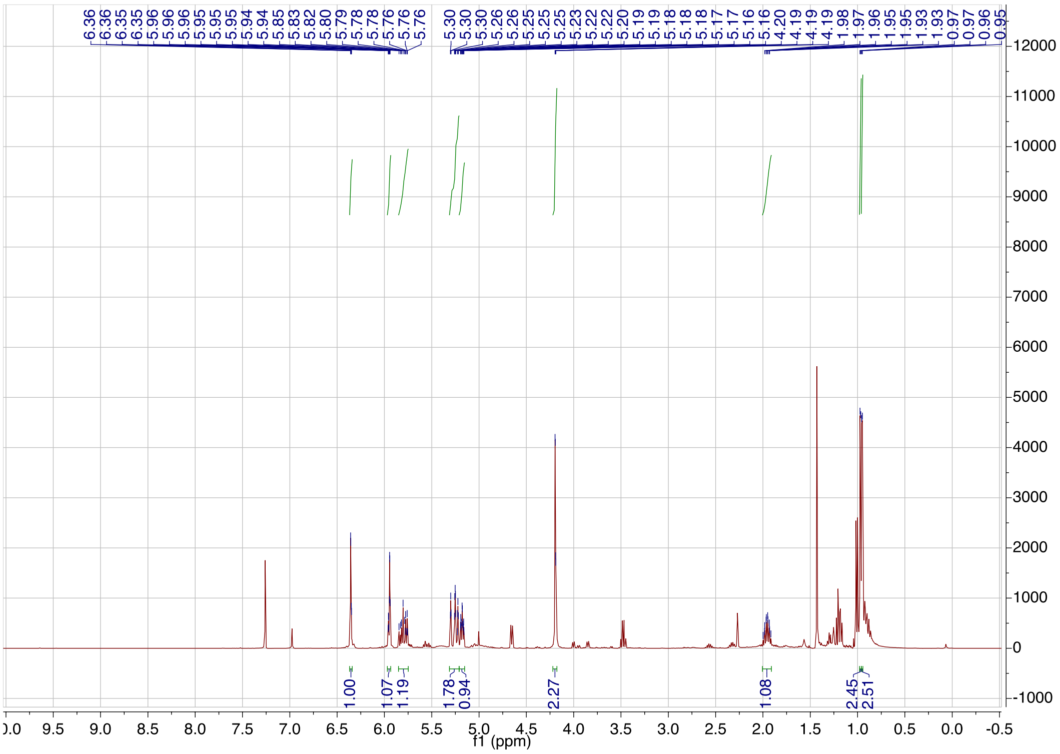

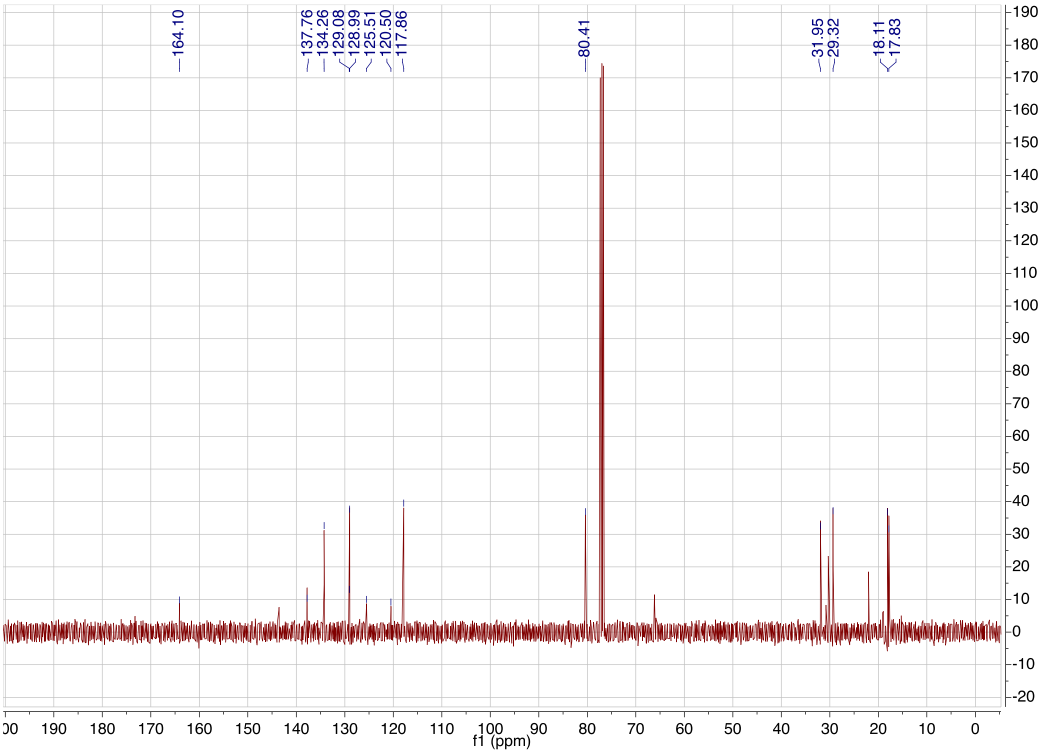

Supplementary figure 78: ^{1}H and ^{13}C NMR spectra of compound **S-21b**

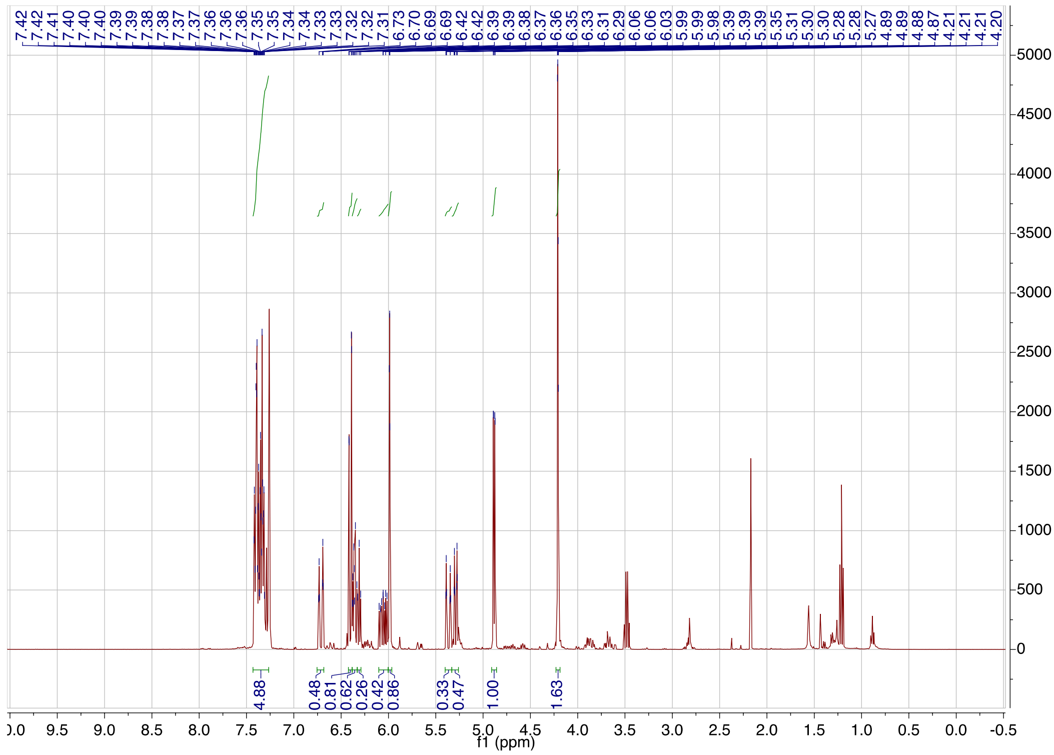

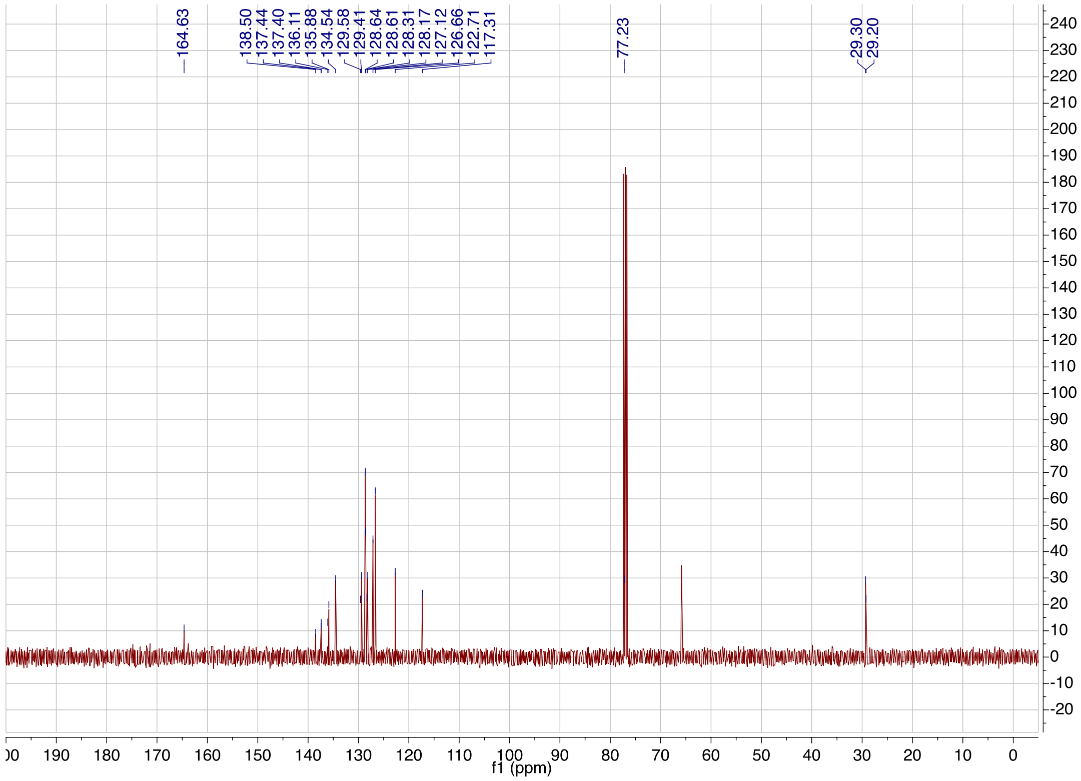

Supplementary figure 79: ^{1}H and ^{13}C NMR spectra of compound **S-21c and regioisomer S-21c'**

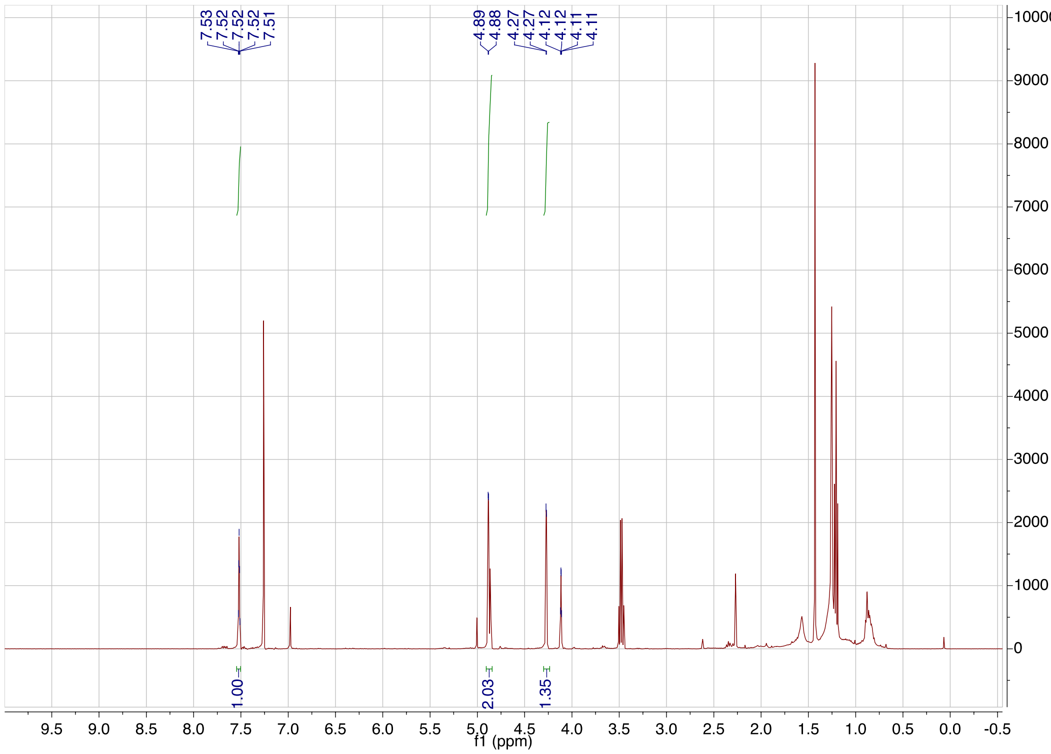

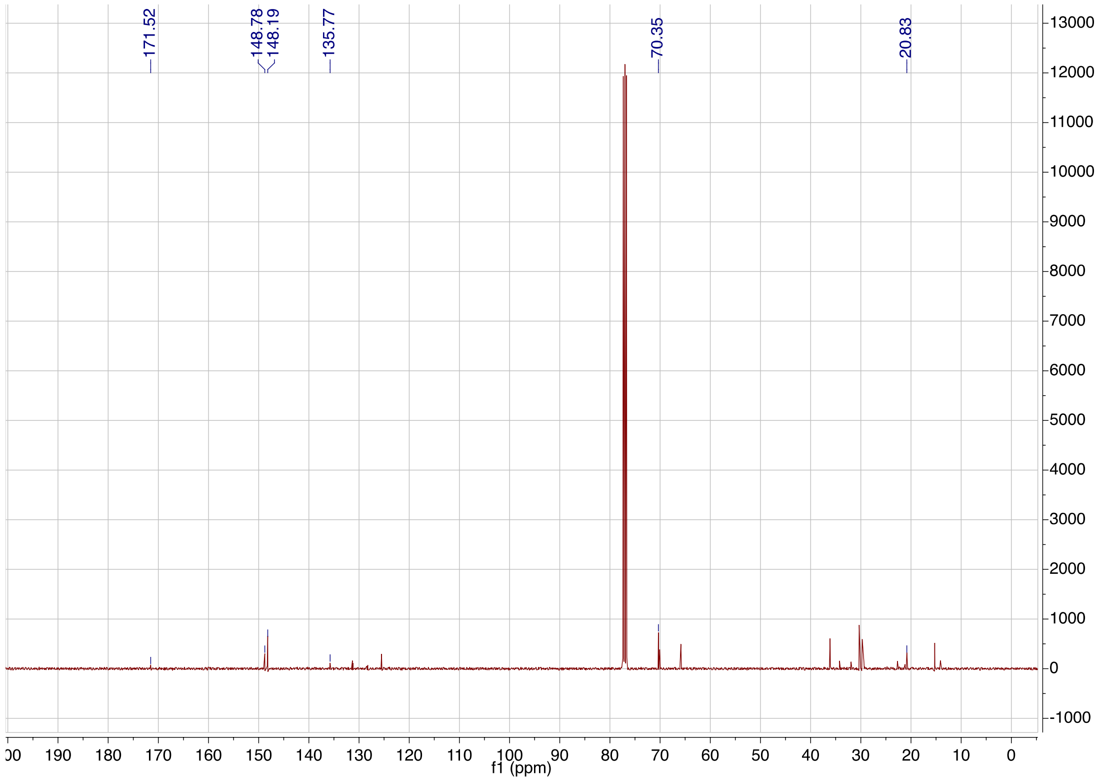

Supplementary figure 80: ^{1}H and ^{13}C NMR spectra of compound **22a**

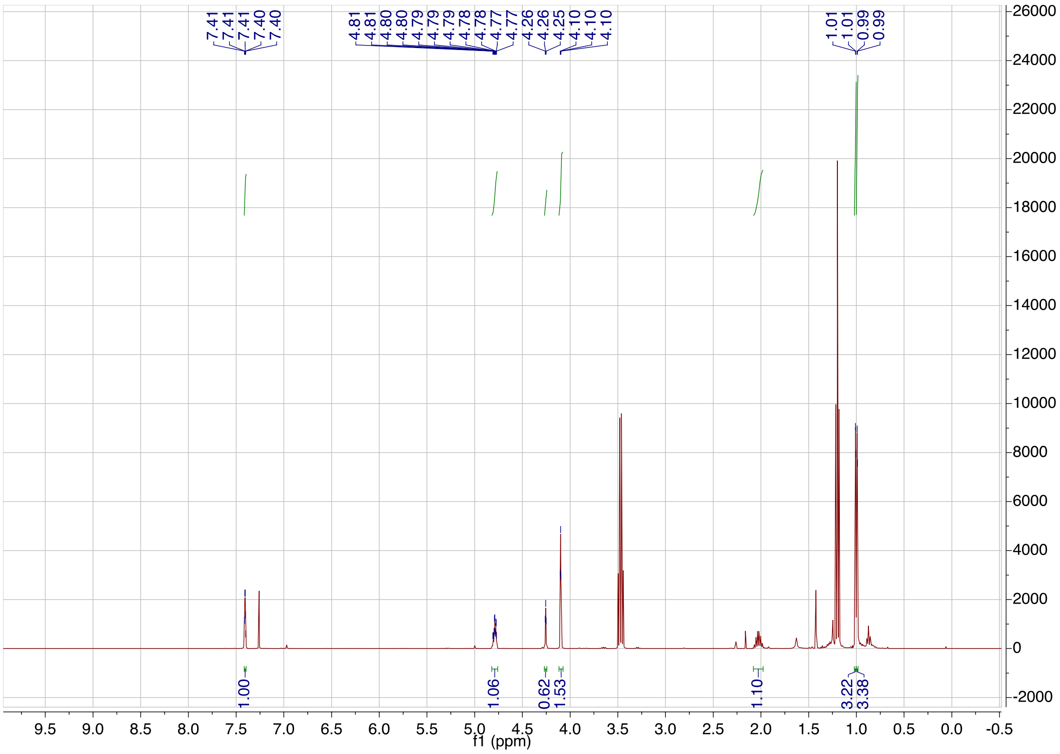

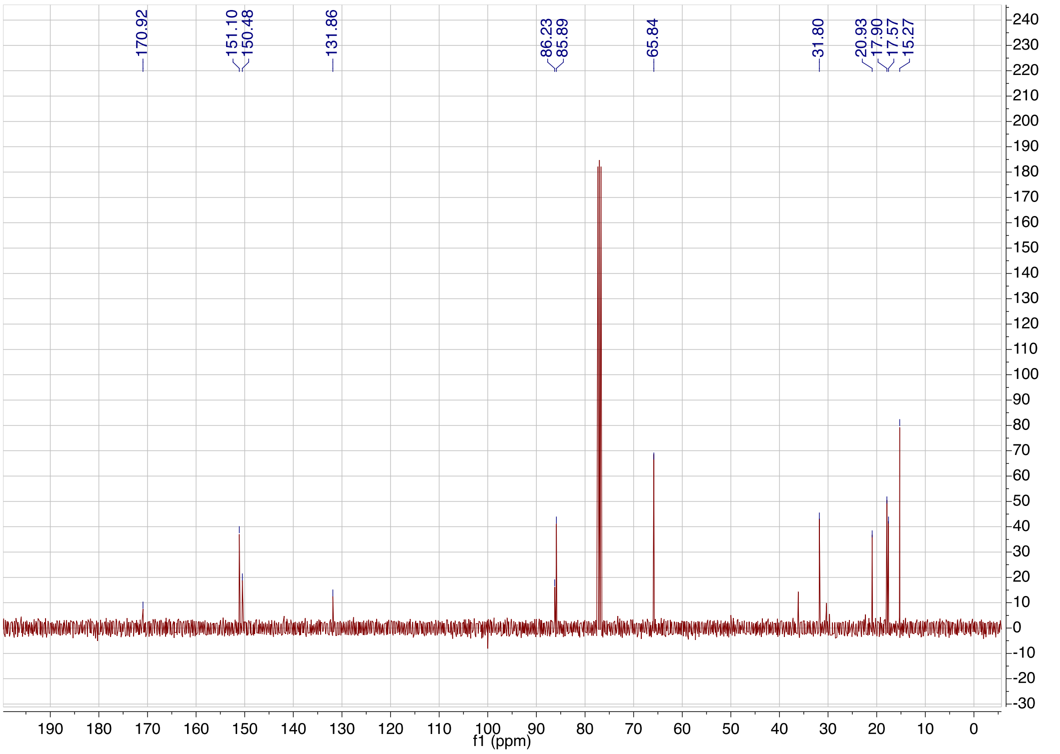

Supplementary figure 81: ^{1}H and ^{13}C NMR spectra of compound **22b**

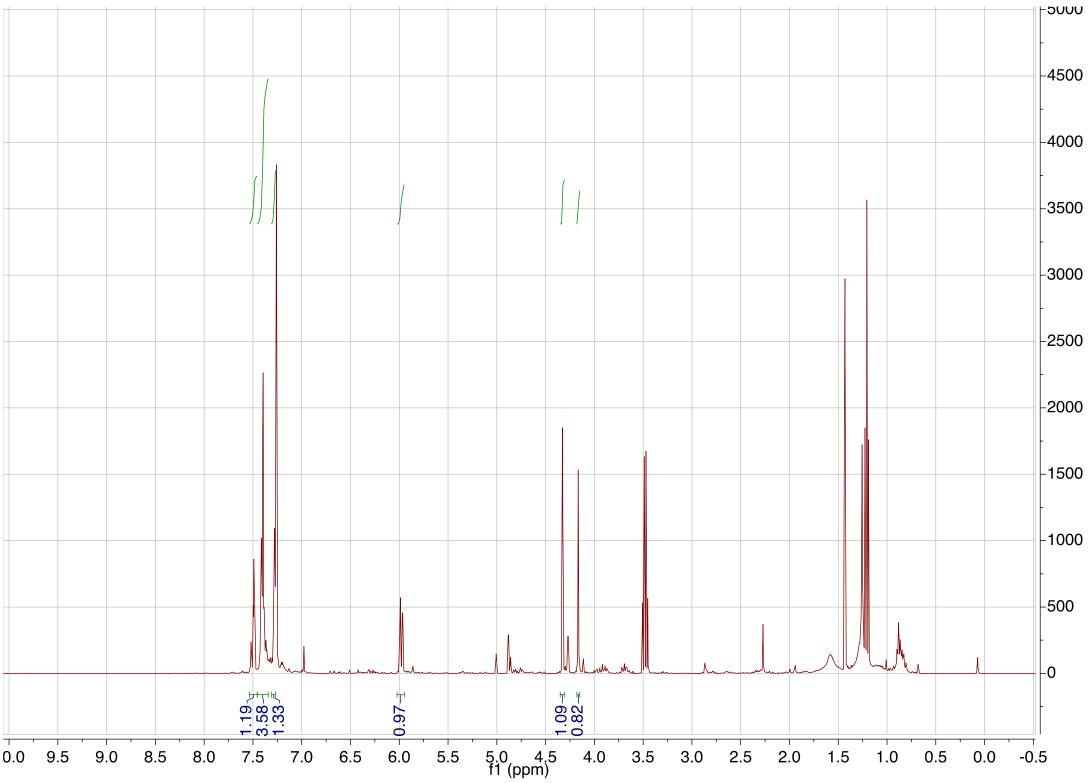

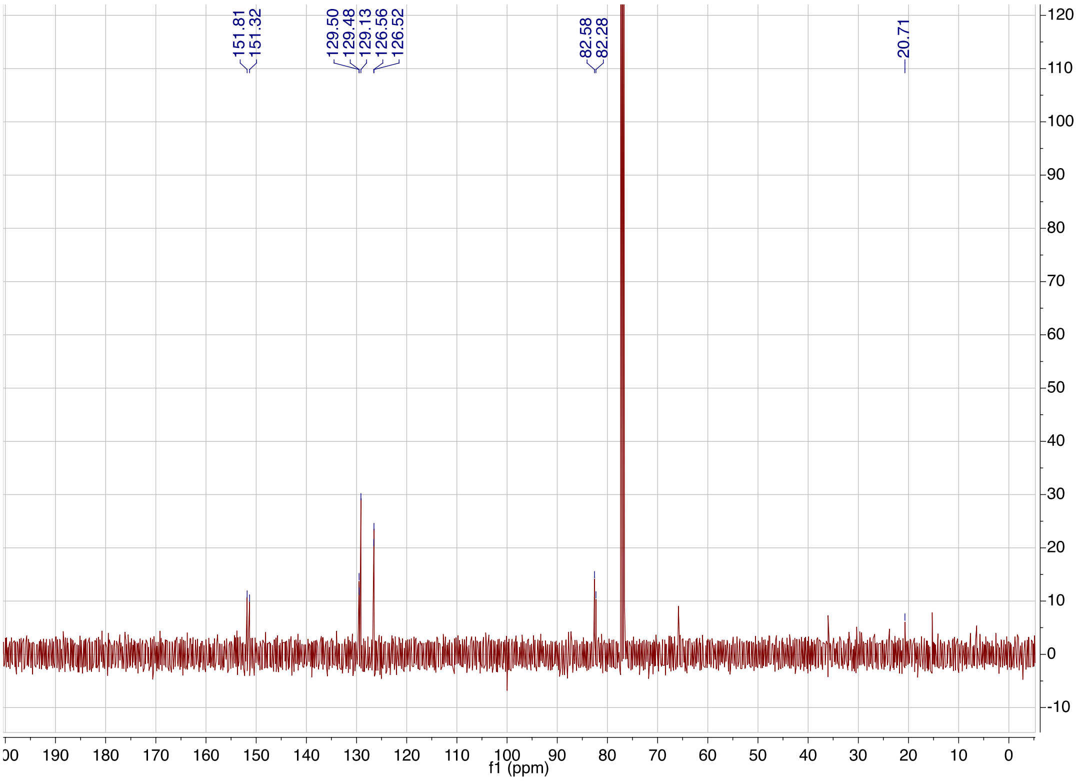

Supplementary figure 82: ^{1}H and ^{13}C NMR spectra of compound **22c**

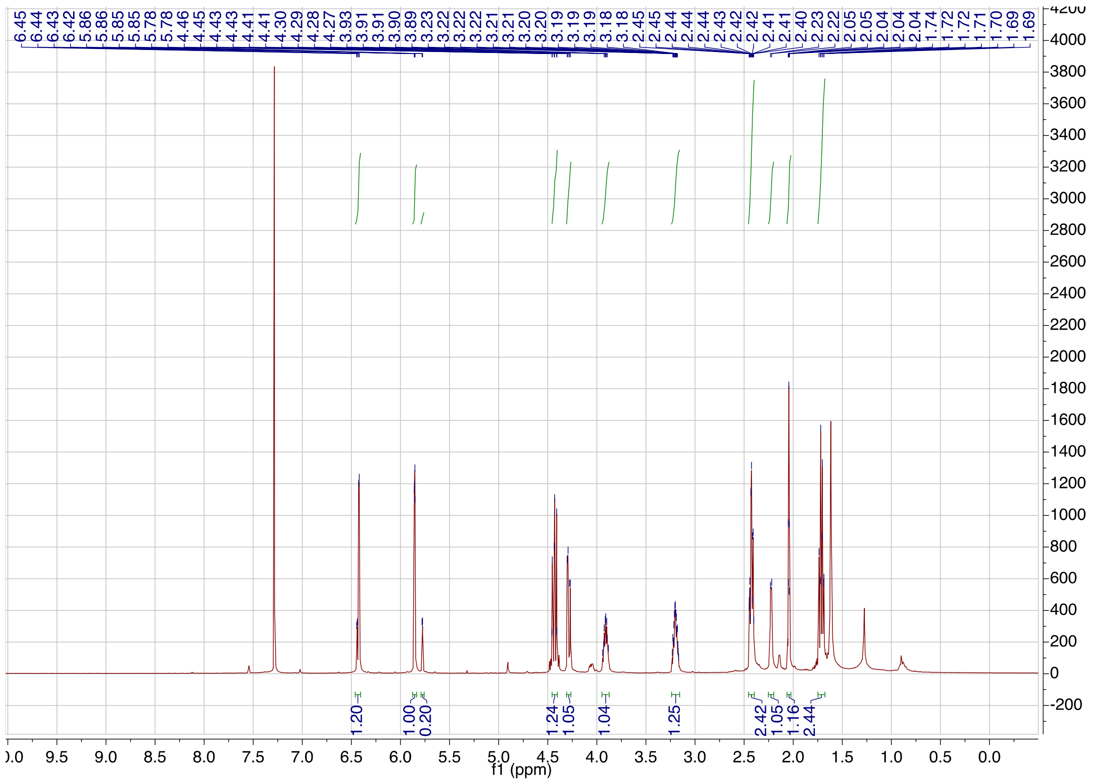

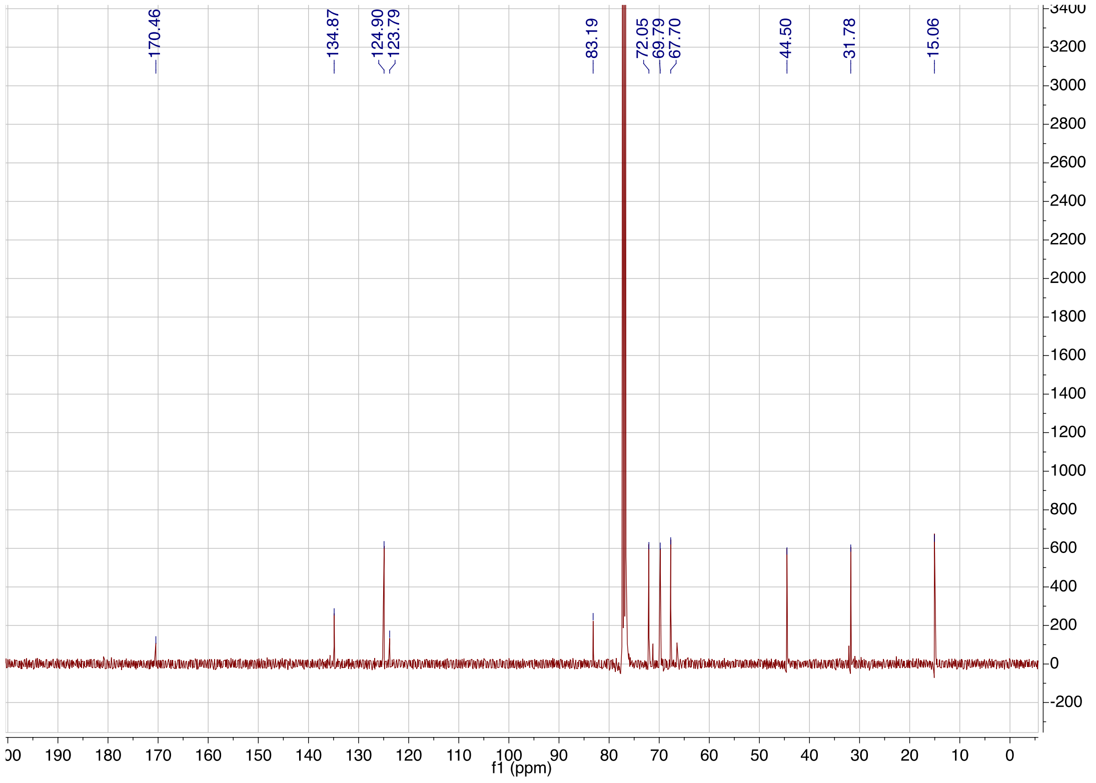

Supplementary figure 83: ^{1}H and ^{13}C NMR spectra of compound **23a**

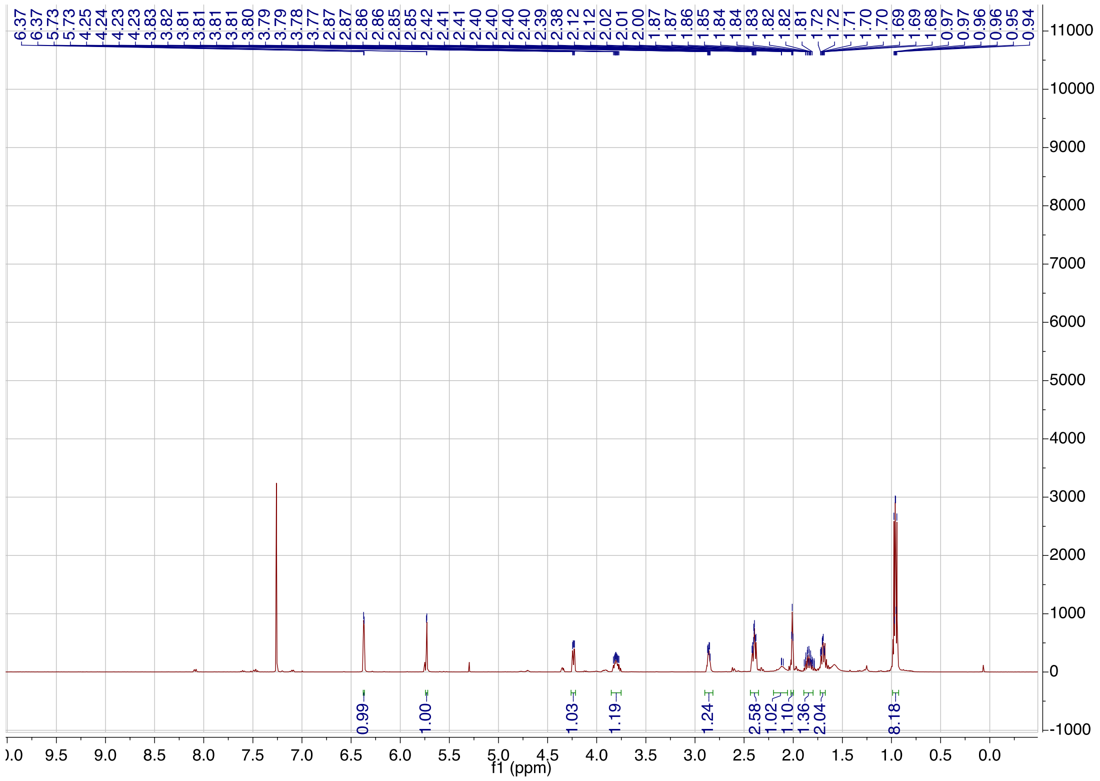

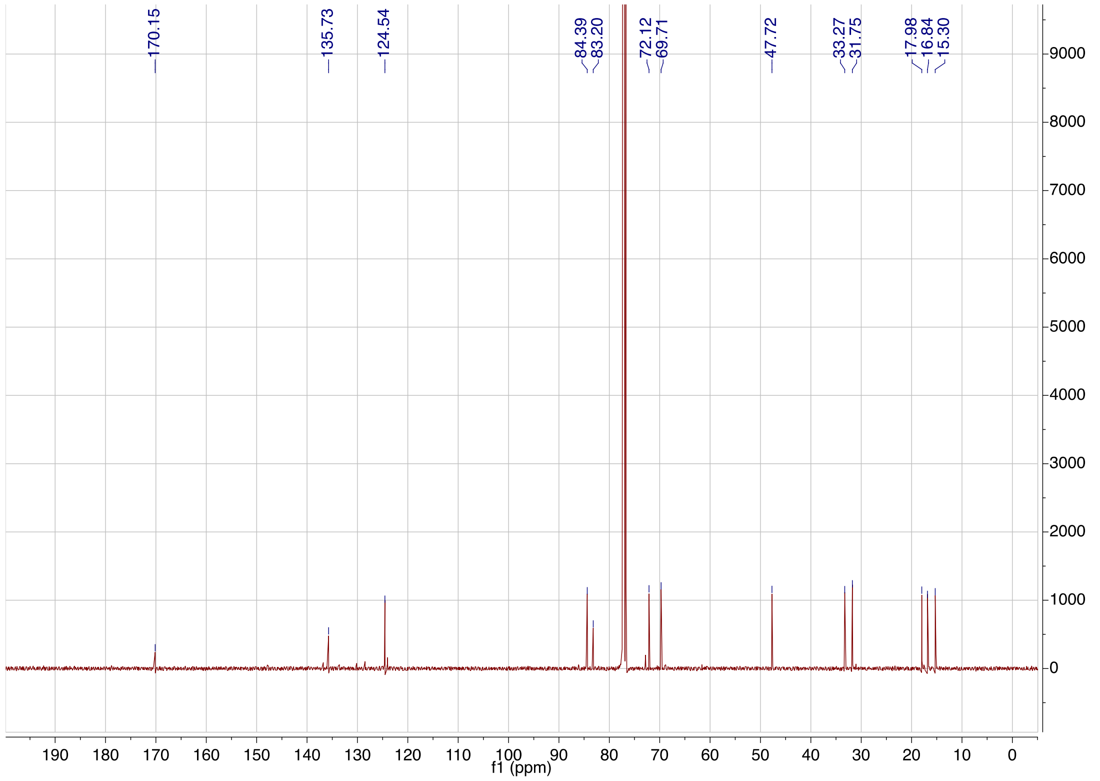

Supplementary figure 84: ^{1}H and ^{13}C NMR spectra of compound **23b**

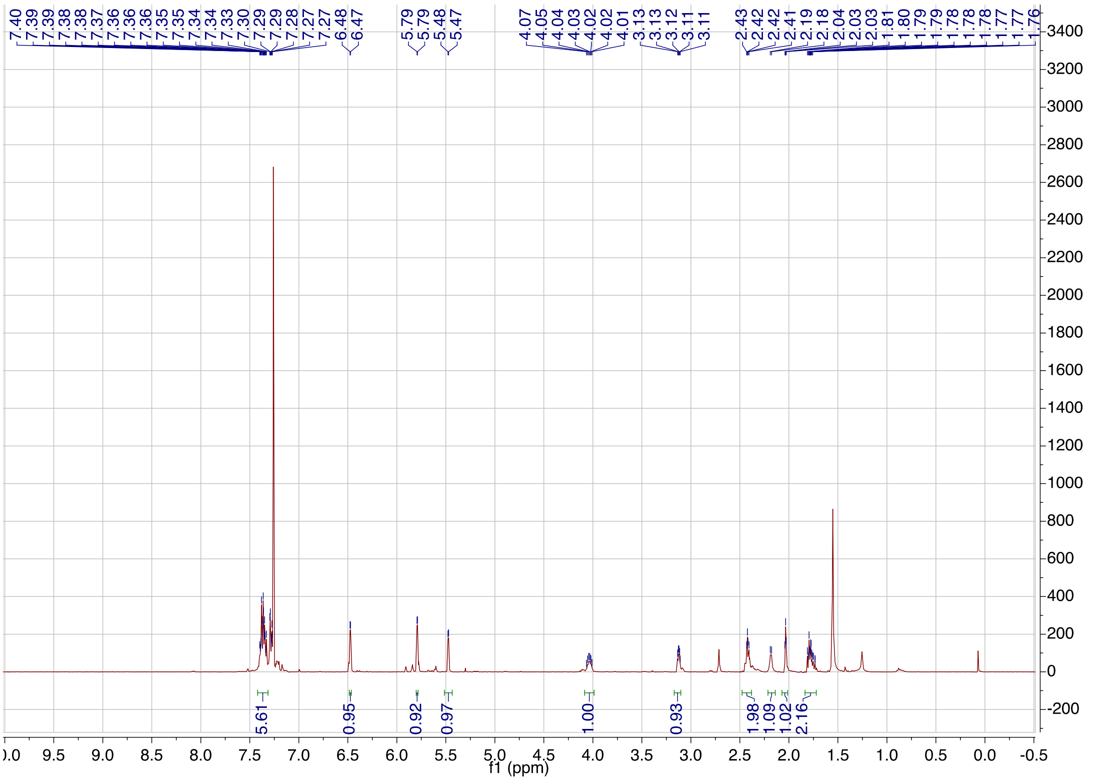

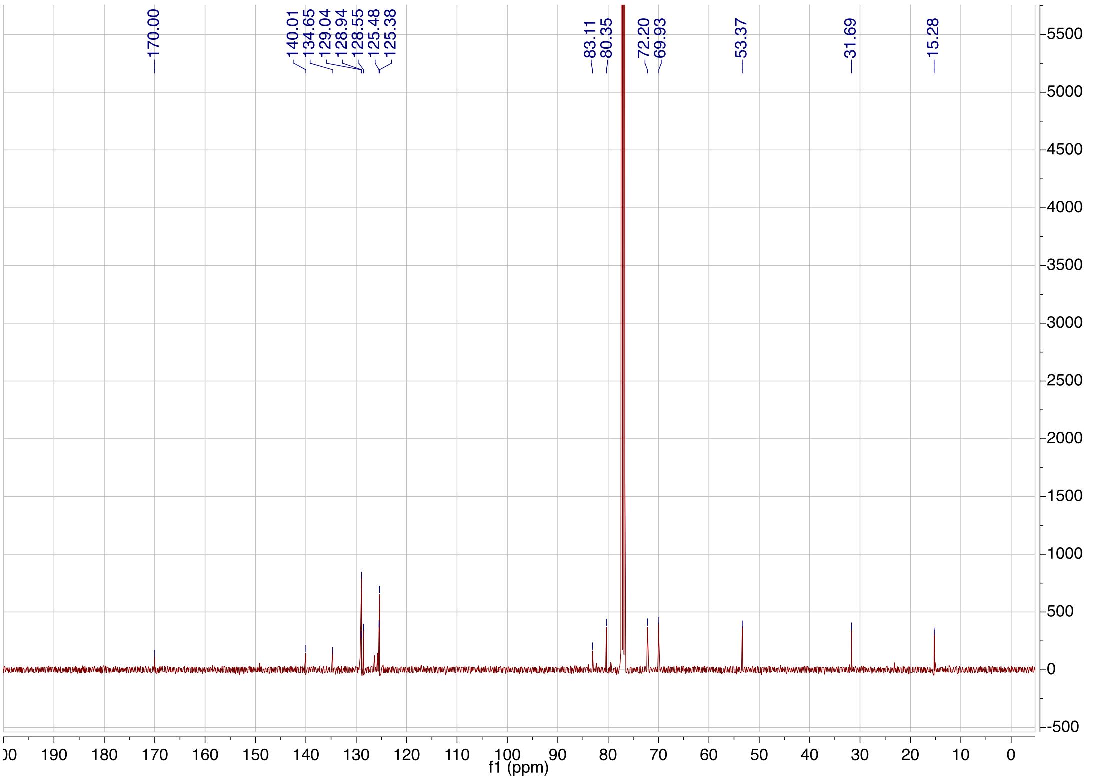

Supplementary figure 85: ^{1}H and ^{13}C NMR spectra of compound **23c**

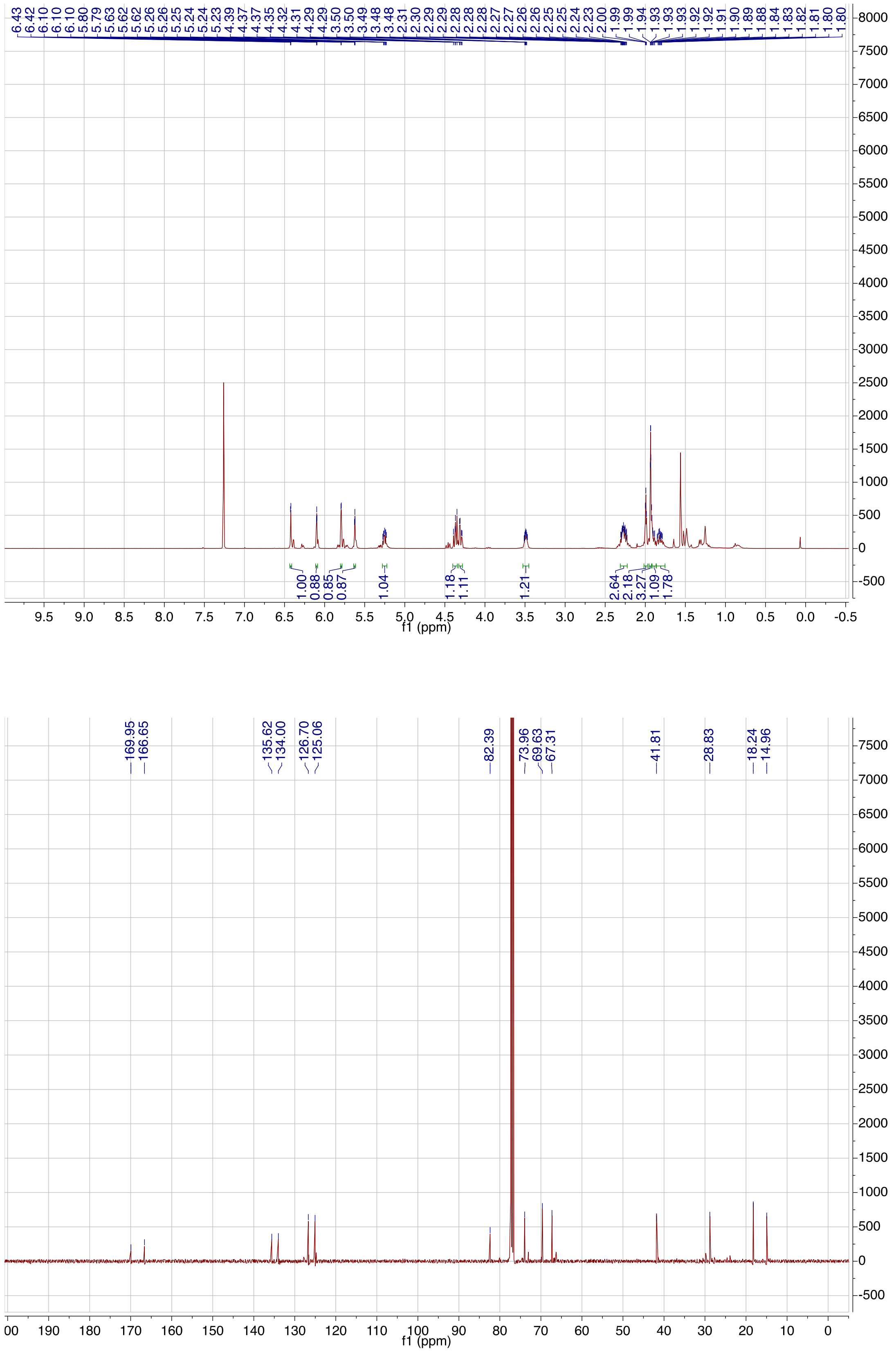

Supplementary figure 86: ^{1}H and ^{13}C NMR spectra of compound **24a**

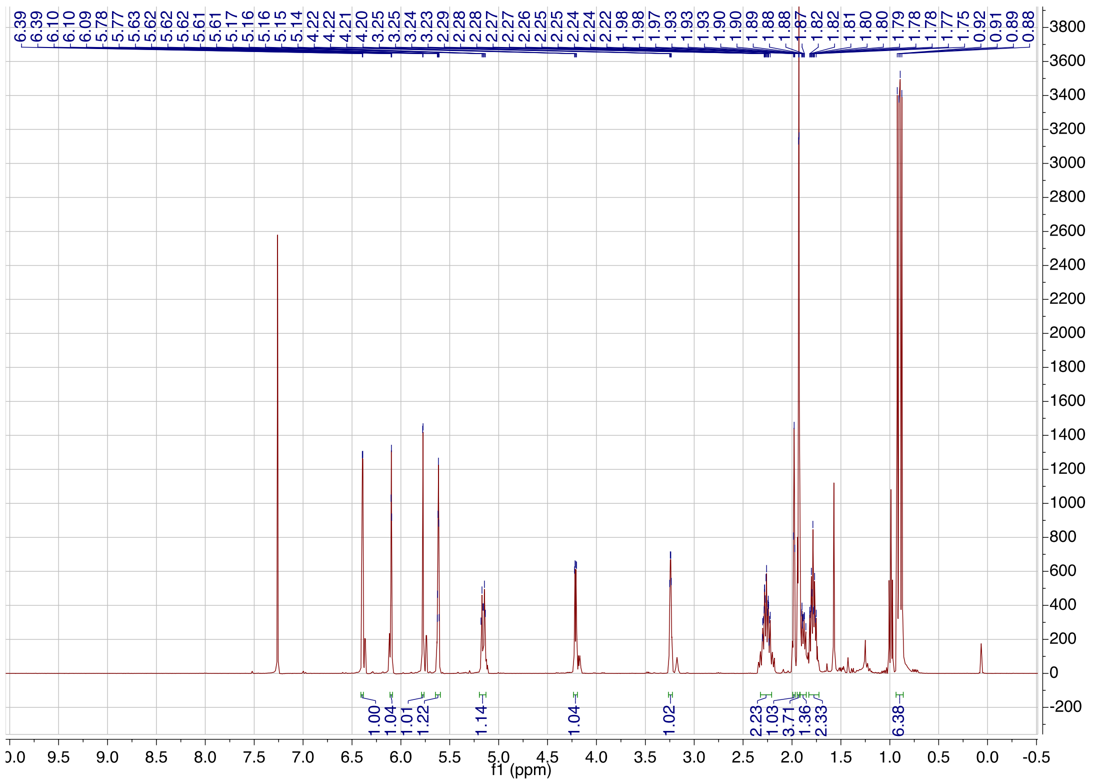

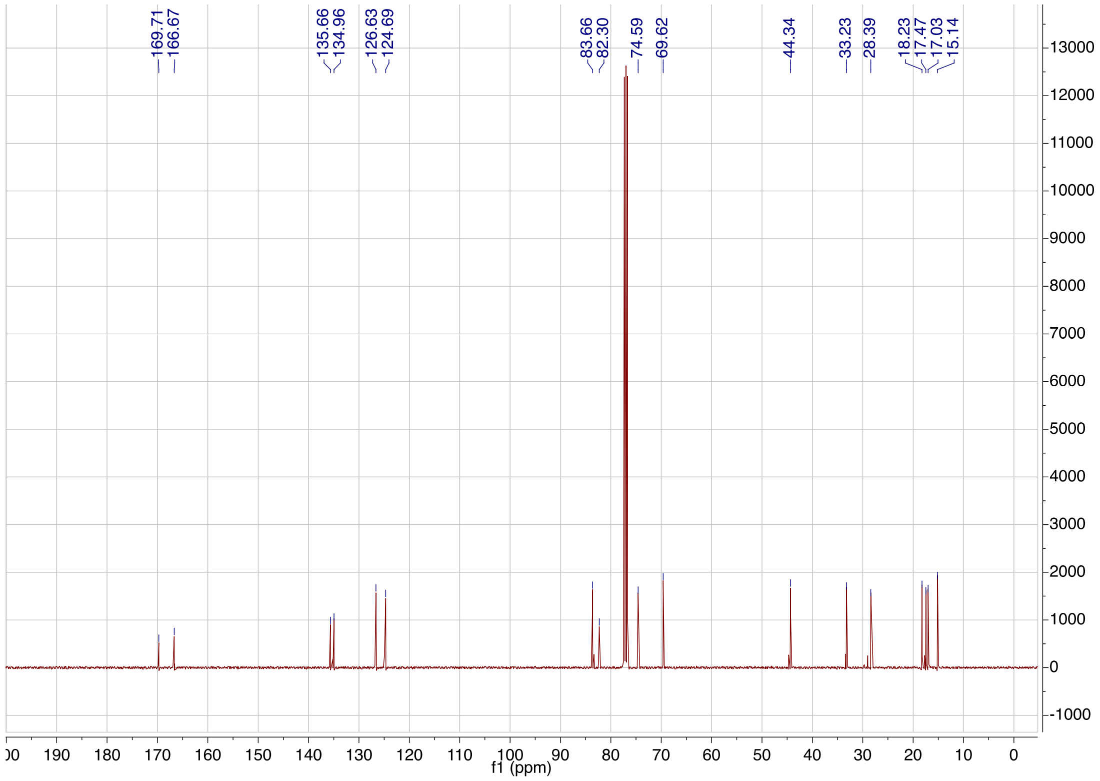

Supplementary figure 87: ^{1}H and ^{13}C NMR spectra of compound **24b**

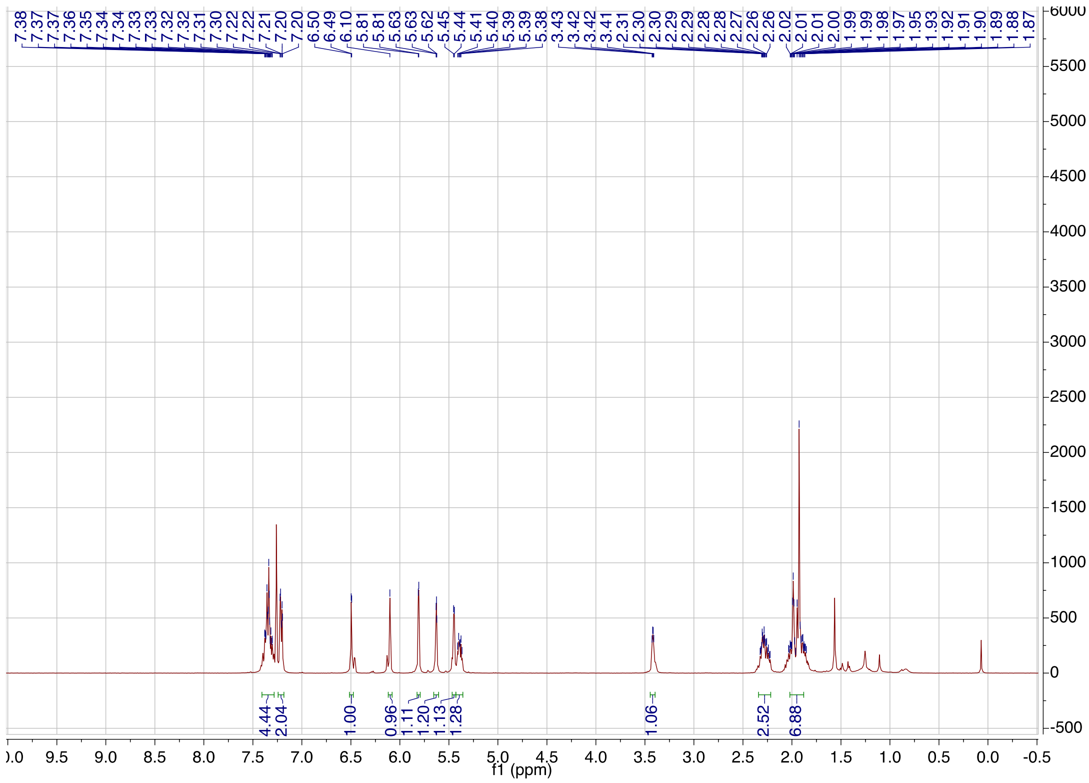

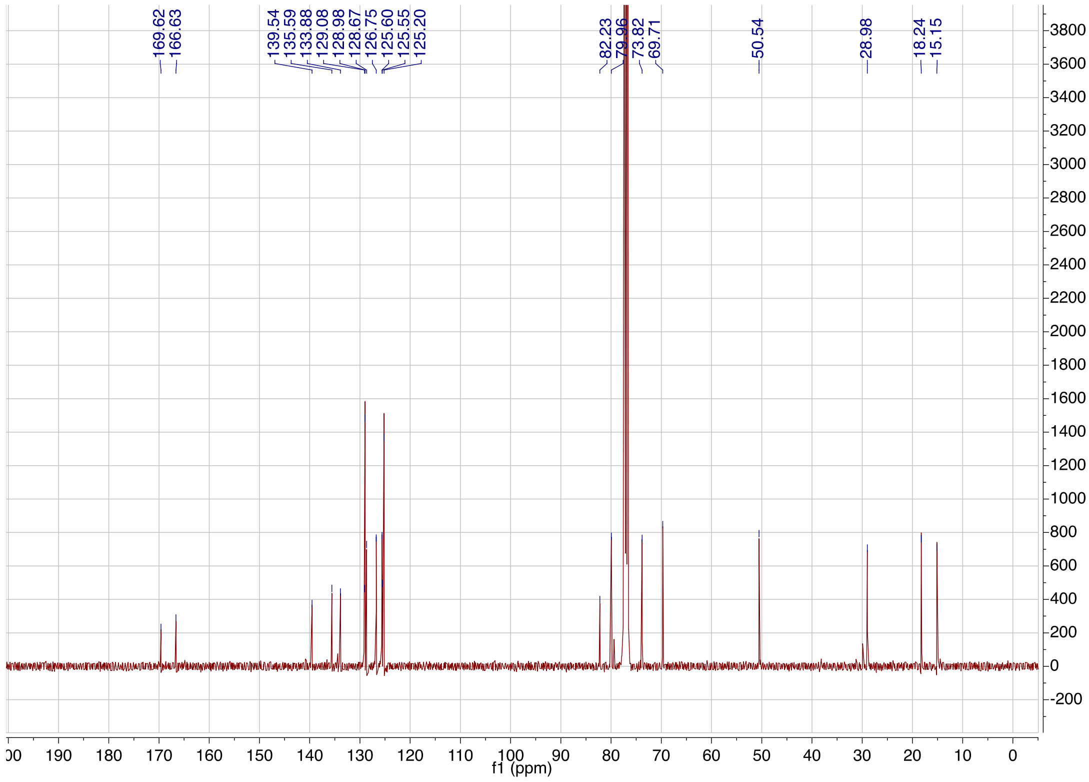

Supplementary figure 88: ^{1}H and ^{13}C NMR spectra of compound **24c**

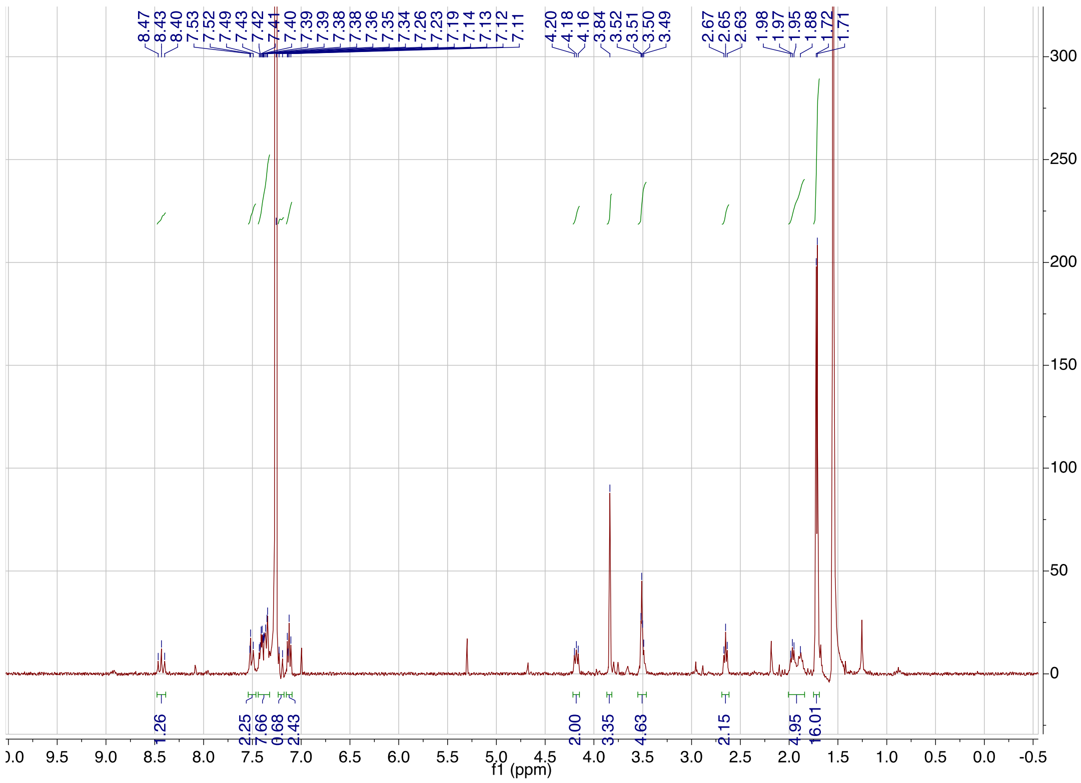

Supplementary figure 89: ^{1}H spectrum of compound **S-23**

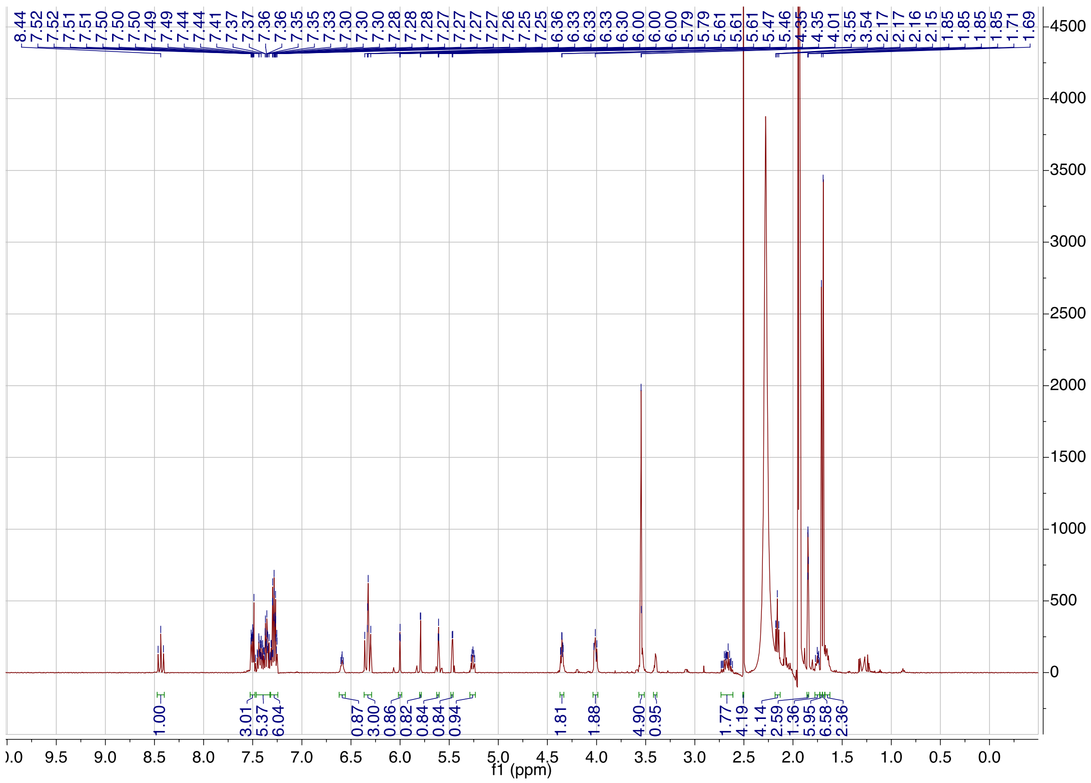

Supplementary figure 90: ^{1}H spectrum of compound **24c-Cy3**

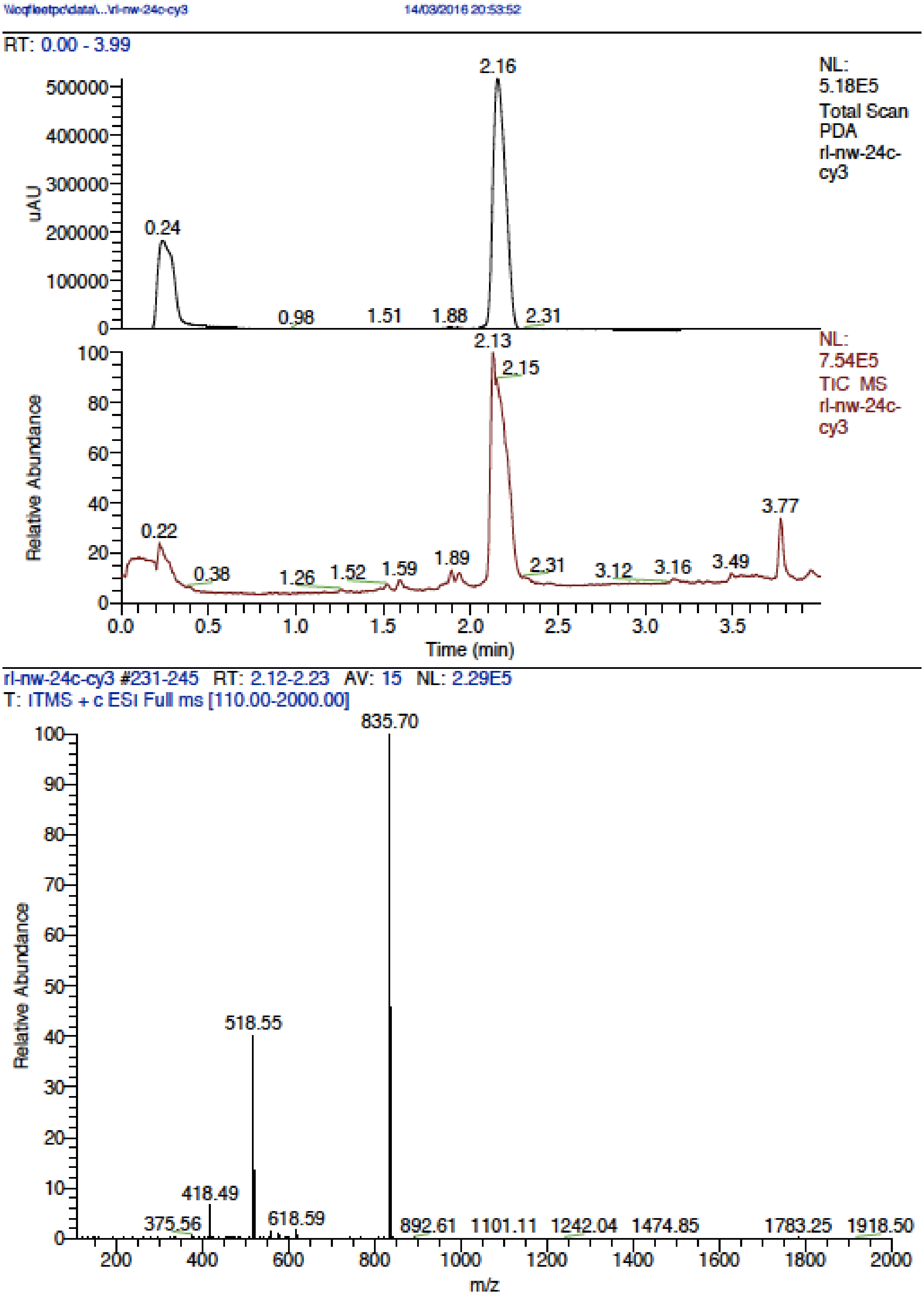

Supplementary figure 91: LC-MS trace of compound **24c-Cy3**

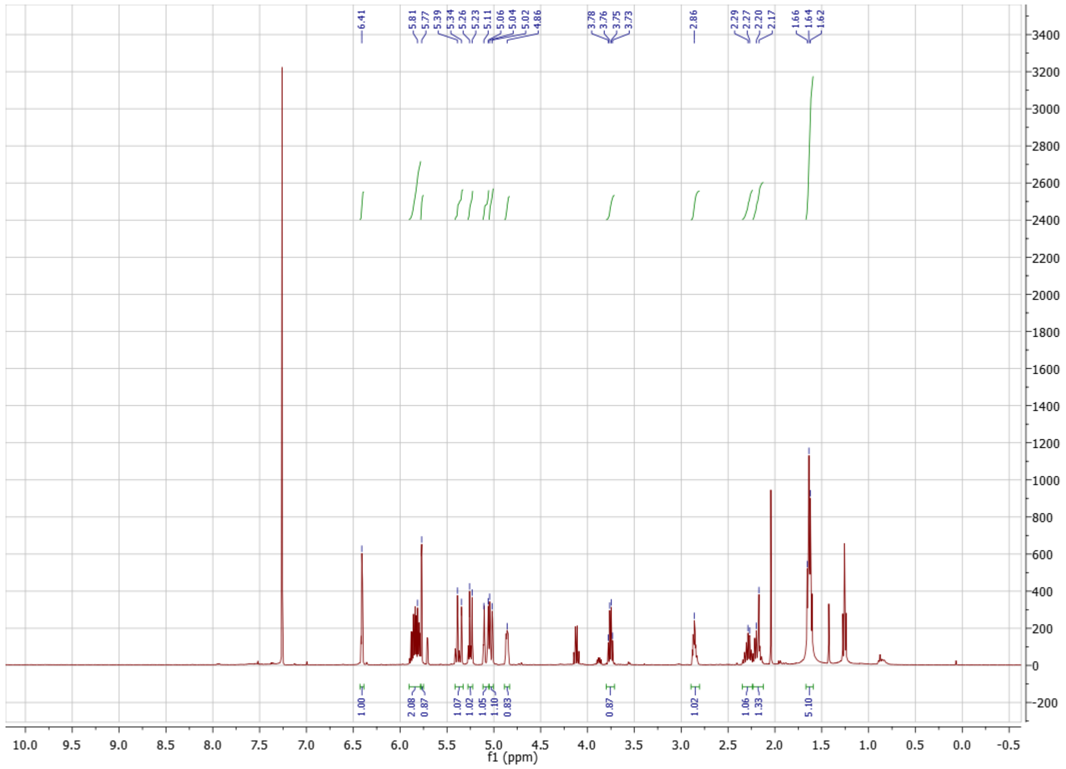

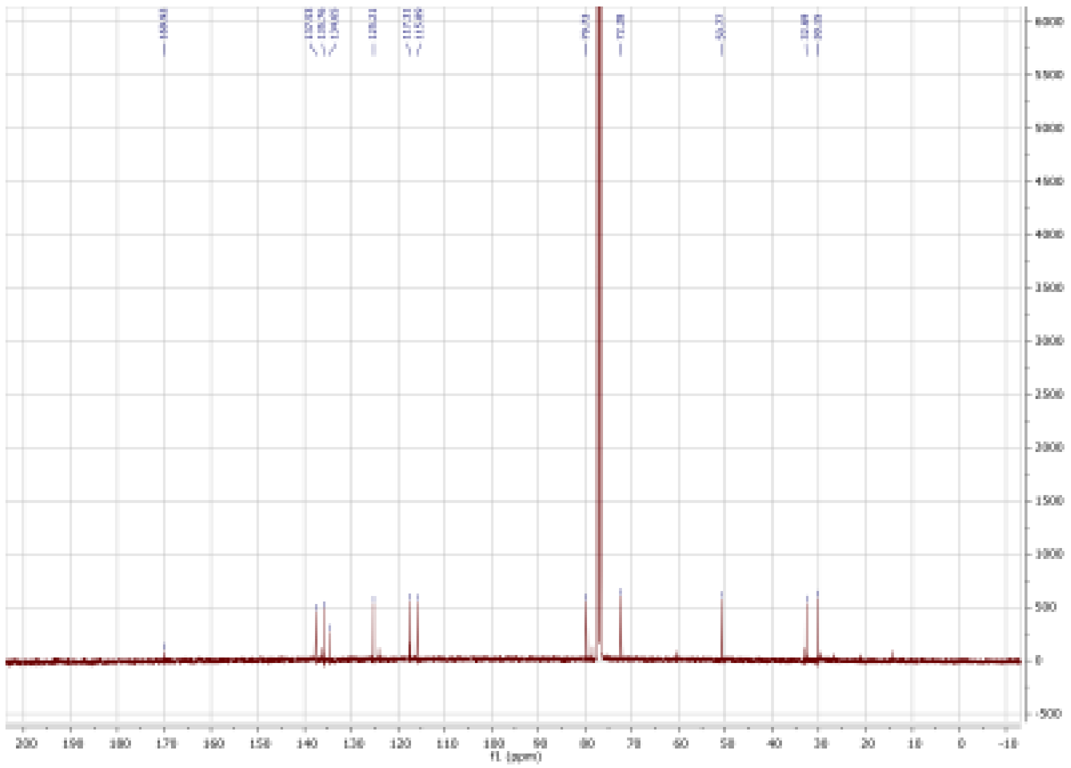

Supplementary figure 92: ^{1}H and ^{13}C NMR spectra of compound **S-25**

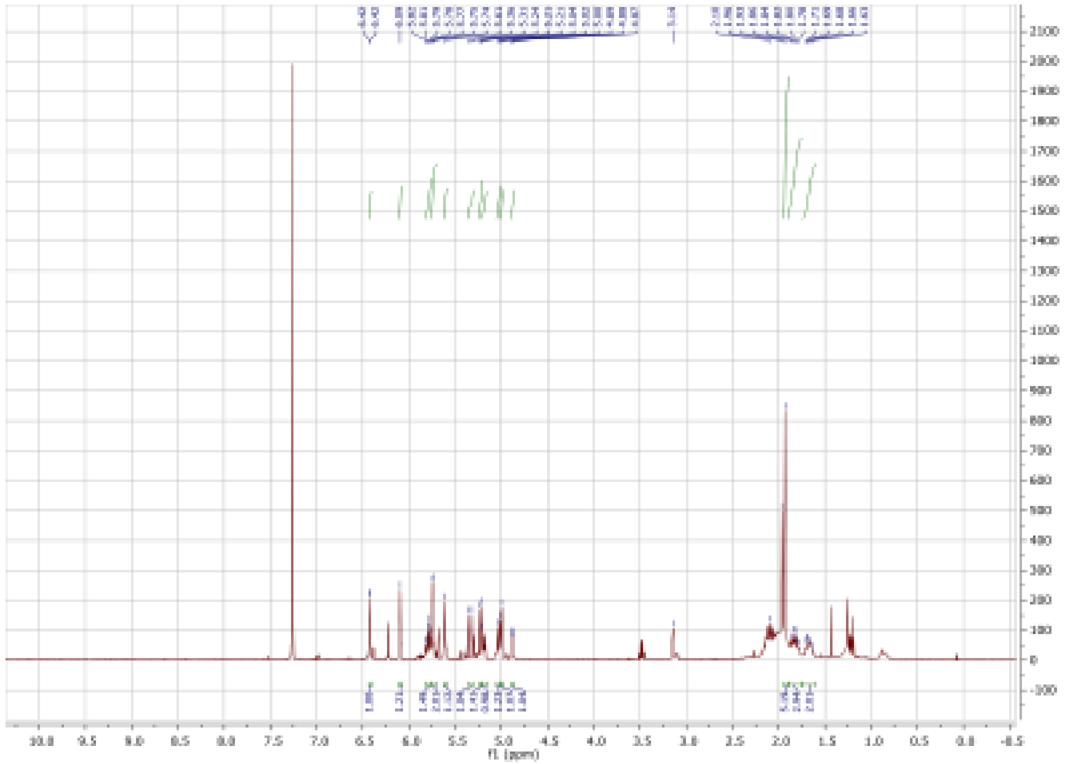

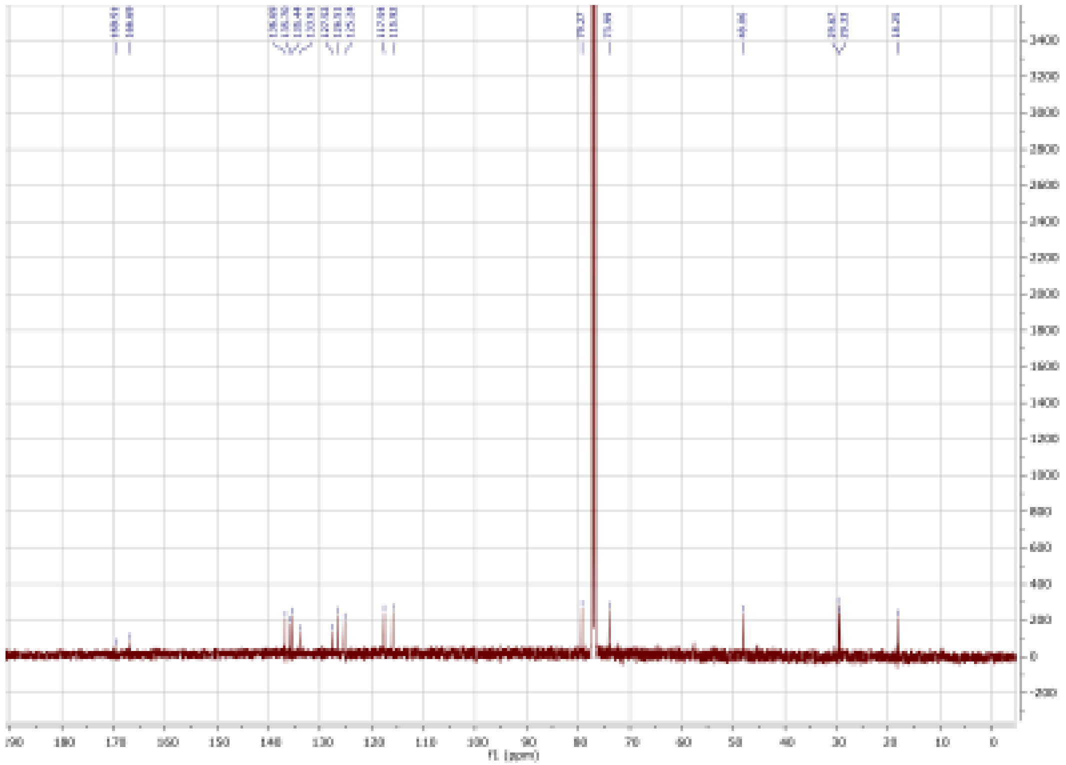

Supplementary figure 93: ^{1}H and ^{13}C NMR spectra of compound **S-26**

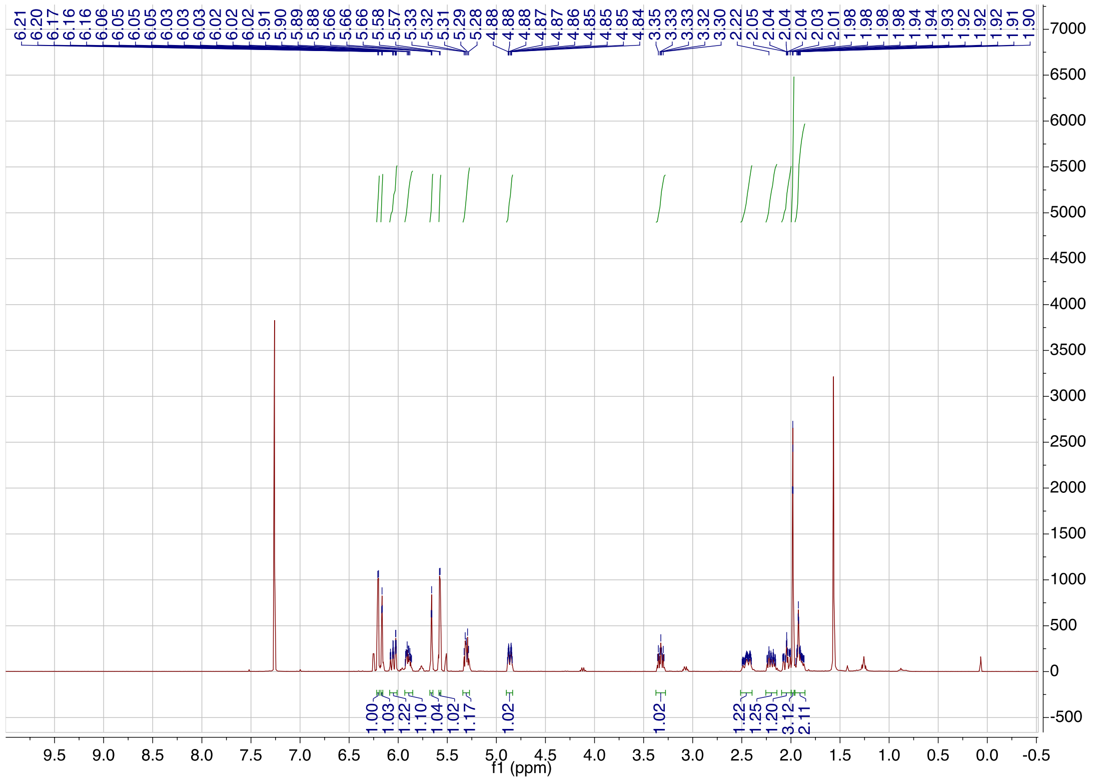

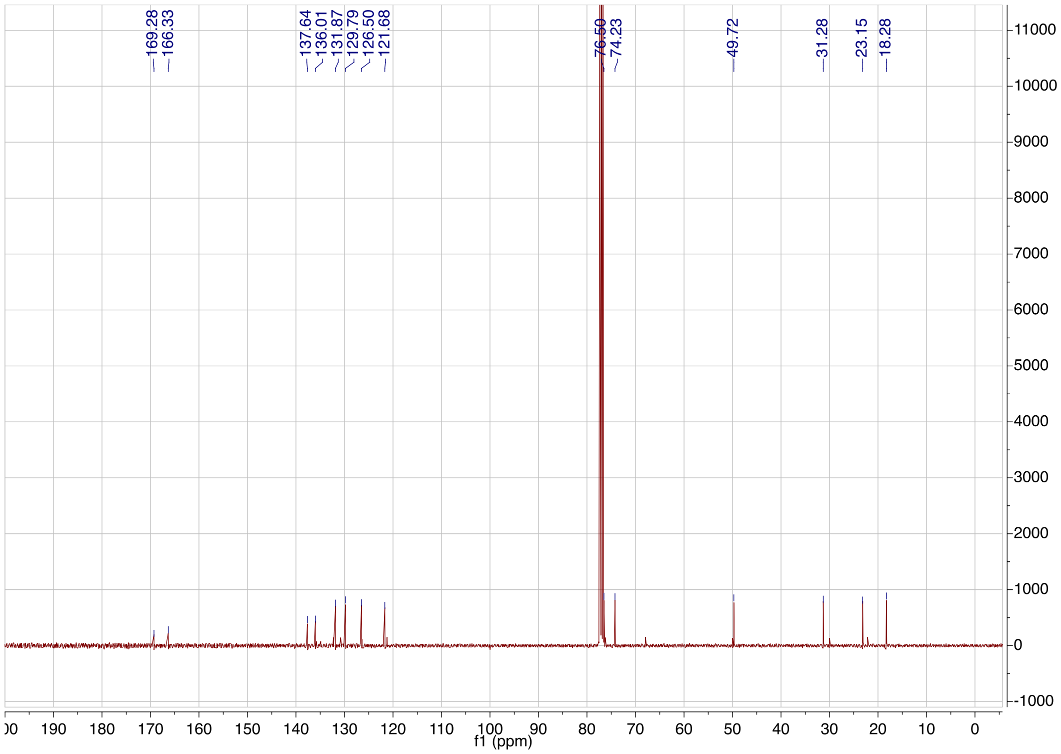

Supplementary figure 94: ^{1}H and ^{13}C NMR spectra of compound **26**

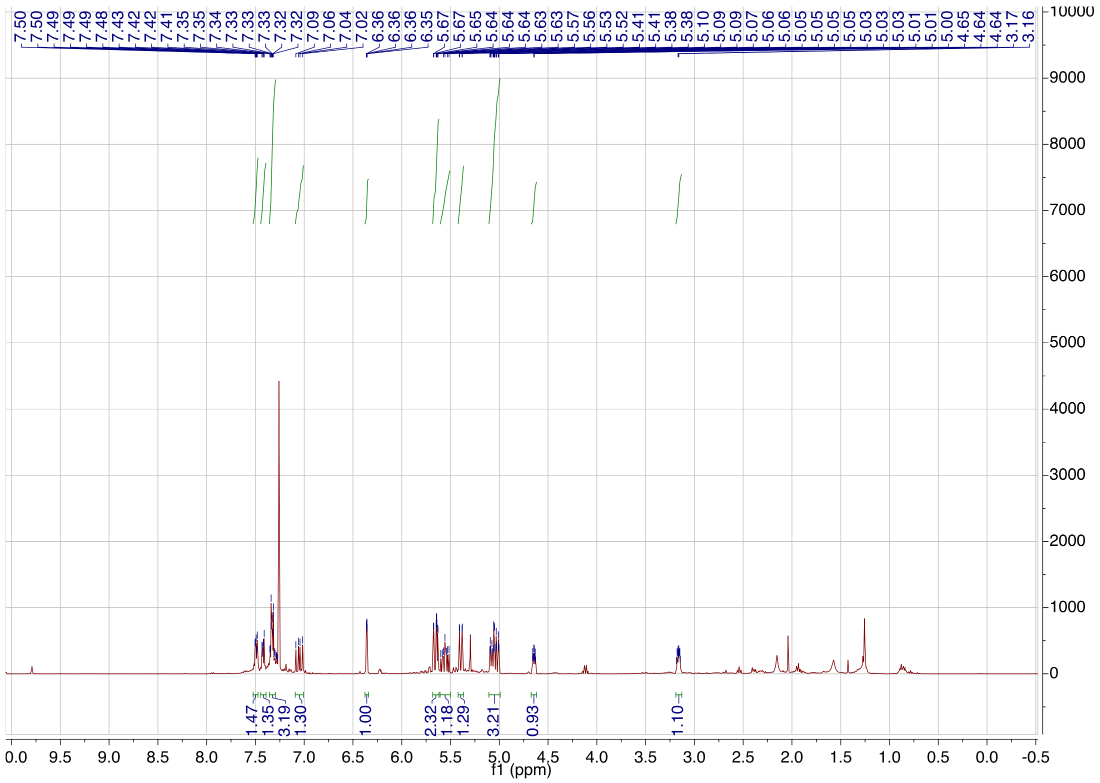

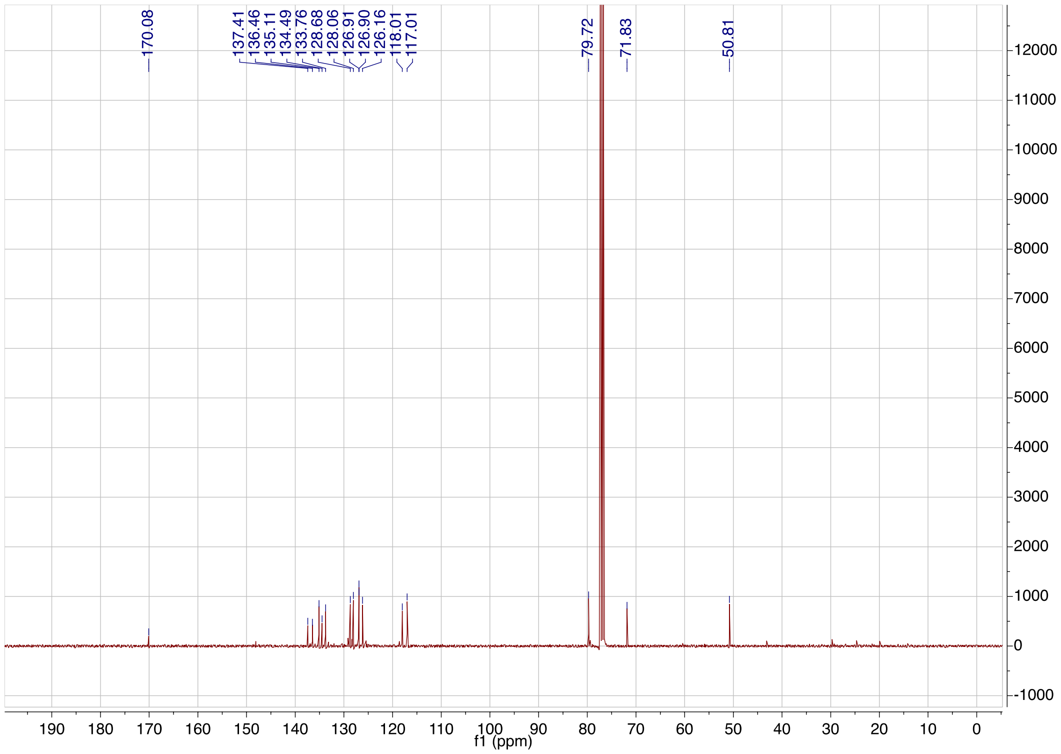

Supplementary figure 95: ^{1}H and ^{13}C NMR spectra of compound **S-27**

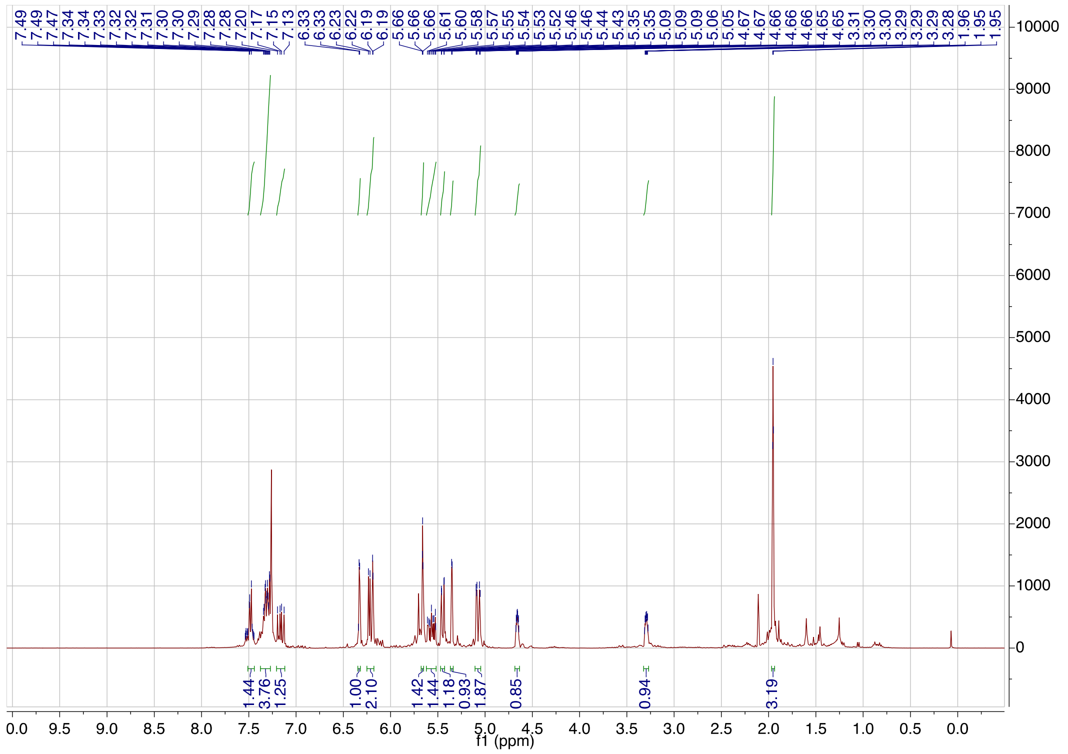

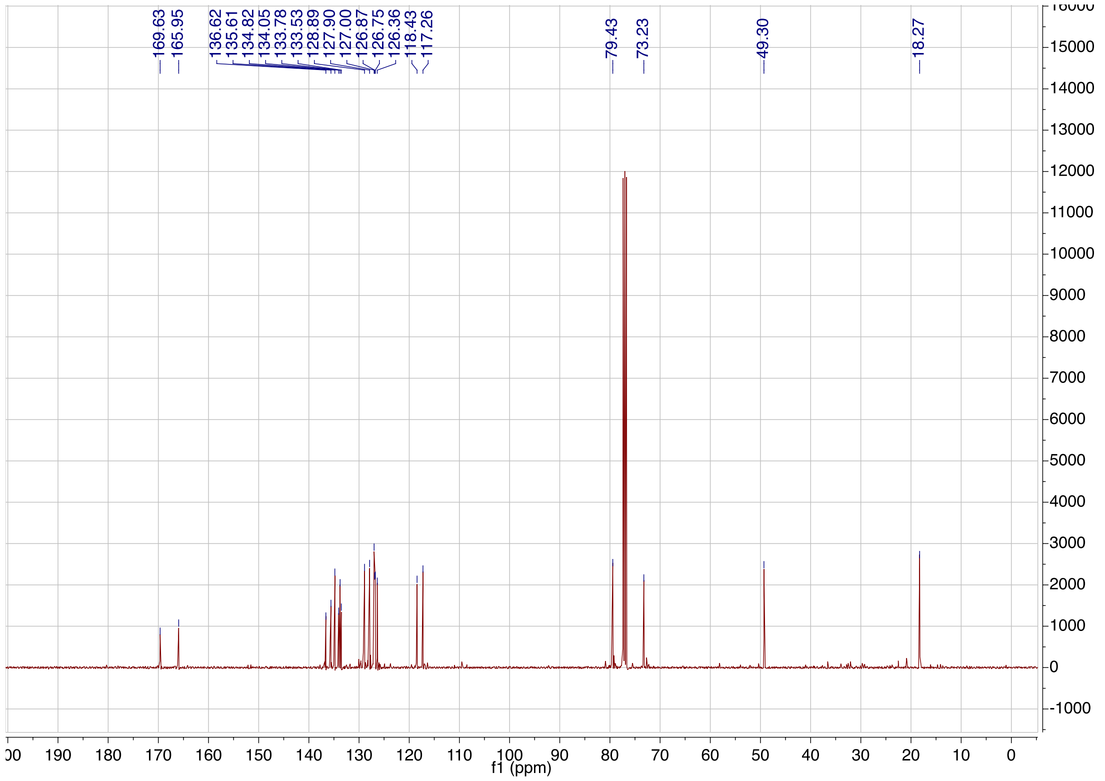

Supplementary figure 96: ^{1}H and ^{13}C NMR spectra of compound **S-28**

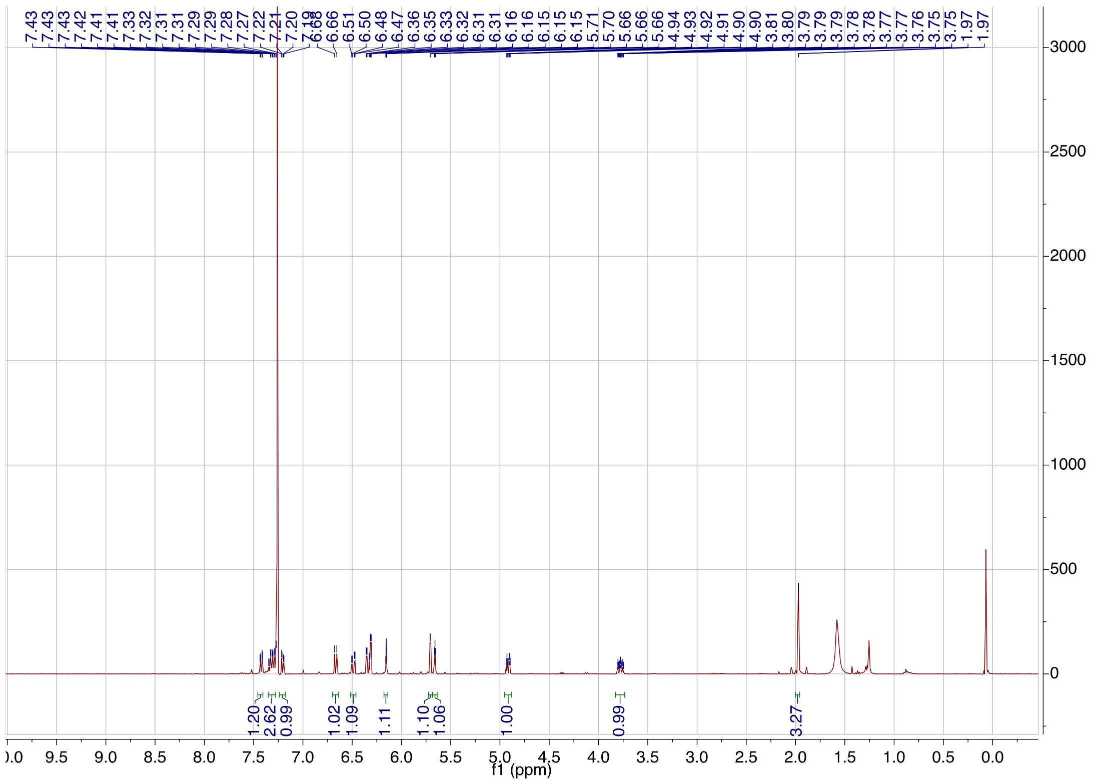

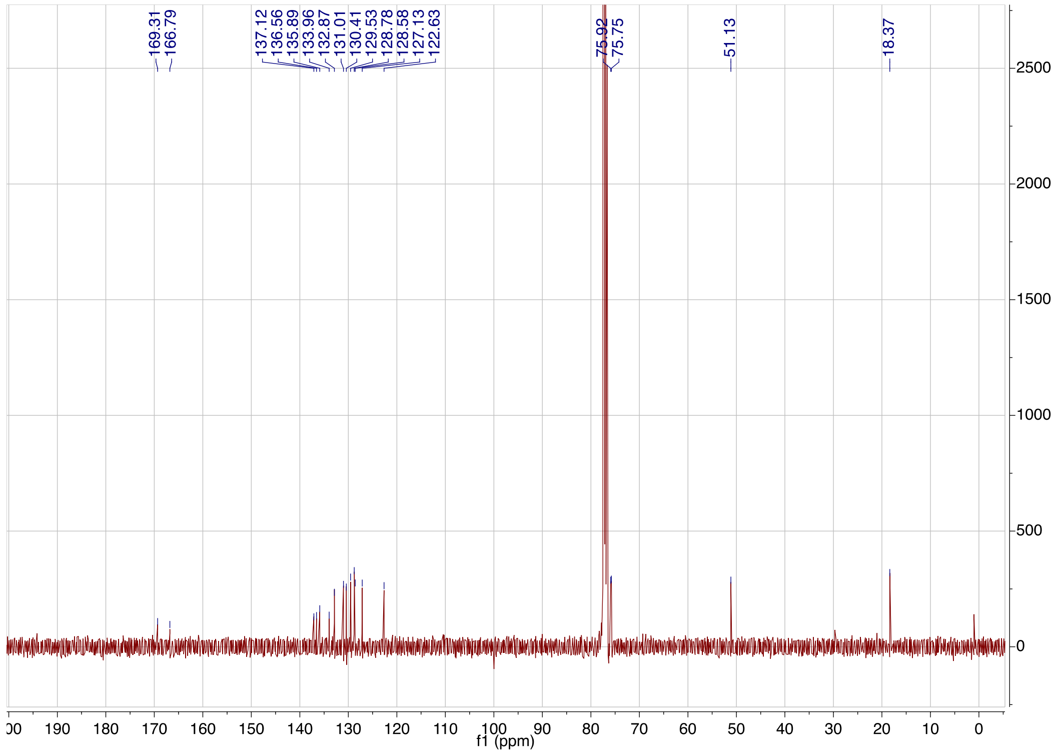

Supplementary figure 97: ^{1}H and ^{13}C NMR spectra of compound **28**

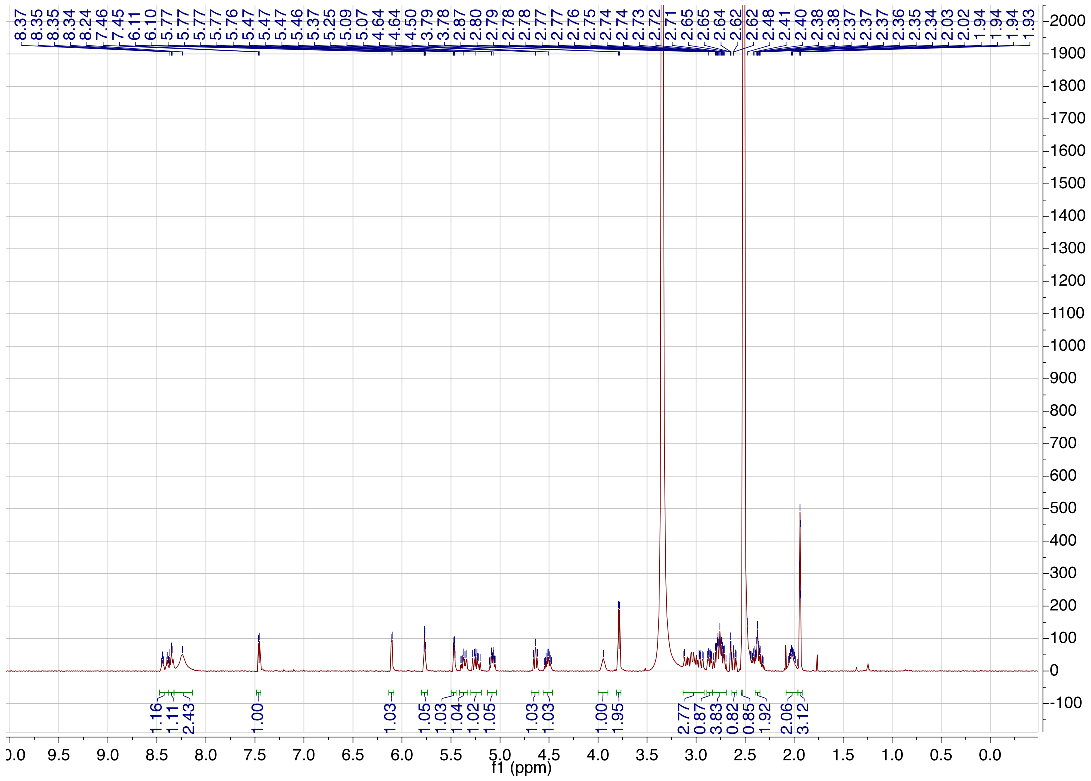

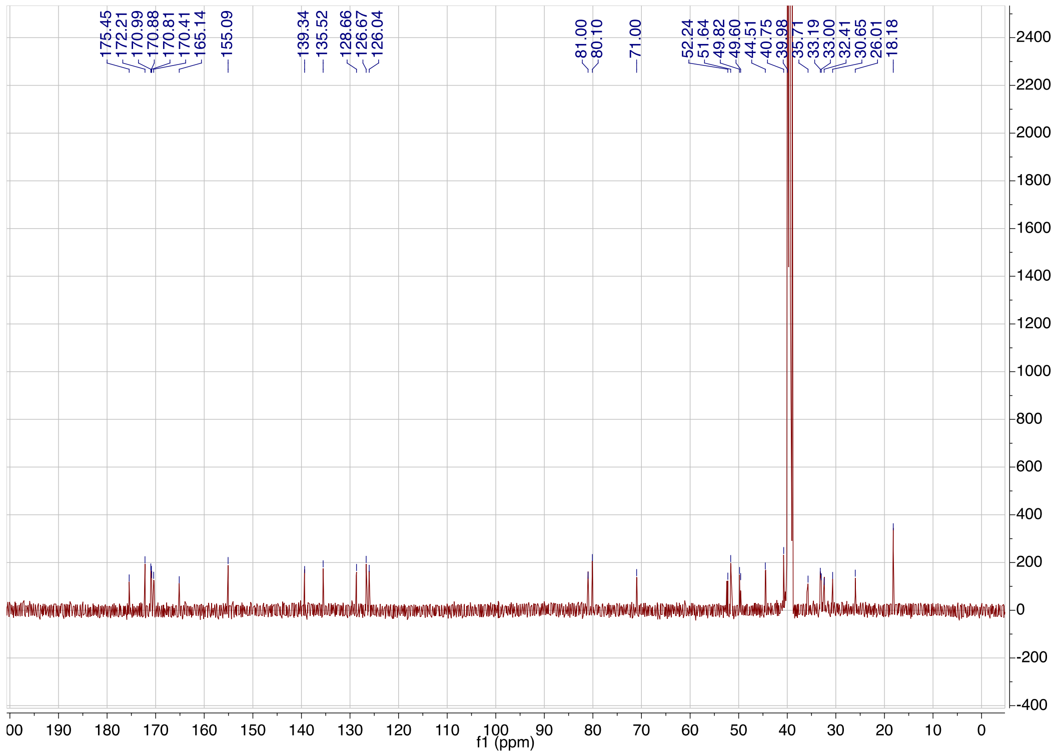

Supplementary figure 98: ^{1}H and ^{13}C NMR spectra of compound **Nordeoxyelephantopin-GSH adduct**

Supplementary methods

Summary of compounds used in biological experiments

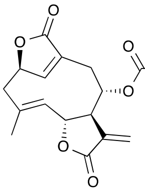

deoxyelephantopin

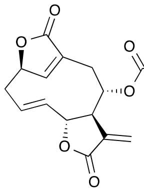

nordeoxyelephantopin

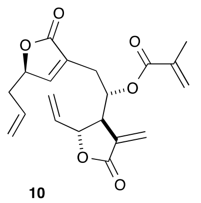

10

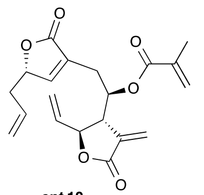

***ent*-10**

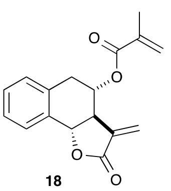

18

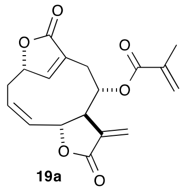

19a

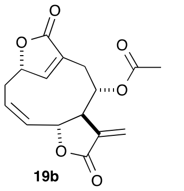

19b

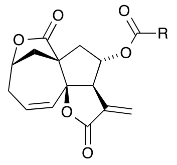

20a: R = C(Me)CH_2
20b: R = Me

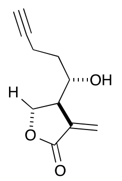

23a

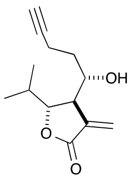

23b

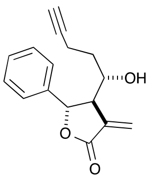

23c

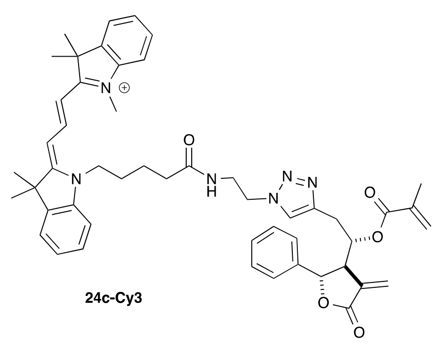

24c-Cy3

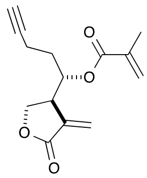

24a

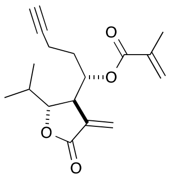

24b

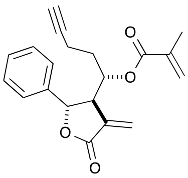

24c

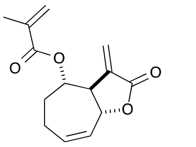

26

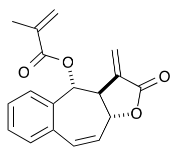

28

Biological materials. All materials were purchased from Sigma Aldrich unless otherwise stated. DMEM/High glucose media, phosphate buffered saline (PBS), MEM Non-Essential Amino Acids, Penicillin-Streptomycin (Pen/Strep) and Trypsin-EDTA were obtained from Life Technologies. Protein concentration was determined using the Bradford assay (Bio-Rad).

Cell culture and preparation of lysates. MCF7, Caco-2, HeLa, MDA-MB-231 and 293T cells were maintained in DMEM supplemented with 10% fetal calf serum (FCS), non-essential amino acids and Penicillin-Streptomycin. Cells were grown at 37 °C under 5% CO_2 atmosphere. Cells were allowed to grow to confluence and were harvested by scraping, centrifuged at 1'000 x g for 5 min. at 4 °C and resuspended in PBS. Cells were lysed by sonication to form cell lysates and protein concentration was determined using the Bradford assay.

Cloning and site-directed mutagenesis. The plasmid containing the hPPARγ-WT gene was purchased from OriGene. This plasmid was modified using a PCR based site directed mutagenesis to generate two plasmids containing the mutations D174A (Forward primer: 5' TAT GCC AGA TGT GAT CTT AAC TGT 3'; Reverse primer: 5' TCT GGC ATA GAT AAG CTT CAA TCT 3') or C176A (Forward primer: 5' AGA GCT GAT CTT AAC TGT CGG ATC 3'; Reverse primer: 5' ATC AGC TCT GTC ATA GAT AAG CTT 3'). Afterwards, the PCR reaction was incubated with DpnI (New England BioLabs Inc, 37 °C, 12 hours) to degrade the PPARγ-WT template plasmid. The plasmids were propagated via transformation into E. coli DH5α strain using heat shock.

Cytotoxicity assay. Cells were seeded (MCF7 and Caco-2: 50'000 cells/mL, MDA-MB-231: 20'000 cells/mL and HeLa: 10'000 cells/mL) in a 96-well plate and allowed to attach overnight. Cells were incubated with indicated compound (500x stock in DMSO) or DMSO for 3 days in culture media with media change every day. Cells were imaged using a SpectraMax i3 plate reader equipped with a SpectraMax MiniMax imaging cytometer (Molecular Devices). Alternatively, cells were fixed with 4% paraformaldehyde for 20 min., washed 3 times with PBS, stained with DAPI (5 µg/mL) in the presence of 0.05% saponin for 20 min., washed 3 times with PBS and imaged using the automated microscope ImageXpress (Molecular Devices). Cells or nuclei were then counted using ImageJ or MetaXpress and data was analyzed with GraphPad Prism (V6.03).

Caspase activity labeling for fluorescence cell imaging. MCF7 cells were seeded (100'000 cells/mL) in a 24-well plate containing a poly-lysine coated coverslip and left two days to attach and grow. Cells were washed one time with PBS and incubated with DMSO, 20 µM deoxyelephantopin (500x stock in DMSO) or 1 µM staurosporine for 8 hours at 37 °C. Media was replaced with 10 µM CaspACE (Promega) supplemented media and cells were incubated

for 30 min. at 37 °C under 5% CO_2 atmosphere. Cells were washed one time with PBS and fixed with 4% paraformaldehyde for 20 min. at r.t. Coverslips were washed three times with PBS, one time with water and then mounted on a slide with ProLong Gold antifade mountant with DAPI (Life Technologies). Cells were visualized with an LSM 700 confocal microscope (Zeiss).

Annexin V and PI staining for cell microscopy. MCF7 cells were seeded (100'000 cells/mL) in a 24-well plate containing a poly-lysine coated coverslip and left two days to attach and grow. Cells were washed one time with PBS and incubated with DMSO, 20 µM deoxyelephantopin (500x stock in DMSO) or 10 % ethanol for 8 hours at 37 °C. Cells were washed one time with PBS^{++} (0.1 g/L $CaCl_2$ and 0.1 g/L $MgCl_2$) and coverslips were transferred to a wet box. Cells were stained with Annexin-V-FLUOS staining kit (Roche) for 30 min., washed two times with PBS^{++} and fixed with 4% paraformaldehyde for 20 min. at r.t. Cells were washed three times with PBS^{++}, one time with water and then mounted on a slide with ProLong Gold antifade mountant with DAPI (Life Technologies). Cells were visualized with an LSM 700 confocal microscope (Zeiss).

***In situ* labeling experiments.** MCF7 cells were seeded (350'000 cells/mL) in a 6-well plate and left two days to attach and grow. The wells were washed one time with PBS and indicated concentration of compound (500x stock in DMSO) or DMSO was added in the culture media and incubated for four hours at 37 °C. Lysates (2 mg/mL, 25 µL) were prepared as described above and treated with 10 µM **24c-Cy3** in the dark for 1 hour at r.t. SDS-PAGE reducing loading buffer (4x) was added and proteins were separated using a 10% SDS-PAGE gel. Gels were visualized at 625 nm using a Hitachi FMBIO II Multi-View fluorescence scanner, then stained using Coomassie.

Transfection and gel-based *in situ* competition of PPARγ. HeLa cells were seeded (375'000 cells/mL) in a 6-well plate and left overnight to attach. Cells were transfected with the PPARγ-WT plasmid or GFP plasmid as mock using Lipofectamine 2000 (Invitrogen) according to the manual of the manufacturer. The wells were washed one time with PBS and 20 µM DEP (500x stock in DMSO) or DMSO was added in the culture media and incubated for four hours at 37 °C. Lysates (2 mg/mL, 25 µL) were prepared as described above and treated with 10 µM **24c-Cy3** in the dark for one hour at r.t. Reducing SDS-PAGE and visualization were performed as described above.

Gel-based assay for PPARγ kinetics. Recombinant human PPARγ (50 ng in 12.5 µL PBS) was treated with indicated concentration of either deoxyelephantopin or **19a** (0.5 µL of 25x stock in DMSO) for 10, 20, 30, 40, 50 or 60 min. Samples were then treated with 10 µM **24c-Cy3** (0.5 µL of 25x stock in DMSO) for one hour at r.t in the dark. Reducing SDS-PAGE and

visualization were performed as described above. Images were quantified with ImageJ and kinetics values were calculated using GraphPad Prism (V6.03).

Gel-based IC_{50} determination for PPARγ mutations. 293T cells were seeded (375'000 cells/mL) in a 6-well plate and left overnight to attach. Cells were transfected with the PPARγ plasmids or GFP plasmid as mock using Lipofectamine 2000. Lysates (2 mg/mL, 25 µL) were prepared as described above and treated with indicated concentrations of **19a** for one hour, followed by treatment with 10 µM **24c-Cy3** in the dark for one hour at r.t. Reducing SDS-PAGE and visualization were performed as described above.

SILAC labeling of MCF7 cells. MCF7 cells were passaged 6 times in DMEM without L-lysine and L-arginine (Thermo Scientific) supplemented with 10% dialyzed FCS (Thermo Scientific), non-essential amino acids, Penicillin-Streptomycin and either 100 mg/L $^{13}C_6$ L-lysine-HCl and $^{13}C_6$ $^{15}N_4$ L-arginine-HCl (heavy) or L-lysine-HCl and L-arginine-HCl (light) (Thermo Scientific).

SILAC competitive experiment for mass spectrometry. MCF7 light and heavy lysates (2 mg/ml, 0.5 mL) were treated with deoxyelephantopin (20 µM, 100x stock in DMSO) or DMSO for one hour, followed by labeling with **24c** (10 µM, 100x stock in DMSO) for one hour at r.t. Proteins were subjected to click chemistry. Biotin azide (20 µM, 50x stock in DMSO), tris(2-carboxyethyl)phosphine hydrochloride (TCEP) (1 mM, 50x fresh stock in water), tris[(1-benzyl-1*H*-1,2,3-triazol-4-yl)methyl]amine (TBTA) (100 µM, 16x stock in DMSO:*t*Butanol 1:4), and copper(II) sulfate (1 mM, 50x stock in water) were added to the proteomes and left to react for one hour at r.t. Light and heavy proteomes were mixed together at a ratio of 1:1. Protein was precipitated by adding MeOH (4 vol.), $CHCl_3$ (1 vol.) and water (3 vol.) to the reaction mixture and the turbid mixture was centrifuged for five min. at 14'000 x g at 4 °C yielding a protein layer between the aqueous and organic layers. The protein layer was isolated, dried and solubilized in 2% SDS in PBS via sonication. Tube was centrifuged at 4'700 x g for five min. and soluble fraction was transferred to a new tube. PBS was added to give a final SDS concentration of 0.2%. 140 µL of streptavidin agarose beads were added and the mixture was rotated for four hours at r.t. Beads were washed with 1% SDS in PBS (1x 10 mL), PBS (3x 10 mL), and water (3x 10 mL). Beads were resuspended in 6 M urea in PBS (500 µL), reduced with 10 mM neutralized TCEP (20x fresh stock in water) for 30 min. at r.t., and alkylated with 55 mM iodoacetamide (400 mM fresh stock in water) for 30 min. at r.t. in the dark. Beads were pelleted by centrifugation (1'400 x g, two min.) and resuspended in 150 µL of 2 M urea, 1 mM $CaCl_2$ (100x stock in water) and trypsin (Promega, 1 µL of 0.5 µg/µL) in 50 mM NH_4HCO_3. The digestion was performed for 12 hours at 37 °C. Samples were acidified to a final concentration of 5% acetic acid, desalted over a self-packed C18 spin column and dried.

Samples were analyzed by LC-MS/MS (see below) and the MS data was processed with MaxQuant (see below).

***In situ* competitive experiment for mass spectrometry.** MCF7 or Caco-2 cells were seeded (350'000 cells/mL) in a 10 cm dish and left two days to attach and grow. The dishes were washed one time with PBS and cells were incubated in culture media in the presence of DMSO or 20 µM DEP (1000x stock in DMSO) for four hours. MCF7 lysates were prepared (2 mg/mL, 0.5 mL) and treated with **24c** (10 µM, 100x stock in DMSO) for one hour at r.t, followed by click chemistry (see above). Protein precipitation, streptavidin enrichment, washes, reduction, alkylation and trypsin digestion were performed as described above. Samples were acidified to a final concentration of 5% acetic acid, desalted over a self-packed C18 spin column and dried. Samples were analyzed by LC-MS/MS (see below) using inclusion lists containing m/z and charge of the expected peptides of indicated proteins (see Figure 6c and PPARγ). The MS data was processed with MaxQuant (see below).

***In situ* global proteomics profiling using LC-MS/MS.** Caco-2 cells were seeded (350'000 cells/mL) in a 6-well plate, allowed to grow to confluence and to differentiate for 28 days. The wells were washed one time with PBS and treated with either DMSO, 10 µM rosiglitazone, 50 µM T0070907 or 20 µM DEP for 24 hours. Cells were washed two times with PBS and lysates were prepared. Samples (30 µg) were denatured with 6 M urea in 50 mM NH_4HCO_3, reduced with 10 mM TCEP for 30 min. and alkylated with 25 mM iodoacetamide for 30 min. in the dark. Samples were diluted to 2 M urea with 50 mM NH_4HCO_3, and digested with trypsin (1 µL of 0.5 µg/µL) in the presence of 1 mM $CaCl_2$ for 12 hours at 37 °C. Samples were acidified to a final concentration of 5% acetic acid, desalted over a self-packed C18 spin column and dried. Samples were analyzed by LC-MS/MS (see below) and the MS data was processed with MaxQuant (see below).

PPARγ enrichment for mass spectrometry. MCF7 lysate (2 mg/ml, 0.5 mL) supplemented with 1 µg of human PPARγ, was treated with either **23c** or **24c** (10 µM, 50x stock in DMSO) for one hour at r.t., followed by click chemistry (see above). Protein precipitation, streptavidin enrichment, washes, reduction, alkylation and trypsin digestion were performed as described above. Samples were acidified to a final concentration of 5% acetic acid, desalted over a self-packed C18 spin column and dried. Samples were analyzed by LC-MS/MS (see below) and the MS data was processed with MaxQuant (see below).

LC-MS/MS analysis. Peptides were resuspended in water with 0.1% formic acid (FA) and analyzed using Proxeon EASY-nLC 1000 nano-UHPLC coupled to QExactive Plus Quadrupole-Orbitrap mass spectrometer (Thermo Scientific). The chromatography column consisted of a 30 cm long, 75 µm i.d. microcapillary capped by a 5 µm tip and packed with

ReproSil-Pur 120 C18-AQ 2.4 µm beads (Dr. Maisch GmbH). LC solvents were 0.1% FA in H_2O (Buffer A) and 0.1% FA in MeCN (Buffer B). Peptides were eluted into the mass spectrometer at a flow rate of 300 nL/min. over a 240 min. linear gradient (3-35% Buffer B) at 50 °C. Data was acquired in data-dependent mode (top-20, NCE 30, R = 17'500) after full MS scan (R = 70'000, m/z 400-1'300). Dynamic exclusion was set to 10 s, peptide match to prefer and isotope exclusion was enabled.

MaxQuant analysis. The MS data was analyzed with MaxQuant (V1.5.2.8) and searched against the human proteome (Uniprot, 89,649 entries) and a common list of contaminants (included in MaxQuant). The first peptide search tolerance was set to 20 ppm, 10 ppm was used for the main peptide search and fragment mass tolerance was set to 0.02 Da. The false discovery rate for peptides, proteins and sites identification was set to 1%. The minimum peptide length was set to 6 amino acids and peptide re-quantification, label-free quantification (MaxLFQ) and "match between runs" were enabled. The minimal number of peptides per protein was set to two. Oxidized methionines and N-terminal acetylation were searched as variable modifications and carbamidomethylation of cysteines was searched as a fixed modification. For SILAC experiments $^{13}C_6$ L-lysine and $^{13}C_6$ $^{15}N_4$ L-arginine were set as heavy isotope labels and $^{12}C_6$ L-lysine and $^{12}C_6$ $^{14}N_4$ L-arginine as light isotope labels.

19a docking on human PPARγ. 19a was covalently docked to the crystal structure of human PPARγ (PDB: 3DZU) on Cys^{190} using the GOLD suite (V5.3, CCDC). The default GOLD fitness function was used to determine the best binding configuration. The distance for hydrogen bonding was set to 3 Å.

IAA competition of 23c, 24c and DEP on human PPARγ. Recombinant human PPARγ (500 ng in 9 µL PBS) was treated with either 100 µM DEP, **23c**, **24c** or DMSO (1 µL of 10x stock in DMSO) for one hour. Samples were denatured with 6 M urea in 50 mM NH_4HCO_3, reduced with 10 mM TCEP for 30 min. and alkylated with 25 mM iodoacetamide for 30 min. in the dark. Samples were diluted to 2 M urea with 50 mM NH_4HCO_3, and digested with trypsin (0.25 µL of 0.05 µg/µL) in the presence of 1 mM $CaCl_2$ for 12 hours at 37 °C. Samples were acidified to a final concentration of 5% acetic acid, desalted over a self-packed C18 spin column and dried. Samples were resuspended in 0.1% FA in water and analyzed by LC-MS/MS (see above). MS data was analyzed in MaxQuant (see above).

Labeling of recombinant IKK-β. Recombinant human IKK-β (Adipogen International, 2500 ng in 25 µL PBS) was treated with DMSO or 10 µM of **24c** (1 µL of 25x stock in DMSO) for one hour. Click chemistry was initiated by the addition of TAMRA azide (50 µM, 25x stock in DMSO, tris(2-carboxyethyl)phosphine hydrochloride (TCEP, 1 mM, fresh 50x stock in water), tris[(1-benzyl-1H-1,2,3-triazol-4-yl)methyl]amine (TBTA, 100 µM, 16x stock in

DMSO:tButanol 1:4), and copper(II) sulfate (1 mM, 50x stock in water) to the lysate and incubated in the dark for one hour at r.t. Reducing SDS-PAGE and visualization were performed as described above.

General methods for chemical reaction and analysis. All reactions were carried out under a nitrogen atmosphere with dry solvents under anhydrous conditions, unless otherwise noted. Anhydrous solvents were obtained by passing them through commercially available alumina columns (Innovative technology, Inc., MA). Reactions were monitored by thin-layer chromatography (TLC) carried out on 0.25 mm Merck silica gel plates (60, F_{254}) visualized with UV light and developed using either $KMnO_4$ or vanillin solutions. Merck silica gel (60, particle size 0.040-0.063 mm) was used for flash column chromatography. Purifications by HPLC were carried out on Agilent 1100 series with a column Zorbax Eclipse XD8 C18 (9.4 mm × 25 cm). NMR spectra were recorded on Bruker 400 (^{1}H), 100 (^{13}C) MHz or 500 (^{1}H), 120 (^{13}C) MHz. Chemical shifts δ are given in parts per million (ppm) and calibrated using the residual solvent resonance as an internal reference (^{1}H NMR: $CHCl_3$ 7.26 ppm; ^{13}C NMR: $CDCl_3$ 77.0 ppm). The following abbreviations were used for the multiplicities: b = broad, s = singlet, d = doublet, t = triplet, q = quartet, quint = quintuplet, m = multiplet. LC/MS spectra were recorded on a HPLC Thermo Scientific Accela with a spectrometer Surveyor MSQ Plus and a column Hypersil gold Thermo C18 (5 cm × 2.1 mm, 1.9 mm particles).

General procedure A for the tandem $LiAlH_4$ reduction/iodination reaction. A solution of $LiAlH_4$ in THF (3.0 equiv) was added to a solution of the corresponding alcohol (1.0 equiv) in THF (0.1 M) at 0 °C. The resulting suspension was then heated to reflux for 3 hours. EtOAc (10 equiv) was added at 0 °C. After 20 minutes, iodine (3.0 equiv) was added at 0 °C and the solution was stirred at this temperature for 1 hour. The reaction was quenched with sat. aqueous $NaHCO_3$ and sat. aqueous $Na_2S_2O_3$. The aqueous phase was extracted with EtOAc and the collected organic layers were washed with brine, dried over Na_2SO_4 and filtered. Evaporation of the solvent under reduced pressure was followed by flash chromatography as indicated.

General procedure B for carbonylation using $Ni(CO)_2(PPh_3)_2$. $Ni(CO)_2(PPh_3)_2$ (1.1 equiv) and triethylamine (2.0 equiv) were added to a solution of the corresponding alcohol (1.0 equiv) in toluene (0.07 M) at room temperature. The solution was stirred at reflux for 15 minutes and then cooled down to room temperature. Filtration of the reaction mixture as indicated yielded the desired lactone.

General procedure C for the asymmetric tandem Tsuji-Trost allylation/[3,3]-Cope rearrangement sequence. Pd_2dba_3 (0.025 equiv) was added in one portion to a solution of the appropriate DACH-phenyl Trost ligand (0.06 equiv) in dry NMP (1 M) at room temperature. The resulting mixture was stirred under inert atmosphere at room temperature for 15 minutes and added via cannula to a solution of allyl carbonate **14** (1 equiv) in dry NMP (0.2 M) cooled to −20 °C. Stirring was continued overnight. The reaction mixture was poured into brine and extracted with Et_2O. The combined organic layers were washed with water, brine, dried over Na_2SO_4, filtered and concentrated *in vacuo*. The residue was re-dissolved in toluene (0.2 M), transferred to a microwave vial, sealed and irradiated under microwaves at 180 °C for 30 min. The reaction mixture was filtered on silica and concentrated *in vacuo*. The residue was purified by flash chromatography on silica gel (cyclohexane/EtOAc 10:1 to 1:1).

General procedure D for the acetal hydrolysis/Barbier coupling. To a solution of the appropriate acetal (1 mmol, 1 equiv) in THF (1 mL) was added Amberlyst 15 (100 mg) and water (0.10 mL). Stirring was continued until complete consumption of the starting material. The resulting solution was filtered over Celite® into a round-bottom flask containing bromolactone **2** (1.5 equiv). Zinc powder (5 equiv), indium powder (0.5 equiv) and 10% aqueous NH_4Cl (0.5 mL) were successively added. Upon complete consumption of the aldehyde, the reaction mixture was diluted with EtOAc, washed with 1 M HCl, dried over Na_2SO_4, filtered on silica and concentrated *in vacuo*. The residue was dry-loaded and purified by flash chromatography on silica gel (cyclohexane/EtOAc 10:1 to 1:1) to afford the desired secondary alcohol.

General procedure E for the methacryloylation with methacryloyl anhydride. A solution of the appropriate secondary alcohol (1 equiv) in dry CH_2Cl_2 (0.1 M) cooled to 0 °C was successively treated with Et_3N (1.5 equiv), DMAP (0.2 equiv) and methacryloyl anhydride (1.2 equiv), and the resulting mixture was stirred until complete consumption of the starting material. The reaction mixture was then diluted with Et_2O, water was added and stirring was continued for 30 min, dried over Na_2SO_4, filtered on silica and concentrated *in vacuo*. The residue was dry-loaded and purified by flash chromatography on silica gel (cyclohexane/EtOAc 10:1 to 1:1) to afford the desired methacrylate derivative.

General procedure F for the RCM with Grubbs 1 as catalyst. A refluxing solution of the appropriate diene (1 equiv) in CH_2Cl_2 (0.1 mM) was treated with a freshly prepared solution of Grubbs 1 catalyst (0.2 equiv) and stirring was continued under reflux and N_2 sparging for 4 hours. Silica (10 mg/1 mg of starting diene) was added and the resulting mixture was concentrated *in vacuo*. Partitioning by flash chromatography on silica gel (cyclohexane/EtOAc 10:1 to 1:2) allowed partial elimination of catalyst residues, recovery of the unreacted starting

material and the fraction containing the desired RCM product was submitted to reverse phase preparative HPLC. Injection as a DMSO solution and gradient elution using the stated eluent mixtures afforded the desired analogue.

General procedure G for methacryloylation with methacryloyl chloride. Methacryloyl chloride (1.1 equiv) was added to a solution of the corresponding alcohol (1.0 equiv) and Et_3N (1.2 equiv) in CH_2Cl_2 (0.1 M) at room temperature. The mixture was stirred for 1 hour. Filtration of the reaction mixture as indicated yielded the desired ester.

General procedure H for acetylation. Acetyl chloride (1.1 equiv) was added to a solution of the corresponding alcohol (1.0 equiv) and pyridine (1.2 equiv) in CH_2Cl_2 (0.1 M) at room temperature. The mixture was stirred for 1 hour. Filtration of the reaction mixture as indicated yielded the desired ester.

General procedure I for Mitsunobu esterification. A solution of the corresponding alcohol (1.0 equiv) and PPh_3 (1.0 equiv) in Et_2O was added to a solution of (2-bromomethyl)acrylic acid (1.0 equiv) and DIAD (1.0 equiv) in Et_2O (0.1 M) at 0 °C. The mixture was then stirred at room temperature for 12 hours. Evaporation of the solvent under reduced pressure was followed by flash chromatography as indicated.

General procedure J for the direct Barbier reaction with aldehydes. Zinc powder (1.3 equiv) was added to a solution of the corresponding bromolactone (1.1 equiv) and the corresponding aldehyde (1.0 equiv) in a mixture of THF (1 M) and sat. aqueous NH_4Cl (2 M) at room temperature. The mixture was stirred for 20 minutes. After addition of water, the aqueous phase was extracted with EtOAc and the collected organic layers were washed with brine, dried over Na_2SO_4 and filtered. Evaporation of the solvent under reduced pressure was followed by flash chromatography as indicated.

General procedure H for RCM with Grubbs II as catalyst. A refluxing solution of the appropriate diene (1 equiv) in dry DCM (10 mM) was treated with a freshly prepared solution of Grubbs 2 catalyst (0.2 equiv) in dry DCM and stirring was continued under reflux until complete consumption of the starting material. Silica (10 mg/1 mg of starting diene) was added and the resulting mixture was concentrated *in vacuo*. The dry-loaded residue was purified by flash chromatography on silica gel (cyclohexane/EtOAc 10:1 to 1:1) to afford the desired cyclic analogue.

Synthesis of bromolactone 2

Compound S-1

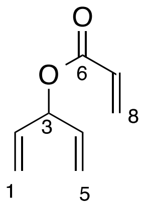

Acryloyl chloride (3.4 mL, 41 mmol, 1.1 equiv) was added to a solution of 1,4-pentadien-3-ol **7** (4.0 mL, 41 mmol, 1.0 equiv) and triethylamine (6.4 mL, 45 mmol, 1.1 equiv) in CH_2Cl_2 (100 mL) at 0 °C. The mixture was stirred for 1 hour and filtered on silica (SiO_2, 95/5 pentane/Et_2O) and concentrated in vacuo to yield ester **S-1** as a colorless oil (5.4 g, 39 mmol, 95%). **R$_f$**=0.85 (75/25 petroleum ether/EtOAc). ^{1}**H-NMR** (400 MHz, $CDCl_3$, 25 °C): δ 6.44 (dd, J=17.3, 1.5 Hz, 1H, H-8), 6.16 (dd, J=17.3, 10.3 Hz, 1H, H-7), 5.92-5.83 (m, 3H, H-4, H-2, H-8), 5.80-5.77 (m, 1H, H-3), 5.33 (d, J=17.3 Hz, 2H, H-5, H-1), 5.25 (d, J=10.3 Hz, 2H, H-5, H-1) ppm. ^{13}**C-NMR** (100 MHz, $CDCl_3$, 25 °C): δ 165.1 (C-6), 134.9 (×2, C-4, C-2), 130.9 (C-8), 128.5 (C-7), 117.5 (C-5, C-1), 75.1 (C-3) ppm.

Compound 8

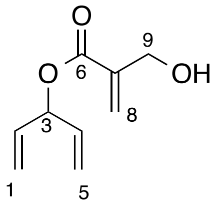

DABCO (4.4 g, 39 mmol, 1.0 equiv) was added to a solution of ester **S-1** (5.4 g, 39 mmol, 1.0 equiv) and formaldehyde (3.0 mL, 37% wt in H_2O, 39 mmol, 1.0 equiv) in H_2O/1,4-dioxane (400 mL, 1/1) at room temperature. The mixture was stirred for 48 hours and diluted with EtOAc. The organic phase was washed with saturated aqueous NH_4Cl, dried over Na_2SO_4 and filtered. Evaporation of the solvent under reduced pressure yielded alcohol **8** as a yellow oil (2.8 g, 17 mmol, 43%). **R$_f$**=0.59 (75/25 petroleum ether/EtOAc). ^{1}**H-NMR** (400 MHz, $CDCl_3$, 25 °C): δ 6.32 (bs, 1H, H-8), 5.92-5.79 (m, 4H, H-4, H-3, H-2, H-8), 5.34 (d, J=17.3 Hz, 2H, H-5, H-1), 5.26 (d, J=10.3 Hz, 2H, H-5, H-1), 4.36 (s, 2H, H-9) ppm (OH signal not visible). ^{13}**C-NMR** (100 MHz, $CDCl_3$, 25 °C): δ 165.2 (C-6), 139.5 (C-7), 134.7 (×2, C-4, C-2), 126.0 (C-8), 117.8 (×2, C-5, C-1), 75.5 (C-3), 62.6 (C-9) ppm.

Compound S-2

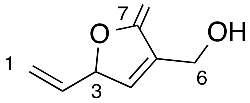

Grubbs II catalyst (127 mg, 0.15 mmol, 0.10 equiv) was added to a refluxing solution of alcohol **8** (252 mg, 1.5 mmol, 1.0 equiv) in CH_2Cl_2 (1.5 L, 1 mM). The mixture was stirred at reflux for 2 hours. DMSO (0.53 mL, 7.5 mmol, 50 equiv/catalyst) was added at room temperature and the mixture was stirred for 12 hours. Evaporation of the solvent under reduced pressure followed by flash chromatography (SiO_2, 80/20 pentane/Et_2O to 100% Et_2O) yielded lactone **S-2** as a yellow oil (157 mg, 1.1 mmol, 75%). **R$_f$**=0.26 (50/50 petroleum ether/EtOAc). ^{1}**H-**

NMR (400 MHz, $CDCl_3$, 25 °C): δ 7.24 (bs, 1H, H-4), 5.71 (ddd, *J*=17.3, 10.3, 6.8 Hz, 1H, H-2), 5.48 (d, *J*=17.3 Hz, 1H, H-1), 5.39 (bd, *J*=6.8 Hz, 1H, H-3), 5.36 (d, *J*=10.3 Hz, 1H, H-1), 4.45 (s, 2H, H-6) ppm (OH signal not visible). **^{13}C-NMR** (100 MHz, $CDCl_3$, 25 °C): δ 172.4 (C-7), 147.7 (C-4), 133.4 (C-5), 131.6 (C-2), 120.0 (C-1), 82.4 (C-3), 57.1 (C-6) ppm. **LC-MS (ESI$^+$)**: m/z calculated for $C_7H_9O_3^+$ $[M+H]^+$: 141.05; found 141.02.

Compound 2

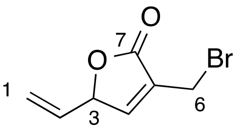

CBr_4 (759 mg, 2.3 mmol, 1.0 equiv) and PPh_3 (599 mg, 2.3 mmol, 1.0 equiv) were added to a solution of lactone **S-2** (320 mg, 2.3 mmol, 1.0 equiv) in CH_2Cl_2 (20 mL) at 0°C. The reaction was stirred for 1 hour at this temperature. Filtration on silica (95/5 to 80/20 pentane/Et_2O) yielded lactone **2** as a pale yellow oil (408 mg, 2.0 mmol, 88%). **R_f**=0.73 (50/50 petroleum ether/EtOAc). **^{1}H-NMR** (400 MHz, $CDCl_3$, 25 °C): δ 7.36 (q, *J*=1.5 Hz, 1H, H-4), 5.71 (ddd, *J*=17.3, 10.3, 6.8 Hz, 1H, H-2), 5.50 (d, *J*=17.3 Hz, 1H, H-1), 5.41-5.38 (m, 1H, H-3), 5.38 (d, *J*=10.3 Hz, 1H, H-1), 4.10 (t, *J*=1.5 Hz, 2H, H-6) ppm. **^{13}C-NMR** (100 MHz, $CDCl_3$, 25 °C): δ 170.6 (C-7), 150.8 (C-4), 140.1 (C-5), 131.2 (C-2), 120.3 (C-1), 81.6 (C-3), 20.6 (C-6) ppm.

Racemic synthesis of butenolide 6

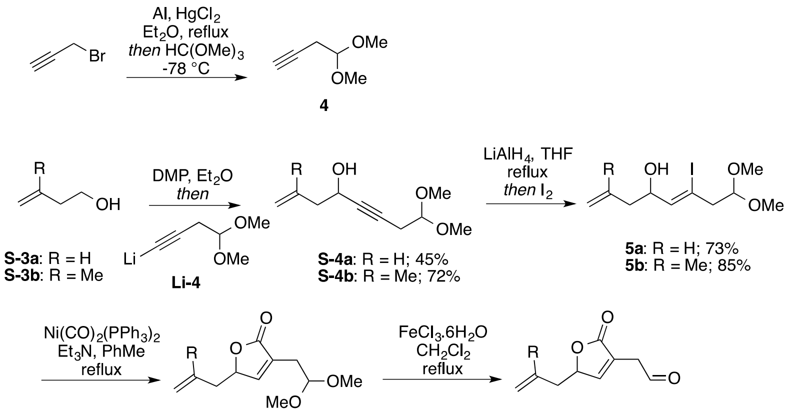

Compound 4

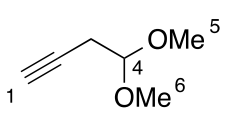

A solution of propargyl bromide in toluene (50 mL, 80% wt, 0.45 mol, 1.5 equiv) was added to a refluxing suspension of aluminium powder (18.6 g, 0.69 mol, 2.3 equiv) and $HgCl_2$ (1.1 g, 3.9 mmol, 0.013 equiv) in Et_2O (300 mL) over 2 hours. The grey suspension was refluxed for 1 hour and cooled to -78 °C. Trimethyl orthoformate (33 mL, 0.30 mol, 1.0 equiv) was added. After 2 hours, water (200 mL) and 1 M aqueous NaOH (60 mL) were added and the mixture was stirred for 15 minutes

at room temperature. The aqueous phase was extracted with Et_2O. The collected organic layers were washed with brine, dried over Na_2SO_4 and filtered. Evaporation of the solvent under reduced pressure followed by flash chromatography (SiO_2, 80/20 pentane/Et_2O) yielded alkyne **4** as a pale yellow oil (34 g, 0.30 mol, quant). $\mathbf{R_f}$ =0.62 (75/25 pentane/EtOAc). **^{1}H-NMR** (400 MHz, $CDCl_3$, 25 °C): δ 4.58 (t, J=5.6 Hz, 1H, H-4), 3.39 (s, 6H, H-5, H-6), 2.56 (dd, J=5.6, 2.7 Hz, 2H, H-3), 2.06 (t, J=2.7 Hz, 1H, H-1) ppm. **^{13}C-NMR** (100 MHz, $CDCl_3$, 25 °C): δ 102.3 (C-4), 79.3 (C-2), 70.0 (C-1), 53.5 (×2, C-5, C-6), 23.7 (C-3) ppm.

Compound S-4a

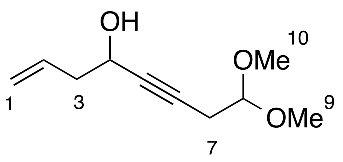

Dess-Martin periodinane (5.0 g, 12 mmol, 1.1 equiv) was added to a solution of 3-buten-1-ol **S-3a** (0.92 mL, 11 mmol, 1.0 equiv) in Et_2O (100 mL) at room temperature. After 1 hour, stirring was stopped and the suspension let to settle. The clear supernatant was transferred to a solution of alkyne **4** (6.1 g, 53 mmol, 5.0 equiv) and *n*-BuLi (26 mL, 1.6 M in hexanes, 43 mmol, 4.0 equiv) in Et_2O (150 mL) at -78 °C. The resulting mixture was stirred for 1 hour at this temperature. The reaction was quenched by addition of sat. aqueous $NaHCO_3$ and the aqueous phase was extracted with Et_2O. The collected organic layers were washed with brine, dried over Na_2SO_4 and filtered. Evaporation of the solvent under reduced pressure followed by flash chromatography (SiO_2, 90/10 to 80/20 pentane/Et_2O) yielded alcohol **S-4a** as a yellow oil (884.4 mg, 4.8 mmol, 45%). $\mathbf{R_f}$=0.30 (75/25 petroleum ether/EtOAc). **^{1}H-NMR** (400 MHz, $CDCl_3$, 25 °C): *δ* 5.94-5.84 (m, 1H, H-2), 5.19 (d, *J*=17.1 Hz, 1H, H-1), 5.18 (d, *J*=10.5 Hz, 1H, H-1), 4.52 (t, *J*=5.7 Hz, 1H, H-8), 4.44-4.40 (m, 1H, H-4), 3.37 (s, 6H, H-9, H-10), 2.55 (dd, *J*=5.7, 2.0 Hz, 2H, H-7), 2.48-2.44 (m, 2H, H-3) ppm (OH signal not visible). **^{13}C-NMR** (100 MHz, $CDCl_3$, 25 °C): *δ* 133.2 (C-2), 118.6 (C-1), 102.4 (C-8), 82.4 (C-5), 80.4 (C-6), 61.6 (C-4), 53.3 (C-9, C-10), 42.2 (C-3), 23.9 (C-7) ppm.

Compound S-4b

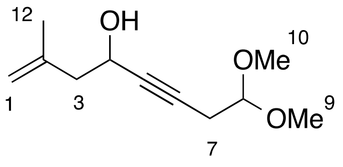

Dess-Martin periodinane (12.7 g, 30 mmol, 1.0 equiv) was added to a solution of 3-methyl-3-buten-1-ol **S-3b** (3.0 mL, 30 mmol, 1.0 equiv) in Et_2O (250 mL) at room temperature. After 1 hour, stirring was stopped and the suspension let to settle. The clear supernatant was transferred *via* cannula to a solution of alkyne **4** (17.1 g, 0.15 mol, 5.0 equiv) and *n*-BuLi (75 mL, 1.6 M in hexanes, 0.12 mol, 4.0 equiv) in Et_2O (300 mL) at -78 °C. The resulting mixture was then warmed up to room temperature. After 2 hours, the reaction was quenched with water and the aqueous phase was extracted with Et_2O. The collected organic layers were washed with brine, dried over Na_2SO_4 and filtered. Evaporation of the solvent under

reduced pressure followed by flash chromatography (SiO_2, 90/10 to 50/50 pentane/Et_2O) yielded alcohol **S-4b** as a yellow oil (4.26 g, 21 mmol, 72%). **R_f**=0.29 (75/25 petroleum ether/EtOAc). **^{1}H-NMR** (400 MHz, $CDCl_3$, 25 °C): δ 4.90 (s, 1H, H-1), 4.84 (s, 1H, H-1), 4.52 (t, *J*=5.8 Hz, 1H, H-8), 4.52-4.48 (m, 1H, H-4), 3.36 (s, 6H, H-9, H-10), 2.55 (dd, *J*=5.8, 2.0 Hz, 2H, H-7), 2.43 (d, *J*=6.8 Hz, 2H, H-3), 1.79 (s, 3H, H-12) ppm (OH signal not visible). **^{13}C-NMR** (100 MHz, $CDCl_3$, 25 °C): δ 141.2 (C-2), 114.2 (C-1), 102.4 (C-8), 82.6 (C-6), 80.2 (C-5), 60.5 (C-4), 53.4 C-9, C-10), 46.2 (C-3), 24.0 (C-7), 22.6 (C-12) ppm.

Compound 5a

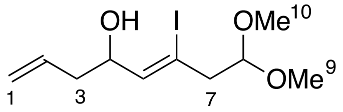

Following general procedure A, alcohol **S-4a** (864.4 mg, 4.7 mmol, 1.0 equiv) gave, after purification by flash chromatography (SiO_2, 80/20 pentane/EtOAc), alcohol **5a** as a yellow oil (1.07 g, 3.4 mmol, 73%). **^{1}H-NMR** (400 MHz, $CDCl_3$, 25 °C): δ 5.89-5.79 (m, 1H, H-2), 5.75 (d, *J*=7.5 Hz, 1H, H-5), 5.17 (d, *J*=17.1 Hz, 1H, H-1), 5.16 (d, *J*=10.5 Hz, 1H, H-1), 4.66 (t, *J*=5.7 Hz, 1H, H-8), 4.38-4.33 (m, 1H, H-4), 3.36 (s, 3H, H-9), 3.35 (s, 3H, H-10), 2.83-2.80 (m, 2H, H-7), 2.44-2.29 (m, 2H, H-3), 1.93 (bs, 1H, OH) ppm. **^{13}C-NMR** (100 MHz, $CDCl_3$, 25 °C): δ 139.7 (C-5), 133.5 (C-2), 118.6 (C-1), 103.0 (C-8), 101.2 (C-6), 75.4 (C-4), 53.5 (×2, C-9, C-10), 48.1 (C-3), 40.5 (C-7) ppm.

Compound 5b

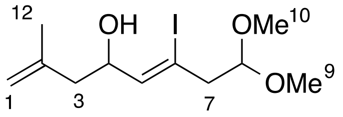

Following general procedure A, alcohol **S-4b** (8.2 g, 41 mmol, 1.0 equiv) gave, after purification by flash chromatography (SiO_2, 80/20 pentane/EtOAc), alcohol **5b** as a yellow oil (11.5 g, 35 mmol, 85%). **R_f**=0.32 (75/25 petroleum ether/EtOAc). **^{1}H-NMR** (400 MHz, $CDCl_3$, 25 °C): δ 5.74 (d, *J*=7.3 Hz, 1H, H-5), 4.90 (s, 1H, H-1), 4.83 (s, 1H, H-1), 4.65 (t, *J*=5.6 Hz, 1H, H-8), 4.47-4.38 (m, 1H, H-4), 3.35 (s, 3H, H-9), 3.34 (s, 3H, H-10), 2.86-2.76 (m, 2H, H-7), 2.34-2.21 (m, 2H, H-3), 1.82 (s, 3H, H-12) ppm (OH signal not visible). **^{13}C-NMR** (100 MHz, $CDCl_3$, 25 °C): δ 141.5 (C-2), 139.9 (C-5), 114.2 (C-1), 103.1 (C-8), 100.9 (C-6), 74.0 (C-4), 53.5 (×2, C-9, C-10), 48.1 (C-3), 44.4 (C-7), 22.4 (C-12) ppm.

Compound 6a

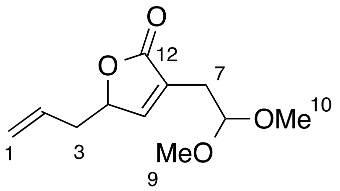

Following general procedure B, alcohol **5a** (1.07 g, 3.4 mmol, 1.0 equiv) gave, after purification by flash chromatography (SiO_2, 95/5 to 50/50 pentane/EtOAc), lactone **6a** as a yellow oil (228.4 mg, 1.07 mmol, 31%). **R_f**=0.38 (75/25 petroleum ether/EtOAc). **^{1}H-NMR** (400 MHz, $CDCl_3$, 25 °C): δ 7.19 (q,

J=1.6 Hz, 1H, H-5), 5.75 (ddt, J=17.0, 10.3, 7.0 Hz, 1H, H-2), 5.18 (d, J=17.0 Hz, 1H, H-1), 5.17 (d, J=10.3 Hz, 1H, H-1), 4.97 (tq, J=6.4, 1.6 Hz, 1H, H-4), 4.63 (t, J=5.7 Hz, 1H, H-8), 3.35 (s, 6H, H-9, H-10), 2.61 (dt, J=5.7, 1.6 Hz, 2H, H-7), 2.54-2.42 (m, 2H, H-3) ppm. **^{13}C-NMR** (100 MHz, $CDCl_3$, 25 °C): δ 150.0 (C-5), 131.2 (C-2), 129.8 (C-6), 119.4 (C-1), 102.1 (C-8), 80.5 (C-4), 53.3 (×2, O-CH_3), 37.5 (C-3), 29.0 (C-7) ppm (one quaternary carbon not visible).

Compound 6b

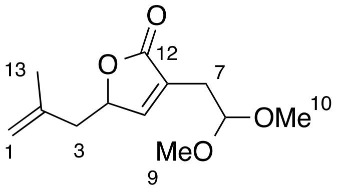

Following general procedure B, alcohol **5b** (200.0 mg, 0.61 mmol, 1.0 equiv) gave, after purification by flash chromatography (SiO_2, 95/5 to 50/50 pentane/EtOAc), lactone **6b** as a yellow oil (62 mg, 0.27 mmol, 45%). **R_f**=0.63 (50/50 petroleum ether/EtOAc). **^{1}H-NMR** (400 MHz, $CDCl_3$, 25 °C): δ 7.21 (q, J=1.6 Hz, 1H, H-5), 5.06 (ddq, J=7.2, 6.9, 1.6 Hz, 1H, H-4), 4.91 (s, 1H, H-1), 4.82 (s, 1H, H-1), 4.63 (t, J=5.7 Hz, 1H, H-8), 3.35 (s, 6H, H-9, H-10), 2.61 (dt, J=5.7, 1.6 Hz, 2H, H-7), 2.43 (dd, J=13.9, 7.2 Hz, 1H, H-3), 2.34 (dd, J=13.9, 6.9 Hz, 1H, H-3), 1.80 (s, 3H, H-13) ppm. **^{13}C-NMR** (100 MHz, $CDCl_3$, 25 °C): δ 173.5 (C-12), 150.4 (C-5), 139.6 (C-2), 129.3 (C-6), 114.3 (C-1), 102.1 (C-8), 80.0 (C-4), 53.3 (×2, C-9, C-10), 41.5 (C-3), 28.9 (C-7), 23.0 (C-13) ppm. **LC-MS (ESI$^+$)**: m/z calculated for $C_{12}H_{19}O_4^+$ $[M+H]^+$: 227.12; found 226.83.

Compound S-5a

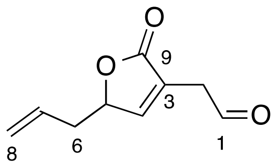

$FeCl_3$-$6H_2O$ (760 mg, 2.8 mmol, 3.5 equiv) was added to a solution of acetal **6a** (213 mg, 1.0 mmol, 1.0 equiv) in CH_2Cl_2 (50 mL) at room temperature. The yellow mixture was stirred for 30 minutes. The reaction was quenched with sat. aqueous $NaHCO_3$ and the aqueous phase was extracted with CH_2Cl_2. The combined organic layers were washed with brine, dried over Na_2SO_4 and filtered. Evaporation of the solvent under reduced pressure yielded a mixture of acetal **6a** and aldehyde **S-5a** which was re-submitted to the same conditions. Evaporation under reduced pressure afforded aldehyde **S-5a** as a pale yellow oil (119 mg, 0.72 mmol, 72%), which was used for the next step without further purification. **R_f**=0.59 (50/50 petroleum ether/EtOAc). **^{1}H-NMR** (400 MHz, C_6D_6, 25 °C): δ 9.02 (t, J=1.2 Hz, 1H, H-1), 6.42 (q, J=1.5 Hz, 1H, H-4), 5.39 (ddt, J=17.1, 10.3, 7.0 Hz, 1H, H-7), 4.91 (bd, J=10.3 Hz, 1H, H-8), 4.85 (dq, J=17.1, 1.6 Hz, 1H, H-8), 4.18 (tq, J=6.4, 1.6 Hz, 1H, H-5), 2.69 (q, J=1.2 Hz, 2H, H-2), 1.90-1.85 (m, 2H, H-6) ppm. **LC-MS (ESI$^+$)**: m/z calculated for $C_9H_{11}O_3^+$ $[M+H]^+$: 167.06; found 166.91.

Compound S-5b

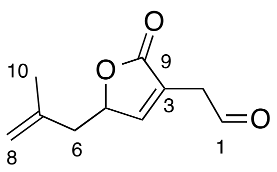

$FeCl_3$-$6H_2O$ (970 mg, 3.6 mmol, 3.5 equiv) was added to a solution of acetal **6b** (232 mg, 1.0 mmol, 1.0 equiv) in CH_2Cl_2 (10 mL) at room temperature. The yellow mixture was stirred for 30 minutes. The reaction was quenched with sat. aqueous $NaHCO_3$ and the aqueous phase was extracted with CH_2Cl_2. The combined organic layers were washed with brine, dried over Na_2SO_4 and filtered. Evaporation of the solvent under reduced pressure yielded a mixture of acetal **6b** and aldehyde **S-5b**, which was re-submitted to the same conditions. Evaporation under reduced pressure afforded aldehyde **S-5b** as a pale yellow oil (110 mg, 0.61 mmol, 60%), which was used for the next step without further purification. **R_f**=0.40 (50/50 petroleum ether/EtOAc). **^{1}H-NMR** (400 MHz, C_6D_6, 25 °C): δ 9.79 (t, J=1.0 Hz, 1H, H-1), 7.45 (q, J=1.5 Hz, 1H, H-4), 5.18-5.13 (m, 1H, H-5), 4.94 (s, 1H, H-8), 4.84 (s, 1H, H-8), 3.50 (bs, 2H, H-2), 2.52-2.36 (m, 2H, H-6), 1.82 (s, 3H, H-10) ppm.

Asymmetric synthesis of butenolide 6

Compound S-6

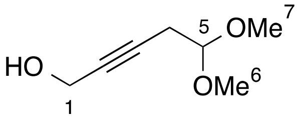

A solution of homopropargyl dimethyl acetal **4** (21.1 g, 185 mmol, 1 equiv) in dry THF (650 mL, 0.3 M) cooled to −78 °C was treated to a slow addition *n*BuLi (120 mL, 1.6 M in hexanes, 192 mmol, 1.04 equiv). After stirring for 30 minutes, paraformaldehyde (15.6 g, 520 mmol HCHO, 2.8 equiv HCHO) was added as a solid, and the resulting mixture was stirred for one hour while slowly warming up to room temperature until the starting material had disappeared. The reaction mixture was quenched with water, diluted with EtOAc, and the aqueous layer was separated and extracted with EtOAc. The combined organic layers were washed with brine, dried over Na_2SO_4, filtered on silica and concentrated *in vacuo*. Purification by flash chromatography on silica gel (cyclohexane/EtOAc 10:1 to 1:1) afforded propargyl alcohol **S-6** as a yellow oil (26.6 g, 185 mmol, quant.). $\mathbf{R_f}$=0.41 (cyclohexane/EtOAc 2:1). **^{1}H-NMR** (400 MHz, $CDCl_3$, 25 °C): δ 4.53 (t, *J* = 5.6 Hz, 1H, H-5), 4.31-4.23 (m, 2H, H-1), 3.37 (s, 6H, H-6, H-7), 2.56 (dt, *J* = 5.6, 2.2 Hz, 2H, H-4) ppm.

Compound S-7

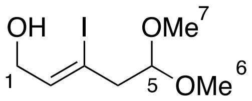

A solution of propargyl alcohol **S-6** (14.4g, 100 mmol, 1 equiv) in dry THF (500 mL, 0.2 M) and cooled at 0 °C was treated with a solution of $LiAlH_4$ (100 mL, 2.4 M in THF, 240 mmol, 2.4 equiv). The reaction mixture was then refluxed for 3 hours. After cooling to 0 °C, dry EtOAc (100 mL) was slowly added. Stirring was continued for 30 minutes, and a solution of I_2 (76 g, mmol, equiv) in dry THF was added. After stirring for 3 hours, the reaction was quenched with a 1:1 mixture of saturated aqueous $NaHCO_3$ and saturated aqueous Na_2SO_3. The organic layer was separated and the aqueous layer was extracted with EtOAc. The combined organic layers were washed with water and brine, dried over Na_2SO_4, filtered on silica and concentrated *in vacuo*. The residue was purified by flash chromatography on silica gel (cyclohexane/EtOAc 10:1 to 1:1) to afford vinyl iodide **S-7** as a yellow oil (16.1 g, 59 mmol, 59%). $\mathbf{R_f}$=0.39 (cyclohexane/EtOAc 2:1). **^{1}H-NMR** (400 MHz, $CDCl_3$, 25 °C): δ 5.99 (t, *J* = 5.7 Hz, 1H, H-2), 4.68 (t, *J* = 5.7 Hz, 2H, H-1), 4.22 (d, *J* = 5.0 Hz, 2H, H-5), 3.37 (s, 6H, H-6, H-7), 2.84 (d, *J* = 1.1 Hz, 2H, H-4) ppm. **^{13}C-NMR** (100 MHz, $CDCl_3$, 25 °C): δ 136.8 (C-2), 103.1 (C-5), 67.3 (C-1), 53.5 (C-6, C-7), 47.9 (C-4) ppm.

Compound 13

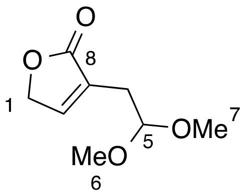

A solution of vinyl iodide **S-7** (16.1 g, 59 mmol, 1 equiv) and triethylamine (16.6 mL, 118 mmol, 2 equiv) in acetonitrile (590 mL, 0.1 M) was degassed by freeze-pump-thaw cycles and the atmosphere replaced with CO (balloon). The mixture was warming up to room temperature, $Pd(PPh_3)_4$ (681 mg, 0.59 mmol, 0.01 equiv) was added in one portion and the reaction mixture was refluxed overnight. Upon complete consumption of the starting material, the reaction was cooled down to room temperature and poured into brine. The organic layer was separated and the aqueous layer was extracted with EtOAc. The combined organic layers were washed with brine, dried over Na_2SO_4, filtered on silica and concentrated *in vacuo*. The residue was purified by flash chromatography on silica gel (cyclohexane/EtOAc 10:1 to 1:1) to afford butenolide **13** as a yellow oil (8.9 g, 52 mmol, 88%). $\mathbf{R_f}$=0.35 (cyclohexane/EtOAc 2:1). **^{1}H-NMR** (400 MHz, $CDCl_3$, 25 °C): δ 7.30 (p, *J* = 1.7 Hz, 1H, H-2), 4.80 (q, *J* = 1.7 Hz, 2H, H-1), 4.64 (t, *J* = 5.6 Hz, 1H, H-5), 3.36 (s, 6H, H-6, H-7), 2.63 (dq, *J* = 5.4, 1.7 Hz, 2H, H-4) ppm. **^{13}C-NMR** (100 MHz, $CDCl_3$, 25 °C): δ 174.3 (C-8), 146.9 (C-2), 129.2 (C-3), 102.1 (C-5), 70.4 (C-1), 53.3 (C-6, C-7), 28.9 (C-4) ppm.

Compound 14

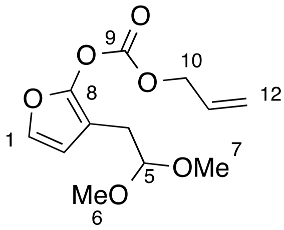

To a solution of butenolide **13** (1.84 g, 10.7 mmol, 1 equiv) in THF (5 mL) was slowly added to a solution of NaHMDS (13 mL, 1 M in THF, 13 mmol, 1.2 equiv) in dry THF (50 mL, M) cooled to −60 °C. After stirring for 30 min, neat allyl chloroformate (3.4 mL, 32 mmol, 3.0 equiv) was rapidly added, and the reaction mixture was stirred for 30 min. The reaction mixture was quenched with water, dried over Na_2SO_4, filtered on silica and concentrated *in vacuo*. The residue was purified by flash chromatography on silica gel (cyclohexane/EtOAc 10:1 to 1:1) to afford allyl carbonate **14** as a pale orange oil (2.66 g, 10.4 mmol, 97%). $\mathbf{R_f}$=0.62 (cyclohexane/EtOAc 2:1). **^{1}H-NMR** (400 MHz, $CDCl_3$, 25 °C): δ 7.04 (d, *J* = 2.2 Hz, 1H, H-2), 6.34 (d, *J* = 2.2 Hz, 1H, H-1), 5.98 (ddt, *J* = 16.9, 10.4, 5.9 Hz, 1H, H-11), 5.43 (dd, *J* = 16.9, 1.4 Hz, 1H, H-12), 5.35 (dt, *J* = 10.2, 1.1 Hz, 1H, H-12), 4.75 (dt, *J* = 5.9, 1.4 Hz, 2H, H-10), 4.45 (t, *J* = 5.6 Hz, 1H, H-5), 3.34 (s, 6H, H-6, H-7), 2.61 (d, *J* = 5.6 Hz, 2H, H-4) ppm. **^{13}C-NMR** (100 MHz, $CDCl_3$, 25 °C): δ 152.0 (C-9), 147.5 (C-8), 135.8 (C-2), 130.5 (C-11), 120.0 (C-12), 113.1 (C-1), 103.8 (C-5), 102.2 (C-3), 70.0 (C-10), 53.3 (C-6, C-7), 27.5 (C-4) ppm.

Compound *rac*-6 via Pd-catalysed AAA/[3,3]-Cope rearrangement

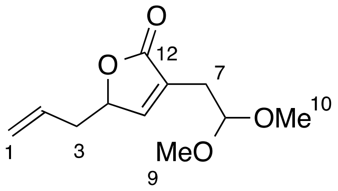

A solution of allyl carbonate **14** (723 mg, 2.8 mmol, 1 equiv) in toluene (15 mL) in a microwave vial was added Pd(PPh_3)$_4$ (60 mg, 56 μmol, 0.02 equiv) portionwise, leading to the rapid evolution of CO_2. Stirring was continued for 15 min until complete consumption of the starting material. The vial was then sealed and the reaction mixture irradiated under microwaves at 180 °C for 30 min. The reaction mixture was filtered on silica and concentrated *in vacuo*. The residue was purified by flash chromatography on silica gel (cyclohexane/EtOAc 10:1 to 1:1) to afford butenolide ***rac*-6** as a pale yellow oil (534 mg, 2.52 mmol, 90%). The NMR data was in agreement with those obtained for **6** using a different synthetic approach.

Compound (*R*)-6 via Pd-catalysed AAA/[3,3]-Cope rearrangement

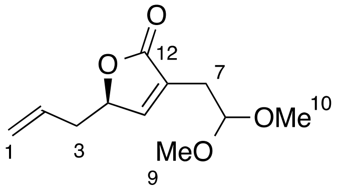

Starting with allyl carbonate **14** (1.15 g, 4.5 mmol) and using general procedure C, butenolide **(*R*)-6** was obtained as a pale yellow oil (575 mg, 2.71 mmol, 60%) in 92:8 er (84% ee). The NMR data was in agreement with those obtained for **6** using a different synthetic approach. α_D^{25} -31.1° (*c*=0.50, $CHCl_3$).

Compound (*S*)-6 via Pd-catalysed AAA/[3,3]-Cope rearrangement

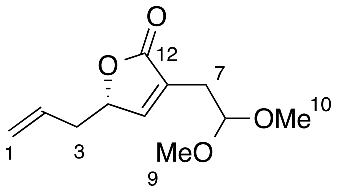

Starting with allyl carbonate **14** (1.15 g, 4.5 mmol) and using general procedure C, butenolide **(*S*)-6** was obtained as a pale yellow oil (561 mg, 2.65 mmol, 59%) in 8:92 er (84% ee). The NMR data was in agreement with those obtained for **6** using a different synthetic approach. α_D^{25} +31.1° (*c*=0.50, $CHCl_3$).

Synthesis of Nordeoxyelephantopins and compounds 11 and 12

Compound *rac*-9

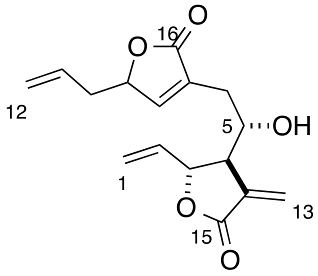

Following general procedure D with acetal ***rac*-6** (212 mg, 1 mmol) and bromolactone **2**, alcohol ***rac*-9** was obtained as a mixture of diastereomers as a pale yellow oil (65 mg, 0.23 mmol, 23%). $\mathbf{R_f}$=0.22 (cyclohexane/EtOAc 1:1). **^{1}H-NMR** (400 MHz, $CDCl_3$, 25 °C): δ 7.23 (bs, 1H, H-8), 6.41 (bs, 1H, H-13), 5.90-5.65 (m, 2H, 11, H-2), 5.82 (bs, 1H, H-13), 5.38 (d, *J*=17.0 Hz, 1H, H-1), 5.24 (d, *J*=10.5 Hz, 1H, H-1), 5.19 (d, *J*=16.7 Hz, 1H, H-12), 5.18 (d, *J*=10.7 Hz, 1H, H-12), 5.06-5.00 (m, 1H, H-9), 4.94 (bs, 1H, H-3), 4.05-3.98 (m, 1H, H-5), 3.07-2.88 (m, 1H, H-4), 2.56-2.47 (m, 4H, H-10, H-6) ppm (OH signal not visible). **^{13}C-NMR** (100 MHz, $CDCl_3$, 25 °C): δ 174.6 (C-15), 169.6 (C-16), 151.5 (C-8), 151.4 (C-8), 135.7 (C-2), 135.6 (C-2), 134.3 (C-14), 131.0 (C-7), 130.8 (C-11), 125.7 (C-13), 119.9 (C-12), 119.8 (C-12), 117.7 (C-1), 81.1 (C-9), 80.7 (C-9), 79.3 (C-3), 71.0 (C-5), 50.4 (C-4), 37.4 (C-10), 37.3 (C-10), 30.2 (C-6) ppm. **LC-MS (ESI^{+})**: m/z calculated for $C_{16}H_{19}O_5^{+}$ $[M+H]^{+}$: 291.12; found 290.77.

Compound (*R*)-9

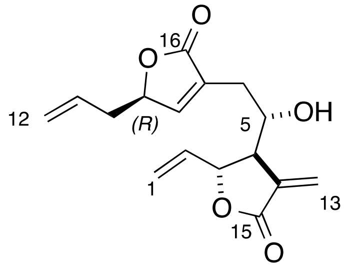

Following general procedure D with acetal **(*R*)-6** (212 mg, 1 mmol), alcohol **(*R*)-9** was obtained as a mixture of diastereomers as a pale yellow oil (66 mg, 0.23 mmol, 23%). $\mathbf{R_f}$=0.22 (cyclohexane/EtOAc 1:1). **^{1}H-NMR** (400 MHz, $CDCl_3$, 25 °C): δ 7.23 (bs, 1H, H-8), 6.41 (bs, 1H, H-13), 5.90-5.65 (m, 2H, 11, H-2), 5.82 (bs, 1H, H-13), 5.38 (d, *J*=17.0 Hz, 1H, H-1), 5.24 (d, *J*=10.5 Hz, 1H, H-1), 5.19 (d, *J*=16.7 Hz, 1H, H-12), 5.18 (d, *J*=10.7 Hz, 1H, H-12), 5.06-5.00 (m, 1H, H-9), 4.94 (bs, 1H, H-3), 4.05-3.98 (m, 1H, H-5), 3.07-2.88 (m, 1H, H-4), 2.56-2.47 (m, 4H, H-10, H-6) ppm (OH signal not visible). **^{13}C-NMR** (100 MHz, $CDCl_3$, 25 °C): δ 174.6 (C-15), 169.6 (C-16), 151.5 (C-8), 151.4 (C-8), 135.7 (C-2), 135.6 (C-2), 134.3 (C-14), 131.0 (C-7), 130.8 (C-11), 125.7 (C-13), 119.9 (C-12), 119.8 (C-12), 117.7 (C-1), 81.1 (C-9), 80.7 (C-9), 79.3 (C-3), 71.0 (C-5), 50.4 (C-4), 37.4 (C-10), 37.3 (C-10), 30.2 (C-6) ppm. **LC-MS (ESI^{+})**: m/z calculated for $C_{16}H_{19}O_5^{+}$ $[M+H]^{+}$: 291.12; found 290.81.

Compound (*S*)-9

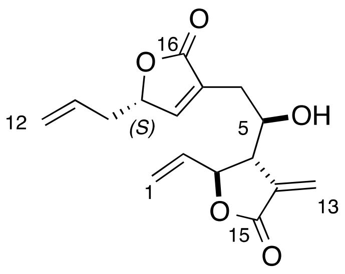

Following general procedure D with acetal **(*S*)-6** (212 mg, 1 mmol), alcohol **(*S*)-9** was obtained as a mixture of diastereomers as a pale yellow oil (68 mg, 0.24 mmol, 24%). **R_f**=0.22 (cyclohexane/EtOAc 1:1). **^{1}H-NMR** (400 MHz, $CDCl_3$, 25 °C): δ 7.23 (bs, 1H, H-8), 6.41 (bs, 1H, H-13), 5.90-5.65 (m, 2H, 11, H-2), 5.82 (bs, 1H, H-13), 5.38 (d, *J*=17.0 Hz, 1H, H-1), 5.24 (d, *J*=10.5 Hz, 1H, H-1), 5.19 (d, *J*=16.7 Hz, 1H, H-12), 5.18 (d, *J*=10.7 Hz, 1H, H-12), 5.06-5.00 (m, 1H, H-9), 4.94 (bs, 1H, H-3), 4.05-3.98 (m, 1H, H-5), 3.07-2.88 (m, 1H, H-4), 2.56-2.47 (m, 4H, H-10, H-6) ppm (OH signal not visible). **^{13}C-NMR** (100 MHz, $CDCl_3$, 25 °C): δ 174.6 (C-15), 169.6 (C-16), 151.5 (C-8), 151.4 (C-8), 135.7 (C-2), 135.6 (C-2), 134.3 (C-14), 131.0 (C-7), 130.8 (C-11), 125.7 (C-13), 119.9 (C-12), 119.8 (C-12), 117.7 (C-1), 81.1 (C-9), 80.7 (C-9), 79.3 (C-3), 71.0 (C-5), 50.4 (C-4), 37.4 (C-10), 37.3 (C-10), 30.2 (C-6) ppm. **LC-MS (ESI$^+$)**: m/z calculated for $C_{16}H_{19}O_5^+$ $[M+H]^+$: 291.12; found 290.83.

Compound (*R*)-10

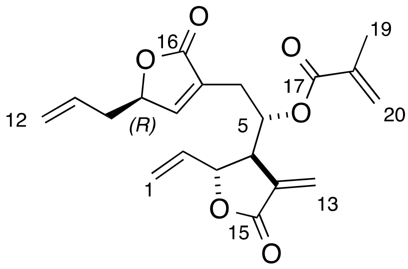

Following general procedure E with alcohol **(*R*)-9** (40 mg, 150 µmol), methacrylate **(*R*)-10** was obtained as a mixture of diastereomers as a pale yellow oil (34 mg, 94 µmol, 63%). **R_f**=0.39 (cyclohexane/EtOAc 1:1). **^{1}H-NMR** (400 MHz, $CDCl_3$, 25 °C): δ 7.14 (bs, 0.5H, H-8), 7.12 (bs, 0.5H, H-8), 6.46 (bs, 1H, H-13), 6.07 (bs, 1H, H-20), 5.90 (bs, 1H, H-13), 5.83-5.64 (m, 2H, H-11, H-2), 5.61 (bs, 1H, H-20), 5.46-5.40 (m, 1H, H-5), 5.35 (d, *J*=16.9 Hz, 1H, H-1), 5.24 (d, *J*=10.3 Hz, 1H, H-1), 5.17 (d, *J*=16.9 Hz, 1H, H-12), 5.14 (d, *J*=10.3 Hz, 1H, H-12), 4.96-4.92 (m, 1H, H-9), 4.89-4.88 (m, 1H, H-3), 3.14 (bs, 1H, H-4), 2.73-2.61 (m, 2H, H-6), 2.47-2.37 (m, 2H, H-10), 1.89 (s, 3H, H-19) ppm. **^{13}C-NMR** (100 MHz, $CDCl_3$, 25 °C): δ 172.9 (C-15), 172.8 (C-15), 169.3 (C-16), 169.2 (C-16), 166.4 (C-17), 166.3 (C-17), 150.5 (C-8), 150.4 (C-8), 135.3 (C-18), 135.0 (C-2), 133.3 (C-14), 133.2 (C-14), 130.9 (C-11), 130.7 (C-11), 129.9 (C-7), 127.1 (C-20), 126.2 (C-13), 119.7 (C-12), 119.6 (C-12), 117.8 (C-1), 80.5 (C-9), 79.4 (C-3), 79.3 (C-3), 72.5 (C-5), 72.4 (C-5), 48.0 (C-4), 37.3 (C-10), 37.2 (C-10), 26.9 (C-6), 26.7 (C-6), 18.2 (C-19) ppm. **HR-MS (+ESI)** $[M+Na]^+$: calcd: 381.1309, found: 381.1309.

Compound (*S*)-10

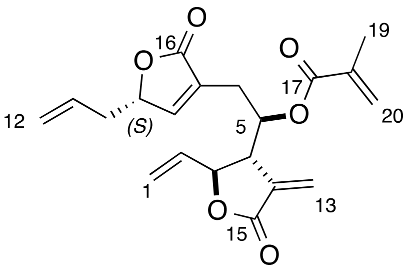

Following general procedure E with alcohol **(*S*)-9** (40 mg, 150 μmol, 1 equiv), methacrylate **(*S*)-10** was obtained as a mixture of diastereomers as a pale yellow oil (30 mg, 93 μmol, 62%). **R$_f$**=0.39 (cyclohexane/EtOAc 1:1). **^{1}H-NMR** (400 MHz, $CDCl_3$, 25 °C): δ 7.14 (bs, 0.5H, H-8), 7.12 (bs, 0.5H, H-8), 6.46 (bs, 1H, H-13), 6.07 (bs, 1H, H-20), 5.90 (bs, 1H, H-13), 5.83-5.64 (m, 2H, H-11, H-2), 5.61 (bs, 1H, H-20), 5.46-5.40 (m, 1H, H-5), 5.35 (d, *J*=16.9 Hz, 1H, H-1), 5.24 (d, *J*=10.3 Hz, 1H, H-1), 5.17 (d, *J*=16.9 Hz, 1H, H-12), 5.14 (d, *J*=10.3 Hz, 1H, H-12), 4.96-4.92 (m, 1H, H-9), 4.89-4.88 (m, 1H, H-3), 3.14 (bs, 1H, H-4), 2.73-2.61 (m, 2H, H-6), 2.47-2.37 (m, 2H, H-10), 1.89 (s, 3H, H-19) ppm. **^{13}C-NMR** (100 MHz, $CDCl_3$, 25 °C): δ 172.9 (C-15), 172.8 (C-15), 169.3 (C-16), 169.2 (C-16), 166.4 (C-17), 166.3 (C-17), 150.5 (C-8), 150.4 (C-8), 135.3 (C-18), 135.0 (C-2), 133.3 (C-14), 133.2 (C-14), 130.9 (C-11), 130.7 (C-11), 129.9 (C-7), 127.1 (C-20), 126.2 (C-13), 119.7 (C-12), 119.6 (C-12), 117.8 (C-1), 80.5 (C-9), 79.4 (C-3), 79.3 (C-3), 72.5 (C-5), 72.4 (C-5), 48.0 (C-4), 37.3 (C-10), 37.2 (C-10), 26.9 (C-6), 26.7 (C-6), 18.2 (C-19) ppm. **HR-MS (+ESI)** $[M+Na]^+$: calcd: 381.1309, found: 381.1309.

Nordeoxyelephantopin

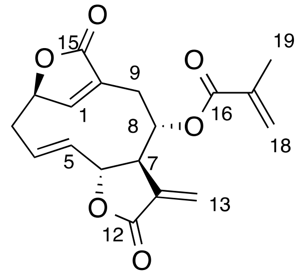

Following general procedure F with diene **(*R*)-10** (45 mg, 126 μmol), **Nordeoxyelephantopin** was obtained as a mixture of diastereomers as a pale yellow oil (3.2 mg, 9.7 μmol, 32% based on recovered starting material and reacted diastereomer). **R$_f$**=0.31 (cyclohexane/EtOAc 1:2). **R$_t$**=7.9 min (HPLC reverse phase MeCN in H_2O, 30% to 90%, 3 mL/min). **^{1}H-NMR** (500 MHz, $CDCl_3$, 25 °C): δ 7.00 (s, 1H, H-1), 6.25 (d, *J*=3.7 Hz, 1H, H-13), 6.12 (bs, 1H, H-18), 5.63 (quint, *J*=1.4 Hz, 1H, H-18), 5.62 (d, *J*=3.4 Hz, 1H, H-13), 5.47 (ddd, *J*=16.1, 10.7, 4.3 Hz, 1H, H-4), 5.38-5.36 (m, 1H, H-2), 5.28 (dd, *J*=16.1, 9.6 Hz, 1H, H-5), 4.89 (dd, *J*=9.6, 7.7 Hz, 1H, H-6), 4.64 (ddd, *J*=11.6, 3.4, 2.0 Hz, 1H, H-8), 3.02 (bdd, *J*=12.9, 2.0 Hz, 1H, H-9), 2.97 (dt, *J*=13.3, 4.3 Hz, 1H, H-3), 2.91 (dq, *J*=7.7, 3.4 Hz, H-7), 2.75 (dd, *J*=12.9, 11.6 Hz, 1H, H-9), 2.47 (ddd, *J*=13.3, 10.7, 2.2 Hz, 1H, H-3), 1.92 (s, 3H, H-19) ppm. **^{13}C-NMR** (125 MHz, $CDCl_3$, 25 °C): δ 172.3 (C-15), 169.0 (C-12), 166.4 (C-16), 152.8 (C-1), 139.4 (C-5), 135.7 (C-17), 133.6 (C-11), 130.0 (C-10), 126.6 (C-18), 125.8 (C-6), 124.2 (C-13), 82.2 (C-6), 80.4 (C-2), 71.6 (C-8), 51.1 (C-7), 34.2 (C-3), 33.6 (C-9), 18.2 (C-19) ppm. (C-14 and H-14 omitted to

respect the original assignment for Deoxyelephantopin). **HR-MS (+ESI)** [M+Na]$^+$: calcd: 353.0996, found: 353.0990.

ent-Nordeoxyelephantopin

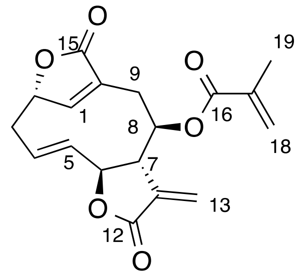

Following general procedure F with diene **(*S*)-10** (40 mg, 112 mmol), ***ent*-Nordeoxyelephantopin** was obtained as a mixture of diastereomers as a pale yellow oil (2.9 mg, 8.8 μmol, 33% based on recovered starting material and reacted diastereomer). **R$_f$**=0.31 (cyclohexane/EtOAc 1:2). **R$_t$**=7.9 min (HPLC reverse phase MeCN in H_2O, 30% to 90%, 3 mL/min). **^{1}H-NMR** (500 MHz, $CDCl_3$, 25 °C): δ 7.00 (s, 1H, H-1), 6.25 (d, *J*=3.7 Hz, 1H, H-13), 6.12 (bs, 1H, H-18), 5.63 (quint, *J*=1.4 Hz, 1H, H-18), 5.62 (d, *J*=3.4 Hz, 1H, H-13), 5.47 (ddd, *J*=16.1, 10.7, 4.3 Hz, 1H, H-4), 5.38-5.36 (m, 1H, H-2), 5.28 (dd, *J*=16.1, 9.6 Hz, 1H, H-5), 4.89 (dd, *J*=9.6, 7.7 Hz, 1H, H-6), 4.64 (ddd, *J*=11.6, 3.4, 2.0 Hz, 1H, H-8), 3.02 (bdd, *J*=12.9, 2.0 Hz, 1H, H-9), 2.97 (dt, *J*=13.3, 4.3 Hz, 1H, H-3), 2.91 (dq, *J*=7.7, 3.4 Hz, H-7), 2.75 (dd, *J*=12.9, 11.6 Hz, 1H, H-9), 2.47 (ddd, *J*=13.3, 10.7, 2.2 Hz, 1H, H-3), 1.92 (s, 3H, H-19) ppm. **^{13}C-NMR** (125 MHz, $CDCl_3$, 25 °C): δ 172.3 (C-15), 169.0 (C-12), 166.4 (C-16), 152.8 (C-1), 139.4 (C-5), 135.7 (C-17), 133.6 (C-11), 130.0 (C-10), 126.6 (C-18), 125.8 (C-6), 124.2 (C-13), 82.2 (C-6), 80.4 (C-2), 71.6 (C-8), 51.1 (C-7), 34.2 (C-3), 33.6 (C-9), 18.2 (C-19) ppm. (C-14 and H-14 omitted to respect the original assignment for Deoxyelephantopin). **HR-MS (+ESI)** [M+Na]$^+$: calcd: 353.0996, found: 353.0990.

Compound S-8

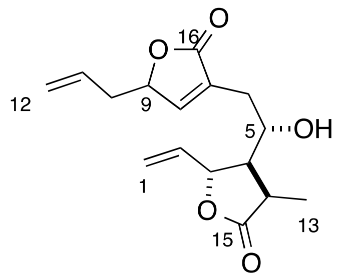

To a solution of olefin **9** (87 g, 0.3 mmol, 1 equiv) in CH_2Cl_2/methanol (1:1, 6 mL) at 0 °C was added a freshly prepared solution of $NaBH_4$ (17 mg, 0.45 mmol, 1.5 equiv) in methanol (3 mL). After stirring for 30 minutes, the reaction mixture was diluted with EtOAc, washed with water and brine, dried over Na_2SO_4, filtered on silica and concentrated *in vacuo*. The residue was purified by flash chromatography on silica gel (cyclohexane/EtOAc 10:1 to 1:1) to afford secondary alcohol **S-8** as a pale yellow oil (55 mg, 0.19 mmol, 63%). **R$_f$**=0.24 (cyclohexane/EtOAc 1:1). **^{1}H-NMR** (400 MHz, $CDCl_3$, 25 °C): δ 7.20 (d, *J* = 4.5 Hz, 1H, H-8), 5.97-5.66 (m, 2H, H-2, H-11), 5.46-5.38 (m, 1H, H-1), 5.35-5.30 (m, 1H, H-1), 5.24-5.15 (m, 2H, H-12), 5.06 (dt, *J* = 13.4, 6.8 Hz, 1H, H-9), 4.71 (t, *J* = 8.0 Hz, 1H, H-3), 3.93 (ddt, *J* = 9.5, 6.6, 3.2 Hz, 1H, H-5), 2.92 (dq, *J* = 10.2, 7.3 Hz, 1H, H-14), 2.60-2.46 (m, 4H, H-6, H-10), 1.97-1.90 (m, 1H, H-4), 1.37 (d, *J* = 7.2 Hz, 2H, H-13). **^{13}C-NMR** (100 MHz, $CDCl_3$, 25

°C): δ 177.7 (C-15), 173.4 (C-16), 173.4 (C-16), 150.3 (C-8), 150.2 (C-8), 134.1 (C-2), 130.6 (C-7), 130.5 (C-7), 129.7 (C-11), 118.9 (C-12), 118.8 (C-12), 118.5 (C-1), 118.4 (C-1), 80.1 (C-9), 79.4 (C-3), 79.4 (C-3), 66.6 (C-5), 66.6 (C-5), 53.9 (C-4), 53.8 (C-4), 36.2 (C-10), 36.1 (C-10), 34.3 (C-14), 34.3 (C-14), 32.2 (C-6), 32.1 (C-6), 15.8 (C-13), 15.8 (C-13) ppm.

Compound S-9

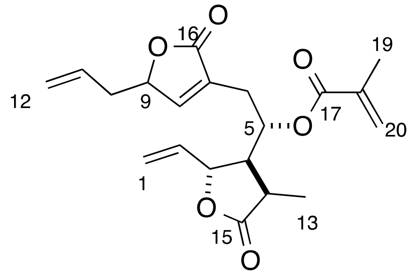

Following general procedure E with alcohol **S-8** (55 mg, 0.19 mmol), methacrylate **S-9** was obtained as a mixture of diastereomers as a pale yellow oil (45 mg, 0.13 mmol, 66%). **R$_f$**=0.41 (cyclohexane/EtOAc 1:1). **^{1}H-NMR** (400 MHz, $CDCl_3$, 25 °C): δ 7.11 (dq, J = 5.1, 1.5 Hz, 1H, H-8), 6.15-6.10 (m, 1H, H-20), 5.84 (ddd, J = 17.2, 10.6, 6.9 Hz, 1H, H-2), 5.76-5.63 (m, 2H, H-11, H-20), 5.50-5.32 (m, 3H, H-1, H-5), 5.22-5.11 (m, 2H, H-12), 4.94 (dddq, J = 6.4, 4.7, 2.8, 1.5 Hz, 1H, H-9), 4.50 (ddt, J = 8.2, 7.2, 1.1 Hz, 1H, H-3), 2.82-2.71 (m, 1H, H-14), 2.71-2.56 (m, 2H, H-6), 2.53-2.29 (m, 2H, H-10), 2.20 (td, J = 8.9, 3.6 Hz, 1H, H-4), 1.93 (d, J = 1.0 Hz, 1H, H-19), 1.40 (d, J = 7.3 Hz, 3H, H-13) ppm. **^{13}C-NMR** (100 MHz, $CDCl_3$, 25 °C): δ 181.9 (C-15), 177.8 (C-16), 149.9 (C-), 149.8 (C-), 135.4 (C-18), 134.56 (C-2), 130.9 (C-11), 130.6 (C-11), 130.1 (C-7), 127.1 (C-20), 120.0 (C-12), 119.6 (C-1), 80.4 (C-9), 80.2 (C-3), 69.9 (C-5), 52.8 (C-4), 37.2 (C-10), 36.5 (C-14), 29.2 (C-6), 18.2 (C-19), 16.6 (C-), 14.1 (C-13) ppm.

Compound S-10

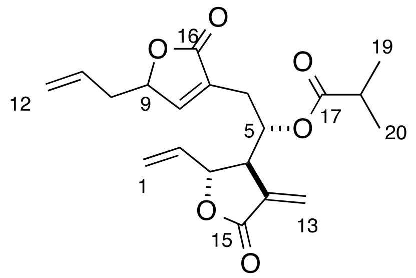

A solution of alcohol ***rac*-9** (87 g, 0.3 mmol, 1 equiv) in dry CH_2Cl_2 (3 mL) cooled to 0 °C was successively added Et_3N (168 µL, 0.6 mmol, 2 equiv), DMAP (7.3 mg, 60 µmol, 0.2 equiv) and isobutyryl chloride (47 µL, 0.45 mmol, 1.5 equiv) and the resulting mixture was stirred until complete consumption of the starting material. The reaction mixture was then diluted with Et_2O, water was added and stirring was continued for 30 min, dried over Na_2SO_4, filtered on silica and concentrated *in vacuo*. The residue was dry-loaded and purified by flash chromatography on silica gel (cyclohexane/EtOAc 10:1 to 1:1) to afford methacrylate **S-10** as a pale yellow oil (47 mg, 131 µmol, 44%). **R$_f$**=0.39 (cyclohexane/EtOAc 1:1). **^{1}H-NMR** (400 MHz, $CDCl_3$, 25 °C): δ 7.14 (bs, 1H, H-8), 6.46 (d, J = 1.9 Hz, 1H, H-13), 5.93-5.86 (d, J = 1.9 Hz, 1H, H-13), 5.85-5.65 (m, 2H, H-2, H-11), 5.44-5.32 (m, 2H, H-5, H-12), 5.25 (d, J = 10.4 Hz, 1H, H-12), 5.22-5.13 (m, 4H, H-1, H-3), 4.99-4.97 (m, 1H, H-9), 3.13-3.05 (m, 1H, H-4), 2.63 (m, 2H, H-6), 2.47- (m, 3H, H-10, H-18), 1.13

(d, J = 4.0 Hz, 1H, H-19), 1.11 (d, J = 4.5 Hz, 4H, H-20) ppm. **^{13}C-NMR** (100 MHz, $CDCl_3$, 25 °C): δ 176.3 (C-15), 176.3 (C-15), 172.8 (C-16), 172.8 (C-16), 169.3 (C-17), 169.3 (C-17), 150.2 (C-), 150.2 (C-), 135.1 (C-2), 135.0 (C-2), 133.2 (C-14), 133.2 (C-14), 130.9 (C-11), 130.8 (C-11), 130.0 (C-7), 126.2 (C-13), 126.1 (C-13), 119.7 (C-1), 119.6 (C-1), 117.8 (C-12), 80.5 (C-9), 79.3 (C-9), 71.8 (C-5), 71.7 (C-5), 48.0 (C-4), 48.0 (C-4), 37.3 (C-10), 37.2 (C-10), 34.0 (C-18), 26.9 (C-6), 26.9 (C-6), 18.8 (C-19, C-20) ppm.

Compound 11

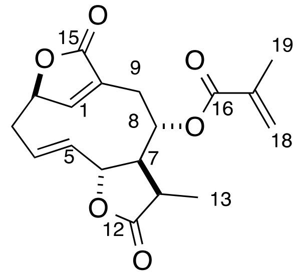

Following general procedure F with diene **S-9** (30 mg, 84 μmol), analogue **11** was obtained as a diastereomer as a pale yellow oil (2.2 mg, 6.7 μmol, 32% based on recovered starting material and reacted diastereomer). **R$_f$**=0.34 (cyclohexane/EtOAc1:2). **R$_t$**=8.1 min (HPLC reverse phase MeCN in H_2O, 30% to 90%, 3 mL/min). **^{1}H-NMR** (500 MHz, $CDCl_3$, 25 °C): δ 6.91 (t, J = 1.4 Hz, 1H, H-1), 6.17 (p, J = 1.0 Hz, 1H, H-18), 5.69 (p, J = 1.0 Hz, 1H, H-18), 5.46 (ddd, J = 16.2, 10.0, 4.8 Hz, 1H, H-4), 5.34 (dq, J = 3.9, 1.8 Hz, 1H, H-2), 5.22 (ddd, J = 11.0, 6.8, 2.3 Hz, 1H, H-8), 5.05 (dd, J = 16.2, 9.5 Hz, 1H, H-5), 4.58 (t, J = 9.4 Hz, 1H, H-6), 2.96 (dt, J = 13.1, 4.7 Hz, 1H, H-3), 2.90 (ddd, J = 12.9, 3.7, 2.3 Hz, 1H, H-9), 2.68 (dd, J = 12.8, 11.0 Hz, 1H, H-9), 2.51 (dq, J = 12.0, 6.9 Hz, 1H, H-11), 2.38 (ddd, J = 13.1, 10.2, 1.1 Hz, 1H, H-3), 2.12 (ddd, J = 12.0, 9.2, 6.8 Hz, 1H, H-7), 1.99 (dd, J = 1.6, 0.9 Hz, 3H, H-19), 1.36 (d, J = 6.9 Hz, 3H, H-13) ppm. **^{13}C-NMR** (125 MHz, $CDCl_3$, 25 °C): δ 177.1 (C-12), 172.0 (C-15), 166.1 (C-16), 151.8 (C-1), 139.9 (C-5), 135.6 (C-17), 130.7 (C-10), 126.9 (C-18), 125.3 (C-4), 80.9 (C-6), 79.8 (C-2), 70.4 (C-8), 56.9 (C-7), 38.9 (C-11), 34.6 (C-3), 34.3 (C-9), 18.4 (C-19), 16.5 (C-13) ppm. (C-14 and H-14 omitted to respect the original assignment for Deoxyelephantopin). **HR-MS (+ESI)** [M+Na]$^+$: calcd: 355.1152, found: 355.1153.

Compound 12

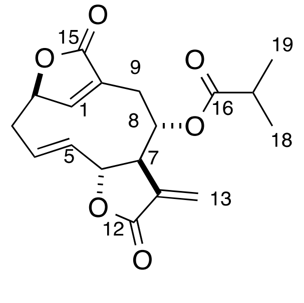

Following general procedure F with diene **S-10** (30 mg, 84 μmol), analogue **12** was obtained as a diastereomer as a pale yellow oil (2.6 mg, 7.9 μmol, 38 % based on recovered starting material and reacted diastereomer). **R$_f$**=0.33 (cyclohexane/EtOAc 1:2). **R$_t$**=8.3 min (HPLC reverse phase MeCN in H_2O, 30% to 90%, 3 mL/min). **^{1}H-NMR** (500 MHz, $CDCl_3$, 25 °C): δ 6.99 (t, J = 1.3 Hz, 1H, H-1), 6.31 (dd, J = 3.7, 0.8 Hz, 1H, H-13), 5.63 (dd, J = 3.2, 0.8 Hz, 1H, H-13), 5.45 (ddd, J = 16.2, 10.6, 4.3 Hz, 1H, H-4), 5.36 (dq, J = 3.8, 1.9 Hz, 1H, H-2), 5.28 (dd, J = 16.2, 9.5 Hz, 1H, H-5), 4.86 (dd, J = 9.4, 7.4 Hz, 1H, H-6), 4.51 (ddd, J = 11.7, 3.5, 2.2 Hz, 1H, H-7), 2.99-2.92 (m,

2H, H-3, H-9), 2.88 (dq, J = 7.0, 3.4 Hz, 1H, H-7), 2.69 (t, J = 12.1 Hz, 1H, H-9), 2.58-2.46 (m, 1H, H-17), 2.46 (ddd, J = 13.0, 10.9, 2.1 Hz, 2H, H-3), 1.18 (d, J = 7.0 Hz, 3H, H-18), 1.12 (d, J = 7.0 Hz, 3H, H-19) ppm. **^{13}C-NMR** (125 MHz, $CDCl_3$, 25 °C): δ 176.2 (C-12), 172.3 (C-15), 169.0 (C-16), 152.8 (C-1), 139.3 (C-5), 133.4 (C-11), 130.0 (C-10), 125.7 (C-4), 124.1 (C-13), 82.3 (C-2), 80.3 (C-6), 71.0 (C-8), 51.0 (C-7), 34.2 (C-3), 33.6 (C-17), 29.7 (C-9), 19.0 (C-18), 18.6 (C-19) ppm. (C-14 and H-14 omitted to respect the original assignment for Deoxyelephantopin). **HR-MS (+ESI)** $[M+Na]^+$: calcd: 355.1152, found: 355.1150.

Synthesis of compounds 18-20

Compound S-13

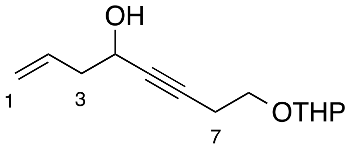

Dess-Martin periodinane (10.7 g, 25 mmol, 1.1 equiv) was added to a solution of 3-buten-1-ol **S-11** (2.0 mL, 23 mmol, 1.0 equiv) in Et_2O (200 mL) at room temperature. After 1 hour, the stirring was stopped and the suspension let to settle. The clear supernatant was transferred *via* cannula to a solution of 2-(3-butynyloxy)tetrahydro-2*H*-pyran **S-12** (14.5 g, 94 mmol, 4.1 equiv) and *n*-BuLi (55 mL, 1.6 M, 88 mmol, 3.8 equiv) in Et_2O (300 mL) at -78 °C. The resulting mixture was then warmed to room temperature. After 2 hours, the reaction was quenched with water and the aqueous phase was extracted with Et_2O. The collected organic layers were washed with brine, dried over Na_2SO_4 and filtered. Evaporation of the solvent under reduced pressure followed by flash chromatography (SiO_2, 90/10 to 50/50 pentane/Et_2O) yielded alcohol **S-13** as a pale yellow oil (2.1 g, 9.4 mmol, 41%). **R_f**=0.39 (75/25 petroleum ether/EtOAc). **^{1}H-NMR** (400 MHz, $CDCl_3$, 25 °C): δ 5.89 (ddt, J=16.9, 10.5, 7.1 Hz, 1H, H-2), 5.17 (bd, J=16.9 Hz, 1H, H-1), 5.17 (bd, J=10.5 Hz, 1H, H-1), 4.65-4.63 (m, 1H, H-THP), 4.40 (qt, J=6.0, 2.0 Hz, 1H, H-4), 3.91-3.78 (m, 2H, H-8, H-THP), 3.57-3.45 (m, 2H, H-8, H-THP), 2.52 (td, J=7.2, 2.0 Hz, 2H, H-7), 2.46-2.42 (m, 2H, H-3), 1.90 (d, J=6.0 Hz, 1H, OH), 1.86-1.50 (m, 6H, H-THP) ppm. **^{13}C-NMR** (100 MHz, $CDCl_3$, 25 °C): δ 133.2 (C-2), 118.7 (C-1), 98.7 (C-THP), 82.7 (C-6), 81.5 (C-5), 65.6 (C-8), 62.2 (C-THP), 61.7 (C-4), 42.4 (C-3), 30.5 (C-THP), 25.4 (C-THP), 20.2 (C-THP), 19.4 (C-7) ppm.

Compound 15

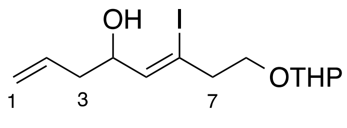

Following general procedure A, alcohol **S-13** (1.0 g, 4.5 mmol, 1.0 equiv) gave, after purification by flash chromatography (SiO_2, 80/20 pentane/Et_2O), alcohol **15** as a yellow oil (1.2 g, 3.4 mmol, 76%). **R_f**=0.45 (75/25 petroleum ether/EtOAc). **^{1}H-NMR** (400 MHz, $CDCl_3$, 25 °C): δ 5.90-5.79 (m, 1H, H-2), 5.73 (d, J=8.2 Hz, 1H, H-5), 5.17 (bd, J=16.9 Hz, 1H, H-1), 5.15 (bd, J=10.5 Hz, 1H, H-1), 4.61-4.59 (m, 1H, H-THP), 4.38-4.33 (m, 1H, H-4), 3.88-3.77

(m, 2H, H-8, H-THP), 3.58-3.47 (m, 2H, H-8, H-THP), 2.85-2.73 (m, 2H, H-7), 2.42-2.28 (m, 2H, H-3), 1.96-1.51 (m, 6H, H-THP) ppm (OH signal not visible). **^{13}C-NMR** (100 MHz, $CDCl_3$, 25 °C): δ 138.6 (C-5), 138.5 (C-5), 133.6 (C-2), 118.6 (C-1), 98.9 (C-THP), 75.5 (C-4), 65.8 (C-8), 62.3 (C-THP), 45.6 (C-7), 45.5 (C-7), 40.5 (C-3), 30.5 (C-THP), 30.6 (C-THP), 25.4 (C-THP), 19.5 (C-THP). 19.3 (C-THP) ppm (one quaternary carbon).

Compound S-14

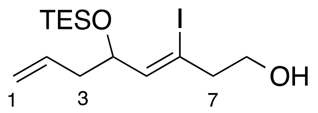

2,4,6-Trimethylpyridine (797 uL, 6 mmol, 4.0 equiv) and TESOTf (970 uL, 4.5 mmol, 3.0 equiv) were added to a solution of alcohol **15** (528 mg, 1.5 mmol, 1.0 equiv) in CH_2Cl_2 (10.5 mL) at 0 °C. The resulting solution was stirred for 1 hour at this temperature. The reaction was quenched with water and let stirred 15 minutes further. The aqueous phase was extracted with EtOAc. The collected organic layers were washed with brine, dried over Na_2SO_4 and filtered. Evaporation of the solvent under reduced pressure followed by flash chromatography (SiO_2, 95/5 to 80/20 pentane/EtOAc), yielded primary alcohol **S-14** as a yellow oil (336 mg, 0.88 mmol, 59%). $\mathbf{R_f}$=0.82 (75/25 petroleum ether/EtOAc). **^{1}H-NMR** (400 MHz, $CDCl_3$, 25 °C): δ 5.86 (ddt, J=17.2, 10.2, 7.2 Hz, 1H, H-7), 5.69 (d, J=7.7 Hz, 1H, H-4), 5.08 (d, J=17.2, 1H, H-8), 5.06 (d, J=10.2, 1H, H-8), 4.37 (dd, J=7.7, 6.3 Hz, 1H, H-5), 3.75-3.68 (m, 2H, H-1), 2.73-2.70 (m, 2H, H-2), 2.39-2.24 (m, 2H, H-6), 0.95 (t, J=7.8 Hz, 9H, Si-CH_2-CH_3), 0.61 (q, J=7.8 Hz, 6H, Si-CH_2-CH_3) ppm (OH signal not visible). **^{13}C-NMR** (100 MHz, $CDCl_3$, 25 °C): δ 141.1 (C-4), 134.4 (C-7), 117.4 (C-8), 102.1 (C-3), 76.9 (C-1), 60.8 (C-5), 48.1 (C-2), 42.1 (C-6), 6.9 (Si-CH_2-*C*H_3), 4.9 (Si-*C*H_2-CH_3) ppm.

Compound 16

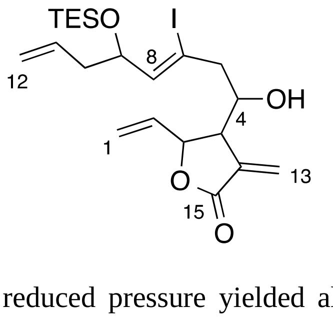

Dess-Martin periodinane (610 mg, 1.4 mmol, 1.1 equiv) was added to a solution of alcohol **S-14** (500 mg, 1.3 mmol, 1.0 equiv) in Et_2O (15 mL) at room temperature. After 1 hour, the stirring was stopped and the suspension let to settle. The clear supernatant was washed with sat. aqueous $NaHCO_3$, water and brine, dried over Na_2SO_4 and filtered. Evaporation of the solvent under reduced pressure yielded aldehyde **S-15**, which was used in the next step without further purification. To a solution of the crude aldehyde **S-15** (0.50 g, 1.3 mmol, 1.0 equiv) and bromolactone **2** (290 mg, 1.4 mmol, 1.1 equiv) in a mixture of THF (1.3 mL) and sat. aqueous NH_4Cl (0.65 mL) at room temperature was added zinc powder (110.5 mg, 1.7 mmol, 1.3 equiv). The mixture was stirred for 20 minutes. After addition of water, the aqueous phase was extracted with EtOAc and the collected organic layers were washed with brine, dried over

Na_2SO_4 and filtered. Evaporation of the solvent under reduced pressure followed by flash chromatography as indicated (SiO_2, 90/10 pentane/Et_2O), alcohol **16** as a mixture of diastereomers as a yellow oil (140 mg, 0.28 mmol, 21% over 2 steps). **R$_f$**=0.65 (75/25 petroleum ether/EtOAc). **^{1}H-NMR** (400 MHz, $CDCl_3$, 25 °C): δ 6.43 (bs, 1H, H-13), 5.89-5.64 (m, 4H, H-11, H-8, H-13, H-2), 5.47-5.24 (m, 2H, H-1), 5.10-5.05 (m, 2H, H-12), 4.91-4.89 (m, 1H, H-3), 4.38-4.41 (m, 1H, H-9), 4.09-4.14 (m, 1H, H-5), 3.00-2.98 (m, 1H, H-4), 2.67-2.57 (m, 2H, H-6), 2.40-2.26 (m, 2H, H-10), 0.95 (t, J=8.0 Hz, 9H, Si-CH_2-CH_3), 0.64-0.57 (m, 6H, Si-CH_2-CH_3) ppm (OH signal not visible). **^{13}C-NMR** (100 MHz, $CDCl_3$, 25 °C): δ 169.7 (C-15), 142.8 (C-8), 142.7 (C-8), 135.7 (C-2), 135.6 (C-2), 134.6 (C-11), 134.4 (C-11), 125.7 (C-13), 125.5 (C-13), 117.7 (C-12), 117.6 (C-12), 117.5 (C-1), 117.4 (C-1), 100.7 (C-7), 100.5 (C-7), 79.5 (C-3), 79.3 (C-3), 76.7 (C-9), 76.5 (C-9), 70.7 (C-5), 70.2 (C-5), 49.1 (C-4), 49.0 (C-6), 42.4 (C-10), 6.8 (Si-CH_2-CH$_3$), 4.9 (Si-CH$_2$-CH_3) ppm (one quaternary carbon not visible).

Compound S-16a

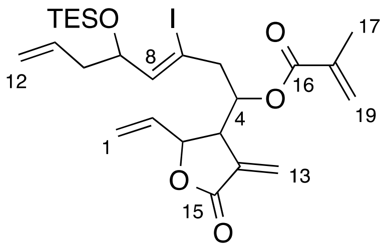

Following general procedure G, alcohol **16** (280 mg, 0.56 mmol, 1.0 equiv) gave, after purification by flash chromatography (SiO_2, 90/10 pentane/Et_2O), ester **S-16a** as a pale yellow oil (117 mg, 0.21 mmol, 38%). **R$_f$**=0.80 (75/25 petroleum ether/EtOAc). **^{1}H-NMR** (400 MHz, $CDCl_3$, 25 °C): δ 6.47-6.45 (m, 1H, H-13), 6.08 (s, 1H, H-19), 5.90-5.61 (m, 5H, H-11, H-8, H-19, H-13, H-2), 5.48-5.23 (m, 3H, H-5, H-1), 5.08-5.00 (m, 2H, H-12), 4.91-4.86 (m, 1H, H-3), 4.34-4.19 (m, 1H, H-9), 3.21-3.11 (m, 1H, H-4), 2.90 (dd, J=14.4, 8.6 Hz, 1H, H-6), 2.75 (dd, J=14.4, 4.8 Hz, 1H, H-6), 2.34-2.15 (m, 2H, H-10), 1.93 (s, 1.5H, H-17), 1.90 (s, 1.5H, H-17), 0.97-0.90 (m, 9H, Si-CH_2-CH_3), 0.61-0.55 (m, 6H, Si-CH_2-CH_3) ppm. **^{13}C-NMR** (100 MHz, $CDCl_3$, 25 °C): δ 169.4 (C-15), 166.4 (C-16), 165.9 (C-16), 142.5 (C-8), 142.3 (C-8), 135.3 (C-2), 135.2 (C-2), 134.3 (C-11), 127.0 (C-19), 126.9 (C-19), 125.8 (C-13), 125.6 (C-13), 117.6 (C-1), 117.5 (C-12), 98.6 (C-7), 79.6 (C-3), 79.4 (C-3), 76.9 (C-9), 76.6 (C-9), 73.5 (C-5), 72.9 (C-5), 48.4 (C-4), 46.7 (C-4), 45.8 (C-6), 44.9 (C-6), 43.1 (C-10), 41.8 (C-10), 18.2 (C-17), 6.8 (Si-CH_2-CH$_3$), 4.8 (Si-CH$_2$-CH_3) ppm (two quaternary carbons not visible).

Compound S-16b

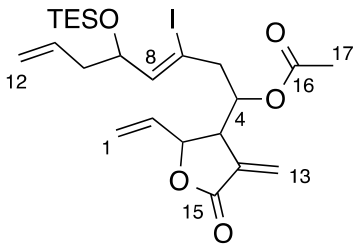

Following general procedure H, alcohol **16** (10.5 mg, 20 µmol, 1.0 equiv) gave, after purification by flash chromatography (SiO_2, 90/10 pentane/Et_2O), ester **S-16b** as a yellow oil (2.5 mg, 5.0 µmol, 50%). **R_f**=0.57 (75/25 petroleum ether/EtOAc). **^{1}H-NMR** (400 MHz, $CDCl_3$, 25 °C): δ 6.45-6.40 (m, 1H, H-13), 5.86-5.65 (m, 4H, H-11, H-8, H-13, H-2), 5.48-5.34 (m, 2H, H-5, H-1), 5.26 (d, *J*=10.6 Hz, 1H, H-1), 5.09-5.03 (m, 2H, H-12), 4.89-4.87 (m, 1H, H-3), 4.34-4.25 (m, 1H, H-9), 3.17-3.13 (m, 1H, H-4), 2.86-2.64 (m, 2H, H-6), 2.30-2.20 (m, 2H, H-10), 2.03 (s, 1.5H, H-17), 2.02 (s, 1.5H, H-17), 0.94 (t, *J*=8.0 Hz, 9H, Si-CH_2-C*H_3*), 0.59 (q, *J*=8.0 Hz, 6H, Si-C*H_2*-CH_3) ppm. **^{13}C-NMR** (100 MHz, $CDCl_3$, 25 °C): δ 170.1 (C-15), 169.9 (C-15), 169.6 (C-16), 169.3 (C-16), 142.4 (C-8), 142.2 (C-8), 135.3 (C-2), 135.2 (C-2), 133.8 (C-11), 125.7 (C-13), 125.6 (C-13), 117.7 (C-1), 117.6 (C-1), 117.5 (C-12), 98.7 (C-7), 98.6 (C-7), 79.4 (C-3), 79.3 (C-3), 76.8 (C-9), 76.6 (C-9), 73.0 (C-5), 72.5 (C-5), 48.3 (C-4), 47.2 (C-4), 45.6 (C-6), 44.9 (C-6), 43.1 (C-10), 41.8 (C-10), 20.9 (C-17), 20.8 (C-17), 6.8 (Si-CH_2-*C*H_3), 4.9 (Si-*C*H_2-CH_3) ppm (one quaternary carbon not visible).

Compound 17a

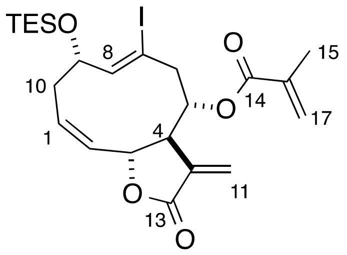

Grubbs II catalyst (48 mg, 57 µmol, 0.30 equiv) was added to a solution of compound **S-16a** (110 mg, 0.19 mol, 1.0 equiv) in CH_2Cl_2 (2 L, 0.1 mM) at reflux. The mixture was stirred for 12 hours. Evaporation of the solvent under reduced pressure followed by flash chromatography (SiO_2, 90/10 pentane/Et_2O) yielded cyclic compound **17a** as a yellow oil (34 mg, 63 µmol, 33%). **R_f**=0.59 (75/25 petroleum ether/EtOAc). **^{1}H-NMR** (400 MHz, $CDCl_3$, 25 °C): δ 6.35 (s, 1H, H-11), 6.08 (s, 1H, H-17), 5.89 (td, *J*=10.4, 6.9 Hz, 1H, H-1), 5.85 (d, *J*=6.0 Hz, 1H, H-8), 5.70 (s, 1H, H-11), 5.59 (s, 1H, H-17), 5.47 (t, *J*=10.4 Hz, 1H, H-2), 5.19 (td, *J*=10.3, 3.7 Hz, 1H, H-5), 4.87 (d, *J*=10.4 Hz, 1H, H-3), 4.46-4.43 (m, 1H, H-9), 3.05-2.92 (m, 3H, 6, H-4), 2.85-2.78 (m, 1H, H-10), 2.36-2.22 (m, 1H, H-10), 1.91 (s, 3H, H-15), 0.96 (t, *J*=8.0 Hz, 9H, Si-CH_2-C*H_3*), 0.60 (q, *J*=8.0 Hz, 6H, Si-C*H_2*-CH_3) ppm. **^{13}C-NMR** (100 MHz, $CDCl_3$, 25 °C): δ 169.2 (C-13), 165.7 (C-14), 145.1 (C-8), 135.9 (C-16), 134.0 (C-11), 129.9 (C-2), 128.4 (C-1), 127.6 (C-12), 126.1 (C-17), 94.7 (C-7), 76.1 (C-9), 75.7 (C-3), 69.9 (C-5), 52.3 (C-6), 50.2 (C-4), 32.8 (C-10), 18.3 (C-15), 6.8 (Si-CH_2-*C*H_3), 4.7 (Si-*C*H_2-CH_3) ppm.

Compound 17b

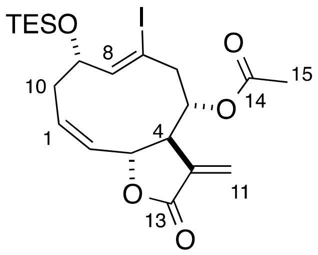

Grubbs II catalyst (14 mg, 16 µmol, 0.30 equiv) was added to a solution of compound **S-16b** (30 mg, 55 µmol, 1.0 equiv) in CH_2Cl_2 (500 mL, 0.1 mM) at reflux. The mixture was stirred for 12 hours. Evaporation of the solvent under reduced pressure followed by flash chromatography (SiO_2, 90/10 pentane/Et_2O) yielded cyclic compound **17b** as a yellow oil (8.0 mg, 15 µmol, 30%). $\mathbf{R_f}$=0.55 (75/25 petroleum ether/EtOAc). **^{1}H-NMR** (400 MHz, $CDCl_3$, 25 °C): δ 6.39 (s, 1H, H-11), 5.88 (td, *J*=10.4, 6.8 Hz, 1H, H-1), 5.83 (d, *J*=5.7 Hz, 1H, H-8), 5.74 (s, 1H, H-11), 5.47 (t, *J*=10.4 Hz, 1H, H-2), 5.11 (td, *J*=10.4, 3.6 Hz, 1H, H-5), 4.85 (dt, *J*=10.4, 1.5 Hz, 1H, H-3), 4.44 (dt, *J*=5.8, 3.0 Hz, 1H, H-9), 3.01-2.77 (m, 4H, H-10, H-6, H-4), 2.20-2.14 (m, 1H, H-10), 2.01 (s, 3H, H-15), 0.96 (t, *J*=8.0 Hz, 9H, Si-CH_2-CH_3), 0.60 (q, *J*=8.0 Hz, 6H, Si-CH_2-CH_3) ppm. **^{13}C-NMR** (100 MHz, $CDCl_3$, 25 °C): δ 169.2 (C-13), 169.1 (C-14), 145.1 (C-8), 134.4 (C-11), 129.9 (C-2), 128.4 (C-1), 127.2 (C-12), 94.7 (C-7), 76.1 (C-9), 75.6 (C-3), 69.4 (C-5), 52.3 (C-6), 50.0 (C-4), 32.7 (C-10), 20.9 (C-15), 6.7 (Si-CH_2-CH_3), 4.7 (Si-CH_2-CH_3) ppm.

Compound 18

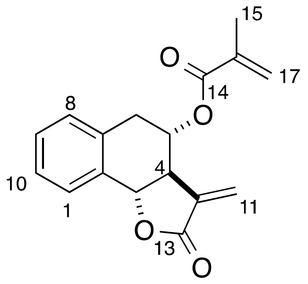

Methyl boronic acid (1.1 mg, 19 µmol, 3.0 equiv), K_3PO_4-H_2O (4.4 mg, 13 µmol, 2.0 equiv), $Pd(OAc)_2$ (0.29 mg, 1.3 µmol, 0.2 equiv), 2-dicyclohexylphosphino-2',6'-dimethoxybiphenyl (1.1 mg, 2.6 µmol, 0.4 equiv) were added to a solution of cyclic compound **17a** (3.5 mg, 6.4 µmol, 1.0 equiv) in toluene (0.20 mL). The resulting mixture was stirred at 60 °C for 48 hours. Filtration of the reaction mixture (SiO_2, 80/20 pentane/Et_2O) yielded a yellow oil, which was used in the next step without further purification. $\mathbf{R_f}$=0.77 (75/25 petroleum ether/EtOAc). PTSA monohydrate (0.12 mg, 0.64 µmol, 0.10 equiv) was added to a solution of the crude product (2.8 mg, 6.4 µmol, 1.0 equiv) in 5/1 THF/H_2O (0.60 mL) at room temperature. After 2 hours, the reaction was quenched by addition of sat. aqueous $NaHCO_3$. The organic phase was dried over Na_2SO_4 and filtered. Evaporation of the solvent under reduced pressure followed by flash chromatography (SiO_2, 90/10 pentane/EtOAc) yielded tricyclic **18** (1.4 mg, 4.9 µmol, 60% over 2 steps). $\mathbf{R_f}$=0.63 (75/25 petroleum ether/EtOAc). **^{1}H-NMR** (500 MHz, $CDCl_3$, 25 °C): δ 7.47-7.46 (m, 1H, H-Ar), 7.30-7.28 (m, 2H, H-Ar), 7.19-7.17 (m, 1H, H-Ar), 6.28 (d, *J*=3.1 Hz, 1H, H-11), 6.17 (s, 1H, H-15), 5.74 (d, *J*=3.1 Hz, 1H, H-11), 5.68 (t, *J*=1.7 Hz, 1H,

H-15), 5.53 (ddd, *J*=10.6, 8.0, 5.7 Hz, 1H, H-5), 4.99 (d, *J*=11.6 Hz, 1H, H-3), 3.64 (dd, *J*=17.7, 8.0 Hz, 1H, H-6), 3.14 (ddt, *J*=11.6, 10.6, 3.1 Hz, 1H, H-4), 2.95 (dd, *J*=17.7, 5.7 Hz, 1H, H-6), 2.00 (bs, 3H, H-15) ppm. **^{13}C-NMR** (125 MHz, $CDCl_3$, 25 °C): δ 169.7 (C-13), 166.6 (C-14), 136.5 (C-12), 135.7 (C-16), 134.7 (C-Ar), 132.2 (C-Ar), 128.8 (C-Ar), 128.3 (C-Ar), 126.7 (×2, C-17, C-Ar), 122.5 (C-Ar), 121.1 (C-11), 77.4 (C-3), 69.7 (C-5), 49.3 (C-4), 36.3 (C-6), 18.3 (C-15) ppm. **HR-MS (+ESI)** $[M+Na]^+$: calcd: 307.0941, found: 307.0941.

Compound S-17a

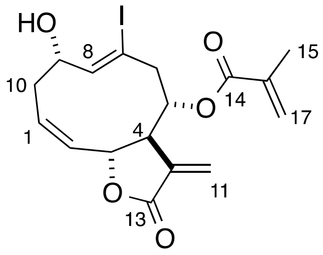

PTSA monohydrate (0.59 mg, 3.1 µmol, 0.10 equiv) was added to a solution of compound **17a** (17 mg, 31 µmol, 1.0 equiv) in 5/1 THF/H_2O (2.4 mL) at room temperature. After 2 hours, the reaction was quenched by addition of sat. aqueous $NaHCO_3$. The organic phase was dried over Na_2SO_4 and filtered. Evaporation of the solvent under reduced pressure yielded alcohol **S-17a** as a yellow oil, which was used in the next without further purification. **^{1}H-NMR** (400 MHz, $CDCl_3$, 25 °C): δ 6.36 (s, 1H, H-11), 6.08 (s, 1H, H-17), 5.93-5.86 (m, 2H, H-8, H-1), 5.73 (s, 1H, H-11), 5.60 (s, 1H, H-17), 5.52 (t, *J*=10.4 Hz, 1H, H-2), 5.21 (td, *J*=10.4, 3.6 Hz, 1H, H-5), 4.88 (d, *J*=10.4 Hz, 1H, H-3), 4.56 (dt, *J*=6.0, 3.3 Hz, 1H, H-9), 3.08-2.86 (m, 4H, H-10, H-6, H-4), 2.39-2.30 (m, 1H, H-10), 1.92 (s, 3H, H-15) ppm.

Compound S-17b

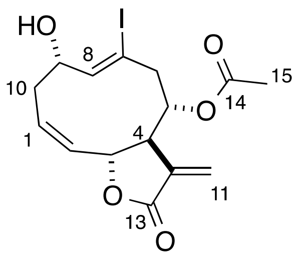

PTSA monohydrate (0.26 mg, 1.4 µmol, 0.10 equiv) was added to a solution of compound **17b** (7.0 mg, 14 µmol, 1.0 equiv) in 5/1 THF/H_2O (0.60 mL) at room temperature. After 2 hours, the reaction was quenched by addition of sat. aqueous $NaHCO_3$. The organic phase was dried over Na_2SO_4 and filtered. Evaporation of the solvent under reduced pressure yielded alcohol **S-17b** as a yellow oil, which was used in the next without further purification. **^{1}H-NMR** (400 MHz, $CDCl_3$, 25 °C): δ 6.40 (s, 1H, H-11), 5.92-5.85 (m, 2H, H-8, H-1), 5.76 (s, 1H, H-11), 5.51 (t, *J*=10.4 Hz, 1H, H-2), 5.12 (td, *J*=10.4, 3.6 Hz, 1H, H-5), 4.86 (dt, *J*=10.4, 1.5 Hz, 1H, H-3), 4.55 (dt, *J*=5.8, 3.0 Hz, 1H, H-9), 3.04-2.84 (m, 4H, H-10, H-6, H-4), 2.38-2.32 (m, 1H, H-10), 2.02 (s, 3H, H-15) ppm.

Compound 19a

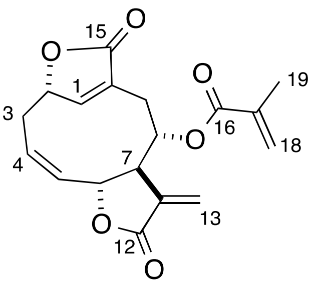

Following general procedure B, alcohol S-**17a** (14 mg, 33 µmol, 1.0 equiv) gave, after purification by flash chromatography (SiO_2, 80/20 to 50/50 pentane/EtOAc), lactone **19a** as a colorless oil (5.6 mg, 17 µmol, 52% over 2 steps). **R_f**=0.38 (50/50 petroleum ether/EtOAc). **^{1}H-NMR** (400 MHz, $CDCl_3$, 25 °C): δ 7.30 (t, *J*=1.7 Hz, 1H, H-1), 6.37 (d, *J*=2.4 Hz, 1H, H-13), 6.14 (bs, 1H, H-18), 5.78 (d, *J*=2.4 Hz, 1H, H-13), 5.66 (bs, 1H, H-18), 5.60-5.46 (m, 2H, H-4, H-5), 5.38 (ddd, *J*=8.3, 2.4, 1.7 Hz, 1H, H-2), 5.27 (ddd, *J*=11.1, 9.3, 5.5 Hz, 1H, H-8), 4.73 (dd, *J*=10.4, 3.4 Hz, 1H, H-6), 3.04 (dt, *J*=14.1, 8.3 Hz, 1H, H-3), 3.00-2.93 (m, 2H, H-9, H-7), 2.81 (dd, *J*=14.5, 11.1 Hz, H-9), 2.49 (dbd, *J*=14.1, 7.3 Hz, 1H, H-3), 1.94 (s, 3H, H-19) ppm. **^{13}C-NMR** (100 MHz, $CDCl_3$, 25 °C): δ 173.4 (C-12), 168.7 (C-15), 166.0 (C-16), 151.9 (C-1), 135.7 (C-17), 133.1 (C-13), 132.8 (C-5), 128.5 (C-11), 128.3 (C-10), 126.8 (C-18), 123.0 (C-4), 77.2 (C-2), 77.0 (C-6), 71.4 (C-8), 49.0 (C-7), 29.2 (C-9), 28.1 (C-3), 18.3 (C-19) ppm. (C-14 and H-14 omitted to respect the original assignment for Deoxyelephantopin). **HR-MS (+ESI)** $[M+Na]^+$: calcd: 353.0996, found: 353.0996.

Compound 19b

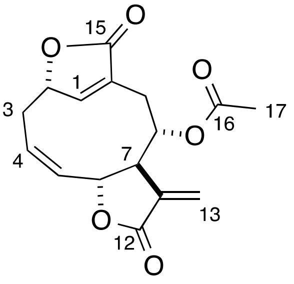

Following general procedure B, alcohol **S-17b** (4.0 mg, 9.9 µmol, 1.0 equiv) gave, after purification by flash chromatography (SiO_2, 80/20 to 50/50 pentane/EtOAc), lactone **19b** as a colorless oil (2.0 mg, 6.6 µmol, 49% over 2 steps). **R_f**=0.34 (50/50 petroleum ether/EtOAc). **^{1}H-NMR** (400 MHz, $CDCl_3$, 25 °C): δ 7.26 (bs, 1H, H-1), 6.43 (d, *J*=2.4 Hz, 1H, H-13), 5.83 (d, *J*=2.4 Hz, 1H, H-13), 5.59-5.45 (m, 1H, H-4), 5.37 (dt, *J*=8.2, 1.8 Hz, 1H, H-2), 5.16 (ddd, *J*=11.2, 9.4, 5.4 Hz, 1H, H-8), 4.70 (dd, *J*=10.5, 3.3 Hz, 1H, H-6), 3.03 (dt, *J*=14.2, 8.2 Hz, 1H, H-3), 2.97-2.90 (m, 2H, H-9, H-7), 2.75 (dd, *J*=14.3, 11.2 Hz, 1H, H-9), 2.47 (dd, *J*=14.2, 7.3 Hz, 1H, H-3), 2.08 (s, 3H, H-17) ppm. **^{13}C-NMR** (100 MHz, $CDCl_3$, 25 °C): δ 177.5 (C-12), 173.3 (C-15), 169.7 (C-16), 151.9 (C-1), 133.3 (C-11), 132.8 (C-5), 128.4 (C-13), 128.2 (C-4), 123.0 (C-4), 77.2 (C-2), 77.1 (C-6), 71.1 (C-8), 48.8 (C-7), 29.1 (C-9), 28.1 (C-3), 21.0 (C-17) ppm. (C-14 and H-14 omitted to respect the original assignment for Deoxyelephantopin). **HR-MS (+ESI)** $[M+Na]^+$: calcd: 327.0839, found: 327.0837.

Compound 20a

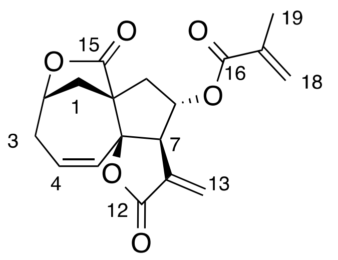

A solution of compound **19a** (1.6 mg, 4.8 µmol) in MeCN-d_8 (1.0 mL) was irradiated under UV (254 nm, 4W) for 7 days. Evaporation of the solvent yielded tetracyclic **20a** (1.6 mg, 4.8 µmol, quant). **R$_f$**=0.91 (50/50 petroleum ether/EtOAc). **^{1}H-NMR** (400 MHz, $CDCl_3$, 25 °C): δ 6.44 (s, 1H, H-11), 6.37 (s, 1H, H-18), 6.13 (s, 1H, H-13), 5.75 (dd, *J*=11.9, 6.8 Hz, 1H, H-4), 5.69 (dd, *J*=11.9, 2.6 Hz, 1H, H-5), 5.66 (bs, 1H, H-18), 4.97 (bd, *J*=8.1 Hz, 1H, H-8), 4.87 (bt, *J*=6.2 Hz, 1H, H-2), 3.56 (bs, 1H, H-7), 2.92 (dt, *J*=18.0, 6.2 Hz, 1H, H-3), 2.80 (d, *J*=13.2 Hz, 1H, H-1), 2.60-2.54 (m, 2H, H-7, H-1), 2.48 (dd, *J*=15.3, 8.1 Hz, 1H, H-9), 2.36 (d, *J*=15.3 Hz, 1H, H-9), 2.00 (s, 3H, H-19) ppm. **^{13}C-NMR** (100 MHz, $CDCl_3$, 25 °C): δ 176.7 (C-12), 168.2 (C-16), 167.3 (C-15), 136.6 (C-11), 136.0 (C-17), 131.1 (C-4), 126.9 (C-18), 126.5 (C-13), 126.4 (C-5), 94.1 (C-6), 81.0 (C-8), 74.3 (C-2), 58.3 (C-7), 54.4 (C-10), 38.8 (C-9), 35.7 (C-3), 34.8 (C-1), 18.1 (C-19) ppm. (C-14 and H-14 omitted to respect the original assignment for Deoxyelephantopin). **HR-MS (+ESI)** $[M+Na]^+$: calcd: 353.0996, found: 353.0994.

Compound 20b

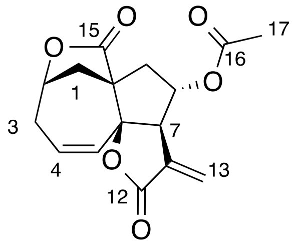

A solution of compound **19b** (0.5 mg, 1.6 µmol) in MeCN-d_8 (1.0 mL) was irradiated under UV (254 nm, 4W) for 3 days. Evaporation of the solvent yielded the tetracyclic **20b** (0.5 mg, 1.6 µmol, quant). **R$_f$**=0.91 (50/50 petroleum ether/EtOAc). **^{1}H-NMR** (500 MHz, $CDCl_3$, 25 °C): δ 6.41 (d, *J*=1.3 Hz, 1H, H-13), 6.06 (d, *J*=1.3 Hz, 1H, H-13), 5.75 (dd, *J*=12.2, 6.6 Hz, 1H, H-4), 5.68 (dd, *J*=12.2, 2.8 Hz, 1H, H-5), 4.89-4.86 (m, 2H, H-2, H-8), 3.55 (bs, 1H, H-7), 2.92 (dt, *J*=18.4, 6.6 Hz, 1H, H-3), 2.78 (d, *J*=13.2 Hz, 1H, H-1), 2.59-2.52 (m, 2H, H-3, H-1), 2.46 (dd, *J*=15.3, 8.5 Hz, 1H, H-9), 2.33 (d, *J*=15.3 Hz, 1H, H-9), 2.16 (s, 3H, H-17) ppm. **^{13}C-NMR** (125 MHz, $CDCl_3$, 25 °C): δ 176.2 (C-12), 171.5 (C-16), 168.2 (C-15), 136.7 (C-11), 131.0 (C-4), 126.2 (×2, C-13, C-5), 94.1 (C-6), 80.9 (C-8), 74.4 (C-2), 58.0 (C-7), 54.5 (C-10), 39.0 (C-6), 35.7 (C-3), 34.7 (C-1), 21.1 (C-17) ppm. (C-14 and H-14 omitted to respect the original assignment for Deoxyelephantopin). **HR-MS (+ESI)** $[M+Na]^+$: calcd: 327.0839, found: 327.0836.

Synthesis of compounds 24a-c and 24c-Cy3

Compound S-19

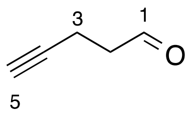

Dess-Martin periodinane (501 mg, 1.2 mmol, 1.1 equiv) was added to a stirred solution of alcohol **S-18** (0.10 mL, 1.1 mmol, 1.0 equiv) in CH_2Cl_2 (10.0 mL) at room temperature. The mixture was stirred for 1 hour at room temperature. The reaction was quenched by addition of sat. aqueous $NaHCO_3$ and the aqueous phase was extracted with CH_2Cl_2. The collected organic layers were washed with brine, dried over Na_2SO_4 and filtered. Evaporation of the solvent under reduced pressure followed by flash chromatography (SiO_2, 60/40 pentane/Et_2O) yielded aldehyde **S-19** as a yellow oil (50 mg, 0.61 mmol, 57%). **R_f**=0.65 (75/25 petroleum ether/EtOAc). **^{1}H-NMR** (400 MHz, $CDCl_3$, 25 °C): δ 9.80 (t, *J*=1.1 Hz, H-1), 2.70 (bt, *J*=7.1 Hz, 2H, H-2), 2.51 (td, *J*=7.1, 2.6 Hz, 2H, H-3), 1.99 (t, *J*=2.7 Hz, 1H, H-5) ppm.

Compound S-21a

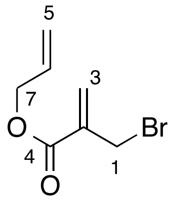

Following general procedure I for Mitsunobu esterification, allyl alcohol **21a** (0.31 mL, 0.26 g, 4.5 mmol, 1.5 equiv) gave, after purification by flash chromatography (SiO_2, 95/5 pentane/Et_2O), ester **S-21a** as a pale yellow oil (305 mg, 1.5 mmol, 50%). **R_f**=0.78 (75/25 petroleum ether/EtOAc). **^{1}H-NMR** (400 MHz, $CDCl_3$, 25 °C): δ 6.37 (s, 1H, H-3), 5.98 (s, 1H, H-3), 6.01-5.92 (m, 1H, H-6), 5.37 (dq, *J*=17.2, 1.5 Hz, 1H, H-5), 5.27 (dq, *J*=10.5, 1.5 Hz, 1H, H-5), 4.72 (dt, *J*=5.7, 1.5 Hz, 2H, H-7), 4.19 (s, 2H, H-1) ppm. **^{13}C-NMR** (100 MHz, $CDCl_3$, 25 °C): δ 164.5 (C-4), 137.3 (C-2), 131.8 (C-6), 129.3 (C-3), 118.5 (C-5), 65.8 (C-7), 29.2 (C-1) ppm.

Compound S-21b

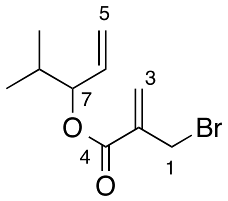

Following general procedure I for Mitsunobu esterification, 4-methyl-1-penten-3-ol **21b** (450.7 mg, 4.5 mmol, 1.5 equiv) gave, after purification by flash chromatography (SiO_2, 95/5 pentane/Et_2O), ester **S-21b** as a pale yellow oil (280 mg, 1.1 mmol, 38%). **R_f**=0.83 (75/25 petroleum ether/EtOAc). **^{1}H-NMR** (400 MHz, $CDCl_3$, 25 °C): δ 6.36 (s, 1H, H-3), 5.95 (s, 1H, H-3), 5.79 (ddt, *J*=17.4, 10.6, 1.5 Hz, 1H, H-6), 5.28 (dt, *J*=17.4, 1.5 Hz, 1H, H-5), 5.24 (dt, *J*=10.5, 1.5 Hz, 1H, H-5), 5.20-5.16 (m, 1H, H-7), 4.20 (s, 2H, H-1), 2.00-1.92 (m, 1H, C*H*-*i*Pr), 0.90 (d, *J*=6.8 Hz, CH_3-*i*Pr), 0.89 (d, *J*=6.8 Hz, CH_3-*i*Pr) ppm. **^{13}C-NMR** (100 MHz, $CDCl_3$, 25 °C): δ 164.1 (C-4), 137.7 (C-2), 134.2 (C-6), 129.0 (C-3), 117.9 (C-5), 80.4 (C-7), 31.9 (*C*H-*i*Pr), 29.3 (C-1), 18.1 (*C*H_3-*i*Pr), 17.8 (*C*H_3-*i*Pr) ppm.

Compound S-21c

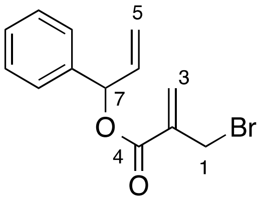

Following general procedure I, α-vinylbenzyl alcohol **21c** (2.7 g, 20 mmol, 1.2 equiv) gave, after purification by flash chromatography (SiO_2, 95/5 pentane/Et_2O), a unseparable 1:1 mixture of ester **S-21c** and its regioisomer **S-21c’** as a pale yellow oil (3.03 g, 11 mmol, 65%). $\mathbf{R_f}$=0.79 (75/25 petroleum ether/EtOAc). **^{1}H-NMR** (400 MHz, $CDCl_3$, 25 °C): δ 7.42-7.25 (m, 5H, H-Ph), 6.42 (s, 1H, H-3), 6.38-6.29 (m, 1H, H-7), 6.06 (ddd, J=17.2, 10.5, 5.9 Hz, 1H, H-6), 5.99 (s, 1H, H-3), 5.37 (dq, J=17.2, 1.5 Hz, 1H, H-5), 5.29 (dq, J=10.5, 1.5 Hz, 1H, H-5), 4.21 (s, 2H, H-1) ppm. **^{13}C-NMR** (100 MHz, $CDCl_3$, 25 °C): δ 164.6 (C-4), 138.5 (C-Ph), 137.4 (C-2), 135.9 (C-6), 129.6 (C-3), 128.6 (×2, C-Ph), 128.3 (C-Ph), 127.1 (×2, C-Ph), 117.3 (C-5), 77.2 (C-7), 29.2 (C-1) ppm.

Regioisomer S-21c’

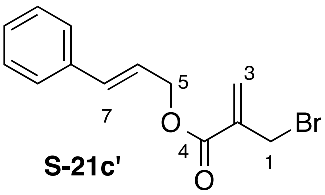

S-21c'

^{1}H-NMR (400 MHz, $CDCl_3$, 25 °C): δ 7.42-7.25 (m, 5H, H-Ph), 6.71 (d, J=15.5 Hz, 1H, H-7), 6.39 (s, 1H, H-3), 6.38-6.29 (m, 1H, H-6), 5.99 (s, 1H, H-3), 4.88 (dd, J=6.4, 1.4 Hz, 2H, H-5), 4.20 (s, 2H, H-1) ppm. **^{13}C-NMR** (100 MHz, $CDCl_3$, 25 °C): δ 164.6 (C-4), 137.4 (C-2), 136.1 (C-Ph), 134.5 (C-7), 129.4 (C-3), 128.6 (×2, C-Ph), 128.2 (C-Ph), 126.6 (×2, C-Ph), 122.8 (C-6), 65.8 (C-5), 29.3 (C-1) ppm.

Compound 22a

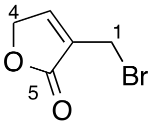

Grubbs II catalyst (60 mg, 72 µmol, 0.30 equiv) was added in portions to a refluxing solution of ester **S-21-a** (50 mg, 0.24 mmol, 1.0 equiv) in CH_2Cl_2 (50 mL, 5 mM) over 2 hours. The mixture was then filtered (SiO_2, 80/20 pentane/Et_2O) to yield lactone **56a** (12 mg, 68 µmol, 28%). $\mathbf{R_f}$=0.48 (75/25 petroleum ether/EtOAc). **^{1}H-NMR** (400 MHz, $CDCl_3$, 25 °C): δ 7.52 (quint, J=1.7 Hz, 1H, H-3), 4.86 (q, J=1.7 Hz, 2H, H-4), 4.11 (q, J=1.7 Hz, 2H, H-1) ppm. **^{13}C-NMR** (100 MHz, $CDCl_3$, 25 °C): δ 171.5 (C-5), 148.8 (C-3), 135.8 (C-2), 70.1 (C-4), 20.8 (C-1) ppm.

Compound 22b

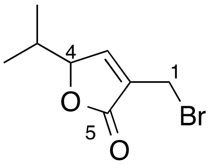

Grubbs II catalyst (212 mg, 0.26 mmol, 0.20 equiv) was added in portions to a refluxing solution of ester **S-21-b** (308 mg, 1.3 mmol, 1.0 equiv) in CH_2Cl_2 (130 mL, 0.01 M) over 1 hour. The mixture was then filtered on silica (SiO_2, 80/20 pentane/Et_2O) and the evaporation of the solvant yielded lactone **22b** (191 mg, 0.87 mmol, 70%). **R$_f$**=0.61 (75/25 petroleum ether/EtOAc). **^{1}H-NMR** (400 MHz, $CDCl_3$, 25 °C): *δ* 7.40 (bs, 1H, H-3), 4.80-4.77 (m, 1H, H-4), 4.10 (t, *J*=1.5 Hz, 2H, H-1), 2.08-1.99 (m, 1H, C*H*-*i*Pr), 1.00 (d, *J*=7.2 Hz, 6H, C*H*$_3$-*i*Pr) ppm. **^{13}C-NMR** (100 MHz, $CDCl_3$, 25 °C): *δ* 170.9 (C-5), 151.1 (C-3), 131.8 (C-2), 85.9 (C-4), 31.8 (*C*H-*i*Pr), 20.9 (C-1), 17.9 (*C*H$_3$-*i*Pr), 17.5 (*C*H$_3$-*i*Pr) ppm.

Compound 22c

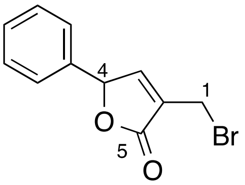

Grubbs II catalyst (318 mg, 0.38 mmol, 0.15 equiv) was added in portions to a refluxing solution of esters **S-21c** and **S-21c'** (702.8 mg, 2.5 mmol, 1.0 equiv) in CH_2Cl_2 (250 mL, 0.01 M) over 1 hour. The mixture was then filtrated on silica (SiO_2, 80/20 pentane/Et_2O) and the evaporation of the solvent yielded lactone **22c** (290 mg, 1.1 mmol, 46%, 92% from **S-21c**). **R$_f$**=0.52 (75/25 petroleum ether/EtOAc). **^{1}H-NMR** (400 MHz, $CDCl_3$, 25 °C): *δ* 7.52-7.48 (m, 1H, H-3), 7.43-7.38 (m, 3H, H-Ph), 7.29-7.27 (m, 2H, H-Ph), 5.97 (q, *J*=1.9 Hz, 1H, H-4), 4.16 (q, *J*=1.9 Hz, 2H, H-1) ppm. **^{13}C-NMR** (100 MHz, $CDCl_3$, 25 °C): *δ* 151.3 (C-3), 129.4 (C-Ph), 129.2 (×2, C-Ph), 126.5 (×2, C-Ph), 82.3 (C-4), 20.7 (C-1) ppm (2 quaternary carbons not visible).

Compound 23a

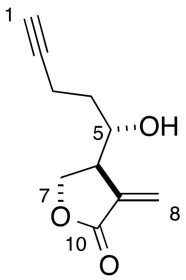

Following general procedure J, aldehyde **S-19** (5.6 mg, 68 µmol, 1.0 equiv) and bromolactone **22a** (13 mg, 75 µmol, 1.1 equiv) gave, after purification by flash chromatography (SiO_2, 80/20 pentane/EtOAc) alcohol **23a** as a yellow oil (7 mg, 39 µmol, 57%). **R$_f$**=0.14 (50/50 petroleum ether/EtOAc). **^{1}H-NMR** (400 MHz, $CDCl_3$, 25 °C): *δ* 6.40 (d, *J*=2.3 Hz, 1H, H-8), 5.83 (d, *J*=2.3 Hz, 1H, H-8), 4.41 (dd, *J*=9.5, 8.0 Hz, 1H, H-7), 4.26 (dd, *J*=9.5, 3.6 Hz, 1H, H-7), 3.92-3.86 (m, 1H, H-5), 3.20-3.14 (m, 1H, H-6), 2.24-2.38 (m, 2H, H-3), 2.20 (d, *J*=4.6 Hz, 1H, OH), 2.02 (t, *J*=2.7 Hz, 1H, H-1), 1.69 (q, *J*=6.6 Hz, 2H, H-4) ppm. **^{13}C-NMR** (100 MHz, $CDCl_3$, 25 °C): *δ* 170.5 (C-10), 134.9 (C-9), 124.9 (C-8), 83.2 (C-2), 72.0

(C-5), 69.8 (C-1), 67.7 (C-7), 44.5 (C-6), 31.8 (C-4), 15.0 (C-3) ppm. **HR-MS (+ESI)** $[M+Na]^+$: calcd: 203.0679, found: 203.0679.

Compound 23b

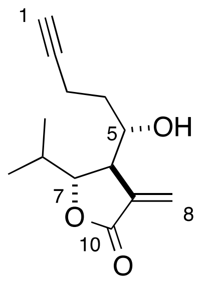

Following general procedure J, aldehyde **S-19** (21 mg, 0.26 mmol, 1.0 equiv) and bromolactone **22b** (163 mg, 0.29 mmol, 1.1 equiv) gave, after purification by flash chromatography (SiO_2, 90/10 to 70/30 pentane/EtOAc), alcohol **23b** as a colorless oil (30 mg, 0.13 mmol, 50%). $\mathbf{R_f}$=0.52 (50/50 petroleum ether/EtOAc). **^{1}H-NMR** (400 MHz, $CDCl_3$, 25 °C): δ 6.37 (d, J=2.0 Hz, 1H, H-8), 5.73 (d, J=2.0 Hz, 1H, H-8), 4.24 (dd, J=5.7, 2.5 Hz, 1H, H-7), 3.84-3.78 (m, 1H, H-5), 2.88-2.85 (m, 1H, H-6), 2.42-2.38 (m, 2H, H-3), 2.06 (d, J=4.6 Hz, OH), 2.01 (t, J=2.5 Hz, 1H, H-1), 1.89-1.81 (m, 1H, C*H*-*i*Pr), 1.72-1.64 (m, 2H, H-4), 0.97 (d, J=5.2 Hz, 3H, CH_3-*i*Pr), 0.95 (d, J=5.2 Hz, 3H, CH_3-*i*Pr) ppm. **^{13}C-NMR** (100 MHz, $CDCl_3$, 25 °C): δ 135.7 (C-9), 124.5 (C-8), 84.4 (C-7), 83.2 (C-2), 72.1 (C-5), 69.6 (C-1), 47.7 (C-6), 33.2 (*C*H-*i*Pr), 31.7 (C-4), 18.0 (*C*H_3-*i*Pr), 17.8 (*C*H_3-*i*Pr), 15.3 (C-3) ppm (one quaternary carbon not visible). **HR-MS (+ESI)** $[M+Na]^+$: calcd: 245.1148, found: 245.1149.

Compound 23c

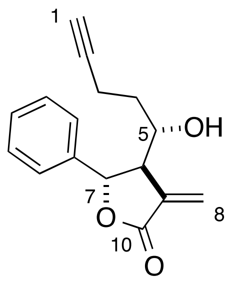

Following general procedure J, aldehyde **S-19** (22 mg, 0.27 mmol, 1.0 equiv) and bromolactone **22c** (75 mg, 0.30 mmol, 1.0 equiv) gave, after purification by flash chromatography (SiO_2, 90/10 to 70/30 pentane/EtOAc), alcohol **23c** as a colorless oil (47 mg, 0.18 mmol, 67%). $\mathbf{R_f}$=0.52 (50/50 petroleum ether/EtOAc). **^{1}H-NMR** (400 MHz, $CDCl_3$, 25 °C): δ 7.40-7.27 (m, 5H, H-Ph), 6.47 (d, J=2.1 Hz, 1H, H-8), 5.79 (d, J=2.1 Hz, 1H, H-8), 5.47 (d, J=2.9 Hz, 1H, H-7), 4.06-4.01 (m, 1H, H-5), 3.14-3.11 (m, 1H, H-6), 2.44-2.40 (m, 2H, H-3), 2.19 (bd, J=5.1 Hz, 1H, OH), 2.02 (t, J=2.6 Hz, 1H, H-1), 1.81-1.75 (m, 2H, H-4) ppm. **^{13}C-NMR** (100 MHz, $CDCl_3$, 25 °C): δ 140.0 (C-Ph), 134.6 (C-9), 128.9 (×2, C-Ph), 128.5 (C-Ph), 125.5 (C-8), 125.3 (×2, C-Ph), 83.2 (C-2), 80.4 (C-7), 72.1 (C-5), 69.8 (C-1), 53.3 (C-6), 31.7 (C-4), 15.2 (C-3) ppm (one quaternary carbon not visible). **HR-MS (+ESI)** $[M+Na]^+$: calcd: 279.0992, found: 279.0993.

Compound 24a

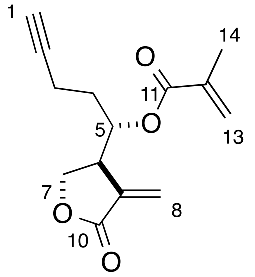

Following general procedure G, alcohol **23a** (7 mg, 39 µmol, 1.0 equiv) gave, after purification by flash chromatography (SiO_2, 80/20 pentane/Et_2O), ester **24a** as a yellow oil (2.2 mg, 8.9 µmol, 23%). **R$_f$**=0.56 (75/25 petroleum ether/EtOAc). **^{1}H-NMR** (400 MHz, $CDCl_3$, 25 °C): δ 6.42 (d, J=2.2 Hz, 1H, H-8), 6.10 (bs, 1H, H-13), 5.79 (d, J=2.2 Hz, 1H, H-8), 5.62 (bs, 1H, H-13), 5.27-5.23 (m, 1H, H-5), 4.37 (dd, J=9.5, 7.6 Hz, 1H, H-7), 4.30 (dd, J=9.5, 3.3 Hz, 1H, H-7), 3.52-3.45 (m, 1H, H-6), 2.31-2.23 (m, 2H, H-3), 1.99 (t, J=3.1 Hz, 1H, H-1), 1.93 (bs, 3H, H-14), 1.92-1.77 (m, 2H, H-4) ppm. **^{13}C-NMR** (100 MHz, $CDCl_3$, 25 °C): δ 126.7 (C-9), 125.1 (C-12), 82.4 (C-2), 74.0 (C-5), 69.6 (C-1), 67.3 (C-7), 41.8 (C-6), 28.8 (C-4), 18.2 (C-14), 14.9 (C-3) ppm (3 quarternary carbons not visible). **HR-MS (+ESI)** $[M+Na]^+$: calcd: 271.0941, found: 271.0941.

Compound 24b

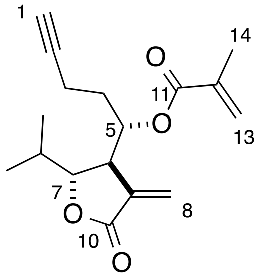

Following general procedure G, alcohol **23b** (17 mg, 77 µmol, 1.0 equiv) gave, after purification by flash chromatography (SiO_2, 80/20 pentane/Et_2O), ester **24b** as a yellow oil (5.5 mg, 19 µmol, 25%). **R$_f$**=0.76 (75/25 petroleum ether/EtOAc). **^{1}H-NMR** (400 MHz, $CDCl_3$, 25 °C): δ 6.39 (d, J=2.0 Hz, 1H, H-8), 6.10 (bs, 1H, H-13), 5.78 (d, J=2.0 Hz, 1H, H-8), 5.62 (bs, 1H, H-13), 5.16 (dt, J=9.9, 4.2 Hz, 1H, H-5), 4.22 (dd, J=6.1, 2.3 Hz, 1H, H-7), 3.24 (dq, J=4.2, 2.3 Hz, 1H, H-6), 2.30-2.22 (m, 2H, H-3), 1.98 (t, J=2.6 Hz, 1H, H-1), 1.93 (bs, 3H, H-14), 1.95-1.75 (m, 1H, H-4), 1.82-1.75 (m, 1H, C*H*-*i*Pr), 0.92 (d, J=6.8 Hz, 3H, CH_3-*i*Pr), 0.89 (d, J=6.8 Hz, 3H, CH_3-*i*Pr) ppm. **^{13}C-NMR** (100 MHz, $CDCl_3$, 25 °C): δ 135.6 (C-9), 135.0 (C-12), 126.6 (C-13), 124.7 (C-8), 83.7 (C-7), 74.6 (C-5), 69.6 (C-1), 44.3 (C-6), 33.2 (*C*H-*i*Pr), 28.4 (C-4), 18.2 (C-14), 17.5 (*C*H_3-*i*Pr), 17.0 (*C*H_3-*i*Pr), 15.1 (C-3) ppm (2 quaternary carbons not visible). **HR-MS (+ESI)** $[M+Na]^+$: calcd: 313.1410, found: 313.1410.

Compound 24c

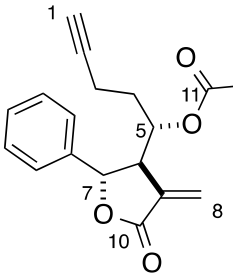

Following general procedure G, alcohol **23c** (30 mg, 0.12 mmol, 1.0 equiv) gave, after purification by flash chromatography (SiO_2, 80/20 pentane/Et_2O), ester **24c** as a yellow oil (5.0 mg, 15 µmol, 13%). **R$_f$**=0.67 (75/25 petroleum ether/EtOAc). **^{1}H-NMR** (400 MHz, $CDCl_3$, 25 °C): *δ* 7.38-7.31 (m, 3H, H-Ph), 7.22-7.20 (m, 2H, H-Ph), 6.49 (d, *J*=2.2 Hz, 1H, H-8), 6.10 (bs, 1H, H-13), 5.81 (d, *J*=2.2 Hz, 1H, H-8), 5.63 (bs, 1H, H-13), 5.45 (d, *J*=3.1 Hz, 1H, H-7), 5.39 (dt, *J*=9.7, 3.9 Hz, 1H, H-5), 3.43-3.40 (m, 1H, H-6), 2.37-2.22 (m, 2H, H-3), 1.99 (t, *J*=2.7 Hz, 1H, H-1), 1.93 (bs, 3H, H-14), 2.07-1.83 (m, 2H, H-4) ppm. **^{13}C-NMR** (100 MHz, $CDCl_3$, 25 °C): *δ* 139.5 (C-Ph), 135.6 (C-9), 133.8 (C-12), 129.0 (×2, C-Ph), 128.7 (C-Ph), 126.7 (C-13), 125.5 (C-8), 125.3 (×2, Ph), 82.2 (C-2), 80.0 (C-7), 73.8 (C-5), 69.7 (C-1), 50.5 (C-6), 29.0 (C-4), 18.3 (C-14), 15.1 (C-5) ppm (2 quaternary carbons visible). **HR-MS (+ESI)** $[M+Na]^+$: calcd: 347.1254, found: 347.1254.

Compound S-23

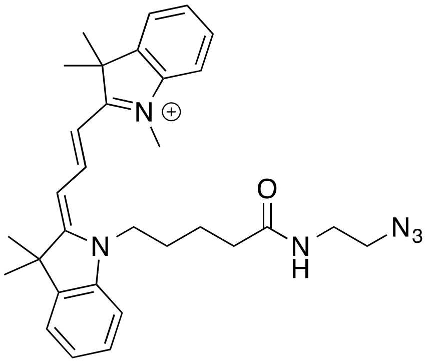

EDC (54 mg, 0.35 mmol, 1.0 equiv) and HOBt (54 mg, 0.35 mmol, 1.0 equiv) were added to a solution of Cyanine 3 carboxylic acid derivative **S-22** (200 mg, 0.35 mmol, 1.0 equiv) in CH_2Cl_2 (4 mL) at room temperature. After 20 minutes, 2-azidoethan-1-amine (30 mg, 0.35 mmol, 1.0 equiv) was added. After 2 hours, the reaction was quenched with 1 M aqueous HCl. The organic phase was then washed with sat. aqueous $NaHCO_3$ and brine, and dried over Na_2SO_4 and filtered. Evaporation of the solvent yielded azide **S-23** as a red powder (140 mg, 0.25 mmol, 73%). **^{1}H-NMR** (400 MHz, $CDCl_3$, 25 °C): *δ* 8.43 (t, *J*=13.4 Hz, 1H), 7.52-7.10 (m, 10H), 4.20-4.16 (m, 2H), 3.84 (s, 3H), 3.52-3.48 (m, 4H), 2.65 (t, J=6.7Hz, 2H), 1.99-1.86 (m, 4H), 1.72 (s, 6H), 1.71 (s, 6H) ppm (NH signal not visible). **LC-MS (ESI$^+$)**: m/z calculated for $C_{31}H_{39}N_6O^+$ $[M]^+$: 511.32; found 511.33.

Compound 24c-Cy3

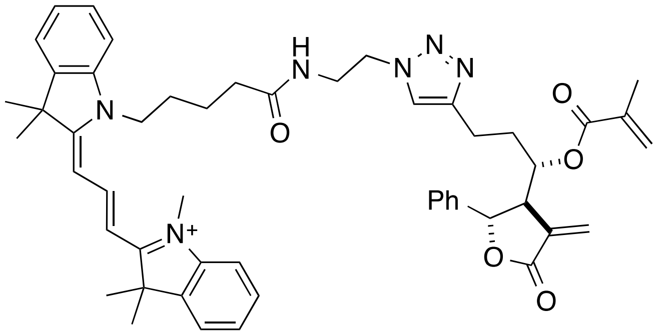

CuBr (1.5 mg, 10 µmol, 0.5 equiv) and TBTA (5.3 mg, 10 µmol, 0.5 equiv) were added to a solution of azide **S-23** (11.2 mg, 21 µmol, 1.0 equiv) and alkyne **24c** (7.0 mg, 21 µmol, 1.0 equiv) in *t*BuOH/H_2O (2/1, 0.75 mL) at room temperature. After 3 hours, water is added and the mixture lyophilized. Purification by flash chromatography (Reverse phase silica gel, 50/50 MeCN/H_2O to MeCN) yielded probe **24c-Cy3** as a red solid (12 mg, 14 µmol, 67%). **^{1}H-NMR** (400 MHz, CD_3CN, 25 °C): δ 8.46 (t, *J*=13.4 Hz, 1H), 7.59-7.28 (m, 16H), 6.63-6.60 (m, 1H), 6.35 (s, 1H), 6.03 (s, 1H), 5.82 (s, 1H), 5.63 (s, 1H), 5.50 (s, 1H), 5.31-5.26 (m, 1H), 4.41-4.38 (m, 2H), 4.06-4.02 (m, 2H), 3.58 (s, 5H), 3.42 (bs, 1H), 2.74-1.69 (m, 8H), 1.87 (s, 3H), 1.73 (s, 6H), 1.72 (s, 6H) ppm. **HR-MS (+ESI)** $[M]^+$: calcd: 835.4541, found: 835.4533.

Synthesis of compounds 26 and 28

Compound S-25

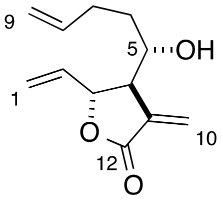

Following general procedure J, aldehyde **25** (160 mg, 1.9 mmol) gave alcohol **S-25** as a yellow oil (139 mg, 0.67 mmol, 35%). **R_f**=0.10 (cyclohexane/EtOAc 1:1). **^{1}H-NMR** (400 MHz, $CDCl_3$, 25 °C): δ 6.41 (d, *J* = 2.4 Hz, 1H, H-10), 5.90-5.78 (m, 2H, H-2, H-8), 5.77 (d, *J* = 2.0 Hz, 1H, H-10), 5.38 (ddt, *J* = 17.1, 9.6, 1.2 Hz, 1H, H-1), 5.25 (ddt, *J* = 10.4, 5.5, 1.1 Hz, 1H, H-1), 5.08 (dq, *J* = 17.1, 1.6 Hz, 1H, H-9), 5.03 (dq, *J* = 10.0, 1.3 Hz, 1H, H-9), 4.86 (ddd, *J* = 6.0, 3.2, 1.6 Hz, 1H, H-3), 3.76 (q, *J* = 6.1 Hz, 1H, H-5), 2.90-2.81 (m, 1H, H-4), 2.37-2.12 (m, 2H, H-7), 1.68-1.58 (m, 2H, H-6) ppm. **^{13}C-NMR** (100 MHz, $CDCl_3$, 25 °C): δ 169.9 (C-12), 137.5 (C-8), 135.8 (C-2), 134.7 (C-11), 125.2 (C-10), 117.31 (C-1), 115.9 (C-9), 79.7 (C-3), 72.3 (C-5), 50.8 (C-4), 32.7 (C-6), 30.1 (C-7) ppm.

Compound S-26

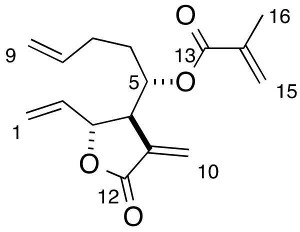

Following general procedure E with alcohol **S-25** (139 mg, 0.67 mmol), methacrylate **S-26** was obtained as a mixture of diastereomers as a pale yellow oil (121 mg, 0.439 mmol, 66%). $\mathbf{R_f}$=0.59 (cyclohexane/EtOAc 2:1). **^{1}H-NMR** (400 MHz, $CDCl_3$, 25 °C): δ 6.42 (d, J = 2.2 Hz, 1H, H-10), 6.09 (d, J = 5.4 Hz, 1H, H-15), 5.84-5.76 (m, 1H, H-8), 5.74 (dq, J = 4.5, 2.9, 2.2 Hz, 1H, H-2), 5.61 (t, J = 1.7 Hz, 1H, H-15), 5.34 (d, J = 17.0 Hz, 1H, H-9), 5.23 (d, J = 10.3 Hz, 1H, H-9), 5.20 (d, J = 4.8 Hz, 1H, H-3), 5.03 (d, J = 8.2 Hz, 1H, H-1), 5.00 (d, J = 1.6 Hz, 1H, H-1), 4.88 (dd, J = 6.2, 3.2 Hz, 1H, H-5), 3.14 (dq, J = 5.0, 2.3 Hz, 1H, H-4), 2.10 (tp, J = 16.0, 8.5, 7.7 Hz, 2H, H-7), 1.93 (d, J = 1.4 Hz, 3H, H-16), 1.90-1.77 (m, 1H, H-6), 1.731.60 (m, 1H, H-6) ppm. **^{13}C-NMR** (100 MHz, $CDCl_3$, 25 °C): δ 169.5 (C-12), 166.7 (C-13), 136.7 (C-8), 135.7 (C-14), 135.4 (C-2), 133.9 (C-11), 126.5 (C-15), 125.2 (C-8), 117.5 (C-9), 115.9 (C-1), 79.3 (C-3), 74.0 (C-5), 48.1 (C-4), 29.7 (C-7), 29.3 (C-6), 18.3 (C-16) ppm.

Compound 26

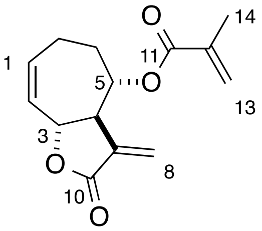

Following general procedure H with diene **S-26** (54 mg, 0.20 mmol), cyclic **26** was obtained as a pale yellow oil (40 mg, 0.16 mmol, 82%). $\mathbf{R_f}$=0.53 (cyclohexane/EtOAc 2:1). **^{1}H-NMR** (400 MHz, $CDCl_3$, 25 °C): δ 6.20 (d, J = 3.4 Hz, 1H, H-8), 6.16 (t, J = 1.2 Hz, 1H, H-13), 6.09-6.00 (m, 1H, H-1), 5.89 (dddd, J = 10.9, 6.6, 4.1, 2.6 Hz, 1H, H-2), 5.68-5.64 (m, 1H, H-13), 5.58 (d, J = 3.1 Hz, 1H, H-8), 5.31 (dt, J = 9.1, 4.3 Hz, 1H, H-5), 4.90-4.83 (m, 1H, H-3), 3.33 (tt, J = 10.0, 3.2 Hz, 1H, H-4), 2.50-2.40 (m, 1H, H-7), 2.21 (dddd, J = 16.3, 8.4, 4.3, 1.5 Hz, 1H, H-7), 2.04 (dddd, J = 15.0, 10.9, 4.2, 1.8 Hz, 1H, H-6), 1.98 (dd, J = 1.6, 0.9 Hz, 3H, H-14), 1.92 (pt, J = 2.7, 1.0 Hz, 1H, H-6) ppm. **^{13}C-NMR** (100 MHz, $CDCl_3$, 25 °C): δ 169.2 (C-10), 166.3 (C-11), 137.6 (C-9), 136.0 (C-12), 131.8 (C-2), 129.7 (C-1), 126.5 (C-13), 121.6 (C-8), 76.5 (C-3), 74.2 (C-5), 49.7 (C-4), 31.2 (C-6), 23.1 (C-7), 18.2 (C-14) ppm. **HR-MS (+ESI)** $[M+Na]^+$: calcd: 271.0941, found: 271.0943.

Compound S-27

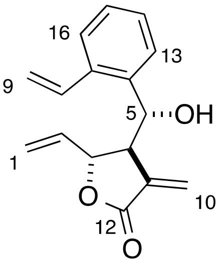

Following general procedure J, aldehyde **27** (192 mg, 1.45 mmol) gave alcohol **S-25** as a yellow oil (60 mg, 0.23 mmol, 17%). $\mathbf{R_f}$=0.22 (cyclohexane/EtOAc 2:1). **^{1}H-NMR** (400 MHz, $CDCl_3$, 25 °C): δ 7.52-7.47 (m, 1H, H-Ar), 7.44-7.40 (m, 1H, H-Ar), 7.36-7.30 (m, 2H, H-Ar), 7.05 (dd, J = 17.3, 11.0 Hz, 1H, H-8), 6.36 (dd, J = 2.4, 0.8 Hz, 1H, H-

10), 5.68-5.62 (m, 2H, H-9, H-10), 5.56 (ddd, J = 16.8, 10.7, 6.1 Hz, 1H, H-2), 5.39 (dd, J = 11.0, 1.3 Hz, 1H, H-9), 5.12-4.99 (m, 3H, H-1, H-5), 4.64 (ddt, J = 6.3, 4.0, 1.3 Hz, 1H, H-3), 3.16 (ddt, J = 7.3, 4.1, 2.2 Hz, 1H, H-4) ppm. **^{13}C-NMR** (100 MHz, $CDCl_3$, 25 °C): δ 170.0 (C-12), 137.4 (C-11), 136.4 (C-Ar), 135.1 (C-2), 134.4 (C-Ar), 133.7 (C-8), 128.6 (C-Ar), 128.0 (C-Ar), 126.9 (C-Ar), 126.9 (C-Ar), 126.1 (C-10), 118.0 (C-9), 117.0 (C-1), 79.7 (C-3), 71.8 (C-5), 50.8 (C-4) ppm.

Compound S-26

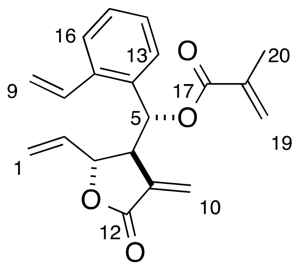

Following general procedure E with alcohol **S-27** (60 mg, 0.23 mmol), methacrylate **S-28** was obtained as a pale yellow oil (33 mg, 0.10 mmol, 44%). **R_f**=0.57 (cyclohexane/EtOAc 2:1). **^{1}H-NMR** (400 MHz, $CDCl_3$, 25 °C): δ 7.55-7.43 (m, 1H, H-Ar), 7.38-7.27 (m, 3H, H-Ar), 7.16 (dd, J = 17.2, 10.9 Hz, 1H, H-8), 6.33 (d, J = 2.2 Hz, 1H, H-10), 6.24-6.17 (m, 2H, H-5, H-19), 5.72-564 (m, 2H, H-9, H-19), 5.57 (ddd, J = 16.8, 10.4, 6.0 Hz, 1H, H-2), 5.45 (dd, J = 10.9, 1.3 Hz, 1H, H-9), 5.35 (d, J = 1.9 Hz, 1H, H-10), 5.10-5.04 (m, 2H, H-1), 4.66 (ddt, J = 6.1, 3.1, 1.4 Hz, 1H, H-3), 3.29 (ddd, J = 7.3, 3.6, 1.9 Hz, 1H, H-4), 1.95 (t, J = 1.3 Hz, 3H, H-20) ppm. **^{13}C-NMR** (100 MHz, $CDCl_3$, 25 °C): δ 169.6 (C-12), 165.9 (C-17), 136.6 (C-Ar), 135.6 (C-Ar), 134.8 (C-2), 134.0 (C-Ar), 133.7 (C-8), 133.5 (C-11), 128.8 (C-Ar), 127.9 (C-Ar), 127.0 (C-Ar), 126.8 (C-19), 126.7 (C-18), 126.3 (C-10), 118.4 (C-9), 117.2 (C-1), 79.4 (C-3), 73.2 (C-5), 49.3 (C-4), 18.2 (C-20) ppm.

Compound 28

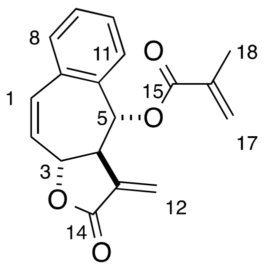

Following general procedure H with diene **S-28** (25 mg, 77 µmol), cyclic **28** was obtained as a pale yellow oil (8 mg, 27 µmol, 35%). **R_f**=0.53 (cyclohexane/EtOAc 2:1). **^{1}H-NMR** (400 MHz, $CDCl_3$, 25 °C): δ 7.46-7.39 (m, 1H, H-Ar), 7.38-7.27 (m, 2H, H-Ar), 7.20 (dd, J = 7.6, 1.7 Hz, 1H, H-Ar), 6.67 (d, J = 9.3 Hz, 1H, H-5), 6.49 (dd, J = 11.5, 2.5 Hz, 1H, H-1), 6.34 (dd, J = 11.5, 3.1 Hz, 1H, H-2), 6.31 (d, J = 3.5 Hz, 1H, H-12), 6.16-6.14 (m, 1H, H-17), 5.70 (d, J = 3.1 Hz, 1H, H-12), 5.66 (p, J = 1.6 Hz, 1H, H-17), 4.91 (dt, J = 10.3, 2.7 Hz, 1H, H-3), 3.78 (ddt, J = 10.4, 9.4, 3.3 Hz, 1H, H-4), 1.97 (t, J = 1.2 Hz, 3H, H-18) ppm. **^{13}C-NMR** (100 MHz, $CDCl_3$, 25 °C): δ 169.3 (C-14), 166.7 (C-15), 137.1 (C-Ar), 136.5 (C-13), 135.8 (C-16), 133.9 (C-Ar), 132.8 (C-Ar), 131.0 (C-Ar), 130.4 (C-2), 129.5 (C-1), 128.7 (C-q), 128.7 (C-Ar), 128.5 (C-Ar), 127.1 (C-17), 122.6 (C-12), 75.9 (C-3), 75.7 (C-5), 51.1 (C-4), 18.3 (C-18) ppm. **HR-MS (+ESI)** $[M+Na]^+$: calcd: 319.0941, found: 319.0940.

Comparison of the coupling constants for Nordeoxyelephantopin and Deoxyelephantopin. The comparison between the coupling constants in Nordeoxyelephantopin and Deoxyelephantopin suggests that both have very similar conformations in solution, as coupling constants are directly correlated to dihedral angles, and hence conformation, via the Karplus equation. Differences are observed around olefin $\Delta^{4,5}$ and may be attributed to allylic strain with H^6 and steric repulsion of the butenolide across the ring imposed by the olefinic methyl group in Deoxyelephantopin, absent in Nordeoxyelephantopin.

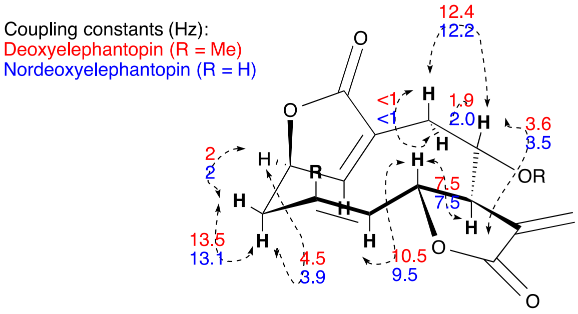

NOESY correlations for Deoxyelephantopin and Nordeoxyelephantopin. Clear NOESY correlations between protons on the same face (alpha face in red and, beta face in blue) are observed, confirming the similarity in the conformation of both compounds (Deoxyelephantopin and Nordeoxyelephantopin).

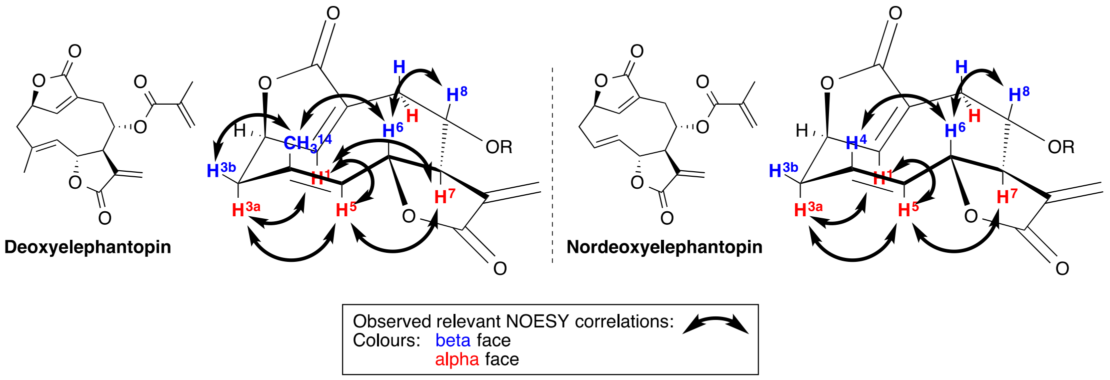

Qualitative reactivity ranking of the Michael acceptors in Nordeoxyelephantopin with Glutathione by ^{1}H-NMR. Deuterated PBS salts were obtained by five cycles or dissolution of the salts in D_2O and lyophilisation. The salts thus obtained were dissolved in D_2O to form a 0.33X deuterated PBS buffer (dPBS hereafter), which was used to prepare

all the other solutions. Exchange of the polar protons of commercially available reduced glutathione was achieved by five cycles or dissolution in D_2O and lyophilisation. The resulting deuterated glutathione was then redissolved in dPBS to a 50 mM stock solution. Commercially available NaOCHO was used directly as an internal standard as a 100 mM stock solution in dPBS. Synthetic Nordeoxyelephantopin was dissolved in DMSO-d_6 (1 M) and added to dPBS to form a 2 mM stock solution.

In an NMR tube, to a solution of Nordeoxyelephantopin (300 μL) were successively added NaOCHO (60 μL), dPBS (180 μL) and glutathione (60 μL). The resulting solution (600 μL; noredeoxyelephantopin 1 mM, GSH 5 mM, NaOCHO 10 mM) was vortexed and incubated at room temperature. ^{1}H NMR measurements were performed at different time points.

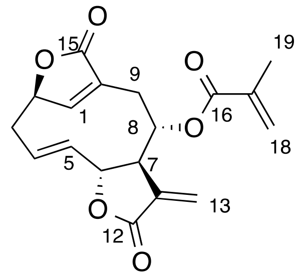

A ^{1}H NMR spectrum was recorded prior to addition of GSH, with NaOCHO as internal standard. **^{1}H-NMR** (400 MHz, D_2O+DMSO-d_6, 25 °C): δ 7.50 (s, 1H, H-1), 6.24 (d, J = 3.6 Hz, 1H, H-13), 6.21 (s, 1H, H-18), 5.88 (d, J = 3.2 Hz, 1H, H-13), 5.79 (s, 1H, H-18), 5.59-5.55 (m, 1H, H-2), 5.51 (m, 2H, H-5, H-4), 4.94 (dd, J = 9.0, 7.3 Hz, 1H, H-6), 4.57 (dt, J = 11.7, 3.6, 2.6 Hz, 1H, H-8), 3.37 (dq, J = 7.2, 3.5 Hz, 1H, H-7), 3.04 (dd, J = 12.8, 11.7 Hz, 1H, H-9), 2.96-2.83 (m, 2H, H-3, H-9), 2.63 (m, 1H, H-3), 1.91 (s, 3H, H-19) ppm. (H-14 omitted to respect the original assignment for Deoxyelephantopin).

^{1}H NMR measurements were performed at different time points showed rapid consumption of nordeoxyelephantopin upon addition of glutathione. A single product was formed resulting from a single thia-Michael addition of glutathione at the α-*exo*-methylene-γ-butyrolactone, even upon prolonged exposure to excess glutathione (*vide infra*). **LC-MS** (H_2O:MeCN, 0.1% TFA) m/z calculated for $C_{28}H_{35}DN_3O_{12}S^+$ $[M+H]^+$: 639.21; found: 639.42.

This allows us to conclude that, despite the ring tension, the *endo* butenolide is completely unreactive towards thia-Michael addition. Surprisingly, the methacrylate side-chain did not participate in the reaction and only the conjugate addition product at the α-*exo*-methylene was formed, suggesting that the latter is the most reactive Michael acceptor.

Structural confirmation: In order to ascertain the structure of the product obtained, the reaction described above was repeated under regular conditions, i.e. non-deuterated buffer and reagents. The single product was purified by reverse phase HPLC (H_2O:MeCN, 0.1% TFA).

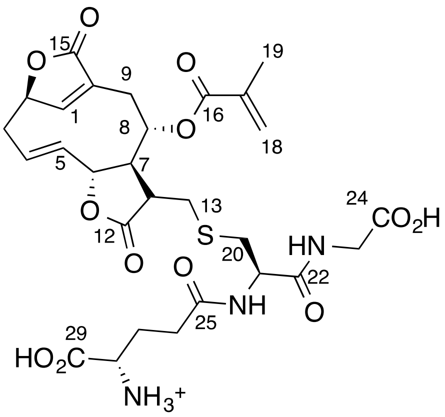

^{1}H-NMR (500 MHz, DMSO-d_6, 25 °C): δ 8.42 (dt, J = 24.5, 6.1 Hz, 1H, N*H*), 8.35 (dd, J = 8.4, 6.4 Hz, 1H, N*H*), 8.24 (s, 3H, NH_3), 7.46 (d, J = 6.5 Hz, 1H, H-1), 6.11 (d, J = 5.2 Hz, 1H, H-18), 5.77 (dt, J = 3.3, 1.7 Hz, 1H, H-18), 5.47 (dt, J = 4.0, 1.9 Hz, 1H, H-2), 5.37 (ddd, J = 15.3, 9.9, 4.8 Hz, 1H, H-4), 5.24 (ddd, J = 16.1, 12.7, 9.5 Hz, 1H, H-5), 5.08 (dtd, J = 10.2, 7.3, 2.6 Hz, 1H, H-8), 4.64 (td, J = 9.2, 2.4 Hz, 1H, H-6), 4.51 (dtd, J = 17.9, 8.5, 8.5, 5.2 Hz, 1H, H-21), 3.95 (bs, 1H, H-28), 3.79 (d, J = 5.8 Hz, 2H, H-23), 3.14-2.91 (m, 3H, H-13, H-20, H-11), 2.86 (ddd, J = 13.4, 4.9, 2.6 Hz, 1H, H-20), 2.83-2.68 (m, 4H, H-13, H-7, H-3, H-9), 2.63-2.59 (m, 1H, H-9), 2.55-2.51 (m, 1H, H-3), 2.45-2.30 (m, 2H, H-26), 2.02 (tq, J = 12.8, 6.4 Hz, 2H, H-27), 1.94 (q, J = 1.3 Hz, 3H, H-19). **^{13}C-NMR** (125 MHz, DMSO-d_6, 25 °C): δ 175.5 (C-29), 172.2 (C-24), 171.0 (C-25), 170.9 (C-22), 170.8 (C-15), 170.4 (C-12), 165.1 (C-16), 155.1 (C-1), 139.3 (C-5), 135.5 (C-17), 128.7 (C-10), 126.7 (C-18), 126.0 (C-4), 81.0 (C-6), 80.1 (C-2), 71.0 (C-8), 52.2 (C-21), 51.64 (C-28), 49.8 (C-7), 44.51 (C-11), 40.8 (C-23), 35.7 (C-20), 33.2 (C-3), 33.0 (C-9), 32.4 (C-13), 30.7 (C-26), 26.0 (C-27), 18.2 (C-19). (C-14 and H-14 omitted to respect the original assignment for Deoxyelephantopin). **LC-MS (H_2O:MeCN, 0.1% TFA)**: m/z calculated for $C_{28}H_{36}N_3O_{12}S^+$ $[M+H]^+$: 638.20; found: 638.28.
